# Weight-loss Independent Clinical and Metabolic Biomarkers Associated with Type 2 Diabetes Remission Post-bariatric/metabolic Surgery

**DOI:** 10.1007/s11695-023-06905-8

**Published:** 2023-11-01

**Authors:** Kusuma Chaiyasoot, Naomi S. Sakai, Roxanna Zakeri, Janine Makaronidis, Luís Crisóstomo, Marco G. Alves, Wei Gan, Chloe Firman, Friedrich C. Jassil, Margaret A. Hall-Craggs, Stuart A. Taylor, Rachel L. Batterham

**Affiliations:** 1 Department of Medicine, Centre for Obesity Research, University College London, London, UK; 2 Division of Nutrition, Department of Medicine, Faculty of Medicine Siriraj Hospital, Mahidol University, Bangkok, Thailand; 3 The Siriraj Center of Research Excellence for Diabetes and Obesity (SiCORE-DO), Mahidol University, Bangkok, Thailand; 4 UCL Centre for Medical Imaging, London, UK; 5 National Institute of Health Research, University College London Hospitals Biomedical Research Centre, London, UK; 6 Department of Immunophysiology and Pharmacology, ICBAS - School of Medicine and Biomedical Sciences, UMIB - Unit for Multidisciplinary Research in Biomedicine, University of Porto, Porto, Portugal; 7 ITR - Laboratory for Integrative and Translational Research in Population Health, Porto, Portugal; 8 Institute of Biomedicine, University of Turku, Turku, Finland; 9 Genetics Department, Novo Nordisk Research Centre Oxford, Innovation Building, Old Road Campus, Headington, OX37LQ UK

**Keywords:** Type 2 diabetes, Remission, Obesity, Metabolic surgery

## Abstract

**Purpose:**

Remission of type 2 diabetes (T2D) can be achieved by many, but not all, people following bariatric/metabolic surgery. The mechanisms underlying T2D remission remain incompletely understood. This observational study aimed to identify novel weight-loss independent clinical, metabolic and genetic factors that associate with T2D remission using comprehensive phenotyping.

**Materials and Methods:**

Ten patients without T2D remission (non-remitters) were matched to 10 patients with T2D remission (remitters) for age, sex, type of surgery, body weight, BMI, post-operative weight loss, duration from surgery and duration of T2D. Detailed body composition assessed using magnetic resonance imaging, gut hormones, serum metabolomics, insulin sensitivity, and genetic risk scores for T2D and anthropometric traits were assessed.

**Results:**

Remitters had significantly greater β-cell function and circulating acyl ghrelin levels, but lower visceral adipose tissue (VAT): subcutaneous adipose tissue (SAT) ratio than non-remitters. Branched-chain amino acids (BCAAs) and VLDL particle size were the most discriminant metabolites between groups. A significant positive correlation between, VAT area, VAT:SAT ratio and circulating levels of BCAAs was observed, whereas a significant negative correlation between BCAAs and β-cell function was revealed.

**Conclusion:**

We highlight a potentially novel relationship between VAT and BCAAs, which may play a role in glucoregulatory control. Improvement in β-cell function, and the role ghrelin plays in its recovery, is likely another key factor influencing T2D remission post-surgery. These findings suggest that adjunctive approaches that target VAT loss and restoration of BCAA metabolism might achieve higher rates of long-term T2D remission post-surgery.

**Graphical Abstract:**

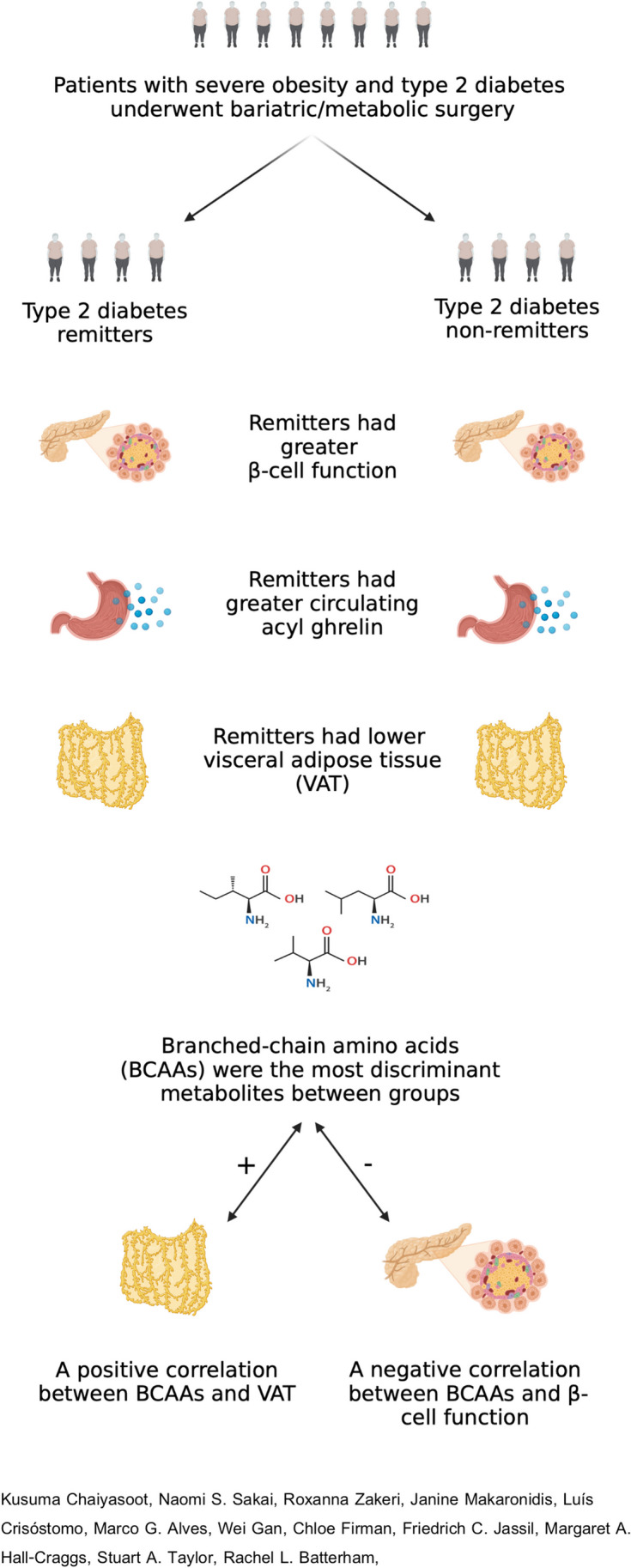

**Supplementary Information:**

The online version contains supplementary material available at 10.1007/s11695-023-06905-8.

## Introduction

Compared to lifestyle intervention and pharmacotherapy, bariatric/metabolic surgery is currently the most effective treatment for people living with severe obesity. Bariatric/metabolic surgery provides other benefits beyond weight reduction; in patients with T2D a remarkable reduction in glycaemia is often observed post-surgery, typically before any weight loss occurs [1]. Bariatric/metabolic surgery results in T2D remission in 70% of patients at 1-year post-surgery [2].

However, the rate of T2D remission post-surgery decreases to 45% at 5 years [3] and 36% at 10 years [4] and a significant number of patients do not achieve post-operative T2D remission at all [2]. As a result, research has focused on identifying predictors of T2D remission induced by bariatric/metabolic surgery. A number of clinical factors including age, T2D duration, pre-operative use of anti-hyperglycaemic medication, HbA_1c_, c-peptide levels, post-operative weight loss and type of bariatric/metabolic surgery have been reported to be independent determinants of T2D remission after bariatric/metabolic surgery [5, 6].

The mechanisms underlying suboptimal metabolic responses and variation of T2D remission status following bariatric/metabolic surgery in some patients remain to be answered. There is a paucity of data comparing people with and without T2D remission post-surgery. Hence, this study aimed to identify novel post-operative weight-loss independent clinical and metabolic biomarkers associated with the heterogeneity between patients who did not achieve T2D remission (non-remitters) and matched individuals who achieved partial or complete T2D remission (remitters). Detailed body composition, β-cell function, insulin sensitivity, gut hormones, systemic metabolomics, and genetic risk scores for T2D and anthropometric trait were compared between groups. A comprehensive understanding of the factors that may influence post-operative T2D remission is crucial to optimise patient outcomes and improve clinical management.

## Materials and Methods

### Subjects

This study was approved by the National Health Service Research Ethics Committee (ID#09/H0715/65). All subjects (age ≥ 18 years) underwent primary bariatric/metabolic surgery at the University College London Hospitals (UCLH) Bariatric Centre for Weight Management and Metabolic Surgery and had T2D at the time of surgery. After surgery, the definition of complete remission was normal HbA_1c_ levels (< 5.7% [39 mmol/mol]) and fasting plasma glucose (FPG) < 5.6 mmol/l for ≥ 1 year without active pharmacotherapy and partial remission was HbA_1C_ levels at 5.7 – 6.4% (39–46 mmol/mol) and FPG at 5.6–6.9 mmol/l for ≥ 1 year without active pharmacotherapy [7].

Non-remitters were identified from an electronic database of patients at the UCLH Bariatric Centre for Weight Management and Metabolic Surgery. We then identified subjects who achieved partial or complete remission and were matched to the non-remitters for age, sex, type of surgery, body weight (BW), BMI, percentage weight loss (PWL), duration since surgery and duration of T2D from the database.

The exclusion criteria were: use of insulin prior to surgery, current use of glucagon-like peptide-1 (GLP-1) receptor agonists, active cardiovascular disease, malignant disease, significant renal or hepatic impairment, contraindications to magnetic resonance imaging (MRI) scanning, revisional bariatric/metabolic surgery and presence of hypoglycaemia related to post-bariatric surgery. Written informed consent was given by all subjects.

### Surgical Procedures

Laparoscopic Roux-en-Y gastric bypass (LRYGB) included construction of a bilio-pancreatic limb with a 100- to 120-cm alimentary limb and approximately a 30-ml gastric pouch. Laparoscopic one-anastomosis gastric bypass (LOAGB) involved creation of a 50- to 150- ml gastric pouch with an ante-colic, isoperistaltic gastrojejunal anastomosis and 200 cm bilio-pancreatic limb. Laparoscopic sleeve gastrectomy (LSG) was conducted according to international best practice [8].

### Study Protocol

Subjects attended for a mixed-meal tolerance test (MMTT) at the UCLH from September 2018 to January 2020. Prior to the test day, patients were asked to refrain from alcohol for 24 h and to fast overnight for 12 h. After cannulation, 45 min of acclimatisation was allowed [9]. At time ‘0 min’, subjects consumed 200 ml of the test meal (Resource 2.0 Fibre, Nestle Nutrition, Croydon, UK) consisting of 400 kcal, 18% of protein (22.5 g), 40% carbohydrate (50 g) and 39% fat (21.8 g) within 15 min and a blood sample was collected. Blood samples were then taken repeatedly at 15, 30, 60, 90, 120, 150 and 180 min. Samples were processed strictly according to a previous protocol [9]. After the MMTT, subjects underwent a quantitative MRI scan of the abdomen and pelvis. Individual organ fat (liver, pancreas) and body composition measurements were derived from the quantitative MR images.

### Anthropometric Measurement

BW was measured using a calibrated weighing scale (Seca 877, Seca, UK). Height was measured by a wall-mounted stadiometer (242 Measuring Rod, Seca, UK). Percentage weight loss (PWL) was calculated by the following formula: PWL = ([BW at the time of surgery – BW at the study visit]/ BW at the time of surgery) × 100.

### Magnetic Resonance Imaging (MRI)

See details in Supplementary material for MRI. MRI scans were anonymised and analysed independently by two readers (both radiologists) blinded to the identity of the subjects and T2D remission status. Hepatic, pancreatic and skeletal muscle fat (expressed as PDFF) were quantified. Body composition parameters included the ratio of visceral adipose tissue (VAT) area to subcutaneous adipose tissue (SAT) area (VAT:SAT ratio), and indices of total body fat mass, total body fat free mass and skeletal muscle (adjusted for patient height).

### Hormone Assays

Insulin, active GLP-1, peptide YY (PYY), fibroblast growth factor-19 (FGF-19), acyl ghrelin (AG) and des-acyl ghrelin (DAG) were assayed by ELISA (respectively: sensitivity, 1 µU/Ml, 2 Pm, 6.5 pg/Ml, 1.17 pg/Ml, NA, NA; inter-assay variability, 9.1 – 11.4%, < 1 – 13%, 3.7 – 16.5%, 4.5 – 5.5%, NA, NA; intra-assay variability, 4.6 – 7%, 6 – 9%, 0.9 – 5.78%, 3.6 – 6.4%, NA, NA) (insulin, active GLP-1 and PYY by Millipore, Watford, UK; FGF-19 by Bio-techne, Abingdon, UK; AG and DAG by SCETI K.K., Tokyo, Japan). Area under curve (AUC) of the hormones during a MMTT were produced using the trapezoid rule. An ∆AUC was produced as an AUC calculated by subtracting the fasting (t0) hormone level from every time-point level during the MMTT.

Insulin sensitivity was calculated using QUICKI score (= 1/ (log [fasting plasma insulin] + log [fasting plasma glucose])). Homeostatic model assessment of insulin resistance (HOMA-IR), computed from (fasting plasma glucose x fasting plasma insulin)/ 22.5 in molar units, was used to indicate insulin resistance. HOMA-β (= [20 × fasting plasma insulin] / [fasting plasma glucose – 3.5] in molar units), insulinogenic index [10] (IGI = Δinsulin [30–0 min] [μIU/mL] /Δglucose [30–0 min] [mg/dL]), and oral disposition index [10] (Oral DI = IGI /HOMA-IR) were utilised as indicators of β-cell function.

### Metabolites Quantification

All samples for metabolomics study were analysed by the Nightingale Health Ltd., Helsinki, Finland. A high-throughput nuclear magnetic resonance (NMR) metabolomics platform was utilised to quantify 249 metabolites from nutrient-stimulated plasma samples [11, 12]. This set of metabolic features covers a variety of biomarkers related to multiple metabolic pathways, including comprehensive lipoprotein lipid profiles within 14 subclasses, fatty acids, amino acids, glycolysis related metabolites, ketone bodies, creatinine, albumin and glycoprotein acetyls (GlycA). An AUC of serum metabolites during MMTTs were produced using the trapezoid rule.

### Genetic Risk Score (GRS) Analysis

Genomic DNA was extracted from blood or saliva samples. Genotyping was performed using Illumina HumanCoreExome-24 BeadChip genotyping arrays and imputed with the 1000 Genomes Project. Quality control of genotyping was conducted according to a previous criteria [13]. Six GRSs associated with T2D and anthropometric traits were constructed [14–16] (Supplementary Table S1). The scores were then weighted by variant-specific coefficients from the study. A logistic regression analysis was performed to examine the association between GRSs and T2D remission after surgery.

### Statistical Analysis

Continuous data with normal distribution was expressed as mean ± SD and unpaired t-tests were used for the comparison. Non-normally distributed data was presented as median (25^th^, 75^th^ percentiles) and Mann–Whitney tests were used for the comparison. Categorical variables were reported as percentages and χ^2^ tests were used to compare variables between groups. Linear regression analysis was performed to test an association between parameters. See Supplementary material for metabolomics analysis.

This is an observational exploratory analysis; the exact sample size cannot be predetermined. However, based on a previous study [17], 10 subjects in each group would be sufficient to provide metabolomics differences associated with T2D remission.

## Results

### Anthropometric, Clinical, and Metabolic Features

Ninety-seven subjects were approached and 77 subjects were excluded due to various reasons (Supplementary Figure S1). Ten non-remitters and 10 remitters (4 complete remission and 6 partial remission) were matched for age, sex, BW, BMI, PWL, type of bariatric/metabolic surgery, duration since surgery and pre-operative T2D duration. The majority of subjects were female (Table 1). Seven (70%) in the non-remitters group had a LRYGB with the remaining 3 (30%) who underwent a LSG, whereas 6 (60%) in the remitters group had a LRYGB with 3 (30%) underwent a LSG and 1(10%) had LOAGB. The levels of triglyceride (TG) in non-remitters were significantly greater than remitters (P = 0.03) (Table 1).

**Table 1:** Comparison of patient characteristics, glycaemic, lipid, insulin sensitivity, β-cell function indices, gut hormone profiles and MRI parameters between T2D partial and complete remitters vs. non-remitters. Fat mass index was calculated by fat mass/ height (m)^2^; fat free mass index was calculated by fat free mass/ height (m)^2^; skeletal muscle index was calculated by skeletal muscle area/ height (m)^2^

	Non-remitters (n = 10)	Remitters (n = 10)	*P*-value
Age, years	60.4 (56.8, 62.4)	61.1 (56.8, 65.5)	0.48
Female, %	50	70	0.36
Type of surgery			
- LRYGB, n (%)	7 (70)	6 (60)	0.58
- LSG, n (%)	3 (30)	3 (30)	
- LOAGB, n (%)	0	1 (10)	
Body weight, kg	91.3 ± 13.6	93.7 ± 24.5	0.79
BMI, kg/m^2^	33.6 ± 4.7	34.9 ± 5.6	0.57
Weight loss, %	21.9 ± 7.1	20.1 ± 5.9	0.53
Diabetes duration, years	7.7 ± 2.3	6.7 ± 4.2	0.52
Duration from surgery, years	6.6 ± 2.1	5.1 ± 2.3	0.14
*Glycaemic and lipid indices*			
Glucose, mmol/L	7.43 (6.75, 8.82)	4.85 (4.3, 5.38)	< 0.0001
HbA_1c_, % (mmol/mol)	7.1 ± 0.8 (54.4 ± 8.2)	5.7 ± 0.5 (39 ± 5.5)	0.0001
Cholesterol, mmol/L	4.36 ± 0.66	4.48 ± 0.9	0.78
Triglyceride, mmol/L	1.53 ± 0.44	1.08 ± 0.3	0.03
HDL-c, mmol/L	1.41 ± 0.27	1.54 ± 0.42	0.52
LDL-c, mmol/L	2.13 ± 1.04	2.45 ± 0.69	0.49
*Insulin sensitivity and β-cell function indices*			
Fasting insulin, pM	25.2 ± 19.7	30.9 ± 25.9	0.59
AUC_0-15_ insulin, pM x min	1,623 ± 1,159	3,513 ± 1,066	0.001
AUC_0-30_ insulin, pM x min	6,211 ± 4,044	13,338 ± 4,134	0.001
AUC_0-180_ insulin, pM x min	41,417 ± 19,404	62,686 ± 27,090	0.06
HOMA-β	11.95 (7.94, 21.69)	58.24 (26.52, 90.65)	0.01
Insulinogenic index (IGI)	0.48 (0.26, 0.71)	1.43 (1.05, 2.04)	0.003
Oral disposition index (DI)	0.54 (0.34, 0.94)	2.52 (0.63, 8.34)	0.005
HOMA-IR	1.42 ± 1.31	1.02 ± 0.9	0.43
QUICKI	0.4 (0.33, 0.46)	0.41 (0.35, 0.5)	0.49
*MRI parameters*			
Total fat area, cm^2^	864 ± 296.4	942.6 ± 357	0.60
SAT area, cm^2^	540.7 ± 230.1	742.2 ± 285.2	0.10
VAT area, cm^2^	311 (186, 452)	163 (129, 247)	0.06
VAT:SAT ratio	0.46 (0.36, 1.06)	0.26 (0.2, 0.36)	0.01
Hepatic fat, %	3.72 (3.07, 4.42)	4.44 (3.48, 6.01)	0.19
Pancreatic fat, %	8.15 (7.46, 10.28)	9.07 (7.68, 11.82)	0.57
Fat mass index, kg/m^2^	17.33 ± 4.99	18.74 ± 4.28	0.51
Fat free mass index, kg/m^2^	28.34 ± 4.29	29.08 ± 3.38	0.68
Skeletal muscle index, cm^2^/m^2^	87.12 ± 14.65	89.3 ± 12.18	0.72
Skeletal muscle fat fraction, %	23.22 ± 4.71	24.94 ± 4.35	0.41
*Gut hormone profiles*			
Fasting AG, fmol/mL	5 (3.37, 16.5)	12.59 (5.53, 19.2)	0.09
AUC_0-150_ AG, fmol x min/mL	807 (477, 1,252)	1,556 (1,042, 1,888)	0.03
ΔAUC_0-150_ AG, fmol x min/mL	-401 ± 652	-392 ± 724	0.98
Fasting DAG, fmol/mL	89.8 ± 52.1	105.7 ± 77.1	0.6
AUC_0-150_ DAG, fmol x min/mL	8,135 (5,979, 13,402)	9,306 (5,438, 15,344)	0.91
ΔAUC_0-150_ DAG, fmol x min/mL	-4,264 ± 4,096	-4,594 ± 5,017	0.87
Fasting AG:DAG	0.095 ± 0.043	0.141 ± 0.034	0.02
AUC_0-150_ AG:DAG	0.089 (0.074, 0.112)	0.164 (0.114, 0.213)	0.03
ΔAUC_0-150_ AG:DAG	0.05 (-0.031, 0.297)	0.072 (-0.021, 0.125)	0.91
Fasting PYY, pg/mL	147 ± 82	91 ± 28	0.05
AUC_0-180_ PYY, pg x min/mL	52,099 (41,285, 84,629)	38,459 (33,204, 52,408)	0.09
ΔAUC_0-180_ PYY, pg x min/mL	28,578 (23,800, 47,998)	26,042 (15,584, 31,367)	0.35
Fasting active GLP-1, pM	4.83 (2.05, 8.02)	1.75 (0.87, 4.14)	0.05
AUC_0-180_ active GLP-1, pM x min	4,782 (1,826, 10,249)	4,267 (2,231, 7,483)	0.69
ΔAUC_0-180_ active GLP-1, pM x min	3,155 (1,539, 9,513)	4,143 (1,926, 7,081)	0.8
Fasting FGF-19, pg/mL	103.2 ± 57.1	161.9 ± 100.9	0.13
AUC_0-180_ FGF-19, pg x min/mL	30,597 (19,339, 89,622)	33,572 (21,190, 64,535)	0.95
ΔAUC_0-180_ FGF-19, pg x min/mL	15,473 (4,575, 54,605)	10,025 (5,461, 19,786)	0.48

### Use of Anti-hyperglycaemic Medications

The pre-operative anti-hyperglycaemic medications used in non-remitters were metformin (10/10[100%]), sulfonylurea (4/10[40%]), pioglitazone (3/10[30%]), and GLP-1 receptor agonists (1/10[10%]). In contrast, metformin (6/10[60%]), sulfonylurea (1/10[10%]) and no antidiabetic medication (4/10[40%]) were used in remitters.

At the time of analysis, anti-hyperglycaemic medications used in non-remitters were metformin (7/10[70%]) and sodium-glucose cotransporters-2 inhibitors (1/10 [10%]), whilst in remitters, all medications have been ceased.

### Insulin Sensitivity and β-cell Function Indices

**Fig. 1:**
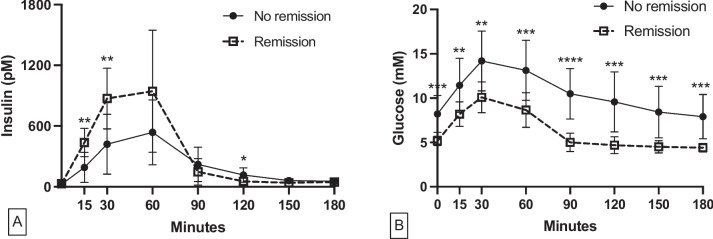
Nutrient-stimulated insulin levels (**A**) and glucose levels (**B**). Results were expressed as mean ± SD. *P < 0.05, **P < 0.01, ***P < 0.001, ****P < 0.0001 of the comparisons between groups

Insulin levels were statistically significantly higher in remitters compared to non-remitters at 15 and 30 min post-meal (Fig. 1A). Accordingly, the levels of AUC_0-15_ and AUC_0-30_ insulin in remitters were significantly greater than non-remitters (P = 0.001 for both, Table 1). Furthermore, the HOMA-β, IGI and oral DI in remitters were significantly greater than in non-remitters (P = 0.01, P = 0.003 and P = 0.005, respectively), (Table 1). The fasting insulin levels, QUICKI index and HOMA-IR were comparable between groups (P = 0.59, P = 0.49 and P = 0.43, respectively Table 1).

### MRI Parameters

Remitters had a significantly lower VAT:SAT ratio compared to non-remitters (P = 0.01, Table 1). Non-remitters had a greater VAT area compared to remitters, although this did not reach statistical significance (P = 0.06). Hepatic and pancreatic fat content was similar in both groups as well as the area of total fat and SAT (Table 1). There was no statistically significant difference between remitters and non-remitters in fat mass (FM) index, fat free mass (FFM) index, skeletal muscle (SM) index and SM fat fraction (Table 1).

### Metabolomics Study

**Fig. 2:**
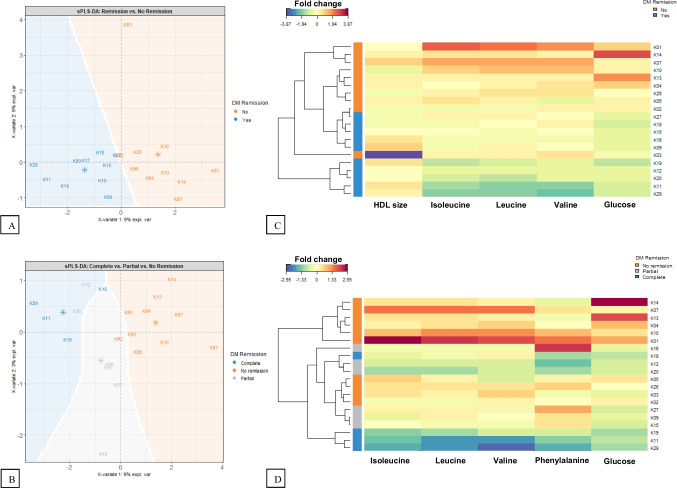
Sample projection in the space spanned by the two first Latent Structures obtained by sPLS-DA (**A** and **B**). Sample clustering according to their likelihood (complete linkage) with heatmaps of fold change of the most relevant variables (**C** and **D**). Variables included in the model (comp 1: 4; comp 2: 1) were selected by performance analysis to minimize the overall classification error. The background represents the “area of influence” where a sample is more likely to be classified either as (**A**) No remission vs. partial and complete remission, (**B**) for no remission vs. partial vs. complete remission. Areas were calculated based on the Mahalonobis distance to the group’s centroid. The heatmap represents the fold change for each variable, comparing to the most central sample (the sample closest to the origin of the space spanned by the two first Latent Structures obtained by sPLS-DA) (**C**) for no remission vs. partial and complete remission, (**D**) for no remission vs. partial vs. complete remission

There was a segregation of metabolites between complete and partial remitters vs. non-remitters, and between complete vs. partial vs. non-remitters when analysed by the sample projection in the spaced spanned by the two first Latent Structures obtained by sPLS-DA (Fig. 2A and B). The range of metabolites of partial remitters overlapped complete remitters and non-remitters (Fig. 2B).

Table 2 shows the most discriminant variables for complete and partial remitters vs. non-remitters, and for complete vs. partial vs. non-remitters in component 1 and 2 by the sPLS-DA method (P = 0.001 for both by PERMANOVA statistics). VLDL size and branched-chain amino acids (BCAAs) including isoleucine, leucine and valine were significantly most discriminant variables for partial and complete remitters vs. non-remitters and for complete vs. partial vs. non-remitters. HDL size was discriminant only for partial and complete remission vs. no-remission, whereas phenylalanine was discriminant only for complete vs. partial vs. non-remission (Table 2, Fig. 2C and D).

**Table 2:** Selected variables in the sPLS-DA method using the performance analyses to minimise overall classification error rate. Differences between experimental groups were then tested using PERMANOVA statistics based on spatial coordinates spanned by the two first components of sPLS-DA method. *adjusted *P*-value = 0.001

Variables	Component 1	Variables	Component 2
Complete and partial remission vs. no remission*			
VLDL size	0.187821	HDL size	-1
Isoleucine	0.428503		
Leucine	0.410028		
Valine	0.426999		
Complete vs. partial vs. no remission*			
VLDL size	0.142857	Phenylalanine	-1
Isoleucine	0.469549		
Leucine	0.437919		
Valine	0.423811		

**Fig. 3:**
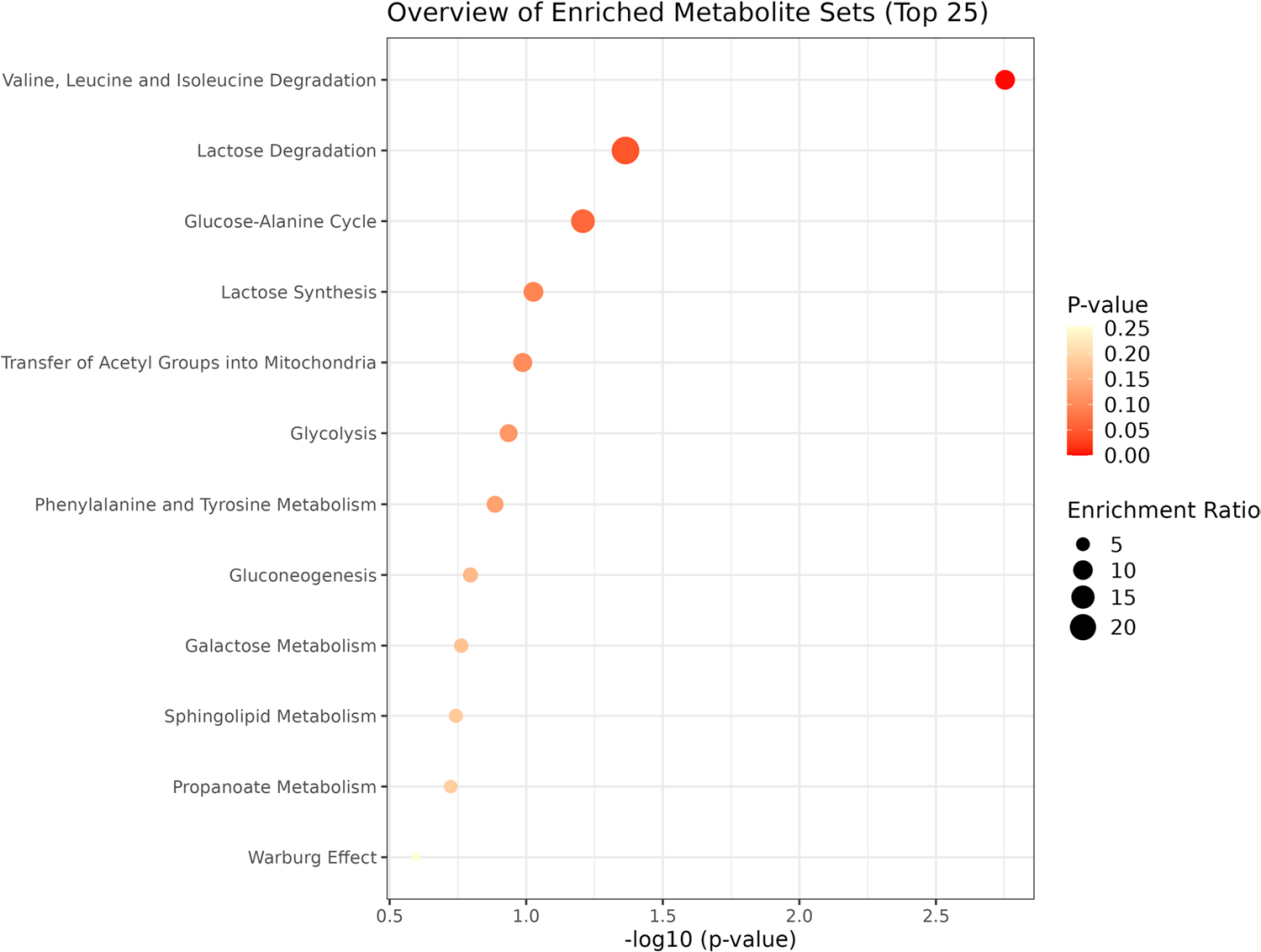
Enrichment analysis of metabolic pathways related to diabetes remission status, obtained by MetaboAnalyst 5.0. The most discriminant variables in the optimised sPLS-DA were compared against the SMPDB of human metabolites to estimate the metabolic pathways more related to diabetes remission. Significance was considered when *p* < 0.1

Enrichment analysis showed that degradation of BCAAs was the top metabolic pathway related to T2D remission status, followed by lactose degradation and glucose-alanine cycle (Fig. 3).

Given that BCAAs are most discriminant variables by the sPLS-DA method and their degradation are the top metabolic pathway from enrichment analysis, an ROC curve analysis was performed to determine the best cut-off value of AUC_0-180_ total BCAAs for being non-remitters. The optimal cut-off was AUC_0-180_ total BCAAs at 94.7 mmol x min/L with a sensitivity of 80% and specificity of 100% for being non-remitters (Supplementary Figure S2).

### Association of AUC_0-180_ Total BCAAs with Visceral Fat Parameters and Insulin Sensitivity Indices

**Fig. 4:**
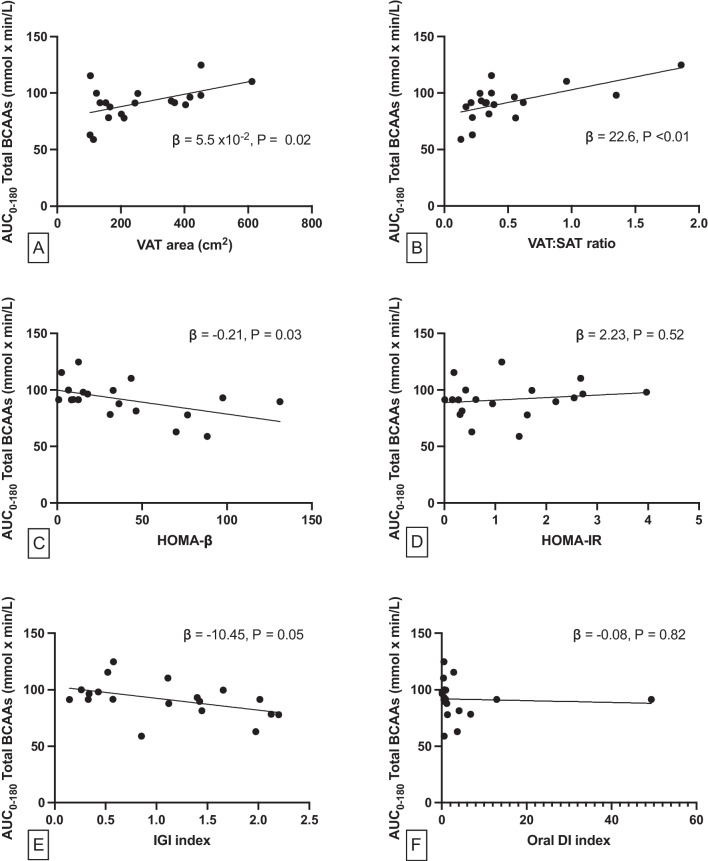
Association of AUC_0-180_ total BCAAs with visceral fat parameters and insulin sensitivity indices; **A**, visceral adipose tissue (VAT) area (cm.^2^); **B**, VAT:subcutaneous adipose tissue (VAT:SAT) ratio; **C**, HOMA-β; **D**, HOMA-IR; **E**, insulinogenic index (IGI); **F**, oral disposition index (DI)

Linear regression analysis revealed that AUC_0-180_ total BCAAs positively correlated with VAT area (P = 0.02) and VAT:SAT ratio (P < 0.01) (Fig. 4A and B). In contrast, the AUC_0-180_ total BCAAs negatively correlated with HOMA-β (P = 0.03, Fig. 4C).

### Gut Hormone Profiles

The levels of AUC_0-150_ AG, fasting AG:DAG and AUC_0-150_ AG:DAG in remitters were significantly greater than non-remitters (P = 0.03, 0.02 and 0.03, respectively, Table 1). The levels of fasting DAG, AUC_0-150_ DAG and **Δ**AUC_0-150_ DAG were comparable between groups (Table 1). There was no significant difference between groups in PYY, GLP-1 and FGF-19 parameters (Table 1).

### Association between Genetic Risk Scores and Type 2 Diabetes Remission

There is no significant association between any GRSs and T2D remission after bariatric/metabolic surgery in this study (Supplementary Table S2).

## Discussion

In our study, the key findings were that T2D remitters had greater β-cell function, fasting AG:DAG, levels of plasma AG during a MMTT, and lower VAT:SAT ratio than their matched non-remitters. Circulating levels of BCAAs and the size of VLDL particle were significantly discriminant for T2D remission status, analysed by the sPSL-DA method. The levels of AUC_0-180_ total BCAAs significantly positively correlated with VAT:SAT ratio and VAT area, whereas they significantly negatively associated with HOMA-β.

One of the most noteworthy findings from our study is that non-remitters had a significantly greater VAT:SAT ratio (1.8x) and a VAT area (1.9x) (although statistically insignificant) than remitters. This leads us to propose that VAT is a key determinant of T2D remission post-bariatric/metabolic surgery and that lower VAT and VAT:SAT area plays a crucial role in the metabolic advantage in remitters resulting in improved glycaemic control and T2D remission. Importantly, this trend is observed up to 14 years following bariatric/metabolic surgery whereby a 1SD increase in VAT volume was significantly associated with reduced T2D remission (0.6x) and a 2.3 × increase in diabetes incidence [18].

This raises the question of whether T2D remission is due to a greater post-operative loss of VAT or is pre-determined by individuals’ pre-operative VAT mass and further longitudinal studies are required to examine. A 2011 paper by Kim et al. concluded that patients with lower pre-operative VAT and VAT:SAT was a positive predictor of T2D remission at 1 year post bariatric/metabolic surgery [19]. The addition of pre-operative VAT mass may enhance predictive T2D remission scores.

Existing evidence has shown that higher VAT is associated with lower ghrelin levels and insulin resistance. A recent paper [20] showed that elevated fasting ghrelin levels were associated with enhanced insulin sensitivity and VAT regression independent of weight-loss. This is in accordance with our study whereby T2D remitters who had lower VAT:SAT ratio had a twofold greater increase of circulating AG and AG:DAG compared to non-remitters. These findings are also in agreement with a previous study by Yang et al*.* who found that T2D remission following Roux-en-Y gastric bypass (RYGB) was associated with increased plasma AG [21].

The role of AG and DAG on insulin and glucose metabolism remains to be fully understood. It has been demonstrated in *in-vitro* human studies that both AG and DAG promote proliferation, cell survival and inhibit apoptosis of pancreatic β-cells and have complementary roles on insulin and glucose metabolism [21, 22]. This may translate towards our other significant findings of increased AUC_0-15_ and AUC_0-30_ insulin, HOMA-β, IGI and oral DI in T2D remitters indicating a restoration of β-cell function, which could be the key determinant for T2D remission after bariatric/metabolic surgery.

In our present study, analysis of sPLS-DA and sample clustering revealed an evident discrimination of identified metabolites between non-remitters and remitters. Our next significant finding is that the top metabolites determining clustering were the AUC_-0–180_ of leucine, isoleucine and valine, also known as ‘BCAAs’.

Increasing evidence indicates that BCAAs act as metabolic markers of insulin resistance and are positively associated with T2D and may contribute to the pathogenesis of T2D in a weight-independent manner [23]. A rapid decrease in BCAA levels after bariatric/metabolic surgery, is typically observed concomitant with enhanced gluconeogenesis and insulin sensitivity [24].

It can be theorised that the initial BCAA dysmetabolism may be caused by excess VAT as, in our study, linear regression analysis reveals that AUC_0-180_ total BCAAs positively correlated with VAT area, VAT:SAT ratio and negatively correlated with HOMA-β. In support, *Lackey *et al. [25] suggests that the BCAA catabolic pathway is responsive to insulin changes, indicating that in an insulin resistant state, this downregulates expression of BCAA catabolic enzymes, with visceral white adipose tissue in particular playing a prominent role in modulating systemic BCAA levels.

These findings potentially indicate that inefficient BCAA catabolism may be responsible for the elevated levels of BCAAs that were observed in non-remitters in our study and in others [26, 27]. However, a consensus has yet to be reached on whether elevated levels of BCAAs are causal or a result of the metabolic dysregulations of T2D. An opportunity arises for new therapeutic approaches for T2D treatment following bariatric/metabolic surgery by targeting restoration of BCAA catabolism [28].

In existing literature, preoperative severity of T2D, as evidenced by pre-operative medication usage, is also highly likely to play a role as compared to the remitters in our study, non-remitters were more likely to be on pre-operative T2D pharmacotherapies. This could indicate poorer pre-operative β-cell function in non-remitters compared to remitters which may play a key role in the capacity and capability of β-cell restoration and subsequent T2D remission after bariatric/metabolic surgery.

The state of chronic energy excess leading to raised hepatic and pancreatic fat contents has been postulated to be a part of the pathogenesis of T2D [29]. In contrast, a previous genetic association study of the UK Biobank showed that pancreatic fat had no impact on developing T2D [30]. We provide further evidence that T2D remission is unlikely to be due to differences in pancreatic and/or hepatic fat content as these factors were comparable between groups. Taylor and colleagues also found that following a primary care-led weight management program, hepatic and pancreatic fat was comparable between T2D remitters and non-remitters [31].

The concept of elevated incretin hormones contributing to the surgery-induced improvement in glucose homeostasis and diabetes remission has been widely accepted [5, 32, 33]. However, other studies have found that fasting GLP-1 and PYY concentrations are significantly higher in patients with insulin resistance and T2D, compared to those with NGT [34, 35]. In our study, there was no significant difference between groups in PYY and GLP-1 parameters. The GLP-1 responses during the MMTT did not differ, indicating that differences in GLP-1 responses did not explain the poor β-cell function in non-remitters.

Our metabolomics data also revealed that the size of VLDL and HDL particles was another most discriminant variable for remitters vs. non-remitters, consistent with other findings linking lipoprotein particle size, insulin resistance and T2D [36]. This is in accordance with the well-established association between T2D and a distinct dyslipidaemic profile coined ‘diabetic dyslipidemia’[37].

There are several limitations of this study. First, pre-operative anti-hyperglycaemic agent use in non-remitters were greater than remitters at the time of surgery, indicating greater pre-operative T2D severity in this group which is an established risk factor for non-remission post bariatric surgery. Second, owing to the cross-sectional study design, the present study cannot prove the causal relationship of different factors between groups with the remission of T2D. Therefore, further longitudinal studies are now warranted.

This study identified novel weight-loss independent clinical and metabolic differences between T2D remitters and non-remitters at long-term post-bariatric/metabolic surgery. We highlight the link between VAT and BCAA metabolism which may play a role in glucoregulatory control. Improvement in β-cell function, and the role ghrelin plays in its recovery, is likely another key factor influencing T2D remission status post bariatric/metabolic surgery. Investigation of pharmacological agents that target BCAA catabolism, acyl ghrelin and VAT loss to restore β-cell function may pave the way for increased rates of long-term T2D remission after bariatric/metabolic surgery.

### Supplementary Information

Below is the link to the electronic supplementary material.

Supplementary file1 (DOCX 9330 KB)
