# Supplementary material for: Weight-loss Independent Clinical and Metabolic Biomarkers Associated with Type 2 Diabetes Remission Post-bariatric/metabolic Surgery
Source: Obes Surg. 2023 Nov 1;33(12):3988–98. doi: 10.1007/s11695-023-06905-8 (PMC10687127; doi:10.1007/s11695-023-06905-8)
Supplement: Supplementary file 1 — Supplementary file1 (DOCX 9330 KB) [file 11695_2023_6905_MOESM1_ESM.docx]

**Supplementary material for MRI**

All scans were acquired on the same 3T Philips scanner (Ingenia, Philips, Amsterdam, Netherlands) at UCLH. The acquisition consisted of chemical shift-encoded (CSE)-MRI sequences with proton density fat fraction (PDFF) maps generated in-line using Philips mDixon Quant. Acquisition parameters were TE/ΔTE 0.95/0.82ms, number of echoes 6, TR 25 ms, flip angle 3°, matrix size 320×320×40, resolution 2.5×2.5×5 mm^3^.

Liver and pancreatic PDFF were measured using an in-house MATLAB (MathWorks, Natick, MA, USA) tool. For the liver, a circular region of interest (ROI) measuring up to 20 mm in diameter was placed in each of the Couinaud segments on the PDFF maps. For the pancreas, circular ROIs with an area of 100 mm^2^ were placed on PDFF maps of the parenchyma of the head, body and tail of the pancreas. Two ROIs were placed in each region of the pancreas on different slices (*i.e.* 6 ROIs in total). The pancreatic fat content for each region of the pancreas was calculated as the mean PDFF of the two ROIs and a total mean pancreatic PDFF was calculated as the mean of the three regions. Images were analysed independently by 2 observers; the mean of the two observers’ ROI readings was used in the analysis. Interobserver agreement was assessed by comparing the results of the PDFF reads performed by the 2 observers.


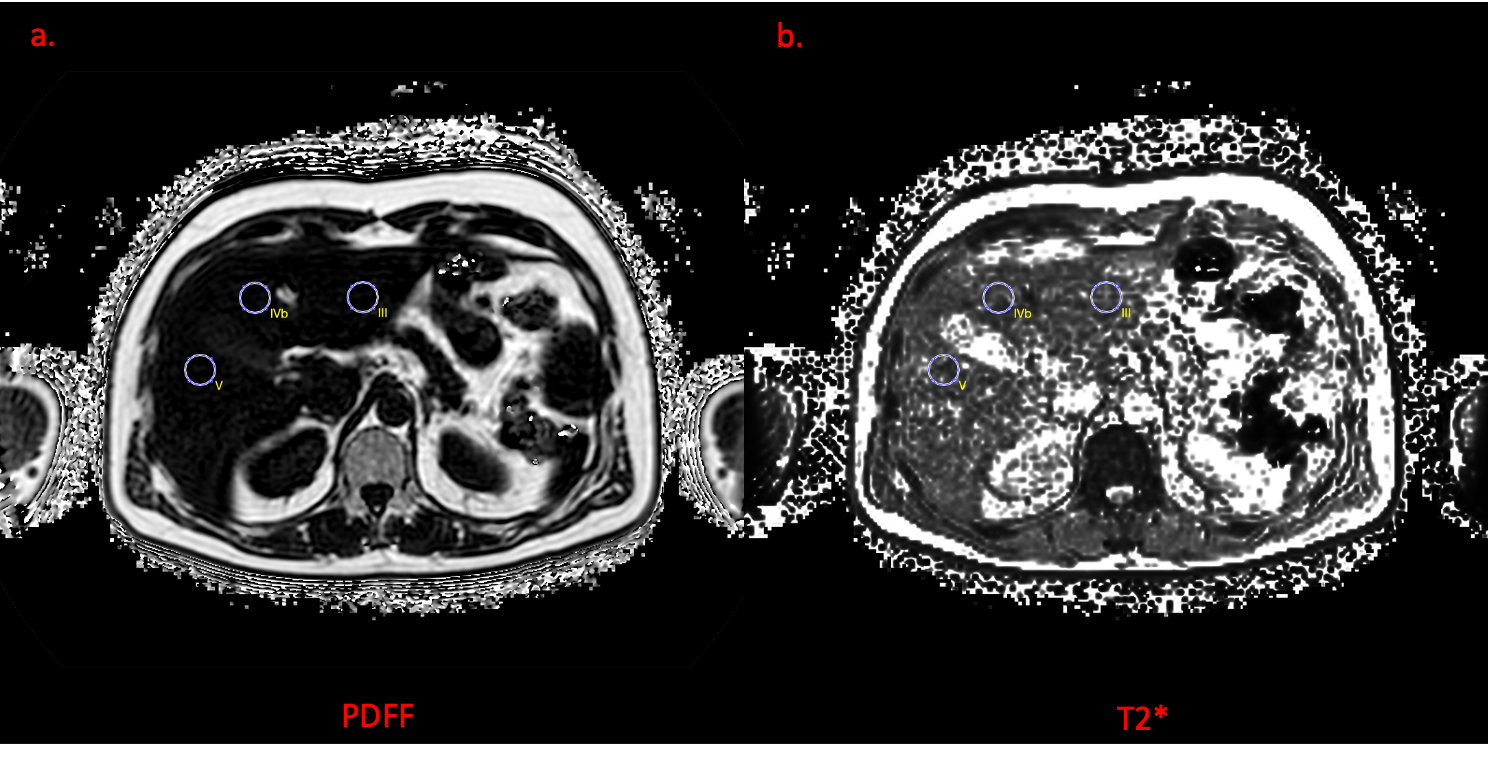

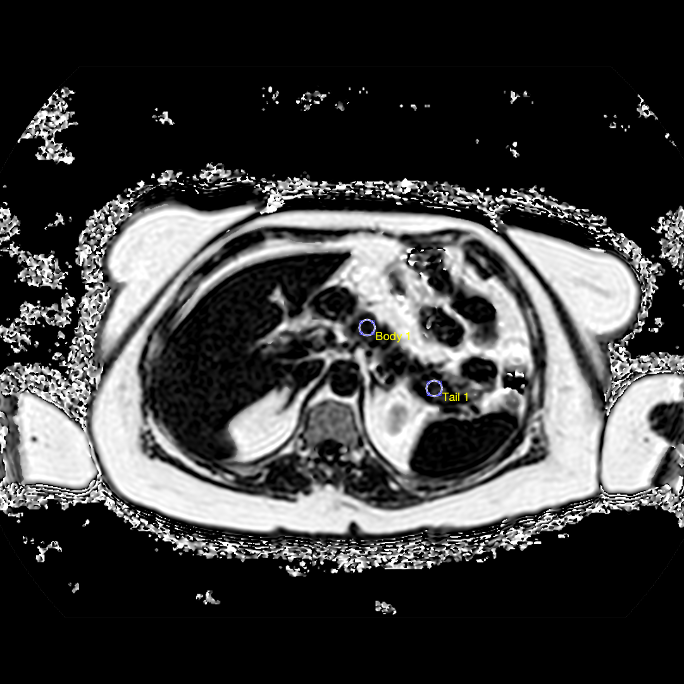


a.

b.

Figure 1 – Example of colocalised ROI placement on (a) a PDFF map of the liver and (b) a PDFF map of the pancreas.

An in-house MATLAB tool (BONSAI, described in detail in previous work (1))was used to segment adipose tissue and muscle to give measurements of body composition. The BONSAI tool uses paired axial fat-only and water-only images, acquired as part of the CSE-MRI sequences, at the level of the L3 vertebral body as input data. ROIs were generated in the BONSAI tool by two observers.

The visceral:subcutaneous fat ratio (VAT:SAT) was calculated from the visceral and subcutaneous fat areas (in cm^2^) at the level of the L3 vertebral body.

Total body fat mass (FM) and fat-free mass were estimated according to previously published regression equations (2):

$$\begin{aligned} Total body FM\left( kg \right)=0.042\times\left[ total adipose tissue at L3\left( {cm}^{2} \right) \right]+11.2\# \end{aligned}$$

$$\begin{aligned} Total body FFM \left( kg \right)=0.3\times\left[ skeletal muscle at L3\left( {cm}^{2} \right) \right]+6.06\# \end{aligned}$$

FM and FFM were normalized for stature to derive the FM index (kg/m^2^) and FFM index (kg/m^2^) respectively:

$$\begin{aligned} FM index {(kg}/{m^{2})=\frac{Total body FM}{height\left( m^{2} \right)}}\# \end{aligned}$$

$$\begin{aligned} FFM index{(kg}/{m^{2})=\frac{Total body FFM}{height\left( m^{2} \right)}}\# \end{aligned}$$

Skeletal muscle (SM) area was also normalised for stature to give the skeletal muscle index (cm^2^/m^2^):

$$\begin{aligned} SM index{({cm}^{2}}/{m^{2})=\frac{SM area {cm}^{2}}{height\left( m^{2} \right)}}\# \end{aligned}$$

SM PDFF was measured from the PDFF maps.

**References**

1. Sakai NS, Bhagwanani A, Bray TJP, Hall-Craggs MA, Taylor SA, On behalf of Streamline Investigators. Assessment of body composition and association with clinical outcomes in patients with lung and colorectal cancer. BJR Open 2021; 3: 20210048.
2. Mourtzakis M, Prado CMM, Lieffers JR, Reiman T, McCargar LJ, Baracos VE. A practical and precise approach to quantification of body composition in cancer patients using computed tomography images acquired during routine care. Appl Physiol Nutr Metab 2008; 33: 997–1006.

**Supplementary material for metabolomics analysis**

A Discriminant Analysis based on sparse Projection of Latent Structures (sPLS-DA) was performed using the AUC of the metabolites over the duration of the MMTT as predictors of diabetes remission (1). A performance analysis was conducted to minimize overall error classification rate. Briefly, PLS-DA models with different number of independent variables in each of the first two latent structures (LS) were generated using an automated script. The number of variables in each latent structure achieving the lowest error classification rate were then selected to run the sPLS-DA model. PERMANOVA was used to numerically compare sample distribution in the space spanned by the two first LSs according to the diabetes remission status (2). A matrix of Euclidean distances between samples was calculated and 999 permutations were performed. P-values were corrected for multiple hypothesis testing by controlling the False Discovery Rate (FDR) with the Benjamini-Hochberg method (3). Significance was considered whenever the adjusted p-value <0.05. These methods were performed with R 4.2.0 (4), using the R packages mixOmics 6.14.0 (5) and vegan 2.5-7 (6). Enrichment analysis was performed using MetaboAnalyst 5.0 (7), taking the most relevant variables in the sPLS-DA model as input. P-values of univariate analysis were adjusted using the Benjamini-Hochberg test. Receiver operating characteristic (ROC) curves were then obtained for variables with significant differences, using logistic regression. These methods were performed using IBM SPSS Statistics v27.0.1.

**References**

1. Le Cao KA, Rossouw D, Robert-Granie C, Besse P. A sparse PLS for variable selection when integrating omics data. Stat Appl Genet Mol Biol. 2008;7(1):Article 35.

2. Anderson MJ. Permutational Multivariate Analysis of Variance (PERMANOVA). Wiley StatsRef: Statistics Reference Online2017. p. 1-15.

3. Benjamini Y, Hochberg Y. Controlling the False Discovery Rate: A Practical and Powerful Approach to Multiple Testing. Journal of the Royal Statistical Society: Series B (Methodological). 1995;57(1):289-300.

4. R Core Team. R: A Language and Environment for Statistical Computing Vienna, Austria: R Foundation for Statistical Computing; 2021 [Available from: https://www.r-project.org/.

5. Rohart F, Gautier B, Singh A, Le Cao KA. mixOmics: An R package for 'omics feature selection and multiple data integration. PLoS Comput Biol. 2017;13(11):e1005752.

6. Oksanen J, Blanchet FG, Friendly M, Kindt R, Legendre P, McGlinn D, et al. vegan: Community Ecology Package 2020 [Available from: https://cran.r-project.org/package=vegan.

7. Chong J, Wishart DS, Xia J. Using MetaboAnalyst 4.0 for Comprehensive and Integrative Metabolomics Data Analysis. Curr Protoc Bioinformatics. 2019;68(1):e86.

**Supplementary Table S1** SNPs lists for genetic risk score analysis

| **BMI SNPs** | **CHR** | **POS** | **Effect Allele** | **Other Allele** | **Effect Allele Frequency** | **BETA** | **SE** | **P** | **N** | **INFO** |
| --- | --- | --- | --- | --- | --- | --- | --- | --- | --- | --- |
| rs544722 | 1 | 10743042 | T | C | 0.4545 | 0.0113 | 0.0019 | 2.76E-09 | 484680 | 0.995679 |
| rs1730859 | 1 | 107617707 | A | G | 0.651 | -0.012 | 0.0017 | 2.96E-12 | 806755 | 0.988579 |
| rs12035149 | 1 | 107885018 | C | G | 0.782 | -0.0134 | 0.0021 | 7.42E-11 | 718693 | 0.996338 |
| rs11185111 | 1 | 107962328 | A | G | 0.2906 | -0.0127 | 0.0019 | 1.08E-11 | 718693 | 0.979492 |
| rs3820667 | 1 | 109968410 | A | C | 0.471 | 0.0095 | 0.0016 | 3.43E-09 | 806242 | 0.997019 |
| rs17024393 | 1 | 110154688 | T | C | 0.9689 | -0.0644 | 0.0049 | 7.11E-39 | 782554 | 0.987842 |
| rs116715046 | 1 | 111355258 | T | C | 0.047 | -0.0246 | 0.0045 | 4.79E-08 | 484680 | 0.9912 |
| rs1546924 | 1 | 112273485 | T | C | 0.4997 | 0.0139 | 0.0016 | 2.34E-17 | 794849 | 0.993581 |
| rs3128365 | 1 | 112841203 | A | G | 0.3157 | 0.0101 | 0.0018 | 3.95E-08 | 718377 | 0.996104 |
| rs10779751 | 1 | 11284336 | A | G | 0.2787 | 0.0131 | 0.0018 | 2.66E-13 | 806531 | 0.999604 |
| rs6663115 | 1 | 115272254 | A | G | 0.662 | -0.0105 | 0.0018 | 2.48E-09 | 718522 | 0.999495 |
| rs7534091 | 1 | 118864616 | A | G | 0.7182 | -0.012 | 0.0018 | 6.65E-11 | 803017 | 0.99869 |
| rs1318408 | 1 | 11925781 | A | G | 0.8845 | -0.0138 | 0.0025 | 4.62E-08 | 806511 | 0.988308 |
| rs10923724 | 1 | 119546842 | T | C | 0.5562 | -0.0126 | 0.0016 | 1.14E-14 | 806787 | 0.992599 |
| rs74887628 | 1 | 147032779 | A | G | 0.0339 | 0.0305 | 0.0054 | 1.76E-08 | 484680 | 0.938048 |
| rs12733421 | 1 | 150616117 | T | C | 0.0584 | 0.0174 | 0.0031 | 1.57E-08 | 793528 | 0.991499 |
| 1_151018861_A_G | 1 | 151018861 | A | G | 0.241 | 0.0157 | 0.002 | 2.17E-15 | 718276 | 0.995893 |
| rs905938 | 1 | 154991389 | T | C | 0.7223 | -0.0143 | 0.0019 | 1.71E-14 | 806412 | 1 |
| rs4276913 | 1 | 155131673 | A | G | 0.5896 | 0.0096 | 0.0017 | 2.45E-08 | 718302 | 0.999496 |
| rs11577179 | 1 | 155983710 | A | G | 0.3806 | -0.012 | 0.0017 | 6.85E-13 | 805734 | 0.993931 |
| rs61813324 | 1 | 156049877 | T | C | 0.1312 | 0.0289 | 0.0028 | 3.20E-24 | 484680 | 0.966354 |
| rs75160483 | 1 | 156238735 | T | C | 0.0252 | 0.0447 | 0.0062 | 4.12E-13 | 484680 | 0.96432 |
| rs11264483 | 1 | 156406381 | C | G | 0.5899 | 0.0138 | 0.0018 | 3.51E-15 | 696134 | 0.980215 |
| rs7544205 | 1 | 156493074 | A | G | 0.6483 | -0.0111 | 0.002 | 2.19E-08 | 484680 | 0.994724 |
| 1_15808872_A_G | 1 | 15808872 | A | G | 0.2272 | -0.0107 | 0.0018 | 4.63E-09 | 806782 | 1 |
| rs79113395 | 1 | 1590521 | A | G | 0.266 | -0.02 | 0.0022 | 2.03E-20 | 484680 | 0.988786 |
| rs10733051 | 1 | 167280354 | A | G | 0.512 | 0.0093 | 0.0016 | 6.96E-09 | 794799 | 0.997308 |
| rs78886584 | 1 | 16859325 | A | G | 0.5018 | -0.0106 | 0.0019 | 3.44E-08 | 484680 | 0.970864 |
| rs34720381 | 1 | 171455322 | T | C | 0.0889 | 0.0229 | 0.0033 | 7.89E-12 | 484680 | 0.993262 |
| rs761423 | 1 | 17301672 | T | C | 0.5412 | 0.0105 | 0.0017 | 6.51E-10 | 718637 | 1 |
| rs61828641 | 1 | 174321997 | A | G | 0.1092 | 0.0223 | 0.003 | 2.49E-13 | 484680 | 0.996355 |
| rs7547843 | 1 | 174507529 | A | G | 0.07 | -0.0186 | 0.0033 | 1.47E-08 | 714564 | 0.999041 |
| rs6700816 | 1 | 174883994 | A | T | 0.9691 | 0.0346 | 0.005 | 4.83E-12 | 713101 | 0.997836 |
| rs10913118 | 1 | 175954755 | A | C | 0.6845 | -0.0097 | 0.0017 | 1.78E-08 | 806767 | 0.999613 |
| rs10489219 | 1 | 177326069 | A | G | 0.1004 | 0.0148 | 0.0027 | 3.01E-08 | 806707 | 0.986321 |
| rs148731480 | 1 | 177684113 | C | G | 0.03 | 0.0336 | 0.0059 | 9.92E-09 | 484680 | 0.897863 |
| rs12402666 | 1 | 177753473 | T | C | 0.2194 | -0.0118 | 0.0019 | 5.54E-10 | 705868 | 0.997092 |
| rs10489881 | 1 | 177839612 | T | G | 0.7433 | 0.012 | 0.0018 | 4.57E-11 | 805777 | 0.962794 |
| 1_177889480_A_G | 1 | 177889480 | A | G | 0.7786 | -0.0479 | 0.002 | 3.06E-125 | 806688 | 1 |
| rs55924135 | 1 | 177924354 | A | G | 0.2475 | -0.0173 | 0.0022 | 3.80E-15 | 484680 | 0.993197 |
| rs1336779 | 1 | 177968197 | A | C | 0.2111 | 0.0166 | 0.0021 | 1.48E-15 | 717924 | 0.992833 |
| rs10797987 | 1 | 184651822 | T | C | 0.5116 | -0.0117 | 0.0019 | 7.69E-10 | 484680 | 0.99535 |
| rs1884447 | 1 | 185021410 | A | G | 0.4087 | -0.0106 | 0.0019 | 3.68E-08 | 484680 | 0.997304 |
| 1_1865298_A_G | 1 | 1865298 | A | G | 0.4551 | -0.0142 | 0.0017 | 1.88E-16 | 716462 | 0.991647 |
| rs10920678 | 1 | 190239907 | A | G | 0.425 | 0.0149 | 0.0016 | 7.15E-20 | 805921 | 0.996095 |
| 1_190804630_G_T | 1 | 190804630 | T | G | 0.4436 | -0.0095 | 0.0017 | 2.44E-08 | 718441 | 0.995177 |
| rs12086884 | 1 | 192996357 | A | T | 0.2428 | 0.0108 | 0.002 | 4.18E-08 | 717691 | 0.99836 |
| rs2400414 | 1 | 194965200 | T | C | 0.3461 | -0.0126 | 0.0018 | 5.52E-13 | 718698 | 0.996849 |
| rs1999981 | 1 | 195088871 | T | G | 0.5724 | 0.0103 | 0.0017 | 1.88E-09 | 718600 | 0.996236 |
| 1_197257090_C_T | 1 | 197257090 | T | C | 0.7137 | 0.0124 | 0.0019 | 7.15E-11 | 718619 | 0.995335 |
| 1_197559324_C_T | 1 | 197559324 | T | C | 0.2347 | 0.0114 | 0.002 | 9.42E-09 | 718674 | 0.999106 |
| rs61740466 | 1 | 19934900 | A | G | 0.2346 | -0.0142 | 0.0022 | 2.24E-10 | 484680 | 0.997594 |
| rs2820295 | 1 | 201800868 | A | G | 0.3266 | 0.0235 | 0.0018 | 5.56E-39 | 718545 | 0.994889 |
| rs9077 | 1 | 202116238 | A | G | 0.2713 | -0.0136 | 0.0019 | 2.97E-13 | 699288 | 0.985561 |
| rs4971212 | 1 | 203491392 | T | G | 0.7893 | -0.0149 | 0.0023 | 1.19E-10 | 484680 | 1 |
| rs823075 | 1 | 205774897 | T | C | 0.4198 | -0.0096 | 0.0017 | 2.40E-08 | 718559 | 0.996194 |
| rs11576725 | 1 | 208706936 | A | G | 0.2998 | -0.0106 | 0.0019 | 2.81E-08 | 716831 | 0.980213 |
| rs11119208 | 1 | 209211968 | A | G | 0.3879 | 0.0108 | 0.0017 | 6.94E-11 | 806749 | 0.997302 |
| rs17014375 | 1 | 209543560 | T | G | 0.8691 | -0.0165 | 0.0025 | 4.03E-11 | 718328 | 0.994594 |
| rs6661316 | 1 | 210095527 | T | C | 0.5893 | 0.012 | 0.0016 | 1.72E-13 | 806610 | 0.999019 |
| rs11118308 | 1 | 219633869 | A | G | 0.5537 | 0.0098 | 0.0016 | 1.55E-09 | 806492 | 0.99903 |
| 1_225668524_A_G | 1 | 225668524 | A | G | 0.2786 | -0.0108 | 0.0019 | 1.87E-08 | 718369 | 0.987584 |
| rs4653942 | 1 | 228505204 | A | G | 0.2074 | -0.0113 | 0.002 | 2.25E-08 | 806528 | 1 |
| rs10864728 | 1 | 230304914 | A | G | 0.3978 | 0.011 | 0.0019 | 1.20E-08 | 484680 | 0.999863 |
| 1_23312025_C_T | 1 | 23312025 | T | C | 0.8362 | -0.0173 | 0.0022 | 6.20E-15 | 718666 | 0.995703 |
| rs1874129 | 1 | 235700023 | T | G | 0.8686 | -0.0137 | 0.0025 | 3.41E-08 | 706621 | 0.992619 |
| rs2491864 | 1 | 242986063 | A | G | 0.2155 | 0.0129 | 0.002 | 1.60E-10 | 717225 | 0.99872 |
| rs12042959 | 1 | 243533273 | A | G | 0.8637 | 0.0153 | 0.0024 | 2.68E-10 | 718663 | 0.984695 |
| rs946824 | 1 | 243684019 | T | C | 0.1355 | 0.0197 | 0.0025 | 4.82E-15 | 717750 | 0.993448 |
| rs7535528 | 1 | 2444414 | A | G | 0.3718 | -0.0154 | 0.0018 | 1.58E-17 | 668295 | 0.987723 |
| rs945211 | 1 | 32191798 | C | G | 0.6269 | 0.0127 | 0.0018 | 5.03E-13 | 704966 | 1 |
| rs12022461 | 1 | 33232525 | A | G | 0.1674 | -0.0167 | 0.0022 | 9.78E-14 | 718684 | 0.999388 |
| rs4653017 | 1 | 33776728 | T | C | 0.6679 | 0.0118 | 0.0018 | 1.11E-10 | 716691 | 0.993429 |
| rs7512146 | 1 | 34283008 | T | G | 0.5272 | -0.0097 | 0.0017 | 1.29E-08 | 716980 | 0.994691 |
| rs12031634 | 1 | 34584393 | A | G | 0.2905 | -0.0122 | 0.0021 | 5.26E-09 | 484680 | 0.987722 |
| rs11577094 | 1 | 38026600 | T | C | 0.0791 | 0.0186 | 0.003 | 3.28E-10 | 791496 | 0.999644 |
| rs6665962 | 1 | 39534947 | A | G | 0.2857 | 0.0118 | 0.0022 | 4.25E-08 | 484680 | 0.935026 |
| rs112566467 | 1 | 39562627 | T | C | 0.2071 | 0.0184 | 0.0024 | 5.86E-15 | 484680 | 0.986296 |
| rs61743745 | 1 | 40036944 | A | G | 0.029 | -0.0343 | 0.0057 | 1.54E-09 | 484680 | 0.984983 |
| rs7523668 | 1 | 42408159 | A | G | 0.4443 | -0.0105 | 0.0017 | 7.24E-10 | 718687 | 0.995435 |
| rs1707322 | 1 | 46505147 | A | G | 0.3155 | -0.0136 | 0.0018 | 1.59E-14 | 806793 | 0.999466 |
| rs2984618 | 1 | 47690438 | T | G | 0.4408 | 0.0165 | 0.0016 | 3.80E-24 | 806433 | 0.997039 |
| 1_49589847_A_G | 1 | 49589847 | A | G | 0.4042 | 0.0188 | 0.0016 | 3.17E-30 | 798331 | 0.987417 |
| rs4926853 | 1 | 50709222 | A | G | 0.6746 | 0.0112 | 0.0017 | 6.23E-11 | 802812 | 1 |
| rs630602 | 1 | 54728864 | C | G | 0.6073 | 0.0126 | 0.0018 | 1.06E-12 | 716881 | 0.988556 |
| 1_56962821_A_G | 1 | 56962821 | A | G | 0.9033 | -0.0158 | 0.0028 | 1.37E-08 | 805725 | 1 |
| rs17425707 | 1 | 57874879 | T | C | 0.9 | -0.0173 | 0.0028 | 6.16E-10 | 714959 | 1 |
| rs991191 | 1 | 62521305 | T | C | 0.5975 | 0.0129 | 0.0017 | 1.15E-13 | 718001 | 0.981095 |
| rs12140153 | 1 | 62579891 | T | G | 0.0911 | -0.0353 | 0.0034 | 1.44E-25 | 484680 | 0.947242 |
| rs11208660 | 1 | 65983626 | T | C | 0.0735 | 0.0196 | 0.0029 | 1.27E-11 | 806568 | 0.9919 |
| rs2503185 | 1 | 66461401 | A | G | 0.5097 | 0.013 | 0.0017 | 1.33E-14 | 717297 | 0.986557 |
| rs6577584 | 1 | 6715390 | T | G | 0.6589 | -0.0118 | 0.0018 | 3.37E-11 | 718636 | 0.998663 |
| rs7541380 | 1 | 72421349 | T | C | 0.8898 | 0.0144 | 0.0026 | 4.52E-08 | 718682 | 0.996071 |
| rs2821278 | 1 | 72509683 | T | C | 0.2094 | -0.013 | 0.0021 | 2.64E-10 | 775865 | 0.939027 |
| rs12124523 | 1 | 72621463 | T | C | 0.1077 | 0.0162 | 0.0027 | 2.32E-09 | 774614 | 0.987587 |
| rs3101336 | 1 | 72751185 | T | C | 0.3825 | -0.0254 | 0.0016 | 4.80E-54 | 801552 | 1 |
| rs782226 | 1 | 72976932 | A | C | 0.1837 | -0.0113 | 0.002 | 2.38E-08 | 718465 | 0.994716 |
| rs12566985 | 1 | 75002193 | A | G | 0.5566 | -0.0194 | 0.0016 | 2.80E-33 | 803962 | 0.998187 |
| rs1891215 | 1 | 7727854 | T | C | 0.5397 | -0.0111 | 0.0017 | 7.37E-11 | 718661 | 0.989138 |
| rs2689676 | 1 | 77764869 | A | G | 0.2251 | 0.0148 | 0.0021 | 1.17E-12 | 718650 | 0.99849 |
| rs10873947 | 1 | 77986516 | A | G | 0.3798 | -0.0131 | 0.0016 | 1.47E-15 | 806762 | 1 |
| rs34517439 | 1 | 78450517 | A | C | 0.1168 | 0.0391 | 0.003 | 3.40E-39 | 484680 | 0.971217 |
| 1_78604614_A_G | 1 | 78604614 | A | G | 0.2196 | -0.0141 | 0.0019 | 1.77E-13 | 806605 | 0.997804 |
| rs12742293 | 1 | 78855358 | T | G | 0.2986 | 0.0118 | 0.0019 | 2.54E-10 | 718284 | 0.992105 |
| rs6696828 | 1 | 80812020 | C | G | 0.2976 | 0.012 | 0.0018 | 6.42E-11 | 718664 | 0.997849 |
| rs284227 | 1 | 82379446 | T | C | 0.7275 | -0.0147 | 0.0019 | 6.08E-15 | 806764 | 0.99458 |
| rs11121210 | 1 | 8708529 | T | C | 0.3445 | -0.0111 | 0.0017 | 2.37E-10 | 718632 | 0.998038 |
| rs2166171 | 1 | 91208451 | T | C | 0.6241 | -0.0103 | 0.0018 | 5.49E-09 | 710568 | 0.997302 |
| rs4970712 | 1 | 92993547 | A | C | 0.1939 | 0.014 | 0.002 | 2.75E-12 | 806729 | 0.997213 |
| rs11165468 | 1 | 96160850 | T | C | 0.5985 | -0.0093 | 0.0016 | 1.12E-08 | 794997 | 0.998583 |
| rs10747472 | 1 | 96276198 | C | G | 0.683 | 0.0153 | 0.0018 | 2.40E-17 | 718600 | 0.99334 |
| rs114495530 | 1 | 96282449 | A | G | 0.0263 | 0.0357 | 0.0059 | 1.62E-09 | 484680 | 1 |
| rs12130426 | 1 | 96603035 | T | C | 0.5755 | 0.0123 | 0.0017 | 1.25E-13 | 805801 | 0.989831 |
| rs162016 | 1 | 96831180 | T | C | 0.0301 | -0.0273 | 0.0041 | 2.93E-11 | 789418 | 0.99877 |
| rs11165643 | 1 | 96924097 | T | C | 0.5796 | 0.0185 | 0.0016 | 4.49E-30 | 805410 | 0.997531 |
| rs995258 | 1 | 97431052 | A | C | 0.5607 | 0.0141 | 0.0017 | 1.30E-16 | 718511 | 1 |
| rs12072739 | 1 | 98315893 | A | G | 0.7762 | -0.0169 | 0.0023 | 1.50E-13 | 484680 | 1 |
| rs6707827 | 2 | 100123030 | A | G | 0.3115 | -0.0105 | 0.0019 | 2.12E-08 | 718564 | 0.98214 |
| rs115325974 | 2 | 100819260 | A | G | 0.0737 | 0.0243 | 0.0037 | 3.15E-11 | 484680 | 0.977457 |
| rs4303732 | 2 | 100830040 | T | C | 0.6137 | 0.0169 | 0.0017 | 1.26E-22 | 718627 | 0.998349 |
| rs264941 | 2 | 104297420 | A | C | 0.4679 | -0.0124 | 0.0017 | 1.84E-13 | 718689 | 0.998831 |
| rs77045602 | 2 | 104670961 | A | G | 0.1133 | -0.0166 | 0.003 | 3.62E-08 | 484680 | 0.981501 |
| rs10197031 | 2 | 105454590 | T | C | 0.7262 | -0.0161 | 0.0019 | 5.05E-18 | 718456 | 0.997835 |
| rs10167912 | 2 | 105582749 | T | G | 0.2698 | -0.0135 | 0.0019 | 1.69E-12 | 718629 | 0.999071 |
| rs114792668 | 2 | 105928904 | A | T | 0.979 | 0.037 | 0.0067 | 3.75E-08 | 484680 | 0.966615 |
| rs4851057 | 2 | 105939237 | T | C | 0.1265 | 0.0163 | 0.0026 | 2.94E-10 | 710514 | 0.999843 |
| rs4676084 | 2 | 110010962 | A | G | 0.4031 | 0.0107 | 0.0017 | 4.13E-10 | 717795 | 0.993757 |
| rs3816331 | 2 | 113949736 | A | G | 0.4846 | -0.0107 | 0.0018 | 2.71E-09 | 705191 | 0.940667 |
| rs11902450 | 2 | 12845368 | T | C | 0.1052 | 0.0167 | 0.0027 | 1.06E-09 | 718624 | 0.991612 |
| rs72844740 | 2 | 133418025 | A | C | 0.9752 | -0.0346 | 0.0062 | 2.59E-08 | 484680 | 0.963677 |
| rs13033310 | 2 | 133523605 | A | G | 0.2519 | 0.0146 | 0.0022 | 3.40E-11 | 484680 | 0.984294 |
| rs4988235 | 2 | 136608646 | A | G | 0.7206 | 0.0124 | 0.0017 | 7.09E-13 | 796039 | 0.972914 |
| rs13392503 | 2 | 137431134 | T | G | 0.2502 | -0.0096 | 0.0018 | 4.65E-08 | 799215 | 0.996206 |
| rs17551974 | 2 | 142293146 | A | C | 0.1873 | -0.0127 | 0.0022 | 4.26E-09 | 718575 | 0.994782 |
| rs72851476 | 2 | 142874787 | A | C | 0.8463 | 0.0174 | 0.0027 | 1.12E-10 | 484680 | 0.945567 |
| rs2890652 | 2 | 142959931 | T | C | 0.8465 | -0.0158 | 0.0023 | 6.71E-12 | 703039 | 1 |
| rs4662565 | 2 | 143519238 | A | C | 0.298 | -0.0101 | 0.0019 | 4.73E-08 | 718573 | 0.988498 |
| rs6710871 | 2 | 143960593 | A | G | 0.1422 | 0.0193 | 0.0024 | 3.07E-16 | 752446 | 0.999519 |
| rs7560871 | 2 | 145616899 | A | G | 0.0712 | 0.0219 | 0.0034 | 6.12E-11 | 718536 | 0.994949 |
| rs114964326 | 2 | 146015229 | A | G | 0.0278 | -0.0336 | 0.0058 | 7.89E-09 | 484680 | 0.978032 |
| rs1451077 | 2 | 147901207 | A | G | 0.5776 | -0.0169 | 0.0019 | 1.43E-18 | 484680 | 0.995078 |
| rs2119753 | 2 | 151224579 | A | G | 0.6229 | 0.01 | 0.0017 | 1.00E-08 | 718468 | 0.987068 |
| rs16839578 | 2 | 156629693 | T | C | 0.3351 | -0.0101 | 0.0018 | 1.50E-08 | 718685 | 0.999216 |
| rs10198345 | 2 | 157109395 | T | C | 0.6565 | -0.0109 | 0.0018 | 1.27E-09 | 718386 | 0.996635 |
| rs62176993 | 2 | 157606799 | A | G | 0.3996 | 0.0119 | 0.0019 | 8.63E-10 | 484680 | 0.988372 |
| rs2111877 | 2 | 159388014 | T | C | 0.1419 | -0.0135 | 0.0024 | 1.83E-08 | 718294 | 0.996791 |
| rs12692596 | 2 | 161265910 | T | C | 0.3618 | 0.012 | 0.0017 | 1.03E-12 | 806739 | 0.995441 |
| rs76209259 | 2 | 161536620 | T | C | 0.7802 | 0.013 | 0.0023 | 1.23E-08 | 484680 | 0.997268 |
| rs1227932 | 2 | 162716881 | A | C | 0.5923 | 0.0097 | 0.0018 | 4.04E-08 | 664248 | 0.993494 |
| rs10192119 | 2 | 164581241 | T | G | 0.8196 | -0.0151 | 0.0021 | 1.57E-12 | 806562 | 1 |
| rs1521527 | 2 | 165427825 | C | G | 0.5182 | -0.0102 | 0.0017 | 1.77E-09 | 711599 | 0.986873 |
| rs12692738 | 2 | 165558252 | T | C | 0.7642 | -0.0131 | 0.0019 | 5.24E-12 | 806581 | 0.983823 |
| rs61051952 | 2 | 166148324 | A | G | 0.7549 | 0.0151 | 0.0022 | 6.81E-12 | 484680 | 0.999743 |
| rs51195 | 2 | 166953619 | T | C | 0.5991 | -0.0121 | 0.0019 | 4.51E-10 | 484680 | 0.993992 |
| rs12994080 | 2 | 171599082 | A | G | 0.36 | 0.0098 | 0.0018 | 4.13E-08 | 718599 | 0.999801 |
| rs1047255 | 2 | 172412008 | C | G | 0.7464 | 0.0116 | 0.002 | 4.77E-09 | 718392 | 0.998337 |
| rs10930502 | 2 | 172890588 | A | G | 0.694 | 0.0128 | 0.0018 | 2.74E-13 | 806321 | 0.995525 |
| rs2044469 | 2 | 174961488 | A | G | 0.6619 | -0.0133 | 0.0018 | 1.19E-13 | 718581 | 0.985863 |
| rs72917533 | 2 | 175238924 | T | C | 0.8204 | 0.0178 | 0.0025 | 5.86E-13 | 484680 | 0.990768 |
| rs262255 | 2 | 180903586 | T | C | 0.3902 | 0.0096 | 0.0017 | 3.19E-08 | 718561 | 0.997926 |
| rs918959 | 2 | 181513729 | A | G | 0.0748 | 0.0201 | 0.0029 | 5.52E-12 | 806589 | 1 |
| rs7588437 | 2 | 181575281 | A | G | 0.3664 | -0.0165 | 0.0017 | 2.32E-22 | 806379 | 0.991305 |
| rs7575118 | 2 | 182653725 | T | C | 0.1561 | 0.014 | 0.0024 | 3.33E-09 | 702749 | 1 |
| rs114049355 | 2 | 183075689 | T | C | 0.0568 | -0.0253 | 0.0043 | 3.47E-09 | 484680 | 0.915186 |
| rs2029085 | 2 | 187207393 | A | G | 0.1068 | -0.0154 | 0.0027 | 1.48E-08 | 718590 | 0.99824 |
| rs2882203 | 2 | 188282330 | A | T | 0.3981 | -0.0097 | 0.0017 | 2.00E-08 | 718551 | 0.993281 |
| rs13001304 | 2 | 192949003 | T | C | 0.6994 | 0.0108 | 0.0019 | 7.98E-09 | 716056 | 0.994259 |
| rs7570446 | 2 | 193801010 | A | C | 0.5397 | 0.0111 | 0.0019 | 4.74E-09 | 484680 | 0.997699 |
| rs6733834 | 2 | 198518709 | C | G | 0.8077 | 0.015 | 0.0021 | 1.54E-12 | 718681 | 0.999084 |
| rs72934680 | 2 | 202893965 | A | G | 0.7823 | -0.0129 | 0.0023 | 2.82E-08 | 484680 | 0.978211 |
| rs10497870 | 2 | 203970283 | A | G | 0.6092 | 0.0121 | 0.0016 | 1.97E-13 | 806733 | 0.995036 |
| rs4482463 | 2 | 205375909 | A | C | 0.917 | -0.031 | 0.0031 | 4.85E-23 | 663850 | 0.985924 |
| rs7569010 | 2 | 206084518 | C | G | 0.4285 | 0.0102 | 0.0017 | 2.69E-09 | 718502 | 0.998446 |
| rs972540 | 2 | 207244783 | A | G | 0.7224 | -0.0131 | 0.0018 | 3.83E-13 | 806774 | 0.99856 |
| rs1263627 | 2 | 207977253 | A | T | 0.7595 | -0.013 | 0.002 | 7.26E-11 | 718483 | 0.990844 |
| rs13419066 | 2 | 208161390 | A | G | 0.287 | 0.0116 | 0.0019 | 8.06E-10 | 718688 | 0.992618 |
| rs17808606 | 2 | 208228309 | T | C | 0.2092 | -0.0117 | 0.002 | 9.05E-09 | 718197 | 0.992971 |
| rs11692326 | 2 | 208263279 | T | C | 0.2302 | 0.0147 | 0.0019 | 1.69E-14 | 801583 | 0.999957 |
| rs79675564 | 2 | 211286896 | A | C | 0.0758 | 0.02 | 0.0036 | 2.31E-08 | 484680 | 1 |
| rs715 | 2 | 211543055 | T | C | 0.6953 | -0.0157 | 0.0019 | 1.34E-16 | 660054 | 0.989401 |
| rs4461202 | 2 | 211795919 | T | C | 0.4546 | 0.0102 | 0.0017 | 1.26E-09 | 718565 | 0.996843 |
| rs7421089 | 2 | 211988412 | T | C | 0.2869 | 0.0129 | 0.0019 | 4.25E-12 | 718688 | 0.998811 |
| rs16825005 | 2 | 212304565 | A | G | 0.2937 | 0.0124 | 0.0018 | 3.51E-12 | 806435 | 0.9928 |
| rs7599312 | 2 | 213413231 | A | G | 0.2737 | -0.0182 | 0.0018 | 1.52E-23 | 806704 | 0.978529 |
| rs4480977 | 2 | 215405343 | T | G | 0.5103 | 0.009 | 0.0016 | 2.51E-08 | 806152 | 0.990036 |
| rs7607369 | 2 | 219279097 | A | G | 0.4076 | 0.0121 | 0.0016 | 8.20E-14 | 801568 | 1 |
| 2_219949184_C_T | 2 | 219949184 | T | C | 0.0399 | -0.0237 | 0.0042 | 1.27E-08 | 792958 | 1 |
| rs6725931 | 2 | 220205146 | T | C | 0.8483 | 0.0187 | 0.0024 | 2.30E-15 | 718689 | 0.993955 |
| rs2280039 | 2 | 220363488 | A | G | 0.6497 | 0.0096 | 0.0017 | 1.71E-08 | 806377 | 0.998768 |
| rs12694772 | 2 | 228929141 | T | C | 0.5933 | -0.011 | 0.0017 | 2.00E-10 | 718629 | 0.995134 |
| rs4973618 | 2 | 229002620 | A | G | 0.6623 | -0.0148 | 0.0018 | 1.25E-16 | 706944 | 0.999799 |
| rs2273999 | 2 | 230582774 | A | G | 0.131 | -0.0158 | 0.0026 | 6.56E-10 | 718616 | 0.997371 |
| rs6720868 | 2 | 230663576 | T | C | 0.3083 | 0.0154 | 0.0018 | 1.82E-17 | 718504 | 0.996885 |
| rs7590444 | 2 | 232721071 | T | C | 0.2239 | 0.0111 | 0.002 | 2.15E-08 | 718511 | 0.992359 |
| rs987071 | 2 | 236786086 | T | C | 0.7662 | -0.0126 | 0.002 | 7.91E-10 | 717268 | 0.990908 |
| rs9808302 | 2 | 236854450 | A | G | 0.5278 | -0.0116 | 0.0019 | 9.05E-10 | 484680 | 0.995017 |
| rs1316369 | 2 | 241351756 | A | G | 0.6607 | 0.0104 | 0.0018 | 7.39E-09 | 718656 | 0.992097 |
| rs4640343 | 2 | 24956027 | A | C | 0.9157 | -0.0293 | 0.0031 | 1.15E-20 | 715177 | 0.990392 |
| rs11125788 | 2 | 25022113 | T | C | 0.0416 | -0.0294 | 0.0049 | 1.66E-09 | 484680 | 0.951114 |
| rs2289090 | 2 | 25048847 | A | G | 0.3228 | -0.013 | 0.0018 | 1.79E-12 | 663845 | 0.999381 |
| rs10182181 | 2 | 25150296 | A | G | 0.5052 | -0.0327 | 0.0016 | 2.45E-91 | 806439 | 0.995942 |
| rs3754861 | 2 | 25393722 | A | C | 0.9208 | 0.0206 | 0.0035 | 6.59E-09 | 484680 | 0.960673 |
| rs752208 | 2 | 25455389 | A | G | 0.2151 | 0.0189 | 0.0024 | 8.50E-16 | 484680 | 0.959902 |
| rs137926936 | 2 | 25483847 | A | G | 0.0547 | -0.0297 | 0.0043 | 3.48E-12 | 484680 | 0.952772 |
| rs4517932 | 2 | 25552070 | T | C | 0.5636 | -0.0104 | 0.0016 | 2.72E-10 | 805530 | 0.989098 |
| rs12468863 | 2 | 26940294 | T | C | 0.512 | -0.0148 | 0.0016 | 6.63E-20 | 806266 | 0.986024 |
| rs1260326 | 2 | 27730940 | T | C | 0.3913 | -0.0107 | 0.0017 | 1.16E-10 | 799274 | 1 |
| rs7567655 | 2 | 28273595 | A | G | 0.9647 | -0.0242 | 0.0043 | 2.19E-08 | 794748 | 0.999641 |
| rs4372836 | 2 | 28973883 | T | C | 0.3259 | 0.0125 | 0.0017 | 7.33E-13 | 806681 | 0.99833 |
| rs6548221 | 2 | 295255 | A | G | 0.2264 | 0.0145 | 0.002 | 1.06E-13 | 806578 | 1 |
| rs17327461 | 2 | 35512183 | T | C | 0.4462 | 0.0122 | 0.0016 | 7.65E-14 | 806655 | 0.995257 |
| rs6740249 | 2 | 355800 | T | C | 0.4895 | 0.0123 | 0.0017 | 4.01E-13 | 717920 | 0.985545 |
| rs3770890 | 2 | 36657992 | T | G | 0.9725 | -0.0297 | 0.0053 | 2.10E-08 | 771730 | 0.966644 |
| rs3770799 | 2 | 36788616 | A | G | 0.6367 | -0.0114 | 0.0018 | 1.21E-10 | 718290 | 0.997273 |
| rs6713781 | 2 | 40291940 | C | G | 0.3987 | -0.0126 | 0.0017 | 2.94E-13 | 718231 | 0.975725 |
| rs6753395 | 2 | 41515704 | C | G | 0.6242 | 0.01 | 0.0018 | 1.68E-08 | 717635 | 0.967939 |
| rs10169594 | 2 | 41637688 | T | C | 0.6475 | -0.012 | 0.0018 | 1.34E-11 | 718035 | 0.989258 |
| rs62106258 | 2 | 417167 | T | C | 0.9534 | 0.0912 | 0.0045 | 1.26E-91 | 484680 | 1 |
| rs7580766 | 2 | 42939351 | A | G | 0.4145 | -0.0094 | 0.0017 | 2.98E-08 | 718322 | 0.996816 |
| rs2685230 | 2 | 437664 | A | G | 0.6347 | -0.0165 | 0.0017 | 5.98E-23 | 806750 | 1 |
| rs698838 | 2 | 44738763 | T | C | 0.6196 | 0.0127 | 0.0018 | 8.75E-13 | 701259 | 1 |
| rs6732079 | 2 | 46877963 | A | T | 0.0944 | 0.0219 | 0.0033 | 1.88E-11 | 484680 | 0.979147 |
| rs35722922 | 2 | 47002226 | A | G | 0.6247 | 0.0158 | 0.002 | 8.12E-16 | 484680 | 0.989474 |
| rs2724862 | 2 | 477100 | A | G | 0.1876 | 0.015 | 0.0024 | 8.34E-10 | 484680 | 0.987552 |
| rs7561278 | 2 | 48954905 | T | C | 0.7668 | 0.0169 | 0.0021 | 4.89E-16 | 717019 | 0.974519 |
| rs930295 | 2 | 50233352 | A | C | 0.1548 | 0.0208 | 0.0023 | 2.03E-19 | 716913 | 0.998216 |
| rs1520455 | 2 | 50692168 | T | C | 0.6133 | -0.0147 | 0.0018 | 1.98E-16 | 705043 | 0.960639 |
| rs12615199 | 2 | 51825253 | A | T | 0.8157 | 0.012 | 0.0021 | 2.10E-08 | 718669 | 0.9944 |
| rs2864823 | 2 | 532756 | T | C | 0.9244 | 0.0284 | 0.0031 | 1.63E-19 | 706790 | 0.992062 |
| rs59428052 | 2 | 53861389 | A | G | 0.8493 | 0.0176 | 0.0028 | 1.99E-10 | 484680 | 0.914647 |
| rs7601895 | 2 | 55281901 | C | G | 0.6899 | 0.0152 | 0.0018 | 1.89E-16 | 715262 | 0.987396 |
| rs13432055 | 2 | 56603985 | T | C | 0.712 | -0.0117 | 0.0018 | 5.72E-11 | 805940 | 0.982301 |
| rs17189185 | 2 | 57364860 | A | C | 0.3792 | 0.0097 | 0.0018 | 3.23E-08 | 718663 | 0.996944 |
| rs1106090 | 2 | 58068741 | A | G | 0.6278 | -0.0132 | 0.0017 | 1.99E-15 | 806645 | 0.986035 |
| rs5021156 | 2 | 5830538 | C | G | 0.1791 | 0.0145 | 0.0025 | 3.18E-09 | 484680 | 0.989947 |
| rs1016756 | 2 | 58677349 | A | G | 0.1321 | 0.0187 | 0.0024 | 1.42E-14 | 806623 | 0.991918 |
| rs4671328 | 2 | 58935282 | T | G | 0.4496 | 0.0214 | 0.0017 | 3.42E-36 | 718386 | 0.980863 |
| rs1861100 | 2 | 59133513 | T | C | 0.7344 | -0.0129 | 0.0019 | 2.68E-11 | 716915 | 0.97605 |
| rs17049820 | 2 | 59139507 | T | C | 0.8821 | 0.0193 | 0.0027 | 4.21E-13 | 710115 | 0.979036 |
| rs13002470 | 2 | 59185112 | A | G | 0.0665 | -0.0225 | 0.0033 | 7.57E-12 | 706770 | 0.983084 |
| rs6545714 | 2 | 59307725 | A | G | 0.6112 | -0.0194 | 0.0016 | 4.01E-32 | 806783 | 0.994954 |
| rs185581 | 2 | 59880605 | A | C | 0.2052 | 0.0133 | 0.0022 | 7.93E-10 | 718689 | 0.994702 |
| 2_60203917_A_C | 2 | 60203917 | A | C | 0.3405 | 0.0122 | 0.0018 | 1.36E-11 | 718643 | 0.996328 |
| rs1861151 | 2 | 60284614 | T | C | 0.224 | 0.0149 | 0.0021 | 4.36E-13 | 706936 | 0.992485 |
| rs359234 | 2 | 60471409 | C | G | 0.3747 | -0.0112 | 0.002 | 1.30E-08 | 484680 | 0.988607 |
| rs1011407 | 2 | 60665768 | A | G | 0.8883 | 0.0172 | 0.0026 | 6.81E-11 | 718569 | 0.997095 |
| rs2195086 | 2 | 60814466 | T | G | 0.8315 | -0.014 | 0.0023 | 1.43E-09 | 717437 | 0.990347 |
| rs10929925 | 2 | 6155557 | A | C | 0.4105 | -0.0142 | 0.0016 | 3.05E-18 | 801209 | 0.995122 |
| 2_619653_C_T | 2 | 619653 | T | C | 0.193 | 0.0171 | 0.0021 | 1.32E-16 | 806276 | 0.988249 |
| rs13417156 | 2 | 62848319 | T | C | 0.5755 | -0.0135 | 0.0017 | 6.90E-16 | 767912 | 0.997128 |
| rs13021737 | 2 | 632348 | A | G | 0.1567 | -0.0578 | 0.0021 | 2.89E-161 | 802967 | 0.999856 |
| rs9784046 | 2 | 651641 | A | C | 0.1337 | 0.0191 | 0.0023 | 2.69E-17 | 794334 | 0.994935 |
| rs11689974 | 2 | 654685 | T | G | 0.1896 | 0.0183 | 0.0021 | 4.25E-18 | 806417 | 0.977854 |
| rs2861685 | 2 | 67837553 | T | C | 0.5864 | 0.0165 | 0.0019 | 7.82E-18 | 484680 | 0.997983 |
| rs4581940 | 2 | 69655790 | T | C | 0.5857 | 0.0101 | 0.0017 | 3.43E-09 | 718608 | 0.997828 |
| rs111311129 | 2 | 710112 | T | C | 0.9428 | 0.0405 | 0.0041 | 1.21E-22 | 484680 | 0.968203 |
| rs934515 | 2 | 79482643 | A | G | 0.1175 | 0.0181 | 0.0026 | 6.64E-12 | 718664 | 0.978234 |
| rs11681570 | 2 | 81851452 | T | G | 0.3314 | 0.0115 | 0.0018 | 1.54E-10 | 718453 | 0.998616 |
| rs12714199 | 2 | 86812549 | T | C | 0.6146 | -0.0141 | 0.0017 | 3.22E-16 | 718671 | 1 |
| rs6759670 | 2 | 950291 | A | C | 0.7072 | -0.012 | 0.0019 | 1.99E-10 | 717122 | 0.992512 |
| rs17535749 | 3 | 10027724 | A | G | 0.1081 | 0.0159 | 0.0027 | 3.46E-09 | 793365 | 0.98898 |
| rs1436344 | 3 | 104606144 | C | G | 0.5703 | 0.0147 | 0.0017 | 1.07E-17 | 718660 | 0.997362 |
| rs16850772 | 3 | 104736837 | T | G | 0.3186 | 0.0113 | 0.0018 | 9.71E-10 | 717441 | 0.991142 |
| rs12490065 | 3 | 10627561 | A | G | 0.214 | -0.0126 | 0.0022 | 1.69E-08 | 674630 | 0.892363 |
| rs28447555 | 3 | 107385668 | T | C | 0.1726 | 0.0159 | 0.0025 | 2.43E-10 | 484680 | 0.990765 |
| rs709524 | 3 | 107609481 | A | G | 0.624 | -0.0097 | 0.0017 | 2.52E-08 | 718698 | 0.994036 |
| rs7640424 | 3 | 107820063 | T | C | 0.3106 | -0.0135 | 0.0018 | 1.23E-14 | 805792 | 0.994649 |
| rs10514751 | 3 | 107893149 | T | C | 0.897 | -0.0158 | 0.0028 | 2.28E-08 | 718658 | 0.986772 |
| 3_108119071_C_T | 3 | 108119071 | T | C | 0.5066 | -0.0113 | 0.0016 | 3.80E-12 | 806456 | 1 |
| rs17681451 | 3 | 114399296 | A | G | 0.0762 | -0.0225 | 0.0031 | 7.37E-13 | 718679 | 0.999114 |
| rs3772934 | 3 | 115417865 | T | C | 0.2938 | 0.0107 | 0.0019 | 2.01E-08 | 706122 | 0.989277 |
| rs17609108 | 3 | 11626464 | T | C | 0.8519 | -0.0152 | 0.0024 | 4.14E-10 | 718635 | 0.972957 |
| rs6808814 | 3 | 116852469 | T | C | 0.7335 | 0.0118 | 0.0019 | 1.31E-09 | 718249 | 0.996566 |
| rs6804181 | 3 | 116937546 | A | T | 0.8215 | 0.0141 | 0.0023 | 6.47E-10 | 717937 | 0.990704 |
| rs768917 | 3 | 117805617 | T | C | 0.681 | -0.0115 | 0.0021 | 2.31E-08 | 484680 | 0.987497 |
| rs779206 | 3 | 118023515 | A | G | 0.7218 | -0.0122 | 0.0019 | 3.28E-10 | 718456 | 0.988175 |
| rs2124499 | 3 | 123093541 | C | G | 0.3682 | -0.0115 | 0.0017 | 7.25E-12 | 802110 | 0.996315 |
| rs10668 | 3 | 123211064 | T | G | 0.5049 | -0.0117 | 0.0017 | 1.40E-11 | 715849 | 0.98006 |
| 3_12393125_C_G | 3 | 12393125 | C | G | 0.8952 | -0.017 | 0.0025 | 6.82E-12 | 806784 | 1 |
| rs10510419 | 3 | 12426936 | T | G | 0.1467 | -0.0168 | 0.0023 | 2.23E-13 | 800535 | 1 |
| rs76594121 | 3 | 128189391 | T | G | 0.9547 | 0.0284 | 0.0047 | 1.64E-09 | 484680 | 0.941255 |
| rs765248 | 3 | 13001931 | A | G | 0.4372 | -0.0113 | 0.0017 | 4.55E-11 | 717434 | 1 |
| rs17295394 | 3 | 131506937 | T | G | 0.2876 | -0.0167 | 0.0019 | 8.51E-19 | 718255 | 0.989728 |
| rs9289408 | 3 | 131606610 | T | C | 0.4729 | -0.0113 | 0.0017 | 1.88E-11 | 718406 | 0.996402 |
| rs80273039 | 3 | 131747494 | T | C | 0.0485 | -0.0262 | 0.0044 | 3.03E-09 | 484680 | 0.995896 |
| rs7631156 | 3 | 131751628 | A | G | 0.3091 | 0.0215 | 0.0018 | 3.33E-32 | 718695 | 0.999306 |
| rs3851998 | 3 | 131876605 | C | G | 0.2574 | 0.0123 | 0.0022 | 1.72E-08 | 484680 | 0.988556 |
| rs1048637 | 3 | 13358171 | T | G | 0.5474 | -0.0094 | 0.0017 | 2.94E-08 | 718169 | 0.999057 |
| rs10935143 | 3 | 134665159 | A | G | 0.439 | -0.0099 | 0.0017 | 6.01E-09 | 718627 | 0.995573 |
| rs7621025 | 3 | 136272246 | T | C | 0.2607 | -0.0187 | 0.0019 | 7.54E-24 | 806690 | 0.999217 |
| rs13066401 | 3 | 136345819 | A | G | 0.8783 | -0.0169 | 0.0025 | 2.96E-11 | 715044 | 0.996665 |
| 3_138091140_A_G | 3 | 138091140 | A | G | 0.1896 | 0.0145 | 0.0021 | 1.50E-12 | 806644 | 0.997529 |
| 3_138122122_C_T | 3 | 138122122 | T | C | 0.1545 | -0.0137 | 0.0022 | 1.14E-09 | 806701 | 1 |
| rs16851483 | 3 | 141275436 | T | G | 0.0717 | 0.0352 | 0.0034 | 4.87E-25 | 718609 | 0.998438 |
| rs1568490 | 3 | 153673515 | T | C | 0.3298 | -0.0103 | 0.0018 | 1.27E-08 | 718664 | 0.992024 |
| rs355777 | 3 | 154034950 | C | G | 0.3984 | 0.0151 | 0.0017 | 2.13E-18 | 718342 | 0.996732 |
| rs4600855 | 3 | 155081940 | A | G | 0.6165 | -0.0107 | 0.0018 | 1.94E-09 | 712855 | 0.967484 |
| rs9826775 | 3 | 156295341 | A | G | 0.8513 | 0.0155 | 0.0024 | 6.61E-11 | 715852 | 0.99832 |
| rs6809307 | 3 | 156862041 | T | C | 0.259 | 0.0139 | 0.0019 | 8.15E-13 | 718683 | 0.99687 |
| rs2255248 | 3 | 15754849 | T | C | 0.5788 | -0.0102 | 0.0016 | 5.02E-10 | 803930 | 0.999406 |
| rs2682406 | 3 | 158026489 | A | T | 0.4117 | -0.0126 | 0.0017 | 4.76E-13 | 713395 | 0.999506 |
| rs2404815 | 3 | 161446857 | T | C | 0.4272 | -0.011 | 0.0017 | 8.09E-11 | 718615 | 0.999631 |
| rs329078 | 3 | 168233318 | A | G | 0.5186 | 0.0107 | 0.0019 | 1.63E-08 | 484680 | 0.995095 |
| rs6779973 | 3 | 170530581 | A | G | 0.8144 | -0.0116 | 0.002 | 1.10E-08 | 806726 | 0.984359 |
| rs8192675 | 3 | 170724883 | T | C | 0.7113 | -0.0155 | 0.0018 | 1.74E-18 | 806712 | 1 |
| rs11923305 | 3 | 171118170 | A | G | 0.3415 | 0.0118 | 0.002 | 3.73E-09 | 484680 | 0.999149 |
| rs39654 | 3 | 173095123 | A | G | 0.4513 | -0.0163 | 0.0017 | 1.75E-21 | 717812 | 0.993302 |
| rs2035831 | 3 | 173683791 | C | G | 0.3339 | -0.0102 | 0.0018 | 1.97E-08 | 706336 | 0.990875 |
| rs2718786 | 3 | 180952891 | A | G | 0.3852 | 0.0115 | 0.0017 | 2.52E-11 | 718568 | 0.995224 |
| rs6443750 | 3 | 181329682 | T | C | 0.1994 | -0.0152 | 0.0021 | 7.25E-13 | 792002 | 1 |
| rs10937094 | 3 | 182297057 | A | G | 0.7109 | 0.0116 | 0.0019 | 4.20E-10 | 718633 | 0.993596 |
| rs262956 | 3 | 183486117 | T | G | 0.3469 | 0.0124 | 0.0018 | 4.10E-12 | 718204 | 0.992447 |
| rs3752904 | 3 | 183996068 | T | C | 0.512 | -0.0116 | 0.0016 | 8.90E-13 | 806771 | 0.999644 |
| rs3087964 | 3 | 185348402 | T | C | 0.3369 | -0.0097 | 0.0017 | 2.70E-08 | 740706 | 0.991114 |
| 3_185763219_A_G | 3 | 185763219 | A | G | 0.1429 | 0.0164 | 0.0024 | 8.49E-12 | 734036 | 0.993483 |
| 3_185834499_A_T | 3 | 185834499 | A | T | 0.1755 | -0.0315 | 0.0021 | 1.45E-50 | 794122 | 0.989581 |
| rs4619804 | 3 | 18674644 | A | C | 0.2723 | -0.0114 | 0.0019 | 2.57E-09 | 718428 | 0.987916 |
| rs80236973 | 3 | 188001014 | T | C | 0.1314 | -0.0156 | 0.0028 | 2.81E-08 | 484680 | 0.997388 |
| rs500216 | 3 | 193598283 | A | G | 0.3733 | -0.0097 | 0.0018 | 3.67E-08 | 717152 | 0.998071 |
| rs11721261 | 3 | 194876993 | T | C | 0.292 | -0.0135 | 0.0019 | 8.39E-13 | 717098 | 0.989243 |
| rs34801745 | 3 | 196115497 | C | G | 0.3585 | 0.0129 | 0.002 | 7.00E-11 | 484680 | 0.998492 |
| rs4858193 | 3 | 20441050 | T | C | 0.7199 | 0.0133 | 0.0019 | 2.44E-12 | 718071 | 0.991727 |
| rs78452735 | 3 | 20754810 | T | C | 0.8602 | -0.0179 | 0.0029 | 1.11E-09 | 484680 | 0.87076 |
| 3_25106437_A_G | 3 | 25106437 | A | G | 0.4301 | -0.0141 | 0.0016 | 7.57E-18 | 806143 | 0.991473 |
| rs11721181 | 3 | 34725482 | T | C | 0.6039 | 0.0098 | 0.0017 | 1.32E-08 | 706889 | 0.998982 |
| rs11921432 | 3 | 35117776 | T | C | 0.8906 | -0.0189 | 0.0027 | 5.38E-12 | 706646 | 0.983901 |
| rs13062093 | 3 | 35667057 | T | G | 0.6319 | -0.0122 | 0.0017 | 2.78E-12 | 718696 | 0.998989 |
| rs7374289 | 3 | 38633071 | T | C | 0.4015 | 0.0092 | 0.0016 | 2.29E-08 | 806622 | 0.982588 |
| rs9816029 | 3 | 41311362 | C | G | 0.3443 | 0.0107 | 0.0018 | 2.63E-09 | 718278 | 0.998311 |
| rs10460960 | 3 | 42308735 | A | G | 0.8907 | 0.0216 | 0.0025 | 5.92E-18 | 805284 | 0.994963 |
| rs28350 | 3 | 42418446 | A | G | 0.1717 | 0.0172 | 0.0022 | 1.07E-14 | 717783 | 0.988403 |
| rs1354992 | 3 | 42496509 | T | C | 0.2962 | -0.0127 | 0.0021 | 1.18E-09 | 484680 | 0.996128 |
| rs4017425 | 3 | 44028764 | T | C | 0.4676 | -0.0118 | 0.0017 | 2.91E-12 | 718438 | 0.992597 |
| rs4682718 | 3 | 44226332 | A | G | 0.8172 | -0.0143 | 0.0022 | 8.10E-11 | 713680 | 0.996395 |
| rs10514710 | 3 | 44883971 | A | G | 0.198 | -0.0116 | 0.002 | 1.27E-08 | 806785 | 0.987141 |
| rs55676934 | 3 | 45222958 | A | G | 0.672 | -0.014 | 0.002 | 5.19E-12 | 484680 | 0.994381 |
| rs17575828 | 3 | 45440944 | A | G | 0.7634 | 0.0117 | 0.002 | 5.15E-09 | 717359 | 0.984145 |
| rs34877991 | 3 | 45643648 | C | G | 0.3969 | -0.0117 | 0.0019 | 1.62E-09 | 484680 | 0.987232 |
| rs7429588 | 3 | 46835250 | T | C | 0.7127 | -0.0129 | 0.0022 | 4.88E-09 | 484680 | 0.900668 |
| rs72906474 | 3 | 47817007 | T | G | 0.5809 | -0.0169 | 0.0019 | 3.50E-18 | 484680 | 0.974734 |
| rs80026423 | 3 | 48734861 | T | C | 0.0572 | 0.0246 | 0.0042 | 6.91E-09 | 484680 | 0.926137 |
| rs62262463 | 3 | 48961019 | A | G | 0.903 | -0.0216 | 0.0032 | 1.83E-11 | 484680 | 0.988991 |
| rs11713193 | 3 | 49924424 | A | G | 0.5156 | 0.0246 | 0.0017 | 3.02E-48 | 718286 | 0.999824 |
| rs71326918 | 3 | 50174844 | A | C | 0.1108 | -0.0233 | 0.003 | 2.03E-14 | 484680 | 0.986496 |
| rs10433609 | 3 | 51311574 | A | T | 0.8146 | 0.0163 | 0.0022 | 3.51E-13 | 717977 | 0.99347 |
| rs3852070 | 3 | 52212661 | C | G | 0.4472 | -0.0105 | 0.0019 | 2.19E-08 | 553131 | 1 |
| rs2710323 | 3 | 52815905 | T | C | 0.5386 | -0.0141 | 0.0016 | 3.65E-18 | 806656 | 0.995711 |
| rs1004807 | 3 | 53140603 | T | C | 0.2956 | -0.0136 | 0.0019 | 3.26E-13 | 717960 | 0.984944 |
| rs115002913 | 3 | 53368786 | T | C | 0.0321 | -0.0309 | 0.0054 | 9.60E-09 | 484680 | 1 |
| rs2680648 | 3 | 53777176 | T | C | 0.7548 | 0.0159 | 0.002 | 2.72E-15 | 718658 | 0.990419 |
| rs12488237 | 3 | 56114861 | T | C | 0.9391 | -0.0225 | 0.0036 | 2.68E-10 | 800924 | 1 |
| rs2365389 | 3 | 61236462 | T | C | 0.3999 | -0.0168 | 0.0016 | 6.49E-25 | 801448 | 0.994259 |
| rs1911746 | 3 | 62100840 | T | C | 0.2095 | 0.0113 | 0.0019 | 4.17E-09 | 806670 | 0.997753 |
| 3_62376645_G_T | 3 | 62376645 | T | G | 0.6312 | 0.0102 | 0.0018 | 9.19E-09 | 705463 | 1 |
| 3_62481063_C_T | 3 | 62481063 | T | C | 0.7314 | 0.0128 | 0.0018 | 2.67E-12 | 794919 | 1 |
| rs925018 | 3 | 62713143 | C | G | 0.6665 | -0.013 | 0.0017 | 4.33E-14 | 806677 | 0.995432 |
| rs1374681 | 3 | 63063005 | T | G | 0.8747 | 0.0159 | 0.0029 | 2.88E-08 | 484680 | 1 |
| rs2371767 | 3 | 64718258 | C | G | 0.2682 | 0.0107 | 0.0018 | 2.08E-09 | 787408 | 0.995637 |
| rs12633819 | 3 | 66448307 | A | G | 0.651 | -0.0095 | 0.0017 | 2.68E-08 | 805793 | 0.989515 |
| rs56038322 | 3 | 69925128 | A | G | 0.3082 | 0.012 | 0.0021 | 6.59E-09 | 484680 | 0.981725 |
| rs11915371 | 3 | 70539559 | A | C | 0.8019 | -0.0154 | 0.0021 | 2.29E-13 | 718311 | 0.996183 |
| rs71298357 | 3 | 70866845 | T | C | 0.9688 | -0.0318 | 0.0055 | 5.70E-09 | 484680 | 1 |
| rs9637498 | 3 | 76674928 | A | G | 0.389 | 0.0099 | 0.0017 | 1.07E-08 | 718247 | 0.995534 |
| rs1523768 | 3 | 77667044 | A | G | 0.6721 | -0.0126 | 0.0017 | 4.35E-13 | 806680 | 0.996867 |
| rs6419734 | 3 | 78458928 | T | C | 0.1448 | 0.0161 | 0.0024 | 3.77E-11 | 718501 | 0.988909 |
| rs3773192 | 3 | 78660056 | T | C | 0.7337 | -0.0127 | 0.0019 | 4.23E-11 | 718539 | 0.99635 |
| rs333493 | 3 | 78857263 | C | G | 0.7129 | 0.0106 | 0.0019 | 1.08E-08 | 718645 | 0.994942 |
| rs6781254 | 3 | 80649139 | T | C | 0.3034 | 0.0105 | 0.0018 | 4.56E-09 | 794430 | 0.995958 |
| rs1554194 | 3 | 8138041 | C | G | 0.5046 | 0.0105 | 0.0017 | 9.05E-10 | 715847 | 0.99493 |
| rs3849570 | 3 | 81792112 | A | C | 0.3541 | 0.0134 | 0.0017 | 7.35E-15 | 769019 | 0.999727 |
| rs7647242 | 3 | 82642734 | T | C | 0.3947 | 0.0129 | 0.0017 | 1.01E-13 | 718607 | 0.998258 |
| rs114593013 | 3 | 84113491 | A | G | 0.944 | 0.0333 | 0.0042 | 1.17E-15 | 484680 | 0.991356 |
| rs7630382 | 3 | 85111108 | T | C | 0.5255 | 0.0145 | 0.0017 | 5.31E-18 | 718504 | 0.992093 |
| 3_85570561_A_G | 3 | 85570561 | A | G | 0.3571 | -0.0135 | 0.0018 | 1.34E-14 | 718675 | 1 |
| rs9818122 | 3 | 85861064 | T | C | 0.7933 | -0.0228 | 0.002 | 3.97E-30 | 806655 | 0.996804 |
| rs2137492 | 3 | 86266408 | A | G | 0.3764 | 0.0127 | 0.0017 | 2.63E-13 | 718674 | 0.994257 |
| rs1006896 | 3 | 88104411 | A | C | 0.8804 | 0.0227 | 0.0027 | 5.22E-17 | 718124 | 1 |
| rs11128058 | 3 | 88733287 | A | C | 0.49 | 0.0128 | 0.0019 | 1.82E-11 | 484680 | 0.990516 |
| rs73139133 | 3 | 89527708 | A | G | 0.2521 | 0.0126 | 0.0022 | 7.82E-09 | 484680 | 0.997224 |
| rs1454687 | 3 | 94038085 | C | G | 0.4817 | 0.0185 | 0.0017 | 6.62E-28 | 718692 | 0.998402 |
| rs59302296 | 3 | 9507314 | A | T | 0.0991 | 0.0217 | 0.0032 | 9.12E-12 | 484680 | 0.998958 |
| rs1229984 | 4 | 100239319 | T | C | 0.0312 | -0.0305 | 0.0052 | 4.72E-09 | 484680 | 1 |
| rs2583401 | 4 | 102204529 | T | C | 0.1608 | 0.0162 | 0.0023 | 1.92E-12 | 718534 | 0.996355 |
| rs201081507 | 4 | 102681041 | A | G | 0.9369 | -0.0311 | 0.0042 | 7.35E-14 | 484680 | 0.851761 |
| rs7377083 | 4 | 102708997 | A | C | 0.4332 | 0.0198 | 0.0018 | 2.51E-28 | 662384 | 0.981024 |
| 4_103188709_C_T | 4 | 103188709 | T | C | 0.082 | 0.0468 | 0.0032 | 3.81E-47 | 806141 | 1 |
| rs10516497 | 4 | 103942714 | A | C | 0.8084 | 0.0125 | 0.002 | 8.88E-10 | 806786 | 0.994763 |
| rs326893 | 4 | 112691776 | T | C | 0.5812 | 0.0121 | 0.0017 | 1.89E-12 | 718672 | 0.997471 |
| rs4834272 | 4 | 113313986 | T | C | 0.6852 | -0.0108 | 0.0017 | 3.46E-10 | 806658 | 0.993378 |
| rs12509234 | 4 | 120319434 | T | C | 0.7214 | -0.0116 | 0.0019 | 3.89E-10 | 718653 | 0.999547 |
| rs4864201 | 4 | 130731284 | T | C | 0.3471 | 0.0137 | 0.0017 | 4.30E-16 | 806465 | 1 |
| rs1296328 | 4 | 137083193 | A | C | 0.4464 | 0.0166 | 0.0017 | 3.49E-22 | 717741 | 0.990364 |
| rs2271391 | 4 | 140787695 | T | C | 0.313 | -0.0126 | 0.0018 | 5.53E-12 | 718016 | 0.98534 |
| rs57800857 | 4 | 140863365 | A | C | 0.6344 | 0.0157 | 0.002 | 3.49E-15 | 484680 | 0.986979 |
| rs35107973 | 4 | 143125948 | T | C | 0.1178 | 0.0175 | 0.0029 | 2.75E-09 | 484680 | 0.998773 |
| rs11724872 | 4 | 144140665 | T | C | 0.6104 | -0.0095 | 0.0016 | 8.34E-09 | 806480 | 0.999728 |
| rs17019336 | 4 | 145333609 | A | T | 0.2291 | -0.0128 | 0.0019 | 2.45E-11 | 804991 | 0.997309 |
| rs1455137 | 4 | 145986668 | A | C | 0.637 | -0.011 | 0.0017 | 3.27E-11 | 806166 | 0.999248 |
| rs3914628 | 4 | 147438019 | T | C | 0.8593 | 0.0165 | 0.0023 | 6.91E-13 | 806780 | 0.984491 |
| rs750090 | 4 | 152931436 | T | C | 0.6258 | 0.0113 | 0.0018 | 2.50E-10 | 718676 | 0.97307 |
| rs6827083 | 4 | 153075491 | A | G | 0.5796 | -0.01 | 0.0016 | 1.11E-09 | 806744 | 0.9984 |
| rs13110266 | 4 | 162129844 | A | G | 0.4037 | -0.0124 | 0.0016 | 3.96E-14 | 806749 | 0.992308 |
| rs11931941 | 4 | 163038864 | A | C | 0.2212 | 0.0125 | 0.002 | 4.75E-10 | 718674 | 0.986168 |
| rs7685628 | 4 | 165310133 | A | T | 0.4014 | 0.0101 | 0.0017 | 6.15E-09 | 718680 | 0.986296 |
| 4_16600664_C_T | 4 | 16600664 | T | C | 0.5164 | -0.01 | 0.0017 | 4.57E-09 | 718491 | 0.988886 |
| 4_171055490_A_G | 4 | 171055490 | A | G | 0.5584 | -0.0099 | 0.0017 | 6.29E-09 | 718661 | 0.997472 |
| rs1522569 | 4 | 171632637 | T | G | 0.8183 | 0.0141 | 0.0022 | 1.60E-10 | 718485 | 0.994005 |
| rs1491333 | 4 | 172186288 | T | G | 0.264 | -0.0107 | 0.0019 | 1.72E-08 | 718688 | 0.980406 |
| rs1437842 | 4 | 173597016 | A | G | 0.4907 | -0.0106 | 0.0017 | 8.49E-10 | 705988 | 0.991595 |
| rs74536181 | 4 | 18005123 | A | G | 0.9643 | -0.0304 | 0.0052 | 3.87E-09 | 484680 | 1 |
| rs6850421 | 4 | 180187034 | A | G | 0.4564 | 0.0113 | 0.0019 | 3.66E-09 | 484680 | 0.997433 |
| rs1477890 | 4 | 18511738 | A | G | 0.4875 | -0.0128 | 0.0017 | 6.12E-14 | 706801 | 0.997236 |
| rs67659742 | 4 | 18756806 | A | G | 0.9217 | 0.0201 | 0.0036 | 2.05E-08 | 484680 | 0.971614 |
| rs35408866 | 4 | 187743245 | A | G | 0.1339 | 0.0159 | 0.0028 | 1.50E-08 | 484680 | 0.989769 |
| rs73249175 | 4 | 20219595 | T | G | 0.8754 | -0.0221 | 0.0029 | 1.62E-14 | 484680 | 0.998856 |
| rs2322459 | 4 | 20260028 | T | G | 0.6738 | 0.0116 | 0.002 | 1.17E-08 | 484680 | 0.993627 |
| rs6850639 | 4 | 21010996 | T | C | 0.2006 | 0.0112 | 0.002 | 2.84E-08 | 802057 | 0.99078 |
| rs3796795 | 4 | 25148136 | T | G | 0.1417 | 0.0152 | 0.0024 | 5.02E-10 | 718500 | 1 |
| rs34811474 | 4 | 25408838 | A | G | 0.2224 | -0.0293 | 0.0023 | 8.50E-38 | 484680 | 1 |
| rs73213501 | 4 | 28514830 | A | C | 0.8263 | 0.0194 | 0.0025 | 1.08E-14 | 484680 | 0.995728 |
| rs12331633 | 4 | 30832319 | T | C | 0.5032 | -0.0114 | 0.0017 | 1.28E-11 | 718440 | 0.996873 |
| rs6448733 | 4 | 31028190 | A | G | 0.6146 | -0.0127 | 0.0018 | 5.34E-13 | 718529 | 0.963125 |
| rs2051559 | 4 | 3298800 | T | C | 0.8588 | -0.0167 | 0.0025 | 3.78E-11 | 718374 | 0.991953 |
| rs6852808 | 4 | 34926958 | T | C | 0.7026 | -0.0117 | 0.0021 | 1.86E-08 | 484680 | 0.995199 |
| rs3209570 | 4 | 38699657 | A | G | 0.3715 | -0.0145 | 0.0017 | 8.77E-17 | 718433 | 0.976935 |
| rs12498157 | 4 | 44430025 | A | T | 0.7201 | 0.0117 | 0.0019 | 1.10E-09 | 718474 | 0.990189 |
| rs979580 | 4 | 45127762 | T | G | 0.0606 | -0.0201 | 0.0037 | 3.72E-08 | 718125 | 0.996405 |
| rs1512300 | 4 | 45162103 | T | C | 0.8921 | 0.0147 | 0.0025 | 7.57E-09 | 806796 | 0.984238 |
| rs10938397 | 4 | 45182527 | A | G | 0.57 | -0.0322 | 0.0016 | 2.42E-86 | 805635 | 1 |
| rs7671291 | 4 | 45235743 | A | G | 0.9053 | -0.0202 | 0.0033 | 6.46E-10 | 484680 | 0.984962 |
| rs784944 | 4 | 52927229 | A | G | 0.2754 | 0.0129 | 0.0019 | 7.52E-12 | 718536 | 0.993376 |
| rs6832762 | 4 | 53287576 | A | G | 0.5093 | -0.0101 | 0.0017 | 3.02E-09 | 713774 | 0.997512 |
| rs1492767 | 4 | 55221467 | T | C | 0.4681 | 0.0095 | 0.0016 | 3.55E-09 | 806046 | 0.998393 |
| rs2192158 | 4 | 55505360 | A | G | 0.4492 | 0.0137 | 0.0017 | 7.42E-16 | 718510 | 0.997295 |
| rs1119950 | 4 | 60228517 | A | G | 0.5688 | -0.0113 | 0.0019 | 1.27E-09 | 608138 | 0.998433 |
| rs1346841 | 4 | 65651730 | A | G | 0.4116 | -0.0126 | 0.0017 | 3.18E-13 | 718293 | 0.989028 |
| rs17735539 | 4 | 67661511 | A | G | 0.2098 | -0.012 | 0.0022 | 2.83E-08 | 718548 | 0.998326 |
| rs10002111 | 4 | 67815504 | A | G | 0.2227 | 0.0125 | 0.0021 | 1.26E-09 | 718668 | 0.993308 |
| rs10033843 | 4 | 77028783 | A | G | 0.215 | 0.014 | 0.0021 | 1.07E-11 | 714618 | 0.999227 |
| rs35851183 | 4 | 80717182 | A | G | 0.6424 | -0.0121 | 0.002 | 1.07E-09 | 484680 | 0.991377 |
| rs4148155 | 4 | 89054667 | A | G | 0.8867 | 0.0192 | 0.0026 | 1.34E-13 | 806326 | 0.999679 |
| rs6819344 | 4 | 89197519 | A | C | 0.3908 | 0.0102 | 0.0016 | 5.08E-10 | 806778 | 0.996923 |
| rs1903579 | 4 | 91253956 | C | G | 0.5436 | 0.0108 | 0.0017 | 1.83E-10 | 718558 | 0.997748 |
| rs2870710 | 4 | 94411606 | C | G | 0.8057 | 0.0155 | 0.0022 | 9.41E-13 | 706936 | 0.993995 |
| rs7685048 | 4 | 95027784 | T | C | 0.4742 | -0.0101 | 0.0017 | 2.34E-09 | 718663 | 0.99943 |
| rs2241743 | 4 | 96091524 | A | G | 0.5699 | -0.0106 | 0.0016 | 8.57E-11 | 805855 | 1 |
| rs12513212 | 4 | 96149346 | A | C | 0.5913 | -0.0102 | 0.0017 | 4.57E-09 | 718274 | 0.993503 |
| rs4703019 | 5 | 102053558 | A | G | 0.3914 | 0.0102 | 0.0016 | 4.93E-10 | 806351 | 0.998066 |
| rs2447832 | 5 | 103933473 | T | C | 0.4433 | 0.0109 | 0.0017 | 1.88E-10 | 718663 | 0.997119 |
| rs11739877 | 5 | 105876806 | T | C | 0.6231 | 0.0116 | 0.0018 | 4.01E-11 | 718632 | 1 |
| 5_106379106_C_T | 5 | 106379106 | T | C | 0.6155 | 0.0104 | 0.0017 | 1.93E-09 | 718393 | 1 |
| rs2410956 | 5 | 106535576 | T | G | 0.8078 | 0.0127 | 0.0021 | 2.83E-09 | 718523 | 0.992444 |
| rs6888194 | 5 | 106910657 | T | C | 0.8443 | -0.0127 | 0.0023 | 3.58E-08 | 718692 | 0.993134 |
| rs12514413 | 5 | 107323866 | T | C | 0.7654 | -0.0155 | 0.002 | 2.69E-14 | 718397 | 0.98836 |
| rs40067 | 5 | 107439012 | A | G | 0.1701 | -0.0252 | 0.0023 | 9.73E-29 | 714059 | 0.988462 |
| rs400277 | 5 | 108675737 | T | C | 0.3879 | -0.0097 | 0.0017 | 2.28E-08 | 718579 | 0.998921 |
| rs26545 | 5 | 111311760 | T | G | 0.6923 | -0.0107 | 0.0018 | 3.90E-09 | 718540 | 0.990737 |
| rs459552 | 5 | 112176756 | A | T | 0.7747 | -0.0133 | 0.0019 | 8.36E-12 | 797086 | 1 |
| rs4705543 | 5 | 112453071 | T | C | 0.591 | -0.0101 | 0.0017 | 3.87E-09 | 718450 | 0.990001 |
| rs6594734 | 5 | 112884752 | A | G | 0.193 | 0.0124 | 0.0021 | 6.58E-09 | 718700 | 0.99931 |
| rs1402025 | 5 | 113987898 | T | C | 0.7795 | -0.0117 | 0.002 | 7.59E-09 | 718615 | 0.996481 |
| rs9327000 | 5 | 115709156 | A | C | 0.6735 | 0.0112 | 0.0018 | 5.22E-10 | 718629 | 0.993882 |
| rs4895231 | 5 | 119388659 | C | G | 0.4669 | 0.0121 | 0.0017 | 2.49E-12 | 715989 | 0.990682 |
| rs1582931 | 5 | 122657199 | A | G | 0.4745 | -0.0126 | 0.0017 | 1.91E-13 | 717936 | 0.979289 |
| rs6864049 | 5 | 124330522 | A | G | 0.489 | -0.0121 | 0.0016 | 1.48E-13 | 806604 | 0.978247 |
| rs1363695 | 5 | 130378027 | T | C | 0.2359 | -0.0125 | 0.002 | 8.06E-10 | 718676 | 0.981511 |
| rs34914000 | 5 | 131148950 | T | C | 0.7908 | -0.0133 | 0.0023 | 1.18E-08 | 484680 | 1 |
| rs329124 | 5 | 133865452 | A | G | 0.5893 | 0.0135 | 0.0017 | 4.26E-15 | 718669 | 0.995677 |
| rs13163306 | 5 | 136571959 | A | G | 0.4573 | -0.0104 | 0.0017 | 7.67E-10 | 718686 | 0.996244 |
| 5_137667345_A_C | 5 | 137667345 | A | C | 0.8101 | 0.0138 | 0.0022 | 1.84E-10 | 718509 | 0.997786 |
| rs13174863 | 5 | 139080745 | A | G | 0.8542 | -0.0197 | 0.0023 | 1.94E-17 | 793550 | 0.978433 |
| rs2074613 | 5 | 139714564 | T | C | 0.5662 | 0.0115 | 0.0017 | 1.84E-11 | 715988 | 1 |
| rs4912763 | 5 | 140972117 | T | C | 0.2579 | 0.0127 | 0.0022 | 5.73E-09 | 484680 | 0.998932 |
| rs3853474 | 5 | 141816555 | T | C | 0.3867 | -0.0097 | 0.0017 | 2.72E-08 | 718555 | 1 |
| rs4912652 | 5 | 142888684 | T | C | 0.5683 | -0.0098 | 0.0017 | 1.29E-08 | 718032 | 0.993353 |
| rs2190788 | 5 | 144484261 | T | G | 0.3089 | 0.0135 | 0.0018 | 2.43E-13 | 717923 | 0.987566 |
| rs2910026 | 5 | 152529936 | T | C | 0.7222 | -0.0132 | 0.0021 | 5.69E-10 | 484680 | 0.994216 |
| rs2964009 | 5 | 153211441 | A | G | 0.5389 | 0.0105 | 0.0016 | 7.52E-11 | 806800 | 0.997054 |
| rs386841 | 5 | 153366603 | T | C | 0.3631 | -0.0112 | 0.0018 | 1.36E-10 | 718644 | 0.999976 |
| rs192398932 | 5 | 153513975 | T | G | 0.9695 | 0.0326 | 0.0056 | 5.75E-09 | 484680 | 0.978184 |
| rs7715256 | 5 | 153537893 | T | G | 0.5644 | -0.0158 | 0.0016 | 3.98E-22 | 806764 | 0.999087 |
| 5_158460212_A_G | 5 | 158460212 | A | G | 0.4449 | -0.0101 | 0.0016 | 6.08E-10 | 805693 | 0.996461 |
| rs4921301 | 5 | 159984492 | T | C | 0.2127 | -0.0129 | 0.0024 | 4.03E-08 | 484680 | 0.9722 |
| rs2861089 | 5 | 164557954 | A | T | 0.3806 | 0.0105 | 0.0017 | 1.55E-09 | 718566 | 0.999072 |
| rs12519652 | 5 | 165243019 | T | C | 0.6587 | 0.0097 | 0.0017 | 1.58E-08 | 801200 | 0.987851 |
| rs34774377 | 5 | 167001890 | T | C | 0.884 | 0.0164 | 0.003 | 3.64E-08 | 484680 | 1 |
| rs729387 | 5 | 167844571 | A | G | 0.6696 | 0.0116 | 0.002 | 8.17E-09 | 484680 | 0.998815 |
| rs17070611 | 5 | 168343675 | A | G | 0.9787 | 0.0334 | 0.0061 | 4.22E-08 | 701010 | 0.947947 |
| rs2053682 | 5 | 170599327 | A | C | 0.6784 | 0.017 | 0.0018 | 2.59E-20 | 717007 | 0.988057 |
| rs17695092 | 5 | 173337853 | T | G | 0.7081 | 0.0114 | 0.0018 | 1.40E-10 | 790186 | 0.999558 |
| rs12659398 | 5 | 173386203 | T | C | 0.0685 | 0.0195 | 0.0034 | 1.40E-08 | 656144 | 0.979683 |
| 5_176527577_G_T | 5 | 176527577 | T | G | 0.3707 | -0.0113 | 0.0017 | 8.14E-11 | 767716 | 0.983643 |
| rs12055154 | 5 | 176675423 | A | G | 0.9725 | -0.0322 | 0.0054 | 2.30E-09 | 714340 | 0.998176 |
| rs6890310 | 5 | 27193573 | A | G | 0.2923 | -0.0119 | 0.0019 | 3.29E-10 | 718622 | 0.992123 |
| rs698147 | 5 | 3513485 | A | G | 0.447 | 0.0116 | 0.0017 | 9.67E-12 | 716805 | 0.994274 |
| rs7730004 | 5 | 43191033 | T | C | 0.6614 | 0.0139 | 0.0018 | 1.46E-14 | 718548 | 0.995424 |
| rs779850 | 5 | 43383398 | T | C | 0.1895 | 0.0127 | 0.0022 | 4.10E-09 | 717597 | 0.969115 |
| rs12189178 | 5 | 50914726 | T | C | 0.0343 | 0.0348 | 0.0046 | 4.30E-14 | 805880 | 0.995327 |
| rs150215901 | 5 | 50935903 | A | T | 0.0414 | -0.0275 | 0.0049 | 1.54E-08 | 484680 | 0.970231 |
| rs4865796 | 5 | 53272664 | A | G | 0.7044 | -0.0096 | 0.0018 | 4.21E-08 | 800901 | 1 |
| rs6879326 | 5 | 59208302 | T | C | 0.4798 | -0.0095 | 0.0017 | 2.03E-08 | 717898 | 0.997663 |
| rs7714712 | 5 | 60732552 | A | C | 0.3678 | -0.0112 | 0.0018 | 2.31E-10 | 718578 | 0.999035 |
| rs669245 | 5 | 62820508 | T | C | 0.4131 | 0.0097 | 0.0017 | 1.69E-08 | 718667 | 0.996602 |
| rs4700608 | 5 | 63026280 | T | C | 0.5236 | -0.0155 | 0.0017 | 4.32E-20 | 718693 | 0.999546 |
| rs260995 | 5 | 63043437 | T | C | 0.3937 | 0.0106 | 0.0018 | 2.75E-09 | 783539 | 0.919555 |
| rs10461497 | 5 | 63942398 | T | C | 0.518 | 0.0117 | 0.0017 | 3.94E-12 | 718011 | 0.992758 |
| rs10471636 | 5 | 63979856 | A | G | 0.5228 | -0.014 | 0.0017 | 1.00E-15 | 717235 | 0.956941 |
| 5_64322549_A_G | 5 | 64322549 | A | G | 0.2488 | -0.0118 | 0.002 | 1.59E-09 | 718235 | 0.994245 |
| rs249612 | 5 | 66200783 | T | C | 0.7138 | 0.0153 | 0.0021 | 4.91E-13 | 484680 | 0.990225 |
| 5_74302658_A_G | 5 | 74302658 | A | G | 0.6178 | 0.0137 | 0.0018 | 1.15E-14 | 716229 | 0.99047 |
| 5_75015242_G_T | 5 | 75015242 | T | G | 0.6298 | 0.0276 | 0.0017 | 1.17E-61 | 806699 | 1 |
| rs1525769 | 5 | 75158737 | C | G | 0.8775 | -0.0168 | 0.0025 | 2.69E-11 | 706889 | 0.986857 |
| rs6896926 | 5 | 75169264 | T | C | 0.9073 | 0.0172 | 0.003 | 1.17E-08 | 718703 | 0.983917 |
| rs252749 | 5 | 77389973 | A | G | 0.2365 | -0.0112 | 0.002 | 1.43E-08 | 718690 | 0.997643 |
| rs326205 | 5 | 7852282 | A | G | 0.5076 | 0.0095 | 0.0017 | 3.51E-08 | 718644 | 0.942143 |
| rs13182474 | 5 | 80861777 | C | G | 0.3114 | -0.0149 | 0.0018 | 1.88E-16 | 718652 | 0.992449 |
| rs10942474 | 5 | 86164433 | A | T | 0.5207 | 0.0111 | 0.0019 | 9.84E-09 | 484680 | 0.96551 |
| rs323742 | 5 | 86769015 | A | T | 0.9692 | -0.0394 | 0.0054 | 2.91E-13 | 709016 | 0.976189 |
| rs12520006 | 5 | 87100609 | A | G | 0.9142 | -0.0192 | 0.0031 | 4.52E-10 | 697686 | 1 |
| rs6870983 | 5 | 87697533 | T | C | 0.2075 | -0.0208 | 0.0019 | 4.90E-27 | 806785 | 0.993782 |
| rs16903232 | 5 | 87831734 | T | C | 0.1008 | 0.0161 | 0.0027 | 4.88E-09 | 806375 | 0.999215 |
| rs1501673 | 5 | 87963600 | A | G | 0.1355 | 0.0289 | 0.0025 | 2.73E-31 | 718652 | 0.99626 |
| rs186233 | 5 | 88133919 | A | G | 0.253 | 0.0118 | 0.002 | 4.25E-09 | 716687 | 0.990083 |
| rs16867703 | 5 | 88799143 | T | G | 0.625 | -0.0146 | 0.0018 | 8.41E-17 | 718463 | 0.999076 |
| rs2009416 | 5 | 92415111 | T | C | 0.3645 | -0.0119 | 0.0018 | 1.44E-11 | 718639 | 0.998487 |
| rs56821385 | 5 | 92611208 | T | C | 0.971 | -0.0337 | 0.0057 | 2.83E-09 | 484680 | 1 |
| rs159032 | 5 | 94206202 | T | C | 0.2325 | 0.0124 | 0.002 | 6.24E-10 | 709303 | 0.997902 |
| rs17383803 | 5 | 95393883 | T | C | 0.9116 | -0.0198 | 0.003 | 5.02E-11 | 701971 | 1 |
| rs7713317 | 5 | 95716722 | A | G | 0.7198 | -0.0166 | 0.0018 | 1.96E-20 | 802425 | 0.994974 |
| rs1837269 | 5 | 95859144 | T | C | 0.5931 | -0.015 | 0.0017 | 2.66E-18 | 718679 | 0.997496 |
| rs3822683 | 5 | 96080883 | A | G | 0.7851 | 0.0131 | 0.002 | 1.45E-10 | 718354 | 0.987198 |
| rs325220 | 5 | 98304564 | A | G | 0.2976 | -0.0106 | 0.0018 | 3.56E-09 | 805094 | 0.99599 |
| rs57989773 | 6 | 100629078 | T | C | 0.7576 | -0.0142 | 0.0023 | 5.63E-10 | 484680 | 0.937577 |
| rs12209887 | 6 | 101156806 | A | G | 0.463 | 0.0114 | 0.0017 | 1.78E-11 | 718123 | 0.994461 |
| rs1417665 | 6 | 104816944 | T | C | 0.8026 | 0.0186 | 0.0022 | 5.49E-18 | 718622 | 0.988847 |
| rs768023 | 6 | 108876002 | A | G | 0.6033 | 0.0161 | 0.0016 | 1.13E-22 | 806650 | 0.99589 |
| rs9387640 | 6 | 119508871 | T | C | 0.383 | -0.011 | 0.0017 | 8.38E-11 | 798171 | 1 |
| 6_120213880_A_G | 6 | 120213880 | A | G | 0.6735 | 0.0143 | 0.0017 | 2.11E-16 | 806795 | 0.99263 |
| 6_12124855_A_G | 6 | 12124855 | A | G | 0.3374 | -0.0144 | 0.0017 | 5.50E-17 | 806782 | 1 |
| rs1871329 | 6 | 124911534 | A | G | 0.7632 | -0.0131 | 0.002 | 1.03E-10 | 718167 | 0.986416 |
| rs1159974 | 6 | 126090277 | T | C | 0.4904 | -0.011 | 0.0017 | 7.75E-11 | 718359 | 1 |
| rs17802893 | 6 | 12716559 | A | T | 0.0688 | -0.021 | 0.0038 | 3.84E-08 | 484680 | 0.965098 |
| 6_130349119_C_T | 6 | 130349119 | T | C | 0.7656 | -0.0128 | 0.0019 | 2.06E-11 | 806801 | 1 |
| rs11757278 | 6 | 13180454 | T | C | 0.6961 | 0.0133 | 0.0019 | 6.92E-13 | 718346 | 0.998245 |
| rs2246012 | 6 | 131898208 | T | C | 0.8512 | -0.0161 | 0.0022 | 1.15E-13 | 806786 | 1 |
| rs9376577 | 6 | 141426995 | T | C | 0.943 | -0.0229 | 0.0041 | 2.80E-08 | 484680 | 0.988232 |
| rs11754747 | 6 | 141494602 | T | C | 0.2688 | 0.0123 | 0.0019 | 1.18E-10 | 804870 | 0.986554 |
| rs6922607 | 6 | 142703483 | A | G | 0.808 | -0.013 | 0.0022 | 1.85E-09 | 706729 | 0.998426 |
| rs765875 | 6 | 143185683 | T | C | 0.4689 | -0.0132 | 0.0017 | 1.08E-14 | 718653 | 0.996785 |
| rs2256216 | 6 | 147471730 | A | G | 0.454 | -0.0104 | 0.0018 | 3.67E-09 | 718379 | 0.95395 |
| rs2185027 | 6 | 153381622 | A | C | 0.6983 | -0.015 | 0.0018 | 1.40E-17 | 803718 | 0.999933 |
| rs12207125 | 6 | 154301575 | A | G | 0.7756 | 0.0136 | 0.0023 | 2.93E-09 | 484680 | 0.990129 |
| rs10499276 | 6 | 154309808 | T | C | 0.1128 | 0.018 | 0.0024 | 1.79E-13 | 806750 | 0.997672 |
| rs9478671 | 6 | 155987825 | A | G | 0.7945 | -0.0117 | 0.0021 | 2.39E-08 | 718492 | 0.992394 |
| rs487152 | 6 | 160774486 | A | C | 0.4973 | 0.0108 | 0.0016 | 2.98E-11 | 798039 | 0.994786 |
| rs13191362 | 6 | 163033350 | A | G | 0.8572 | 0.0235 | 0.0025 | 4.08E-21 | 806582 | 0.994597 |
| rs9364687 | 6 | 163817911 | T | G | 0.4003 | -0.0097 | 0.0016 | 3.03E-09 | 806594 | 0.99359 |
| rs9356132 | 6 | 164102214 | T | C | 0.6976 | -0.0099 | 0.0018 | 1.79E-08 | 806398 | 0.991598 |
| rs3846783 | 6 | 166395367 | A | C | 0.1266 | 0.0146 | 0.0025 | 1.08E-08 | 711400 | 0.995896 |
| rs5017416 | 6 | 18492350 | T | G | 0.0504 | 0.0285 | 0.0044 | 1.08E-10 | 484680 | 0.970439 |
| 6_19759587_C_T | 6 | 19759587 | T | C | 0.7202 | 0.0108 | 0.0019 | 1.92E-08 | 718155 | 0.996094 |
| rs3806114 | 6 | 20482335 | A | G | 0.6916 | -0.012 | 0.0018 | 1.46E-11 | 804965 | 0.976095 |
| rs11753081 | 6 | 20705590 | T | G | 0.8297 | 0.0119 | 0.0021 | 1.91E-08 | 801622 | 0.999097 |
| rs7760082 | 6 | 21919387 | A | G | 0.6519 | -0.0116 | 0.0018 | 7.49E-11 | 718666 | 0.995143 |
| rs767943 | 6 | 23446691 | A | C | 0.2759 | 0.0109 | 0.0019 | 1.13E-08 | 717938 | 0.981533 |
| rs1355459 | 6 | 23871700 | A | G | 0.3157 | 0.0112 | 0.0018 | 9.56E-10 | 718658 | 0.994293 |
| rs3813680 | 6 | 24848086 | A | G | 0.8456 | 0.0139 | 0.0024 | 4.42E-09 | 718491 | 1 |
| rs34706906 | 6 | 25144242 | T | C | 0.0994 | -0.0181 | 0.0032 | 2.52E-08 | 484680 | 0.959534 |
| rs4712972 | 6 | 25772047 | A | G | 0.1536 | 0.0139 | 0.0023 | 1.27E-09 | 806563 | 1 |
| rs2229768 | 6 | 26104280 | T | C | 0.7765 | 0.0134 | 0.0019 | 2.09E-12 | 806796 | 1 |
| 6_26758479_C_T | 6 | 26758479 | T | C | 0.2539 | -0.0131 | 0.002 | 2.17E-11 | 718687 | 1 |
| rs9257175 | 6 | 28746945 | C | G | 0.8141 | 0.0139 | 0.0021 | 1.05E-10 | 718636 | 0.999537 |
| rs2517827 | 6 | 29832846 | A | C | 0.3131 | 0.0137 | 0.0021 | 2.20E-11 | 484680 | 0.999566 |
| 6_30105154_A_G | 6 | 30105154 | A | G | 0.9779 | -0.036 | 0.0065 | 2.70E-08 | 484680 | 1 |
| rs56248798 | 6 | 30597083 | T | C | 0.0388 | 0.0287 | 0.005 | 9.76E-09 | 484680 | 0.968731 |
| 6_31119589_G_T | 6 | 31119589 | T | G | 0.2516 | -0.0113 | 0.0018 | 5.87E-10 | 803475 | 1 |
| rs4947314 | 6 | 31393374 | T | G | 0.83 | -0.0139 | 0.0025 | 3.58E-08 | 484680 | 0.99986 |
| rs2178899 | 6 | 31606756 | A | T | 0.8833 | 0.0243 | 0.0025 | 5.44E-23 | 797281 | 0.999857 |
| rs3130048 | 6 | 31613739 | T | C | 0.7315 | -0.0169 | 0.0018 | 2.02E-20 | 804682 | 1 |
| rs35880697 | 6 | 32433759 | T | C | 0.1588 | -0.0164 | 0.0027 | 1.38E-09 | 484680 | 0.916585 |
| rs9270549 | 6 | 32560440 | A | G | 0.4629 | 0.0109 | 0.0019 | 1.85E-08 | 484680 | 0.955465 |
| rs9277992 | 6 | 33312455 | A | G | 0.1878 | 0.0204 | 0.0025 | 7.45E-17 | 484680 | 0.983057 |
| 6_33543409_A_C | 6 | 33543409 | A | C | 0.6303 | 0.0118 | 0.0016 | 7.27E-13 | 805330 | 1 |
| rs943466 | 6 | 33731787 | A | G | 0.2398 | -0.0146 | 0.0019 | 1.39E-14 | 805113 | 0.995918 |
| rs10947458 | 6 | 33816451 | T | C | 0.2541 | -0.0121 | 0.0022 | 3.92E-08 | 484680 | 0.975181 |
| rs77111942 | 6 | 33833665 | A | G | 0.0288 | -0.033 | 0.0057 | 7.78E-09 | 484680 | 0.98655 |
| rs41269026 | 6 | 34208773 | A | C | 0.0459 | -0.0277 | 0.0046 | 1.15E-09 | 484680 | 0.998165 |
| 6_34302869_A_G | 6 | 34302869 | A | G | 0.8094 | -0.0187 | 0.002 | 8.83E-21 | 805400 | 1 |
| rs73405697 | 6 | 34470396 | T | C | 0.9092 | -0.0302 | 0.0033 | 7.93E-20 | 484680 | 0.994737 |
| rs12197759 | 6 | 34475822 | A | G | 0.7464 | 0.0143 | 0.002 | 5.21E-13 | 704515 | 0.990346 |
| rs2744974 | 6 | 34579431 | T | C | 0.3239 | 0.0261 | 0.0017 | 1.28E-51 | 804150 | 0.997972 |
| rs41270066 | 6 | 35442122 | T | C | 0.0499 | 0.0292 | 0.0044 | 2.53E-11 | 484680 | 0.995496 |
| rs9394312 | 6 | 35672330 | C | G | 0.4841 | 0.0115 | 0.0017 | 1.46E-11 | 717796 | 1 |
| rs17757975 | 6 | 38214150 | T | C | 0.8462 | 0.0155 | 0.0024 | 1.57E-10 | 718688 | 0.995585 |
| rs4714290 | 6 | 40003502 | T | C | 0.7046 | 0.0112 | 0.0019 | 2.44E-09 | 715538 | 0.996559 |
| 6_40365601_A_G | 6 | 40365601 | A | G | 0.4073 | 0.0189 | 0.0017 | 1.97E-29 | 718640 | 0.997925 |
| rs2436758 | 6 | 40377648 | A | G | 0.3839 | -0.011 | 0.002 | 1.82E-08 | 484680 | 0.989794 |
| rs2183819 | 6 | 40857115 | T | G | 0.4525 | 0.009 | 0.0016 | 3.59E-08 | 806747 | 1 |
| rs7748777 | 6 | 41133806 | A | G | 0.4617 | 0.0107 | 0.0016 | 4.18E-11 | 806317 | 0.993813 |
| rs12193797 | 6 | 41748352 | A | G | 0.8704 | 0.0168 | 0.0028 | 3.33E-09 | 484680 | 0.995243 |
| rs6931385 | 6 | 42677834 | A | C | 0.4815 | 0.0111 | 0.0017 | 5.64E-11 | 718437 | 0.994224 |
| rs35679149 | 6 | 43604167 | A | G | 0.9742 | 0.0413 | 0.006 | 6.89E-12 | 484680 | 1 |
| 6_43764551_C_T | 6 | 43764551 | T | C | 0.4735 | -0.0129 | 0.0017 | 5.15E-15 | 797930 | 0.980433 |
| rs12210292 | 6 | 44925293 | T | C | 0.7639 | 0.0117 | 0.002 | 3.04E-09 | 718185 | 0.990792 |
| rs10498767 | 6 | 46363557 | C | G | 0.4537 | -0.0138 | 0.0017 | 5.03E-16 | 718665 | 0.99299 |
| rs9349538 | 6 | 50357998 | A | G | 0.88 | -0.0201 | 0.0027 | 3.90E-14 | 717240 | 0.997341 |
| rs280322 | 6 | 50633381 | T | C | 0.197 | -0.0169 | 0.002 | 1.63E-16 | 794788 | 0.99067 |
| rs78648104 | 6 | 50683009 | T | C | 0.9154 | 0.0274 | 0.0034 | 1.29E-15 | 484680 | 1 |
| 6_50798526_C_T | 6 | 50798526 | T | C | 0.1557 | 0.0408 | 0.0021 | 1.82E-83 | 806768 | 1 |
| rs283579 | 6 | 50954071 | A | G | 0.284 | -0.0187 | 0.0018 | 1.51E-25 | 806816 | 0.997615 |
| rs2504684 | 6 | 51142230 | T | C | 0.9702 | -0.0275 | 0.0048 | 9.17E-09 | 804964 | 0.982289 |
| rs2478879 | 6 | 51152988 | A | G | 0.6075 | 0.0169 | 0.0017 | 2.36E-22 | 718636 | 0.987907 |
| rs2784187 | 6 | 51468139 | T | C | 0.4088 | -0.0142 | 0.0017 | 1.47E-16 | 718522 | 0.998236 |
| rs9395747 | 6 | 51799902 | T | G | 0.709 | -0.0149 | 0.0019 | 1.72E-15 | 717945 | 0.992197 |
| rs4278019 | 6 | 53693410 | A | T | 0.2797 | 0.0104 | 0.0019 | 2.84E-08 | 718678 | 0.998209 |
| rs816367 | 6 | 53995542 | C | G | 0.6491 | -0.0107 | 0.0017 | 1.02E-09 | 718691 | 0.998593 |
| rs4712089 | 6 | 54970880 | A | T | 0.4107 | 0.0096 | 0.0017 | 3.67E-08 | 718590 | 0.998471 |
| rs9370410 | 6 | 55171842 | A | G | 0.7293 | 0.0105 | 0.0019 | 2.58E-08 | 717054 | 1 |
| rs9378999 | 6 | 5979069 | T | C | 0.3501 | -0.0116 | 0.002 | 6.91E-09 | 484680 | 0.985214 |
| rs7751781 | 6 | 62275462 | T | C | 0.407 | 0.0095 | 0.0017 | 4.01E-08 | 714856 | 0.996573 |
| rs2622274 | 6 | 64240516 | T | G | 0.4553 | -0.0107 | 0.0017 | 3.23E-10 | 718675 | 0.996904 |
| rs72910629 | 6 | 69761994 | A | G | 0.8617 | -0.016 | 0.0028 | 7.89E-09 | 484680 | 0.98604 |
| rs6916553 | 6 | 70146918 | A | G | 0.2352 | -0.0121 | 0.002 | 2.64E-09 | 713926 | 0.997884 |
| rs6924504 | 6 | 73174691 | T | C | 0.283 | -0.0117 | 0.0021 | 3.39E-08 | 484680 | 0.987366 |
| rs13206405 | 6 | 73663814 | A | C | 0.1909 | 0.0122 | 0.0021 | 1.02E-08 | 718669 | 1 |
| rs9688431 | 6 | 73922654 | T | C | 0.9305 | 0.0212 | 0.0034 | 3.62E-10 | 801216 | 0.996838 |
| rs9294260 | 6 | 83433228 | A | G | 0.4689 | 0.014 | 0.0016 | 8.16E-18 | 806412 | 0.986212 |
| rs209403 | 6 | 83544677 | A | C | 0.1495 | 0.0133 | 0.0024 | 2.16E-08 | 718325 | 0.998837 |
| rs9450537 | 6 | 87566319 | A | G | 0.3848 | 0.0102 | 0.0018 | 1.32E-08 | 712007 | 0.995798 |
| rs9362662 | 6 | 90296588 | A | G | 0.4696 | 0.0103 | 0.0017 | 1.44E-09 | 717763 | 0.99079 |
| rs16882001 | 6 | 90322237 | A | G | 0.9499 | -0.0227 | 0.0038 | 3.26E-09 | 715226 | 0.989629 |
| rs1324110 | 6 | 93913200 | C | G | 0.4422 | -0.0097 | 0.0016 | 3.01E-09 | 806168 | 0.989283 |
| rs9463175 | 6 | 9510030 | T | C | 0.3293 | -0.0115 | 0.0017 | 2.24E-11 | 794719 | 0.979739 |
| rs17448885 | 6 | 97385975 | C | G | 0.6522 | 0.0105 | 0.0018 | 4.99E-09 | 718192 | 0.995527 |
| rs13209872 | 6 | 97753223 | C | G | 0.3455 | -0.0148 | 0.0018 | 2.16E-16 | 718013 | 0.992319 |
| rs9320823 | 6 | 98429337 | T | C | 0.4118 | -0.0165 | 0.0017 | 2.07E-21 | 718696 | 0.999049 |
| rs9482585 | 6 | 98773048 | A | G | 0.1285 | 0.0178 | 0.0026 | 8.51E-12 | 718619 | 0.997871 |
| rs2074686 | 7 | 100800635 | A | G | 0.5794 | -0.0111 | 0.0017 | 9.42E-11 | 718497 | 0.996955 |
| rs2299383 | 7 | 103418846 | T | C | 0.3982 | 0.0162 | 0.0016 | 6.89E-23 | 801620 | 0.987922 |
| rs1721447 | 7 | 109214139 | T | G | 0.5096 | -0.01 | 0.0017 | 3.79E-09 | 718656 | 1 |
| rs76672197 | 7 | 111360679 | T | C | 0.9481 | -0.0246 | 0.0044 | 1.77E-08 | 484680 | 0.976878 |
| rs2396625 | 7 | 113028634 | A | T | 0.4183 | -0.0176 | 0.0017 | 2.81E-24 | 718567 | 0.983597 |
| rs13245051 | 7 | 113362799 | A | G | 0.4557 | 0.015 | 0.0017 | 1.12E-18 | 714046 | 0.998728 |
| rs17371272 | 7 | 114078869 | A | G | 0.9366 | -0.0196 | 0.0035 | 2.91E-08 | 701481 | 1 |
| rs2045293 | 7 | 114350102 | T | C | 0.4158 | 0.014 | 0.0017 | 4.35E-16 | 718630 | 0.989167 |
| rs1899689 | 7 | 121964349 | T | C | 0.3939 | 0.012 | 0.0017 | 4.20E-13 | 806684 | 0.99873 |
| rs4731056 | 7 | 122031424 | T | C | 0.5233 | -0.0096 | 0.0018 | 4.90E-08 | 717113 | 0.955662 |
| rs2283093 | 7 | 126721231 | T | C | 0.1949 | 0.0121 | 0.0021 | 1.10E-08 | 718467 | 0.997641 |
| rs6950388 | 7 | 1270699 | A | G | 0.7782 | 0.0135 | 0.0022 | 1.59E-09 | 484680 | 1 |
| rs896183 | 7 | 127831580 | A | G | 0.4102 | 0.0101 | 0.0017 | 1.49E-09 | 791850 | 0.972925 |
| rs35775580 | 7 | 130420740 | A | G | 0.9543 | 0.0258 | 0.0046 | 1.62E-08 | 484680 | 1 |
| 7_130466854_A_G | 7 | 130466854 | A | G | 0.4705 | 0.0089 | 0.0016 | 4.24E-08 | 806776 | 1 |
| rs2341028 | 7 | 131603662 | A | G | 0.7005 | -0.0102 | 0.0019 | 4.55E-08 | 714722 | 0.98999 |
| rs7802342 | 7 | 137435925 | T | G | 0.7048 | -0.0124 | 0.0019 | 6.23E-11 | 712979 | 0.996972 |
| rs11525873 | 7 | 138817193 | T | C | 0.8995 | 0.0232 | 0.0032 | 2.98E-13 | 484680 | 0.991504 |
| rs62491456 | 7 | 139885277 | T | G | 0.0953 | 0.0197 | 0.0033 | 1.29E-09 | 484680 | 0.993634 |
| rs118182061 | 7 | 14655786 | A | T | 0.024 | 0.0373 | 0.0063 | 3.17E-09 | 484680 | 0.985405 |
| 7_147668180_C_T | 7 | 147668180 | T | C | 0.3396 | -0.0105 | 0.0018 | 6.39E-09 | 718218 | 0.997658 |
| rs2907948 | 7 | 150638484 | A | G | 0.244 | -0.0145 | 0.0019 | 1.95E-14 | 806659 | 0.99806 |
| rs56211164 | 7 | 158016764 | A | G | 0.2395 | -0.0128 | 0.0022 | 8.90E-09 | 484680 | 0.995892 |
| rs79826614 | 7 | 158797847 | T | C | 0.0401 | -0.0268 | 0.0049 | 3.81E-08 | 484680 | 0.987723 |
| 7_17287106_A_G | 7 | 17287106 | A | G | 0.3871 | -0.0107 | 0.0017 | 2.34E-10 | 806326 | 0.998076 |
| rs4721089 | 7 | 1872921 | T | C | 0.783 | 0.0167 | 0.0023 | 5.60E-13 | 484680 | 0.996414 |
| rs10263780 | 7 | 19778086 | A | G | 0.1343 | -0.0159 | 0.0026 | 8.00E-10 | 575101 | 0.990721 |
| rs40245 | 7 | 21470536 | A | T | 0.3374 | 0.0104 | 0.0018 | 6.54E-09 | 718125 | 0.985673 |
| rs4307239 | 7 | 24354300 | A | G | 0.5361 | -0.0115 | 0.0017 | 1.47E-11 | 717563 | 0.991771 |
| rs11770345 | 7 | 24483969 | A | G | 0.3306 | 0.01 | 0.0018 | 3.01E-08 | 705917 | 0.998806 |
| rs12666574 | 7 | 26526960 | A | G | 0.697 | 0.0101 | 0.0018 | 3.22E-08 | 718406 | 0.9907 |
| rs11971098 | 7 | 26699061 | A | G | 0.9093 | -0.0196 | 0.003 | 8.08E-11 | 718702 | 0.98831 |
| rs10228276 | 7 | 27247279 | A | G | 0.8088 | -0.0138 | 0.0022 | 2.39E-10 | 718595 | 0.995138 |
| rs849135 | 7 | 28196413 | A | G | 0.4845 | 0.0106 | 0.0016 | 4.74E-11 | 805299 | 0.999714 |
| rs4722398 | 7 | 3125220 | T | C | 0.1461 | 0.0147 | 0.0025 | 3.92E-09 | 718601 | 1 |
| rs215669 | 7 | 32378979 | A | G | 0.6024 | -0.0149 | 0.0017 | 8.94E-18 | 718120 | 0.992828 |
| rs2717926 | 7 | 37714304 | T | C | 0.6668 | 0.01 | 0.0017 | 7.76E-09 | 806770 | 0.998384 |
| rs1229057 | 7 | 39054538 | T | C | 0.1206 | 0.0155 | 0.0027 | 5.80E-09 | 714974 | 0.990131 |
| rs2237403 | 7 | 39448936 | T | C | 0.3582 | -0.0128 | 0.0018 | 1.22E-12 | 718450 | 0.999125 |
| rs10236427 | 7 | 4379718 | T | C | 0.2224 | 0.0123 | 0.0021 | 9.50E-09 | 718269 | 0.940544 |
| rs217433 | 7 | 44553496 | T | C | 0.8031 | -0.0115 | 0.0021 | 3.00E-08 | 805664 | 0.991549 |
| rs2289379 | 7 | 44804225 | T | C | 0.3917 | -0.0137 | 0.0018 | 7.02E-15 | 715836 | 0.989393 |
| rs1358005 | 7 | 46062372 | A | G | 0.1866 | 0.0136 | 0.0024 | 2.84E-08 | 484680 | 0.99559 |
| rs10269783 | 7 | 49616203 | A | G | 0.4171 | 0.0126 | 0.0017 | 2.26E-14 | 804361 | 0.995969 |
| rs10499694 | 7 | 50614173 | A | G | 0.4935 | 0.013 | 0.0016 | 1.29E-15 | 806696 | 0.995145 |
| rs6463489 | 7 | 5542513 | T | C | 0.0959 | 0.0167 | 0.0026 | 2.50E-10 | 805923 | 0.995662 |
| rs836525 | 7 | 6465909 | T | C | 0.1719 | 0.0138 | 0.0023 | 1.20E-09 | 718049 | 0.997911 |
| rs7779296 | 7 | 6779270 | A | G | 0.7159 | -0.0103 | 0.0018 | 4.59E-09 | 805088 | 1 |
| rs1035010 | 7 | 69598328 | T | C | 0.2517 | 0.0139 | 0.002 | 2.01E-12 | 664340 | 0.999241 |
| rs10237317 | 7 | 70045941 | A | G | 0.5872 | -0.0112 | 0.0017 | 1.26E-11 | 805984 | 0.987844 |
| rs12530737 | 7 | 71439807 | T | C | 0.736 | 0.0137 | 0.0019 | 1.02E-12 | 718649 | 0.995807 |
| rs860013 | 7 | 71568867 | T | C | 0.2861 | 0.0106 | 0.0019 | 2.68E-08 | 718675 | 0.998895 |
| rs56383938 | 7 | 74097622 | A | G | 0.9159 | 0.0253 | 0.0034 | 1.53E-13 | 484680 | 1 |
| 7_74101909_C_T | 7 | 74101909 | T | C | 0.772 | -0.0162 | 0.0019 | 7.45E-18 | 778483 | 0.999492 |
| rs17207196 | 7 | 75101065 | T | C | 0.42 | -0.022 | 0.0017 | 1.58E-36 | 694986 | 1 |
| rs2245368 | 7 | 76608143 | T | C | 0.8137 | -0.0238 | 0.0023 | 1.72E-25 | 690355 | 1 |
| rs17722341 | 7 | 76712801 | T | G | 0.8915 | 0.0189 | 0.0027 | 2.37E-12 | 800707 | 0.884463 |
| rs74771153 | 7 | 76806135 | A | G | 0.155 | 0.017 | 0.0026 | 1.20E-10 | 484680 | 0.9865 |
| rs6971149 | 7 | 77024300 | T | C | 0.3117 | 0.0102 | 0.0018 | 1.71E-08 | 718567 | 1 |
| rs740157 | 7 | 77055885 | A | G | 0.4501 | 0.0118 | 0.0016 | 4.76E-13 | 806777 | 0.989294 |
| rs1544459 | 7 | 77417584 | T | C | 0.5149 | -0.01 | 0.0016 | 7.08E-10 | 806731 | 0.999677 |
| rs1852006 | 7 | 77829768 | A | G | 0.376 | -0.0153 | 0.0018 | 6.71E-18 | 718382 | 1 |
| 7_78121458_C_T | 7 | 78121458 | T | C | 0.5432 | 0.0116 | 0.0017 | 8.87E-12 | 718667 | 0.986923 |
| rs274628 | 7 | 86265855 | A | C | 0.3366 | -0.0102 | 0.0018 | 1.36E-08 | 718131 | 0.999376 |
| rs2283006 | 7 | 93085722 | A | G | 0.4881 | 0.0132 | 0.0017 | 8.06E-15 | 717331 | 0.990151 |
| rs12669252 | 7 | 93234426 | A | G | 0.0892 | -0.019 | 0.003 | 2.17E-10 | 718682 | 0.997922 |
| rs13240600 | 7 | 99064466 | A | G | 0.8343 | 0.018 | 0.0022 | 7.90E-16 | 718601 | 0.99928 |
| rs2952177 | 8 | 10141443 | A | G | 0.0864 | 0.019 | 0.0034 | 2.17E-08 | 594044 | 0.992382 |
| rs3134358 | 8 | 101958433 | T | G | 0.6192 | 0.0122 | 0.0018 | 3.85E-12 | 706553 | 0.999184 |
| rs7463228 | 8 | 10506113 | T | C | 0.9012 | -0.0161 | 0.0029 | 4.57E-08 | 669208 | 1 |
| rs2252152 | 8 | 105263751 | A | T | 0.5761 | 0.0096 | 0.0017 | 2.53E-08 | 718510 | 0.997987 |
| rs1383592 | 8 | 106430676 | A | G | 0.212 | 0.0122 | 0.0021 | 4.92E-09 | 718467 | 0.995722 |
| rs56183247 | 8 | 10764512 | C | G | 0.9372 | -0.0238 | 0.004 | 1.80E-09 | 484680 | 0.980631 |
| rs4240673 | 8 | 10787612 | T | C | 0.4545 | 0.0175 | 0.0016 | 1.58E-26 | 806735 | 0.993675 |
| rs6469351 | 8 | 112353205 | T | C | 0.6719 | 0.0111 | 0.0018 | 3.72E-10 | 718614 | 0.994059 |
| rs3808434 | 8 | 116559435 | A | G | 0.412 | 0.0115 | 0.0016 | 1.91E-12 | 806715 | 0.998302 |
| rs61004096 | 8 | 11656924 | T | C | 0.1872 | -0.0146 | 0.0025 | 2.41E-09 | 484680 | 0.990951 |
| rs3808477 | 8 | 116670347 | T | C | 0.274 | -0.0182 | 0.0019 | 8.73E-22 | 718439 | 0.99668 |
| rs72673947 | 8 | 118884379 | A | G | 0.8902 | -0.0223 | 0.0031 | 4.11E-13 | 484680 | 0.98111 |
| rs6991323 | 8 | 124149944 | T | C | 0.3466 | -0.0099 | 0.0018 | 1.93E-08 | 718635 | 0.99783 |
| rs2954021 | 8 | 126482077 | A | G | 0.5089 | -0.0107 | 0.0017 | 2.41E-10 | 718633 | 0.99957 |
| rs12675063 | 8 | 132879047 | A | T | 0.87 | -0.0158 | 0.0025 | 2.70E-10 | 804382 | 0.999416 |
| rs305256 | 8 | 137568252 | T | C | 0.2263 | -0.0115 | 0.0021 | 2.79E-08 | 714665 | 0.999259 |
| rs16906845 | 8 | 138215228 | A | G | 0.06 | -0.0244 | 0.0036 | 1.32E-11 | 718216 | 1 |
| rs13263601 | 8 | 14095900 | A | C | 0.6585 | -0.0145 | 0.0018 | 4.87E-16 | 714980 | 0.998884 |
| rs11782074 | 8 | 142617096 | T | G | 0.3675 | 0.0124 | 0.0018 | 4.24E-12 | 717837 | 0.966652 |
| rs10110727 | 8 | 14324437 | A | G | 0.2591 | 0.0129 | 0.002 | 9.09E-11 | 584654 | 0.992795 |
| rs10099330 | 8 | 143383694 | A | G | 0.538 | -0.0119 | 0.0017 | 3.22E-12 | 714811 | 0.996807 |
| rs2543132 | 8 | 15536311 | C | G | 0.8087 | 0.0139 | 0.0021 | 1.02E-10 | 718603 | 0.99209 |
| 8_17002725_C_T | 8 | 17002725 | T | C | 0.8088 | 0.0141 | 0.002 | 5.65E-12 | 780990 | 0.983408 |
| rs2616192 | 8 | 20668624 | T | G | 0.6789 | 0.0128 | 0.0018 | 3.87E-12 | 718287 | 0.988029 |
| rs73225274 | 8 | 21088909 | A | G | 0.8649 | -0.0156 | 0.0028 | 2.33E-08 | 484680 | 0.99628 |
| rs11781222 | 8 | 23389571 | T | C | 0.853 | 0.0167 | 0.0024 | 3.25E-12 | 806346 | 0.987718 |
| rs73671497 | 8 | 23456634 | A | T | 0.8329 | 0.0151 | 0.0026 | 3.24E-09 | 484680 | 0.999235 |
| rs2081493 | 8 | 25651528 | C | G | 0.2997 | 0.0112 | 0.0018 | 1.30E-09 | 718699 | 0.999519 |
| rs117176448 | 8 | 27261138 | C | G | 0.9067 | -0.0213 | 0.0033 | 7.39E-11 | 484680 | 1 |
| rs3757894 | 8 | 27996440 | C | G | 0.1267 | -0.0154 | 0.0026 | 2.77E-09 | 706205 | 0.979653 |
| rs1982441 | 8 | 28021769 | T | G | 0.1322 | 0.017 | 0.0025 | 2.17E-11 | 715344 | 0.993729 |
| rs2100814 | 8 | 28118130 | A | G | 0.3992 | 0.0109 | 0.0017 | 2.89E-10 | 718614 | 0.988107 |
| rs7828890 | 8 | 28635787 | A | G | 0.0995 | -0.0152 | 0.0028 | 4.48E-08 | 794853 | 0.981636 |
| rs1421334 | 8 | 30865733 | A | C | 0.4546 | 0.0135 | 0.0017 | 3.11E-15 | 717941 | 0.985536 |
| rs7826312 | 8 | 32400115 | T | C | 0.4319 | -0.0109 | 0.0016 | 3.23E-11 | 806458 | 0.98681 |
| rs2466103 | 8 | 32412304 | T | G | 0.6928 | -0.0121 | 0.0018 | 8.00E-12 | 802631 | 0.997746 |
| rs2919389 | 8 | 32588901 | T | C | 0.414 | 0.0097 | 0.0017 | 2.95E-08 | 718102 | 0.985161 |
| rs6468188 | 8 | 33639478 | T | C | 0.4871 | 0.0093 | 0.0016 | 1.14E-08 | 805422 | 0.994093 |
| rs6468266 | 8 | 34380276 | A | T | 0.4176 | -0.0113 | 0.0017 | 7.71E-11 | 718278 | 0.995449 |
| 8_38332318_C_T | 8 | 38332318 | T | C | 0.5836 | -0.0106 | 0.0017 | 7.55E-10 | 717845 | 1 |
| rs78686130 | 8 | 4128311 | T | C | 0.3104 | -0.0119 | 0.0021 | 1.17E-08 | 484680 | 0.981473 |
| rs1658820 | 8 | 4288577 | T | G | 0.2389 | 0.0132 | 0.002 | 3.77E-11 | 704952 | 0.982671 |
| rs11784406 | 8 | 60856330 | T | C | 0.3801 | -0.0111 | 0.002 | 1.36E-08 | 484680 | 0.999364 |
| rs12681792 | 8 | 62054463 | A | C | 0.2024 | 0.015 | 0.0021 | 2.88E-12 | 717806 | 0.98891 |
| rs9298076 | 8 | 64315765 | T | C | 0.6054 | -0.01 | 0.0017 | 1.54E-09 | 806411 | 0.994811 |
| rs6472125 | 8 | 64752533 | A | G | 0.5118 | 0.0109 | 0.0017 | 1.02E-10 | 718631 | 0.998044 |
| rs10092723 | 8 | 67194710 | A | C | 0.2494 | -0.0133 | 0.002 | 1.17E-11 | 718633 | 0.985185 |
| rs12156160 | 8 | 67218772 | A | G | 0.8554 | -0.0153 | 0.0027 | 1.80E-08 | 484680 | 0.991195 |
| rs1808629 | 8 | 73435964 | A | G | 0.674 | -0.0202 | 0.002 | 2.19E-23 | 484680 | 0.994078 |
| rs2170382 | 8 | 74689288 | T | C | 0.1103 | 0.0177 | 0.0027 | 4.26E-11 | 718650 | 0.992808 |
| rs12235100 | 8 | 76591252 | A | G | 0.7761 | -0.0131 | 0.0021 | 1.66E-10 | 718687 | 0.995568 |
| rs17405819 | 8 | 76806584 | T | C | 0.6845 | 0.0211 | 0.0018 | 6.04E-33 | 806765 | 0.99979 |
| rs10093444 | 8 | 77130195 | A | G | 0.5333 | -0.0164 | 0.0017 | 4.81E-22 | 718646 | 0.997195 |
| rs16907751 | 8 | 81375457 | T | C | 0.0877 | -0.0194 | 0.0029 | 1.63E-11 | 792432 | 0.991078 |
| rs12549973 | 8 | 8176554 | T | C | 0.2821 | 0.0119 | 0.0021 | 2.06E-08 | 484680 | 1 |
| rs2196618 | 8 | 85089437 | A | G | 0.2634 | -0.0137 | 0.0019 | 1.49E-12 | 718304 | 0.993251 |
| rs7828076 | 8 | 85523598 | A | G | 0.7592 | 0.0136 | 0.002 | 8.72E-12 | 718505 | 0.995015 |
| rs77883185 | 8 | 85620323 | T | C | 0.0505 | 0.0265 | 0.0044 | 2.26E-09 | 484680 | 0.968058 |
| rs12546331 | 8 | 87505968 | T | C | 0.508 | 0.0108 | 0.0017 | 2.09E-10 | 714878 | 0.998887 |
| rs12386885 | 8 | 87766769 | T | C | 0.1564 | 0.0151 | 0.0026 | 9.90E-09 | 484680 | 0.994121 |
| rs9297524 | 8 | 89637919 | T | C | 0.6874 | 0.0113 | 0.0018 | 5.49E-10 | 718283 | 0.99266 |
| rs2077869 | 8 | 8967434 | A | T | 0.5883 | 0.0108 | 0.0018 | 6.65E-10 | 717101 | 0.97967 |
| rs1899898 | 8 | 93240419 | T | C | 0.6594 | 0.0107 | 0.0018 | 2.96E-09 | 718693 | 1 |
| rs67456872 | 8 | 9437399 | T | C | 0.9447 | 0.0263 | 0.0042 | 2.78E-10 | 484680 | 1 |
| rs12680842 | 8 | 95582606 | A | G | 0.6839 | 0.0142 | 0.0017 | 3.41E-16 | 800300 | 0.993335 |
| rs11786992 | 8 | 95685147 | A | C | 0.6311 | -0.0106 | 0.0017 | 3.65E-10 | 801618 | 0.997938 |
| rs4841280 | 8 | 9956552 | A | G | 0.7698 | -0.0158 | 0.002 | 6.40E-15 | 718082 | 0.993528 |
| rs10118866 | 9 | 10119157 | T | G | 0.2285 | 0.0113 | 0.002 | 1.99E-08 | 718496 | 0.995943 |
| rs420158 | 9 | 101477500 | T | C | 0.7619 | -0.0107 | 0.0019 | 9.41E-09 | 806778 | 1 |
| rs12551906 | 9 | 102119090 | A | G | 0.294 | -0.0117 | 0.0018 | 7.72E-11 | 805800 | 0.989428 |
| rs7025938 | 9 | 103088321 | C | G | 0.6798 | -0.0162 | 0.0018 | 1.47E-19 | 717817 | 0.999805 |
| rs7023745 | 9 | 10408553 | T | C | 0.8606 | -0.0135 | 0.0024 | 2.02E-08 | 706893 | 0.990616 |
| rs10118878 | 9 | 104395970 | A | G | 0.4821 | 0.0099 | 0.0017 | 5.80E-09 | 718603 | 0.996811 |
| rs7024334 | 9 | 109072075 | T | G | 0.2254 | 0.0135 | 0.002 | 4.71E-12 | 799813 | 0.999089 |
| rs6477694 | 9 | 111932342 | T | C | 0.6415 | -0.0126 | 0.0017 | 6.57E-14 | 806728 | 0.990418 |
| rs10817191 | 9 | 114348860 | C | G | 0.441 | 0.0099 | 0.0017 | 1.21E-08 | 717932 | 0.998657 |
| rs2482080 | 9 | 117869684 | T | G | 0.5737 | -0.0098 | 0.0017 | 1.39E-08 | 718664 | 0.997903 |
| rs970433 | 9 | 11859691 | T | G | 0.6302 | 0.0138 | 0.0018 | 5.91E-15 | 718694 | 0.998357 |
| rs9408879 | 9 | 118640119 | T | C | 0.3758 | -0.01 | 0.0018 | 1.04E-08 | 715135 | 0.994739 |
| 9_120378483_C_T | 9 | 120378483 | T | C | 0.5712 | 0.0134 | 0.0016 | 2.23E-16 | 806659 | 0.999926 |
| rs1877875 | 9 | 120664469 | T | C | 0.4341 | -0.0109 | 0.0017 | 2.51E-10 | 718589 | 0.9961 |
| rs10984756 | 9 | 122651784 | C | G | 0.9051 | -0.0176 | 0.0028 | 4.37E-10 | 718010 | 0.988454 |
| rs4308812 | 9 | 124613278 | A | G | 0.4113 | 0.0107 | 0.0018 | 7.98E-09 | 557530 | 0.995839 |
| 9_126096522_A_G | 9 | 126096522 | A | G | 0.3906 | 0.0131 | 0.0017 | 7.71E-15 | 787901 | 1 |
| rs1752156 | 9 | 126534512 | T | C | 0.7968 | -0.0128 | 0.002 | 4.17E-10 | 800350 | 0.99426 |
| 9_127060431_C_T | 9 | 127060431 | T | C | 0.4162 | 0.0112 | 0.0017 | 5.66E-11 | 718685 | 0.993385 |
| rs10819190 | 9 | 129408513 | A | G | 0.3831 | 0.0108 | 0.0018 | 1.04E-09 | 717660 | 0.985614 |
| rs10733682 | 9 | 129460914 | A | G | 0.4594 | 0.0148 | 0.0016 | 1.76E-19 | 805407 | 0.961882 |
| rs7871866 | 9 | 131027982 | C | G | 0.1596 | 0.0179 | 0.0024 | 7.56E-14 | 713754 | 0.989531 |
| rs4740383 | 9 | 133783566 | A | G | 0.4185 | 0.0131 | 0.0018 | 7.03E-14 | 694837 | 0.990892 |
| rs4601460 | 9 | 134849254 | A | G | 0.3218 | 0.0114 | 0.002 | 2.13E-08 | 484680 | 0.990876 |
| rs10858334 | 9 | 137989785 | C | G | 0.8484 | -0.0148 | 0.0025 | 4.91E-09 | 707975 | 0.978181 |
| rs13292270 | 9 | 13940536 | A | G | 0.8114 | 0.0131 | 0.0022 | 1.62E-09 | 716766 | 1 |
| rs28670671 | 9 | 140363045 | T | C | 0.7119 | 0.0122 | 0.0022 | 2.27E-08 | 484680 | 0.926374 |
| rs11792069 | 9 | 140646121 | A | G | 0.8282 | 0.014 | 0.0023 | 1.83E-09 | 672654 | 0.994993 |
| rs7031064 | 9 | 14455076 | A | G | 0.5267 | 0.011 | 0.0017 | 1.22E-10 | 718018 | 1 |
| rs11790280 | 9 | 14651283 | T | C | 0.6102 | -0.0102 | 0.0017 | 5.96E-09 | 717996 | 0.989625 |
| rs10733289 | 9 | 14775859 | A | G | 0.5776 | -0.0104 | 0.0018 | 4.44E-09 | 664295 | 0.990111 |
| rs62571017 | 9 | 15509963 | T | C | 0.9519 | 0.0336 | 0.0045 | 5.42E-14 | 484680 | 0.995593 |
| rs4740619 | 9 | 15634326 | T | C | 0.5436 | 0.0189 | 0.0016 | 3.15E-31 | 806567 | 0.998657 |
| rs1411431 | 9 | 16728721 | A | C | 0.1598 | 0.0229 | 0.0026 | 1.46E-18 | 484680 | 0.99403 |
| rs10757259 | 9 | 2198033 | A | C | 0.2477 | -0.0113 | 0.002 | 2.74E-08 | 691791 | 1 |
| rs1339139 | 9 | 23227372 | A | G | 0.6573 | 0.0123 | 0.002 | 1.47E-09 | 484680 | 0.972892 |
| rs10965780 | 9 | 23341715 | C | G | 0.4098 | -0.0107 | 0.0017 | 8.56E-10 | 717336 | 0.989997 |
| rs7874154 | 9 | 27777012 | T | C | 0.5128 | -0.0127 | 0.0017 | 6.94E-14 | 718563 | 0.990254 |
| rs1412235 | 9 | 28410996 | C | G | 0.3151 | 0.0237 | 0.0017 | 2.28E-42 | 804074 | 0.994739 |
| rs13299788 | 9 | 28941459 | C | G | 0.8492 | 0.0148 | 0.0023 | 3.00E-10 | 718666 | 0.999225 |
| rs10757826 | 9 | 29270224 | A | G | 0.3349 | 0.0102 | 0.0017 | 5.37E-09 | 794739 | 0.998255 |
| rs12238336 | 9 | 29716655 | T | C | 0.3828 | -0.0121 | 0.0017 | 3.76E-12 | 718470 | 0.994466 |
| rs16916303 | 9 | 30823761 | A | G | 0.8778 | 0.0145 | 0.0026 | 2.80E-08 | 704528 | 0.973401 |
| rs17720922 | 9 | 31030917 | T | C | 0.1875 | 0.0128 | 0.0022 | 4.18E-09 | 718607 | 0.99897 |
| rs1335126 | 9 | 31176989 | A | T | 0.2641 | 0.0111 | 0.002 | 1.53E-08 | 714975 | 0.997755 |
| rs10971721 | 9 | 33827694 | T | C | 0.1039 | -0.0201 | 0.0028 | 3.19E-13 | 714990 | 0.998811 |
| rs2281997 | 9 | 34521867 | T | C | 0.3314 | 0.0101 | 0.0018 | 3.42E-08 | 718632 | 0.985213 |
| rs6476617 | 9 | 37200103 | A | G | 0.3797 | -0.0149 | 0.0017 | 1.53E-17 | 718598 | 0.995362 |
| rs7042372 | 9 | 6959840 | A | G | 0.6671 | 0.0115 | 0.0018 | 1.47E-10 | 718414 | 0.984927 |
| rs2134858 | 9 | 73837155 | T | C | 0.5119 | -0.0117 | 0.0017 | 5.87E-12 | 718696 | 0.998694 |
| rs684639 | 9 | 79312579 | A | T | 0.4123 | -0.0098 | 0.0017 | 1.39E-08 | 718462 | 0.992544 |
| rs10869977 | 9 | 80457276 | A | G | 0.448 | -0.0106 | 0.0017 | 4.16E-10 | 718266 | 0.989929 |
| rs1634350 | 9 | 81334684 | A | C | 0.4355 | 0.0114 | 0.0017 | 3.19E-11 | 718495 | 0.982598 |
| rs72738288 | 9 | 81382438 | T | C | 0.0397 | -0.0285 | 0.0051 | 1.88E-08 | 484680 | 0.926343 |
| rs11138313 | 9 | 82240968 | A | G | 0.8966 | 0.0176 | 0.0028 | 6.02E-10 | 706567 | 1 |
| rs2777768 | 9 | 84186734 | A | G | 0.7228 | 0.0119 | 0.0019 | 6.38E-10 | 718589 | 0.993176 |
| 9_85135915_A_C | 9 | 85135915 | A | C | 0.6611 | 0.0097 | 0.0017 | 1.65E-08 | 802925 | 1 |
| rs1187352 | 9 | 87293457 | T | C | 0.3426 | -0.012 | 0.0018 | 2.61E-11 | 718219 | 0.995046 |
| rs12380880 | 9 | 88912765 | T | C | 0.2255 | -0.0114 | 0.002 | 1.94E-08 | 718470 | 0.996612 |
| rs4360376 | 9 | 92074429 | A | C | 0.7743 | -0.0128 | 0.002 | 4.40E-10 | 718539 | 0.978541 |
| rs7357754 | 9 | 92207308 | A | G | 0.4985 | -0.012 | 0.0017 | 1.81E-12 | 718167 | 0.994155 |
| rs3811125 | 9 | 94187247 | T | C | 0.2806 | -0.0152 | 0.0021 | 9.42E-13 | 484680 | 0.991864 |
| rs7018588 | 9 | 96382449 | A | G | 0.2448 | 0.0129 | 0.0021 | 9.64E-10 | 703629 | 0.916744 |
| rs10992867 | 9 | 96461013 | A | G | 0.2687 | 0.0162 | 0.0019 | 3.20E-17 | 718610 | 0.99645 |
| rs117440602 | 9 | 96582776 | A | G | 0.0361 | 0.0341 | 0.0052 | 4.80E-11 | 484680 | 0.971451 |
| 9_98410405_C_T | 9 | 98410405 | T | C | 0.2318 | -0.0112 | 0.002 | 1.25E-08 | 806728 | 0.953852 |
| rs1547205 | 9 | 98815145 | C | G | 0.0846 | -0.0168 | 0.0031 | 4.38E-08 | 576834 | 0.969506 |
| rs7090035 | 10 | 100026791 | C | G | 0.4334 | -0.0122 | 0.0017 | 1.07E-12 | 718519 | 0.9895 |
| rs17094222 | 10 | 102395440 | T | C | 0.7912 | -0.0173 | 0.002 | 4.04E-18 | 806450 | 0.991452 |
| rs41310284 | 10 | 102447647 | A | C | 0.097 | -0.029 | 0.0032 | 3.98E-19 | 484680 | 0.982738 |
| rs36029912 | 10 | 102465253 | T | C | 0.9505 | -0.0247 | 0.0044 | 2.26E-08 | 484680 | 1 |
| rs7916385 | 10 | 102470432 | T | C | 0.1493 | -0.0215 | 0.0029 | 1.81E-13 | 484680 | 0.840944 |
| rs61873510 | 10 | 102626510 | T | G | 0.3211 | 0.0171 | 0.0021 | 2.70E-16 | 484680 | 0.950578 |
| rs6602411 | 10 | 10264200 | T | C | 0.1755 | 0.0147 | 0.0022 | 5.98E-11 | 715658 | 0.997688 |
| rs9787495 | 10 | 103206115 | A | G | 0.425 | -0.0094 | 0.0017 | 3.81E-08 | 718476 | 0.996499 |
| 10_103984060_C_T | 10 | 103984060 | T | C | 0.8309 | 0.0165 | 0.0022 | 1.56E-13 | 806791 | 1 |
| rs3977755 | 10 | 104420210 | T | C | 0.2871 | -0.0132 | 0.0018 | 8.90E-13 | 752370 | 0.999634 |
| 10_104719096_A_G | 10 | 104719096 | A | G | 0.074 | 0.0267 | 0.0029 | 4.73E-20 | 806514 | 1 |
| rs79780963 | 10 | 104952499 | T | C | 0.0804 | 0.0244 | 0.0035 | 2.63E-12 | 484680 | 0.997938 |
| 10_114758349_C_T | 10 | 114758349 | T | C | 0.2787 | -0.0178 | 0.0018 | 1.67E-23 | 806810 | 1 |
| rs10886017 | 10 | 118672531 | A | C | 0.2413 | 0.0146 | 0.0019 | 4.47E-15 | 806301 | 0.982787 |
| rs73423977 | 10 | 120495772 | T | C | 0.8995 | -0.0174 | 0.0032 | 3.96E-08 | 484680 | 0.996786 |
| rs4523610 | 10 | 122069448 | T | C | 0.7224 | -0.0108 | 0.002 | 3.16E-08 | 718611 | 0.988448 |
| rs845084 | 10 | 125220036 | A | G | 0.2666 | 0.0136 | 0.0019 | 3.22E-12 | 718056 | 0.98939 |
| rs17636031 | 10 | 126594078 | T | C | 0.7244 | -0.0154 | 0.0018 | 3.87E-17 | 798189 | 0.994828 |
| rs75011412 | 10 | 126616820 | T | C | 0.0512 | -0.0265 | 0.0045 | 3.02E-09 | 484680 | 0.936767 |
| rs7358274 | 10 | 128464019 | T | C | 0.2931 | -0.0117 | 0.0021 | 4.51E-08 | 484680 | 0.96463 |
| rs2542615 | 10 | 131128952 | T | C | 0.6611 | -0.0112 | 0.0018 | 5.21E-10 | 718683 | 0.973692 |
| rs4880341 | 10 | 133992689 | T | C | 0.5725 | -0.013 | 0.0017 | 3.06E-14 | 716038 | 0.998582 |
| rs7893571 | 10 | 16750129 | T | G | 0.6736 | 0.0125 | 0.0018 | 5.83E-12 | 718186 | 0.993715 |
| rs1277733 | 10 | 18562538 | T | C | 0.7664 | 0.012 | 0.002 | 4.30E-09 | 706870 | 1 |
| rs12776880 | 10 | 19776828 | A | T | 0.7208 | 0.0109 | 0.0019 | 8.59E-09 | 706790 | 0.994242 |
| rs76638898 | 10 | 21099584 | A | G | 0.0234 | -0.0376 | 0.0065 | 8.15E-09 | 484680 | 0.938754 |
| rs7084454 | 10 | 21821274 | A | G | 0.3086 | 0.0198 | 0.0018 | 4.51E-27 | 706792 | 0.995785 |
| rs11251352 | 10 | 2585792 | A | G | 0.4084 | -0.0096 | 0.0017 | 2.64E-08 | 718650 | 0.996342 |
| rs10829164 | 10 | 27318370 | T | C | 0.1468 | 0.0151 | 0.0024 | 2.37E-10 | 718703 | 0.999746 |
| rs4097319 | 10 | 33860515 | T | G | 0.5641 | 0.0107 | 0.0017 | 4.32E-10 | 718651 | 0.99425 |
| rs117369655 | 10 | 33985434 | T | C | 0.0965 | -0.0184 | 0.0033 | 1.82E-08 | 484680 | 0.971486 |
| rs12765914 | 10 | 34013507 | T | C | 0.0801 | 0.0226 | 0.0031 | 1.96E-13 | 718619 | 0.999338 |
| rs10827289 | 10 | 34091492 | T | C | 0.5799 | -0.0106 | 0.0017 | 5.85E-10 | 718592 | 0.996649 |
| rs1624134 | 10 | 34834482 | C | G | 0.3925 | 0.01 | 0.0017 | 6.51E-09 | 717695 | 0.997576 |
| rs10763587 | 10 | 52356264 | T | C | 0.3167 | 0.0111 | 0.0019 | 2.88E-09 | 717903 | 0.967439 |
| rs12259464 | 10 | 53680099 | A | G | 0.4811 | 0.0109 | 0.0017 | 1.48E-10 | 716686 | 0.992198 |
| rs7070670 | 10 | 61842645 | T | C | 0.3228 | -0.0126 | 0.0021 | 7.14E-10 | 484680 | 0.982719 |
| rs1993414 | 10 | 63403235 | T | G | 0.9402 | 0.0189 | 0.0034 | 1.73E-08 | 794602 | 0.993037 |
| rs12247165 | 10 | 64815153 | A | G | 0.1086 | -0.0151 | 0.0027 | 1.44E-08 | 746263 | 0.990968 |
| rs10761785 | 10 | 65318766 | T | G | 0.5136 | -0.0133 | 0.0016 | 3.47E-16 | 806365 | 0.996172 |
| rs2933451 | 10 | 66626611 | A | C | 0.669 | -0.0129 | 0.002 | 1.98E-10 | 484680 | 0.990951 |
| rs12098284 | 10 | 76047464 | T | C | 0.1212 | 0.0184 | 0.0026 | 9.87E-13 | 714745 | 0.998313 |
| rs7075347 | 10 | 76533307 | T | C | 0.0652 | -0.0226 | 0.0035 | 1.42E-10 | 693850 | 1 |
| rs11001221 | 10 | 76716136 | A | G | 0.9091 | -0.0213 | 0.0033 | 1.07E-10 | 484680 | 0.984619 |
| rs4746339 | 10 | 77656509 | A | T | 0.2601 | -0.0131 | 0.0019 | 1.18E-11 | 718627 | 0.998955 |
| rs11001963 | 10 | 78760959 | T | C | 0.55 | 0.0112 | 0.0017 | 8.54E-11 | 718140 | 0.978215 |
| rs1249156 | 10 | 79979737 | T | C | 0.2872 | -0.0102 | 0.0019 | 4.54E-08 | 718677 | 0.998907 |
| rs7899106 | 10 | 87410904 | A | G | 0.9496 | -0.0327 | 0.0037 | 1.72E-18 | 806450 | 0.986531 |
| rs10788494 | 10 | 88110925 | C | G | 0.4835 | 0.0132 | 0.0017 | 5.51E-15 | 718328 | 0.993709 |
| rs2450448 | 10 | 93062111 | A | T | 0.6874 | 0.0128 | 0.0021 | 3.93E-10 | 484680 | 0.998738 |
| rs35808802 | 10 | 97143826 | T | C | 0.9405 | 0.0221 | 0.004 | 3.91E-08 | 484680 | 1 |
| 10_98975893_A_C | 10 | 98975893 | A | C | 0.6176 | -0.011 | 0.0019 | 6.12E-09 | 713913 | 0.990306 |
| rs2439823 | 10 | 99778226 | A | G | 0.4514 | -0.0165 | 0.0017 | 6.51E-22 | 718034 | 0.991052 |
| rs2513999 | 11 | 103019633 | A | G | 0.1593 | -0.0154 | 0.0026 | 3.07E-09 | 484680 | 0.995643 |
| rs1400759 | 11 | 110965115 | A | C | 0.6487 | -0.0101 | 0.0018 | 2.27E-08 | 717412 | 0.994985 |
| 11_112912947_C_T | 11 | 112912947 | T | C | 0.259 | -0.0114 | 0.0019 | 8.17E-10 | 806769 | 0.990184 |
| rs719802 | 11 | 113234679 | T | C | 0.3974 | 0.0106 | 0.0017 | 9.79E-10 | 718636 | 1 |
| rs513249 | 11 | 114032592 | A | T | 0.2501 | 0.0122 | 0.0022 | 2.57E-08 | 484680 | 0.984187 |
| 11_115022404_A_G | 11 | 115022404 | A | G | 0.5007 | -0.0177 | 0.0016 | 1.93E-27 | 806583 | 0.995768 |
| rs76942203 | 11 | 116973247 | A | G | 0.0587 | 0.0263 | 0.0041 | 9.08E-11 | 484680 | 0.997444 |
| 11_11796897_G_T | 11 | 11796897 | T | G | 0.5407 | -0.0098 | 0.0017 | 9.13E-09 | 718480 | 0.99169 |
| rs1064939 | 11 | 118396331 | A | T | 0.9783 | 0.0466 | 0.0066 | 1.88E-12 | 484680 | 0.976745 |
| rs3825061 | 11 | 118944675 | T | C | 0.3863 | 0.014 | 0.0017 | 6.15E-16 | 718354 | 0.999277 |
| rs3809046 | 11 | 119018766 | T | G | 0.0825 | -0.0182 | 0.0032 | 9.17E-09 | 718404 | 0.997202 |
| rs693701 | 11 | 119804461 | T | C | 0.2373 | 0.0108 | 0.0019 | 1.04E-08 | 806706 | 0.996201 |
| rs11218510 | 11 | 121922587 | A | G | 0.3949 | -0.014 | 0.002 | 6.79E-13 | 484680 | 0.998072 |
| rs564649 | 11 | 122012238 | C | G | 0.7155 | -0.0111 | 0.0019 | 5.05E-09 | 718584 | 0.992279 |
| rs10892873 | 11 | 122535333 | C | G | 0.3614 | 0.0118 | 0.0018 | 1.75E-11 | 718243 | 0.992533 |
| rs7950748 | 11 | 122762432 | A | T | 0.7406 | -0.0108 | 0.002 | 3.53E-08 | 718616 | 0.982386 |
| rs10790809 | 11 | 126372550 | A | G | 0.4369 | -0.0113 | 0.0019 | 3.19E-09 | 484680 | 1 |
| rs7944782 | 11 | 130795698 | T | G | 0.5016 | -0.0144 | 0.0017 | 3.61E-17 | 718230 | 0.988343 |
| rs12788343 | 11 | 131452912 | T | C | 0.5983 | -0.0147 | 0.0019 | 3.42E-14 | 484680 | 1 |
| rs12222235 | 11 | 131984330 | T | C | 0.3305 | -0.0128 | 0.0018 | 1.78E-12 | 718613 | 0.990705 |
| rs2007518 | 11 | 132639606 | A | G | 0.5618 | -0.013 | 0.0017 | 1.96E-14 | 718682 | 0.997106 |
| rs900144 | 11 | 13294268 | T | C | 0.5784 | 0.0148 | 0.0017 | 1.53E-18 | 718416 | 0.999577 |
| rs10894670 | 11 | 133221987 | A | C | 0.559 | -0.0096 | 0.0016 | 3.64E-09 | 805154 | 0.990867 |
| rs4554901 | 11 | 133400279 | T | C | 0.1442 | -0.0143 | 0.0025 | 1.01E-08 | 718125 | 0.980177 |
| rs10750547 | 11 | 133729249 | T | G | 0.8902 | -0.0157 | 0.0027 | 9.64E-09 | 701833 | 0.962479 |
| rs329651 | 11 | 133767622 | T | G | 0.806 | 0.016 | 0.0021 | 2.13E-14 | 799668 | 0.985862 |
| rs12806052 | 11 | 134518868 | T | C | 0.1576 | -0.0171 | 0.0026 | 5.39E-11 | 484680 | 0.994822 |
| rs12364470 | 11 | 134601012 | T | G | 0.8535 | -0.0187 | 0.0022 | 2.18E-17 | 799515 | 1 |
| rs6578412 | 11 | 1482582 | T | C | 0.0552 | 0.024 | 0.0042 | 1.14E-08 | 484680 | 0.981892 |
| rs11023948 | 11 | 16481965 | T | C | 0.7976 | -0.0121 | 0.002 | 8.41E-10 | 806624 | 0.992127 |
| rs1557765 | 11 | 17403639 | T | C | 0.3889 | -0.0122 | 0.0017 | 2.61E-13 | 800622 | 0.997384 |
| rs8176786 | 11 | 20959394 | T | C | 0.056 | 0.0217 | 0.0039 | 3.47E-08 | 718244 | 1 |
| rs10840606 | 11 | 2234690 | A | G | 0.817 | -0.0146 | 0.0023 | 2.21E-10 | 681468 | 0.972359 |
| 11_27406844_A_G | 11 | 27406844 | A | G | 0.2957 | -0.0119 | 0.0018 | 1.59E-11 | 806713 | 0.994023 |
| rs10835197 | 11 | 27607543 | T | C | 0.2317 | 0.0274 | 0.0019 | 3.96E-46 | 806794 | 0.998639 |
| 11_27679916_C_T | 11 | 27679916 | T | C | 0.1836 | -0.0413 | 0.0021 | 7.40E-89 | 806782 | 1 |
| rs908867 | 11 | 27745764 | T | C | 0.1053 | 0.0191 | 0.0027 | 1.12E-12 | 806697 | 0.996971 |
| rs2049048 | 11 | 27750586 | A | G | 0.1466 | 0.0202 | 0.0029 | 2.20E-12 | 644275 | 0.790643 |
| rs570463 | 11 | 28739318 | A | C | 0.3266 | -0.0121 | 0.0018 | 4.33E-11 | 718580 | 0.995396 |
| rs4923597 | 11 | 29250910 | A | G | 0.4107 | -0.0115 | 0.0017 | 3.57E-11 | 718174 | 0.998502 |
| 11_30422068_G_T | 11 | 30422068 | T | G | 0.646 | 0.0139 | 0.0018 | 6.07E-15 | 718408 | 0.997725 |
| rs4367923 | 11 | 30500810 | A | G | 0.3167 | 0.0116 | 0.002 | 1.48E-08 | 484680 | 0.997387 |
| rs223058 | 11 | 32125855 | A | G | 0.3349 | -0.0114 | 0.0018 | 3.29E-10 | 718638 | 0.998835 |
| rs11246136 | 11 | 371265 | A | C | 0.1021 | -0.0167 | 0.003 | 2.03E-08 | 655523 | 0.998553 |
| rs4755713 | 11 | 43532201 | T | C | 0.6032 | -0.0141 | 0.0019 | 3.08E-13 | 484680 | 0.995054 |
| rs2862996 | 11 | 43653833 | T | G | 0.7012 | -0.0216 | 0.0017 | 3.60E-35 | 806675 | 1 |
| rs2434467 | 11 | 43899601 | T | C | 0.342 | 0.012 | 0.0018 | 2.02E-11 | 718495 | 0.993532 |
| rs10742752 | 11 | 45438374 | T | C | 0.3761 | -0.0123 | 0.0017 | 1.20E-13 | 806700 | 0.995667 |
| rs118081010 | 11 | 46174948 | T | C | 0.0165 | 0.0518 | 0.0075 | 5.28E-12 | 484680 | 0.983418 |
| rs4319473 | 11 | 46702691 | A | G | 0.1469 | 0.0139 | 0.0022 | 6.26E-10 | 806688 | 0.998613 |
| rs2306028 | 11 | 46900659 | T | G | 0.1105 | -0.013 | 0.0023 | 3.22E-08 | 806679 | 0.999372 |
| rs11570115 | 11 | 47354905 | T | C | 0.8961 | 0.0181 | 0.0027 | 1.52E-11 | 802877 | 0.960717 |
| 11_47529947_A_C | 11 | 47529947 | A | C | 0.4162 | 0.0257 | 0.0016 | 3.96E-55 | 806656 | 1 |
| rs113408284 | 11 | 48160306 | T | C | 0.0236 | 0.0391 | 0.0067 | 6.26E-09 | 484680 | 0.853031 |
| rs6485981 | 11 | 49477266 | T | C | 0.0812 | 0.0222 | 0.0035 | 1.71E-10 | 484680 | 0.986357 |
| rs6591407 | 11 | 56914157 | A | C | 0.1984 | -0.0124 | 0.0021 | 3.58E-09 | 806782 | 0.995198 |
| rs198448 | 11 | 61500487 | T | C | 0.401 | -0.01 | 0.0017 | 9.14E-09 | 718667 | 0.995338 |
| 11_6239344_A_G | 11 | 6239344 | A | G | 0.5635 | -0.0103 | 0.0018 | 4.74E-09 | 698216 | 1 |
| rs530399 | 11 | 63822940 | T | G | 0.1853 | -0.0138 | 0.0022 | 3.89E-10 | 693392 | 0.994452 |
| rs7947143 | 11 | 64090422 | A | G | 0.1541 | -0.0175 | 0.0023 | 8.13E-14 | 718300 | 0.996137 |
| rs893006 | 11 | 64365796 | A | C | 0.7211 | 0.0145 | 0.0018 | 2.83E-16 | 804345 | 0.99448 |
| rs10896012 | 11 | 65278461 | T | C | 0.7849 | -0.0162 | 0.0021 | 1.02E-14 | 718182 | 0.99646 |
| 11_65594820_C_T | 11 | 65594820 | T | C | 0.6437 | -0.0168 | 0.0018 | 3.84E-21 | 718609 | 0.997773 |
| rs4073582 | 11 | 66050712 | A | G | 0.3492 | -0.014 | 0.002 | 1.86E-12 | 484680 | 0.997969 |
| rs497261 | 11 | 68192244 | T | C | 0.6732 | 0.0113 | 0.002 | 2.47E-08 | 484680 | 0.999423 |
| rs10792006 | 11 | 68722306 | T | C | 0.4527 | 0.0123 | 0.0019 | 1.35E-10 | 484680 | 0.994847 |
| rs592483 | 11 | 69445173 | T | C | 0.5933 | -0.0137 | 0.0017 | 1.53E-16 | 802791 | 0.990703 |
| rs7123876 | 11 | 72444583 | T | C | 0.7494 | -0.0118 | 0.0019 | 2.92E-10 | 806649 | 0.996333 |
| rs12282785 | 11 | 76476030 | A | C | 0.2204 | -0.0157 | 0.0023 | 1.43E-11 | 484680 | 0.972667 |
| rs1318241 | 11 | 77930792 | T | C | 0.147 | -0.0123 | 0.0022 | 2.56E-08 | 806734 | 0.997607 |
| rs349088 | 11 | 84814393 | A | C | 0.4777 | -0.013 | 0.0017 | 3.50E-14 | 718429 | 0.990655 |
| 11_85277870_A_C | 11 | 85277870 | A | C | 0.1296 | -0.0147 | 0.0025 | 2.66E-09 | 718633 | 0.992392 |
| rs4256980 | 11 | 8673939 | C | G | 0.3305 | -0.0187 | 0.0017 | 8.63E-29 | 804708 | 0.996063 |
| rs78378788 | 11 | 89075754 | A | G | 0.9325 | -0.0211 | 0.0038 | 3.81E-08 | 484680 | 0.991118 |
| rs12421848 | 11 | 891338 | A | G | 0.3962 | -0.0141 | 0.0019 | 3.99E-13 | 484680 | 0.996989 |
| rs61903695 | 11 | 89922417 | A | G | 0.751 | -0.0145 | 0.0022 | 4.54E-11 | 484680 | 0.998989 |
| rs3019466 | 11 | 92476178 | T | C | 0.1677 | -0.0128 | 0.0024 | 4.91E-08 | 718585 | 0.993304 |
| rs2605603 | 11 | 93221105 | A | G | 0.4812 | -0.0103 | 0.0016 | 2.04E-10 | 806679 | 0.994161 |
| 12 | 12 | 103706754 | C | G | 0.7468 | 0.0194 | 0.0019 | 1.14E-23 | 718487 | 0.99276 |
| 12 | 12 | 107713511 | C | G | 0.497 | 0.0109 | 0.0019 | 1.43E-08 | 484680 | 0.981732 |
| 12 | 12 | 108393845 | A | G | 0.2392 | -0.0174 | 0.002 | 1.34E-17 | 717209 | 0.996072 |
| 12_108618630_C_T | 12 | 108618630 | T | C | 0.2637 | -0.0105 | 0.0019 | 2.53E-08 | 757551 | 1 |
| 12_109883374_C_T | 12 | 109883374 | T | C | 0.358 | -0.0102 | 0.0017 | 1.87E-09 | 806785 | 1 |
| 12 | 12 | 110046698 | T | C | 0.0766 | 0.0179 | 0.0031 | 4.85E-09 | 804090 | 0.99508 |
| 12 | 12 | 110903380 | C | G | 0.6969 | -0.0156 | 0.0017 | 3.57E-19 | 806671 | 0.999312 |
| 12 | 12 | 112610714 | A | G | 0.3762 | -0.0114 | 0.0016 | 3.05E-12 | 805492 | 0.996935 |
| 12 | 12 | 114065412 | A | C | 0.2989 | 0.0116 | 0.0019 | 4.55E-10 | 718685 | 0.999317 |
| 12 | 12 | 114432613 | T | C | 0.0707 | -0.0224 | 0.0034 | 2.30E-11 | 718408 | 0.996213 |
| 12 | 12 | 116097056 | A | G | 0.3626 | 0.0101 | 0.0018 | 9.97E-09 | 718405 | 0.992855 |
| 12 | 12 | 116957607 | T | G | 0.5321 | -0.0093 | 0.0017 | 4.89E-08 | 718328 | 0.99097 |
| 12 | 12 | 117596788 | A | G | 0.4388 | 0.0108 | 0.0017 | 2.65E-10 | 718626 | 0.998977 |
| 12 | 12 | 118414697 | A | G | 0.6844 | 0.0116 | 0.0018 | 2.43E-10 | 718650 | 1 |
| 12 | 12 | 121671261 | A | C | 0.1314 | 0.0177 | 0.0026 | 7.26E-12 | 718518 | 0.997194 |
| 12 | 12 | 122011598 | T | C | 0.96 | 0.0328 | 0.0053 | 7.49E-10 | 484680 | 0.830846 |
| 12 | 12 | 122479308 | A | G | 0.2722 | -0.0128 | 0.0021 | 2.57E-09 | 484680 | 0.991575 |
| 12 | 12 | 122613540 | T | C | 0.5366 | -0.0121 | 0.0016 | 1.06E-13 | 806206 | 0.999544 |
| 12 | 12 | 122963550 | T | C | 0.085 | -0.034 | 0.0031 | 2.32E-28 | 703003 | 0.990568 |
| 12 | 12 | 123063467 | A | G | 0.3519 | 0.0114 | 0.0018 | 9.64E-11 | 718674 | 0.99847 |
| 12 | 12 | 123303586 | A | G | 0.5628 | 0.0123 | 0.0018 | 5.85E-12 | 695707 | 0.985911 |
| 12 | 12 | 123936900 | A | G | 0.591 | 0.011 | 0.002 | 4.07E-08 | 484680 | 0.925382 |
| 12 | 12 | 124267638 | A | G | 0.108 | -0.016 | 0.0026 | 1.28E-09 | 794705 | 0.971054 |
| 12 | 12 | 124409502 | A | G | 0.3335 | 0.0127 | 0.0017 | 2.93E-13 | 802968 | 0.994874 |
| 12 | 12 | 124506631 | T | C | 0.5849 | -0.0113 | 0.0017 | 9.36E-12 | 806663 | 0.993402 |
| 12 | 12 | 132439381 | T | C | 0.8813 | -0.0142 | 0.0026 | 4.04E-08 | 717986 | 0.992022 |
| 12 | 12 | 132688115 | T | C | 0.1261 | 0.0145 | 0.0026 | 2.00E-08 | 678601 | 0.973268 |
| 12 | 12 | 133426483 | T | C | 0.7066 | -0.0117 | 0.0018 | 1.87E-10 | 718698 | 0.999316 |
| 12 | 12 | 14413931 | C | G | 0.2689 | -0.0131 | 0.0019 | 1.78E-11 | 718012 | 0.989646 |
| 12 | 12 | 16470918 | T | C | 0.1614 | 0.0136 | 0.0024 | 9.35E-09 | 718674 | 0.993131 |
| 12 | 12 | 17212881 | A | G | 0.5355 | -0.0113 | 0.0017 | 2.54E-11 | 718403 | 0.99577 |
| 12 | 12 | 18090659 | T | G | 0.9698 | -0.0286 | 0.0049 | 6.40E-09 | 712102 | 0.986125 |
| 12 | 12 | 18789007 | A | C | 0.4543 | -0.0103 | 0.0017 | 2.00E-09 | 718461 | 0.99422 |
| 12 | 12 | 19157396 | A | G | 0.7961 | -0.0126 | 0.0022 | 6.80E-09 | 718160 | 0.992146 |
| 12 | 12 | 19287416 | A | T | 0.844 | 0.0161 | 0.0023 | 3.77E-12 | 718664 | 0.982611 |
| 12 | 12 | 2159556 | A | G | 0.6479 | -0.0122 | 0.002 | 7.31E-10 | 484680 | 0.990602 |
| 12 | 12 | 23559163 | A | G | 0.2095 | -0.0119 | 0.0021 | 2.17E-08 | 718381 | 0.997795 |
| 12 | 12 | 23753927 | T | G | 0.94 | -0.0247 | 0.0041 | 1.44E-09 | 484680 | 0.968386 |
| 12 | 12 | 24060075 | C | G | 0.1256 | 0.0195 | 0.0025 | 1.21E-14 | 718478 | 0.996424 |
| 12 | 12 | 33379440 | C | G | 0.8709 | -0.0147 | 0.0023 | 1.46E-10 | 803427 | 0.999792 |
| 12 | 12 | 39430048 | A | C | 0.7826 | 0.013 | 0.0019 | 1.12E-11 | 806636 | 0.999517 |
| 12 | 12 | 39996693 | A | T | 0.9808 | -0.0379 | 0.0069 | 4.44E-08 | 484680 | 0.998832 |
| 12 | 12 | 41948196 | A | G | 0.4806 | 0.0136 | 0.0016 | 1.17E-16 | 805907 | 0.97536 |
| 12 | 12 | 42090026 | A | T | 0.5339 | -0.0104 | 0.0019 | 4.40E-08 | 484680 | 0.999105 |
| 12_47471439_A_G | 12 | 47471439 | A | G | 0.3114 | -0.0097 | 0.0018 | 3.98E-08 | 806232 | 0.980689 |
| 12 | 12 | 49399132 | C | G | 0.0339 | 0.0341 | 0.0053 | 1.00E-10 | 484680 | 1 |
| 12 | 12 | 49413486 | T | C | 0.0469 | -0.0249 | 0.0043 | 9.02E-09 | 787479 | 1 |
| 12 | 12 | 49474605 | T | C | 0.3903 | 0.0108 | 0.0017 | 3.91E-10 | 718164 | 0.996468 |
| 12_50247468_A_G | 12 | 50247468 | A | G | 0.387 | 0.0297 | 0.0017 | 3.10E-71 | 806772 | 1 |
| 12 | 12 | 50280716 | A | G | 0.1162 | -0.0149 | 0.0026 | 1.40E-08 | 806371 | 0.978999 |
| 12 | 12 | 50284583 | A | T | 0.9035 | 0.0199 | 0.0035 | 1.09E-08 | 484680 | 0.859516 |
| 12 | 12 | 51593616 | T | G | 0.2088 | 0.012 | 0.0022 | 3.10E-08 | 654762 | 0.981884 |
| 12 | 12 | 53785861 | A | G | 0.1019 | 0.0168 | 0.0028 | 2.59E-09 | 718289 | 0.997473 |
| 12 | 12 | 54667285 | T | C | 0.5794 | 0.0118 | 0.0016 | 4.49E-13 | 806786 | 0.997526 |
| 12 | 12 | 56494991 | A | G | 0.396 | -0.0141 | 0.0018 | 9.26E-16 | 706022 | 0.994166 |
| 12 | 12 | 57611285 | A | G | 0.9231 | -0.0222 | 0.0036 | 8.70E-10 | 484680 | 0.972163 |
| 12 | 12 | 57938565 | A | G | 0.6018 | 0.0098 | 0.0017 | 5.30E-09 | 805442 | 1 |
| 12 | 12 | 60964108 | A | G | 0.7712 | -0.0137 | 0.0021 | 3.86E-11 | 718606 | 0.996705 |
| 12 | 12 | 68205604 | A | G | 0.1694 | 0.0142 | 0.0021 | 2.13E-11 | 806479 | 0.993684 |
| 12 | 12 | 69674595 | T | C | 0.7294 | -0.0137 | 0.0019 | 6.43E-13 | 714521 | 0.99618 |
| 12_72179446_C_T | 12 | 72179446 | T | C | 0.0189 | 0.0451 | 0.007 | 1.20E-10 | 484680 | 1 |
| 12 | 12 | 82465797 | T | C | 0.7839 | 0.0131 | 0.0019 | 6.79E-12 | 805743 | 0.994206 |
| 12 | 12 | 84283859 | T | G | 0.6521 | 0.0109 | 0.002 | 4.57E-08 | 484680 | 1 |
| 12 | 12 | 89748240 | C | G | 0.0332 | 0.0331 | 0.0055 | 2.08E-09 | 484680 | 0.923927 |
| 12 | 12 | 89757937 | A | G | 0.5521 | -0.0142 | 0.0017 | 4.69E-17 | 717231 | 0.997118 |
| 12 | 12 | 90215064 | A | G | 0.7579 | 0.0158 | 0.0022 | 1.28E-12 | 484680 | 0.997088 |
| 12 | 12 | 90628230 | T | C | 0.7272 | -0.0131 | 0.002 | 1.99E-11 | 718394 | 0.989331 |
| 12 | 12 | 91237920 | A | T | 0.3729 | -0.0113 | 0.0017 | 1.25E-11 | 801313 | 0.991053 |
| 12 | 12 | 939480 | T | G | 0.199 | 0.0223 | 0.002 | 2.04E-28 | 793063 | 0.995896 |
| 12 | 12 | 97584357 | T | C | 0.1885 | 0.0146 | 0.0024 | 2.47E-09 | 484680 | 0.991437 |
| 12 | 12 | 97913785 | T | C | 0.8217 | -0.0133 | 0.0022 | 3.33E-09 | 718395 | 0.988339 |
| 12_98016881_A_G | 12 | 98016881 | A | G | 0.6091 | -0.0102 | 0.0017 | 5.32E-09 | 718586 | 0.986144 |
| 12 | 12 | 99560183 | A | G | 0.3634 | 0.0146 | 0.0018 | 2.38E-16 | 713780 | 0.991841 |
| rs9514131 | 13 | 104090848 | T | G | 0.1289 | -0.0155 | 0.0026 | 2.92E-09 | 710946 | 1 |
| rs4349008 | 13 | 105803056 | A | G | 0.0623 | 0.0204 | 0.0037 | 3.04E-08 | 557503 | 1 |
| rs9888533 | 13 | 107854612 | T | C | 0.5302 | 0.0125 | 0.0019 | 1.38E-10 | 484680 | 0.962994 |
| rs9559022 | 13 | 108013654 | A | G | 0.8156 | -0.0121 | 0.0022 | 4.86E-08 | 718692 | 0.987646 |
| rs9522177 | 13 | 111952240 | T | C | 0.578 | 0.0101 | 0.0017 | 3.10E-09 | 718690 | 0.99665 |
| rs2479958 | 13 | 111984244 | A | G | 0.4852 | 0.0129 | 0.0018 | 1.79E-13 | 711951 | 0.975215 |
| rs12868881 | 13 | 112218924 | A | T | 0.4066 | 0.0138 | 0.0017 | 1.40E-15 | 718650 | 0.997368 |
| rs9579775 | 13 | 20616557 | A | C | 0.8669 | -0.02 | 0.0029 | 9.23E-12 | 484680 | 0.921363 |
| rs9512646 | 13 | 27930948 | T | C | 0.7779 | 0.0135 | 0.002 | 2.52E-11 | 718586 | 0.99341 |
| rs1967772 | 13 | 28036062 | A | G | 0.2719 | -0.0173 | 0.0019 | 9.40E-20 | 717929 | 0.985925 |
| rs1006353 | 13 | 28047269 | A | G | 0.2609 | 0.0118 | 0.0018 | 1.49E-10 | 806780 | 0.991753 |
| 13_28624294_A_G | 13 | 28624294 | A | G | 0.6214 | -0.0143 | 0.0017 | 2.36E-16 | 718678 | 1 |
| rs12872889 | 13 | 28674628 | T | C | 0.7607 | -0.0141 | 0.0023 | 3.82E-10 | 484680 | 0.970418 |
| rs1045411 | 13 | 31033232 | T | C | 0.2807 | -0.0139 | 0.0019 | 6.66E-14 | 791709 | 0.996695 |
| rs9595908 | 13 | 33184288 | T | C | 0.6427 | 0.0154 | 0.0017 | 3.73E-20 | 806589 | 0.997967 |
| rs9544930 | 13 | 36236428 | A | G | 0.1399 | -0.0136 | 0.0024 | 2.18E-08 | 711440 | 0.982769 |
| 13_40783323_C_T | 13 | 40783323 | T | C | 0.3184 | 0.0134 | 0.0018 | 1.69E-13 | 718556 | 0.993539 |
| rs41284828 | 13 | 50963685 | A | G | 0.0302 | -0.0341 | 0.0057 | 3.00E-09 | 484680 | 0.941826 |
| rs57207557 | 13 | 53485971 | T | G | 0.2683 | -0.0125 | 0.0022 | 7.01E-09 | 484680 | 0.994458 |
| rs1891946 | 13 | 53635837 | T | G | 0.2422 | -0.0125 | 0.0022 | 2.25E-08 | 484680 | 0.98923 |
| rs9596810 | 13 | 54056553 | T | C | 0.4287 | -0.0139 | 0.002 | 3.19E-12 | 484680 | 0.934774 |
| rs12429545 | 13 | 54102206 | A | G | 0.1218 | 0.0313 | 0.0024 | 1.42E-37 | 797614 | 0.978792 |
| rs7982447 | 13 | 54453811 | T | C | 0.8034 | -0.0143 | 0.0021 | 1.05E-11 | 708650 | 0.990746 |
| rs7995015 | 13 | 54828961 | T | G | 0.3254 | 0.0121 | 0.0018 | 3.02E-11 | 717708 | 0.989241 |
| rs9527455 | 13 | 56465597 | A | C | 0.2325 | -0.0121 | 0.002 | 1.85E-09 | 718658 | 0.993208 |
| rs9569808 | 13 | 58631525 | A | G | 0.7558 | 0.0195 | 0.002 | 2.12E-22 | 718693 | 0.998606 |
| rs9538141 | 13 | 59178258 | A | G | 0.5223 | 0.0154 | 0.0017 | 3.06E-19 | 717516 | 0.980165 |
| rs12430890 | 13 | 59317339 | A | G | 0.8684 | 0.0142 | 0.0025 | 1.27E-08 | 715002 | 0.998587 |
| rs722122 | 13 | 59399034 | A | C | 0.7604 | -0.0164 | 0.002 | 2.42E-16 | 718526 | 0.991594 |
| 13_59841918_A_G | 13 | 59841918 | A | G | 0.6515 | -0.0102 | 0.0018 | 1.80E-08 | 718618 | 0.996344 |
| rs2322622 | 13 | 60497331 | T | C | 0.6472 | -0.0098 | 0.0017 | 4.64E-09 | 806789 | 0.994176 |
| rs1394879 | 13 | 62683659 | C | G | 0.3998 | 0.011 | 0.0017 | 1.67E-10 | 718541 | 0.998984 |
| rs8181823 | 13 | 65477940 | A | C | 0.2344 | -0.0125 | 0.002 | 4.36E-10 | 718253 | 0.997754 |
| rs9540493 | 13 | 66205704 | A | G | 0.458 | 0.0129 | 0.0017 | 7.85E-15 | 803641 | 0.988808 |
| rs9599088 | 13 | 66768056 | T | C | 0.5663 | -0.0105 | 0.0019 | 4.85E-08 | 484680 | 0.98764 |
| rs9571687 | 13 | 67472713 | A | C | 0.3404 | -0.0122 | 0.0018 | 1.53E-11 | 718634 | 0.996301 |
| rs1441264 | 13 | 79580919 | A | G | 0.5846 | 0.0174 | 0.0017 | 7.60E-25 | 794966 | 0.952647 |
| rs7998796 | 13 | 81020036 | A | G | 0.6709 | -0.0102 | 0.0018 | 1.69E-08 | 718631 | 0.994687 |
| rs9531786 | 13 | 85983968 | C | G | 0.3715 | -0.0111 | 0.0018 | 4.11E-10 | 714692 | 0.993113 |
| rs77432547 | 13 | 86494817 | A | G | 0.7229 | -0.017 | 0.0021 | 1.44E-15 | 484680 | 0.999215 |
| rs76134593 | 13 | 90395288 | T | G | 0.0218 | 0.0368 | 0.0066 | 2.38E-08 | 484680 | 0.959667 |
| rs1927790 | 13 | 96922191 | T | C | 0.606 | -0.014 | 0.0016 | 1.57E-17 | 806731 | 0.997233 |
| rs2094474 | 13 | 98210083 | A | C | 0.7053 | -0.0105 | 0.0019 | 1.89E-08 | 718628 | 0.999411 |
| rs17574378 | 13 | 99116384 | T | C | 0.7267 | 0.014 | 0.0019 | 7.25E-14 | 718466 | 0.997313 |
| rs12147845 | 14 | 101144596 | T | C | 0.1115 | 0.0187 | 0.0027 | 4.97E-12 | 716257 | 0.991698 |
| rs1004573 | 14 | 101178235 | C | G | 0.1377 | 0.0145 | 0.0025 | 6.77E-09 | 717870 | 0.992042 |
| rs7161194 | 14 | 101529005 | A | G | 0.3437 | 0.019 | 0.0019 | 2.23E-24 | 773952 | 0.91083 |
| rs12431682 | 14 | 101539999 | T | C | 0.3416 | -0.0107 | 0.0017 | 1.61E-10 | 806157 | 0.99578 |
| rs10431745 | 14 | 102302372 | A | G | 0.9287 | 0.0199 | 0.0033 | 1.21E-09 | 718483 | 0.989264 |
| rs7159965 | 14 | 102780553 | C | G | 0.8288 | -0.0144 | 0.0022 | 1.12E-10 | 714850 | 0.996021 |
| rs3803286 | 14 | 103246470 | A | G | 0.3407 | 0.0178 | 0.0018 | 2.75E-23 | 718369 | 0.997594 |
| rs34508350 | 14 | 103493060 | T | C | 0.0353 | 0.0318 | 0.0054 | 3.12E-09 | 484680 | 0.929483 |
| rs2010281 | 14 | 103862322 | A | G | 0.3466 | -0.0155 | 0.0017 | 4.97E-20 | 806796 | 0.9976 |
| rs10438243 | 14 | 104345822 | A | G | 0.4243 | 0.0096 | 0.0017 | 2.47E-08 | 718681 | 1 |
| rs2754079 | 14 | 25886140 | A | G | 0.5709 | -0.0147 | 0.0017 | 1.09E-17 | 717993 | 0.998888 |
| rs10132280 | 14 | 25928179 | A | C | 0.311 | -0.0214 | 0.0018 | 2.28E-33 | 806477 | 0.988362 |
| rs12885454 | 14 | 29736838 | A | C | 0.3559 | -0.018 | 0.0017 | 5.44E-26 | 805503 | 0.997717 |
| rs1191601 | 14 | 30098349 | T | C | 0.4171 | 0.0115 | 0.0019 | 2.88E-09 | 484680 | 0.989768 |
| rs225882 | 14 | 30480123 | T | C | 0.7513 | 0.0113 | 0.0018 | 8.56E-10 | 806807 | 0.995257 |
| rs10483389 | 14 | 30495719 | T | C | 0.041 | 0.0334 | 0.0041 | 3.04E-16 | 794037 | 0.991533 |
| rs17522122 | 14 | 33302882 | T | G | 0.4667 | 0.0167 | 0.0017 | 7.93E-24 | 791700 | 0.991178 |
| rs12879626 | 14 | 34721134 | T | G | 0.3906 | -0.0099 | 0.0017 | 1.19E-08 | 718491 | 0.986149 |
| rs76420714 | 14 | 35628729 | T | C | 0.9018 | 0.019 | 0.0032 | 3.60E-09 | 484680 | 0.988664 |
| rs139866887 | 14 | 40105994 | A | C | 0.7978 | -0.0155 | 0.0025 | 8.98E-10 | 539041 | 0.989904 |
| rs1958898 | 14 | 40886886 | C | G | 0.2098 | -0.0146 | 0.0021 | 4.23E-12 | 718191 | 0.992713 |
| rs9671540 | 14 | 41268319 | T | C | 0.9166 | -0.0211 | 0.0035 | 1.82E-09 | 484680 | 0.966771 |
| rs12889085 | 14 | 42885336 | A | G | 0.5777 | -0.0132 | 0.0019 | 8.28E-12 | 484680 | 0.991863 |
| rs4900714 | 14 | 47302219 | T | G | 0.4776 | 0.015 | 0.0017 | 1.00E-18 | 718662 | 0.99649 |
| rs8022717 | 14 | 60111221 | A | G | 0.3491 | -0.0099 | 0.0018 | 2.62E-08 | 718541 | 0.999262 |
| rs217669 | 14 | 62360075 | T | C | 0.7224 | -0.0172 | 0.0021 | 6.27E-16 | 484680 | 1 |
| 14_62593297_C_T | 14 | 62593297 | T | C | 0.5608 | 0.0097 | 0.0016 | 2.43E-09 | 806720 | 0.988027 |
| rs11158434 | 14 | 63048448 | A | G | 0.1963 | 0.0126 | 0.0021 | 3.63E-09 | 718613 | 0.991604 |
| rs6573463 | 14 | 63352049 | A | C | 0.356 | 0.0101 | 0.0017 | 2.41E-09 | 806229 | 0.985188 |
| rs2296325 | 14 | 65414379 | T | C | 0.1917 | 0.0133 | 0.0024 | 3.70E-08 | 484680 | 0.994969 |
| rs3902951 | 14 | 69789755 | T | G | 0.7691 | -0.0134 | 0.0019 | 2.88E-12 | 794335 | 0.975453 |
| rs1275691 | 14 | 69816344 | A | G | 0.4578 | -0.0091 | 0.0016 | 2.01E-08 | 806683 | 1 |
| rs768840 | 14 | 73143457 | A | G | 0.4359 | 0.0109 | 0.0017 | 3.13E-10 | 717209 | 0.985107 |
| rs6574093 | 14 | 73194830 | T | G | 0.3644 | 0.0103 | 0.0018 | 8.46E-09 | 713636 | 0.980368 |
| rs6574100 | 14 | 73323682 | A | T | 0.5887 | 0.0118 | 0.0017 | 1.10E-11 | 718298 | 0.993101 |
| rs10151024 | 14 | 73746847 | A | G | 0.8073 | -0.0137 | 0.0024 | 1.38E-08 | 484680 | 0.994869 |
| rs17105272 | 14 | 77529783 | T | C | 0.3189 | 0.0117 | 0.0018 | 1.73E-10 | 717959 | 0.984647 |
| rs10146527 | 14 | 79499850 | T | C | 0.6353 | 0.0126 | 0.0017 | 2.38E-13 | 793469 | 0.998494 |
| rs7144011 | 14 | 79940383 | T | G | 0.2334 | 0.0263 | 0.002 | 2.37E-40 | 806721 | 0.996051 |
| rs4517716 | 14 | 83013876 | C | G | 0.7924 | -0.0117 | 0.002 | 4.27E-09 | 806706 | 1 |
| rs7141307 | 14 | 88375040 | T | C | 0.7397 | -0.0141 | 0.002 | 5.95E-13 | 717310 | 0.997471 |
| rs1951455 | 14 | 91512339 | T | C | 0.2813 | -0.0148 | 0.0019 | 6.05E-15 | 718607 | 0.996448 |
| rs60432564 | 14 | 91673259 | C | G | 0.7681 | 0.0142 | 0.0023 | 3.61E-10 | 484680 | 0.988671 |
| rs942066 | 14 | 94031914 | A | G | 0.373 | -0.0202 | 0.002 | 2.36E-24 | 484680 | 0.990093 |
| rs17761028 | 14 | 97455839 | T | C | 0.9438 | 0.0239 | 0.0042 | 1.94E-08 | 484680 | 0.95038 |
| rs17096510 | 14 | 98616999 | A | C | 0.87 | 0.0141 | 0.0025 | 3.05E-08 | 718450 | 0.991984 |
| rs3850422 | 14 | 99671788 | A | G | 0.4255 | -0.0112 | 0.0016 | 6.76E-12 | 806425 | 0.981066 |
| rs12594043 | 15 | 27034988 | C | G | 0.5205 | 0.0102 | 0.0017 | 1.31E-09 | 718508 | 0.99871 |
| rs12898464 | 15 | 29853387 | T | C | 0.8836 | -0.0169 | 0.003 | 1.55E-08 | 484680 | 0.988681 |
| rs7172627 | 15 | 31877690 | A | G | 0.523 | -0.0114 | 0.0017 | 1.81E-11 | 718111 | 0.997858 |
| rs7181610 | 15 | 35826859 | A | T | 0.8553 | 0.0146 | 0.0025 | 4.50E-09 | 696547 | 0.992241 |
| rs11636611 | 15 | 36391965 | T | C | 0.4981 | 0.0104 | 0.0017 | 8.85E-10 | 718657 | 0.998105 |
| rs2577947 | 15 | 42037570 | T | C | 0.8243 | -0.0135 | 0.0022 | 7.35E-10 | 718665 | 0.999187 |
| rs765787 | 15 | 45500047 | A | G | 0.8266 | 0.0137 | 0.0023 | 1.80E-09 | 716703 | 1 |
| rs12439798 | 15 | 46584787 | T | G | 0.438 | 0.0127 | 0.0017 | 1.21E-13 | 718639 | 0.995989 |
| rs12899905 | 15 | 47142090 | T | C | 0.2613 | -0.0113 | 0.0018 | 6.16E-10 | 806664 | 0.977471 |
| 15_47738063_A_G | 15 | 47738063 | A | G | 0.6097 | -0.0115 | 0.0016 | 2.80E-12 | 806738 | 0.999509 |
| rs12912380 | 15 | 47947291 | T | C | 0.1131 | -0.0171 | 0.0027 | 1.52E-10 | 790356 | 0.973345 |
| rs6493498 | 15 | 51754451 | T | C | 0.4465 | 0.0137 | 0.0016 | 4.84E-17 | 805967 | 0.995099 |
| rs117632017 | 15 | 52260107 | A | G | 0.0366 | 0.0372 | 0.0052 | 1.18E-12 | 484680 | 0.937212 |
| rs16965225 | 15 | 53143170 | T | G | 0.067 | 0.022 | 0.0033 | 5.32E-11 | 794658 | 0.974661 |
| rs8024806 | 15 | 53473990 | T | C | 0.9368 | 0.0254 | 0.0035 | 6.73E-13 | 717733 | 0.986416 |
| rs111673558 | 15 | 55469742 | A | G | 0.0237 | -0.0368 | 0.0063 | 5.43E-09 | 484680 | 0.986468 |
| rs12594742 | 15 | 58958465 | A | G | 0.1506 | 0.0138 | 0.0024 | 1.78E-08 | 718504 | 0.989126 |
| rs1032179 | 15 | 59479266 | A | T | 0.7542 | -0.0138 | 0.002 | 6.49E-12 | 718641 | 0.98816 |
| rs340025 | 15 | 60908307 | T | C | 0.4081 | -0.0127 | 0.0016 | 1.40E-14 | 806687 | 0.989995 |
| rs2899663 | 15 | 61194234 | A | G | 0.4883 | -0.0098 | 0.0016 | 2.07E-09 | 805175 | 0.997105 |
| rs8033510 | 15 | 61445514 | T | C | 0.3636 | 0.0107 | 0.0018 | 1.73E-09 | 717292 | 0.993096 |
| 15_62316035_C_T | 15 | 62316035 | T | C | 0.0264 | -0.0382 | 0.0052 | 1.30E-13 | 786284 | 1 |
| rs11635675 | 15 | 63793238 | T | G | 0.6546 | 0.0123 | 0.0018 | 1.14E-11 | 717037 | 0.997817 |
| 15_66040030_C_T | 15 | 66040030 | T | C | 0.1806 | -0.012 | 0.0022 | 4.06E-08 | 718651 | 0.998744 |
| rs113182412 | 15 | 66503584 | A | G | 0.1637 | -0.0145 | 0.0026 | 4.15E-08 | 484680 | 0.953609 |
| rs11629783 | 15 | 66741387 | C | G | 0.772 | 0.0143 | 0.002 | 1.43E-12 | 718567 | 0.996131 |
| rs12916494 | 15 | 66818045 | T | G | 0.0783 | 0.0179 | 0.0032 | 1.84E-08 | 718584 | 0.996229 |
| rs8030476 | 15 | 68046828 | A | G | 0.1 | 0.0177 | 0.0027 | 5.78E-11 | 804777 | 0.989796 |
| 15_68086838_A_G | 15 | 68086838 | A | G | 0.2285 | -0.0298 | 0.0019 | 3.60E-54 | 806797 | 1 |
| rs7164727 | 15 | 73093991 | T | C | 0.6888 | 0.0171 | 0.0017 | 2.38E-23 | 805992 | 0.995285 |
| rs77688257 | 15 | 73634582 | A | G | 0.0706 | -0.0266 | 0.0037 | 1.25E-12 | 484680 | 0.991742 |
| rs35364449 | 15 | 74278126 | T | C | 0.1053 | 0.0219 | 0.0031 | 2.13E-12 | 484680 | 0.989156 |
| rs2290573 | 15 | 75129594 | A | G | 0.5423 | 0.0101 | 0.0016 | 4.57E-10 | 806107 | 0.981029 |
| rs11855853 | 15 | 78012618 | T | C | 0.2593 | -0.0161 | 0.002 | 1.30E-16 | 717703 | 0.985451 |
| rs12595749 | 15 | 79432359 | A | G | 0.5956 | 0.0131 | 0.0017 | 1.53E-14 | 718687 | 0.998732 |
| rs12914623 | 15 | 80993570 | C | G | 0.2722 | -0.0159 | 0.0019 | 2.00E-16 | 718054 | 0.993297 |
| rs186017271 | 15 | 83368605 | A | G | 0.0152 | 0.0459 | 0.0081 | 1.60E-08 | 484680 | 0.925447 |
| rs62020775 | 15 | 89960286 | A | T | 0.1411 | -0.0152 | 0.0028 | 4.38E-08 | 484680 | 0.979704 |
| rs7498044 | 15 | 92573639 | A | G | 0.2134 | -0.0163 | 0.0024 | 4.89E-12 | 484680 | 0.978488 |
| rs11633626 | 15 | 95271378 | A | C | 0.6313 | -0.0157 | 0.0018 | 7.27E-19 | 718669 | 0.997012 |
| rs11638950 | 15 | 98301367 | A | C | 0.5888 | 0.0107 | 0.0017 | 6.25E-10 | 717907 | 0.985654 |
| rs58139454 | 15 | 99230162 | C | G | 0.2216 | -0.0145 | 0.0023 | 2.57E-10 | 484680 | 0.998645 |
| rs12908437 | 15 | 99287375 | T | C | 0.3541 | 0.0094 | 0.0017 | 2.57E-08 | 806113 | 0.991273 |
| 15_99511873_A_G | 15 | 99511873 | A | G | 0.2805 | -0.0115 | 0.0019 | 1.90E-09 | 706401 | 1 |
| rs6497676 | 16 | 10180223 | A | G | 0.6226 | -0.0101 | 0.0017 | 8.70E-09 | 718656 | 0.994633 |
| rs4985155 | 16 | 15129459 | A | G | 0.6689 | 0.0102 | 0.0017 | 3.39E-09 | 806518 | 0.997985 |
| rs12446632 | 16 | 19935389 | A | G | 0.1371 | -0.0353 | 0.0024 | 3.12E-50 | 801438 | 0.999682 |
| rs868554 | 16 | 20050466 | C | G | 0.7725 | -0.0185 | 0.002 | 2.94E-20 | 718529 | 0.983272 |
| rs11074446 | 16 | 20255123 | T | C | 0.8534 | 0.0214 | 0.0024 | 3.67E-19 | 794991 | 0.961765 |
| rs4483850 | 16 | 20375776 | A | T | 0.5214 | 0.0153 | 0.0017 | 1.18E-19 | 718501 | 0.998687 |
| 16_2097158_A_G | 16 | 2097158 | A | G | 0.2033 | -0.0153 | 0.002 | 4.38E-14 | 777941 | 1 |
| rs194809 | 16 | 23804956 | A | G | 0.1871 | 0.0126 | 0.0022 | 4.86E-09 | 718688 | 0.994332 |
| rs2107118 | 16 | 24310282 | A | G | 0.4998 | 0.0111 | 0.0018 | 3.23E-10 | 688172 | 1 |
| rs7195386 | 16 | 24578458 | T | C | 0.4924 | 0.0129 | 0.0017 | 2.17E-14 | 718678 | 0.994062 |
| rs7186893 | 16 | 24806420 | T | G | 0.2631 | -0.0143 | 0.0019 | 1.43E-13 | 717660 | 0.998542 |
| rs113394773 | 16 | 28328916 | T | C | 0.0865 | -0.0207 | 0.0036 | 1.08E-08 | 484680 | 0.871593 |
| rs7498665 | 16 | 28883241 | A | G | 0.6179 | -0.0285 | 0.0017 | 1.14E-66 | 804216 | 1 |
| rs3814883 | 16 | 29994922 | T | C | 0.4795 | 0.0227 | 0.0017 | 1.47E-40 | 717877 | 0.99001 |
| rs72791231 | 16 | 30111922 | A | G | 0.0431 | -0.0315 | 0.0047 | 2.86E-11 | 484680 | 0.977941 |
| rs7196129 | 16 | 30471109 | T | C | 0.461 | 0.0111 | 0.0016 | 1.21E-11 | 804938 | 0.977824 |
| rs9806804 | 16 | 30556490 | A | T | 0.8939 | -0.0174 | 0.0031 | 2.08E-08 | 484680 | 0.984956 |
| rs4889606 | 16 | 31011183 | A | G | 0.6305 | 0.0209 | 0.0017 | 2.90E-36 | 806567 | 0.992276 |
| 16_31523926_A_C | 16 | 31523926 | A | C | 0.3377 | 0.0096 | 0.0017 | 1.75E-08 | 806550 | 0.999279 |
| rs12448257 | 16 | 3599655 | A | G | 0.2185 | 0.0161 | 0.002 | 9.04E-16 | 797946 | 0.985252 |
| rs9925273 | 16 | 375900 | A | G | 0.8193 | 0.0129 | 0.0022 | 8.30E-09 | 718636 | 0.992455 |
| rs11866815 | 16 | 387867 | T | C | 0.2568 | -0.0153 | 0.0019 | 2.21E-16 | 806553 | 0.993912 |
| rs879620 | 16 | 4015729 | T | C | 0.5966 | 0.0226 | 0.0017 | 8.45E-39 | 718515 | 0.993348 |
| rs2239304 | 16 | 4037394 | A | G | 0.2642 | -0.0115 | 0.002 | 4.19E-09 | 717779 | 0.990305 |
| rs437115 | 16 | 4156423 | T | C | 0.5417 | 0.0098 | 0.0017 | 1.11E-08 | 718663 | 0.983129 |
| rs6500208 | 16 | 49011249 | A | G | 0.2021 | 0.0146 | 0.002 | 3.21E-13 | 794515 | 0.992371 |
| rs11862351 | 16 | 4903583 | A | G | 0.2137 | 0.0136 | 0.0023 | 5.75E-09 | 484680 | 0.993179 |
| rs1876359 | 16 | 4930100 | T | C | 0.3679 | 0.0128 | 0.0017 | 2.98E-14 | 806681 | 0.996086 |
| 16_49727574_A_C | 16 | 49727574 | A | C | 0.2501 | -0.0111 | 0.002 | 1.59E-08 | 718403 | 0.980305 |
| rs4784222 | 16 | 52537586 | C | G | 0.4775 | 0.0093 | 0.0017 | 4.56E-08 | 718458 | 0.993042 |
| rs12928335 | 16 | 53614746 | T | C | 0.0902 | -0.0247 | 0.0031 | 1.40E-15 | 706921 | 0.991612 |
| rs1477199 | 16 | 53712135 | A | G | 0.8426 | -0.0221 | 0.0023 | 5.15E-21 | 806707 | 0.997653 |
| rs17805308 | 16 | 53771716 | A | G | 0.963 | -0.0349 | 0.0052 | 1.64E-11 | 484680 | 0.947997 |
| rs13333228 | 16 | 53793798 | T | C | 0.309 | -0.0258 | 0.0018 | 5.57E-49 | 805349 | 0.979457 |
| rs9937053 | 16 | 53799507 | A | G | 0.4372 | 0.0721 | 0.0016 | 0 | 805790 | 0.999538 |
| rs111240785 | 16 | 53801116 | C | G | 0.9554 | -0.0382 | 0.0046 | 1.38E-16 | 484680 | 1 |
| rs16945088 | 16 | 53812524 | A | G | 0.9082 | 0.0285 | 0.0029 | 3.18E-22 | 805892 | 0.990589 |
| rs62033401 | 16 | 53814470 | T | C | 0.1298 | -0.0252 | 0.0028 | 6.97E-19 | 484680 | 0.996295 |
| rs75582195 | 16 | 53843848 | A | G | 0.0304 | 0.0453 | 0.0056 | 4.48E-16 | 484680 | 0.989248 |
| rs11075994 | 16 | 53850079 | A | G | 0.3393 | -0.0122 | 0.002 | 5.13E-10 | 651150 | 0.835894 |
| rs12921970 | 16 | 53865226 | C | G | 0.7382 | -0.0192 | 0.002 | 8.51E-23 | 709798 | 0.989873 |
| rs11076022 | 16 | 54150978 | A | G | 0.5761 | 0.0109 | 0.0016 | 3.02E-11 | 806593 | 0.99385 |
| rs9921416 | 16 | 54244754 | T | C | 0.4385 | -0.0096 | 0.0017 | 3.69E-08 | 717650 | 0.9866 |
| rs7189122 | 16 | 56471410 | T | C | 0.8331 | -0.0146 | 0.0022 | 4.31E-11 | 718692 | 1 |
| rs11075489 | 16 | 62803841 | T | C | 0.4739 | -0.0116 | 0.0017 | 1.23E-11 | 718240 | 0.995686 |
| rs2058527 | 16 | 6704749 | T | G | 0.2715 | -0.0115 | 0.0019 | 1.80E-09 | 718465 | 0.995574 |
| rs2307022 | 16 | 68381978 | A | G | 0.3278 | 0.0134 | 0.0017 | 7.57E-15 | 806658 | 0.999505 |
| rs5011579 | 16 | 69187318 | C | G | 0.2828 | -0.0135 | 0.0021 | 2.00E-10 | 484680 | 0.994326 |
| rs192042440 | 16 | 69285510 | T | C | 0.0243 | -0.0454 | 0.0064 | 9.17E-13 | 484680 | 0.951665 |
| rs889398 | 16 | 69556715 | T | C | 0.4151 | -0.0195 | 0.0016 | 3.23E-32 | 806620 | 0.990464 |
| rs71401807 | 16 | 70449124 | T | C | 0.0387 | -0.0324 | 0.0051 | 1.80E-10 | 484680 | 0.949905 |
| rs7919 | 16 | 70514828 | A | C | 0.4511 | -0.0155 | 0.0017 | 1.22E-19 | 718181 | 0.99177 |
| rs936297 | 16 | 70725815 | T | C | 0.0975 | -0.0186 | 0.003 | 3.07E-10 | 692470 | 0.956401 |
| rs11648802 | 16 | 71315078 | A | G | 0.4119 | 0.0095 | 0.0017 | 3.43E-08 | 718636 | 0.999321 |
| rs11642001 | 16 | 71899586 | A | G | 0.2107 | -0.0126 | 0.0021 | 8.49E-10 | 718665 | 0.998272 |
| rs12926804 | 16 | 71994932 | A | G | 0.873 | -0.0206 | 0.0029 | 5.69E-13 | 484680 | 0.99494 |
| rs811054 | 16 | 72251132 | T | C | 0.5333 | 0.0135 | 0.0016 | 2.36E-16 | 804912 | 0.984128 |
| rs756717 | 16 | 72996162 | A | G | 0.394 | -0.0134 | 0.0017 | 2.38E-15 | 804190 | 0.974174 |
| rs149211987 | 16 | 73249677 | A | C | 0.9742 | 0.0337 | 0.0061 | 2.77E-08 | 484680 | 0.978454 |
| rs9673839 | 16 | 76895693 | A | G | 0.5128 | -0.0121 | 0.0019 | 2.63E-10 | 484680 | 0.989534 |
| rs12449219 | 16 | 77261943 | C | G | 0.856 | -0.0164 | 0.0026 | 1.44E-10 | 717999 | 0.990394 |
| 16_81728081_A_C | 16 | 81728081 | A | C | 0.3826 | 0.0119 | 0.0017 | 1.90E-12 | 804422 | 0.984494 |
| rs12922346 | 16 | 82438337 | C | G | 0.2641 | 0.0133 | 0.002 | 1.48E-11 | 716945 | 0.981833 |
| rs4783241 | 16 | 82650384 | C | G | 0.4797 | -0.0095 | 0.0017 | 2.22E-08 | 716372 | 0.998996 |
| rs7206608 | 16 | 82872628 | C | G | 0.6751 | -0.013 | 0.0018 | 1.20E-12 | 717196 | 0.996158 |
| rs455527 | 16 | 89644001 | T | C | 0.9306 | 0.0194 | 0.0034 | 1.87E-08 | 794740 | 1 |
| rs12933086 | 16 | 9225494 | A | G | 0.746 | -0.0106 | 0.0019 | 3.96E-08 | 718568 | 1 |
| rs249293 | 16 | 9412222 | C | G | 0.6985 | 0.0123 | 0.0019 | 3.31E-11 | 718128 | 0.990379 |
| rs1990573 | 16 | 9713688 | A | G | 0.3061 | -0.0129 | 0.0018 | 2.11E-13 | 804412 | 0.989456 |
| rs1075901 | 17 | 15943910 | T | C | 0.4516 | -0.0118 | 0.0016 | 4.43E-13 | 806810 | 0.99862 |
| rs3923783 | 17 | 1843189 | A | C | 0.1784 | -0.0222 | 0.0022 | 4.12E-23 | 714524 | 0.998507 |
| rs6902 | 17 | 21142572 | A | C | 0.6205 | 0.0111 | 0.002 | 1.88E-08 | 484680 | 0.991502 |
| rs4986044 | 17 | 21261560 | T | C | 0.455 | -0.0177 | 0.0016 | 1.27E-27 | 802591 | 0.994308 |
| rs117642733 | 17 | 21284910 | T | C | 0.0437 | 0.0271 | 0.0049 | 4.16E-08 | 484680 | 0.893522 |
| rs7217226 | 17 | 2136065 | T | G | 0.656 | -0.0137 | 0.0017 | 9.43E-16 | 800625 | 0.997838 |
| rs111335664 | 17 | 2242821 | A | G | 0.7285 | 0.0123 | 0.0021 | 1.18E-08 | 484680 | 0.995908 |
| rs4795195 | 17 | 26318587 | A | G | 0.2872 | 0.0113 | 0.0019 | 3.33E-09 | 716736 | 0.991577 |
| rs11080090 | 17 | 27502029 | A | G | 0.8633 | -0.0138 | 0.0023 | 1.59E-09 | 795011 | 0.98428 |
| rs1038088 | 17 | 28074563 | T | G | 0.4879 | -0.0115 | 0.0016 | 1.37E-12 | 805864 | 1 |
| rs3930349 | 17 | 31475545 | A | C | 0.2089 | -0.014 | 0.002 | 7.68E-12 | 718661 | 0.993721 |
| rs4794977 | 17 | 32247585 | C | G | 0.5749 | 0.0118 | 0.002 | 1.56E-09 | 484680 | 0.977337 |
| rs12150665 | 17 | 34914787 | T | C | 0.5931 | 0.0168 | 0.0016 | 1.74E-24 | 806685 | 1 |
| rs6607337 | 17 | 35057373 | T | C | 0.2978 | -0.0124 | 0.0019 | 2.55E-11 | 718656 | 0.996805 |
| rs7219230 | 17 | 39286180 | T | C | 0.3394 | 0.0113 | 0.0018 | 4.87E-10 | 718657 | 0.998851 |
| rs684214 | 17 | 40696915 | T | C | 0.2786 | 0.0118 | 0.0021 | 2.79E-08 | 484680 | 0.995486 |
| rs2670854 | 17 | 41085683 | A | G | 0.7227 | 0.0111 | 0.0019 | 1.97E-09 | 804311 | 1 |
| rs4473241 | 17 | 42281282 | T | G | 0.3011 | 0.012 | 0.0021 | 5.35E-09 | 484680 | 0.99086 |
| rs242556 | 17 | 44002250 | A | T | 0.2325 | -0.0127 | 0.0021 | 1.34E-09 | 705471 | 0.947992 |
| rs10491182 | 17 | 46120767 | T | C | 0.9329 | 0.02 | 0.0031 | 9.09E-11 | 794959 | 0.997195 |
| rs208015 | 17 | 46252346 | T | C | 0.07 | 0.0352 | 0.0033 | 7.17E-26 | 718529 | 0.995504 |
| 17_46669430_C_T | 17 | 46669430 | T | C | 0.6447 | 0.0119 | 0.0018 | 1.87E-11 | 718669 | 1 |
| rs61162958 | 17 | 47068529 | T | G | 0.0936 | 0.0192 | 0.0033 | 7.39E-09 | 484680 | 0.977162 |
| rs11655587 | 17 | 47140794 | T | C | 0.3572 | -0.021 | 0.002 | 6.87E-26 | 484680 | 0.991833 |
| rs11652146 | 17 | 47422363 | A | G | 0.6866 | 0.0098 | 0.0017 | 2.06E-08 | 806544 | 0.997359 |
| rs3966782 | 17 | 4807098 | T | C | 0.6693 | -0.0119 | 0.0018 | 1.17E-10 | 663764 | 0.996971 |
| rs1000940 | 17 | 5283252 | A | G | 0.7168 | -0.0152 | 0.0018 | 7.80E-18 | 806516 | 0.997741 |
| rs62072006 | 17 | 52938468 | A | C | 0.8591 | -0.016 | 0.0028 | 6.14E-09 | 484680 | 0.990782 |
| rs8071182 | 17 | 55336155 | A | G | 0.1647 | 0.0125 | 0.0022 | 1.26E-08 | 784689 | 0.992106 |
| rs9910424 | 17 | 55799993 | T | C | 0.513 | -0.0092 | 0.0016 | 1.44E-08 | 806002 | 1 |
| rs11649864 | 17 | 56093061 | A | G | 0.0859 | 0.0192 | 0.003 | 2.52E-10 | 690306 | 0.997534 |
| rs8075273 | 17 | 61728881 | A | C | 0.2878 | -0.0137 | 0.0018 | 4.67E-14 | 806500 | 0.998729 |
| rs12602912 | 17 | 65870073 | T | C | 0.212 | 0.0166 | 0.002 | 2.90E-16 | 788746 | 0.999895 |
| rs312750 | 17 | 68343539 | A | G | 0.4986 | 0.0097 | 0.0016 | 2.57E-09 | 806739 | 0.988866 |
| rs2242449 | 17 | 7095507 | T | C | 0.4033 | 0.0099 | 0.0018 | 1.83E-08 | 704792 | 0.98185 |
| rs4969071 | 17 | 71134806 | T | C | 0.4604 | -0.0096 | 0.0017 | 2.21E-08 | 714798 | 0.997691 |
| rs2619976 | 17 | 71754545 | T | C | 0.4011 | 0.0105 | 0.0018 | 1.99E-09 | 716455 | 0.991513 |
| rs3744017 | 17 | 73871467 | A | G | 0.1931 | 0.0139 | 0.0022 | 2.43E-10 | 718595 | 0.998267 |
| rs8081039 | 17 | 75995829 | T | C | 0.0605 | 0.023 | 0.0037 | 6.02E-10 | 705384 | 0.986702 |
| rs1285245 | 17 | 77796889 | C | G | 0.3864 | -0.0106 | 0.0018 | 1.63E-09 | 716945 | 0.995081 |
| rs2279620 | 17 | 7792465 | C | G | 0.1203 | 0.0161 | 0.0028 | 5.05E-09 | 702981 | 0.991731 |
| rs12943620 | 17 | 78444658 | T | G | 0.2333 | -0.0124 | 0.0021 | 3.96E-09 | 688627 | 0.991581 |
| rs12939549 | 17 | 78611724 | A | G | 0.5572 | 0.018 | 0.0016 | 3.68E-28 | 806064 | 0.997733 |
| rs3935190 | 17 | 79084367 | A | G | 0.537 | -0.0155 | 0.0019 | 8.69E-16 | 484680 | 0.985972 |
| rs4969472 | 17 | 79976425 | A | G | 0.5588 | -0.0102 | 0.0018 | 1.07E-08 | 711031 | 0.986875 |
| rs11658335 | 17 | 80084821 | T | C | 0.5816 | -0.0109 | 0.0017 | 8.64E-11 | 744446 | 0.99993 |
| rs17681708 | 17 | 9792872 | T | C | 0.6674 | -0.0105 | 0.0018 | 7.18E-09 | 718674 | 0.999645 |
| rs7239048 | 18 | 13209542 | T | C | 0.8211 | -0.0144 | 0.0025 | 6.60E-09 | 484680 | 0.996471 |
| rs8097672 | 18 | 1839601 | A | T | 0.8545 | -0.0209 | 0.0024 | 3.26E-18 | 718627 | 0.982888 |
| rs891387 | 18 | 21103909 | T | C | 0.4951 | 0.0208 | 0.0017 | 9.26E-35 | 718548 | 0.999065 |
| rs16940823 | 18 | 22137319 | A | C | 0.1705 | -0.0157 | 0.0022 | 2.09E-12 | 718425 | 0.98121 |
| rs11083167 | 18 | 23793150 | A | C | 0.5202 | -0.0109 | 0.0019 | 1.36E-08 | 484680 | 0.983824 |
| rs9304502 | 18 | 24498214 | A | G | 0.2097 | -0.0116 | 0.0021 | 4.37E-08 | 718458 | 0.993923 |
| rs1941697 | 18 | 31251276 | A | G | 0.4466 | 0.0122 | 0.0017 | 8.36E-13 | 718538 | 1 |
| rs1365466 | 18 | 36182440 | T | C | 0.7278 | -0.012 | 0.0018 | 6.04E-11 | 806724 | 0.992983 |
| rs2587614 | 18 | 39105924 | T | C | 0.1431 | -0.0141 | 0.0025 | 2.14E-08 | 718683 | 0.998237 |
| rs1790376 | 18 | 39684795 | C | G | 0.4925 | -0.013 | 0.0017 | 5.24E-14 | 718291 | 0.988311 |
| 18_40147671_A_G | 18 | 40147671 | A | G | 0.6403 | -0.0125 | 0.0017 | 5.58E-14 | 806589 | 0.993979 |
| rs1356506 | 18 | 40708038 | T | C | 0.6287 | 0.0137 | 0.0018 | 8.39E-15 | 718632 | 0.99479 |
| rs143667773 | 18 | 40805664 | T | C | 0.0339 | 0.0325 | 0.0056 | 5.09E-09 | 484680 | 0.89964 |
| rs1435192 | 18 | 41060104 | A | C | 0.3008 | 0.0118 | 0.0019 | 2.18E-10 | 718631 | 0.993212 |
| rs954018 | 18 | 42598463 | A | G | 0.2895 | -0.0127 | 0.0018 | 3.57E-13 | 806752 | 0.999474 |
| rs16978350 | 18 | 42940499 | T | G | 0.2763 | -0.0128 | 0.0019 | 4.58E-11 | 718538 | 0.976509 |
| rs9965170 | 18 | 44788274 | A | G | 0.4346 | -0.0105 | 0.0017 | 1.02E-09 | 718473 | 0.999768 |
| rs7239114 | 18 | 45921214 | A | G | 0.5414 | 0.012 | 0.0017 | 6.24E-13 | 794467 | 0.983676 |
| rs9964756 | 18 | 50441817 | T | G | 0.1014 | -0.0158 | 0.0027 | 4.35E-09 | 718672 | 0.996347 |
| rs1461527 | 18 | 51562666 | C | G | 0.7382 | 0.0106 | 0.0019 | 4.72E-08 | 714988 | 0.997731 |
| rs8092503 | 18 | 52479487 | A | G | 0.7693 | -0.0162 | 0.0019 | 3.76E-17 | 798682 | 0.982595 |
| rs11659764 | 18 | 53335512 | A | T | 0.0511 | -0.0272 | 0.0039 | 3.22E-12 | 718163 | 0.989148 |
| rs7243357 | 18 | 56883319 | T | G | 0.8349 | 0.0198 | 0.0021 | 1.31E-20 | 806787 | 0.990374 |
| 18_57687573_A_G | 18 | 57687573 | A | G | 0.2317 | -0.0183 | 0.0019 | 1.14E-22 | 806581 | 0.989208 |
| rs6567160 | 18 | 57829135 | T | C | 0.7518 | -0.0552 | 0.0019 | 7.82E-184 | 806638 | 0.997773 |
| rs11152214 | 18 | 57854144 | A | C | 0.872 | 0.0249 | 0.0026 | 4.82E-21 | 704658 | 1 |
| rs9947301 | 18 | 57869590 | T | C | 0.0728 | -0.0328 | 0.0031 | 4.67E-26 | 783714 | 0.992597 |
| rs117085301 | 18 | 57926294 | T | C | 0.0467 | -0.0263 | 0.0047 | 1.53E-08 | 484680 | 0.9438 |
| rs9675376 | 18 | 57969244 | A | G | 0.2911 | 0.0349 | 0.0018 | 4.15E-83 | 794858 | 0.999849 |
| rs79131845 | 18 | 58010896 | C | G | 0.9238 | 0.0347 | 0.0036 | 1.55E-21 | 484680 | 0.973839 |
| rs17066842 | 18 | 58040624 | A | G | 0.0393 | -0.0644 | 0.0042 | 6.32E-54 | 781236 | 0.985519 |
| rs8097783 | 18 | 58051294 | A | G | 0.0882 | -0.037 | 0.003 | 4.43E-34 | 806596 | 1 |
| rs1943227 | 18 | 58058711 | A | G | 0.8776 | 0.0152 | 0.0026 | 6.17E-09 | 772443 | 0.929015 |
| rs77162980 | 18 | 58117757 | A | C | 0.9721 | 0.0617 | 0.0059 | 5.45E-26 | 484680 | 0.981425 |
| rs11659639 | 18 | 58167612 | T | G | 0.9804 | -0.0377 | 0.0065 | 5.14E-09 | 698239 | 0.98412 |
| rs67611440 | 18 | 58180796 | T | C | 0.3983 | -0.014 | 0.002 | 7.83E-13 | 484680 | 0.98819 |
| rs17067102 | 18 | 58232130 | T | C | 0.2134 | -0.0126 | 0.0022 | 9.58E-09 | 708846 | 0.922375 |
| rs1031670 | 18 | 60723787 | T | C | 0.5169 | -0.0111 | 0.0017 | 1.03E-10 | 705278 | 0.997743 |
| rs12454712 | 18 | 60845884 | T | C | 0.6245 | -0.0136 | 0.0018 | 1.05E-14 | 728218 | 1 |
| rs2012927 | 18 | 63297672 | A | G | 0.3398 | 0.0144 | 0.0017 | 4.02E-17 | 806712 | 0.994307 |
| rs8089514 | 18 | 69224478 | A | T | 0.3663 | 0.0124 | 0.0018 | 8.09E-12 | 717273 | 0.974849 |
| rs17283867 | 18 | 73202388 | A | C | 0.362 | 0.0105 | 0.0018 | 5.45E-09 | 718688 | 0.996366 |
| rs11150911 | 18 | 73498528 | A | C | 0.2848 | 0.0118 | 0.0018 | 3.72E-11 | 802197 | 0.991862 |
| rs478707 | 18 | 7543207 | T | C | 0.2032 | -0.015 | 0.0024 | 1.98E-10 | 484680 | 0.990857 |
| rs594821 | 18 | 76745589 | T | C | 0.0912 | -0.0191 | 0.003 | 3.22E-10 | 718592 | 1 |
| rs2072597 | 19 | 12996740 | A | G | 0.6946 | -0.0125 | 0.0021 | 1.52E-09 | 484680 | 1 |
| rs17639568 | 19 | 13157540 | A | G | 0.2852 | -0.0101 | 0.0018 | 3.87E-08 | 770506 | 0.991337 |
| rs273505 | 19 | 18217147 | T | C | 0.5783 | -0.0173 | 0.0019 | 3.32E-19 | 484680 | 0.997811 |
| 19_18454825_A_G | 19 | 18454825 | A | G | 0.7197 | 0.0172 | 0.0018 | 5.23E-21 | 804268 | 0.990931 |
| rs16982345 | 19 | 18500722 | A | G | 0.2509 | 0.0118 | 0.002 | 4.36E-09 | 676979 | 0.992741 |
| rs12981256 | 19 | 1865901 | A | G | 0.5284 | 0.0151 | 0.0017 | 1.43E-18 | 715373 | 0.988717 |
| rs7258722 | 19 | 18808915 | A | T | 0.4131 | -0.02 | 0.0017 | 1.81E-30 | 717923 | 0.987088 |
| rs56369935 | 19 | 18814013 | A | G | 0.0438 | 0.0296 | 0.0048 | 5.33E-10 | 484680 | 0.963733 |
| rs4808934 | 19 | 19370917 | T | C | 0.171 | -0.0175 | 0.0022 | 3.85E-15 | 752265 | 0.998032 |
| rs34858588 | 19 | 19457235 | C | G | 0.9217 | -0.0239 | 0.0036 | 1.87E-11 | 484680 | 0.99406 |
| rs4807179 | 19 | 1956035 | A | G | 0.5988 | 0.0138 | 0.0018 | 3.76E-15 | 717407 | 0.995659 |
| 19_19789528_A_G | 19 | 19789528 | A | G | 0.9021 | 0.017 | 0.0029 | 4.32E-09 | 795031 | 1 |
| rs45521740 | 19 | 2245622 | A | G | 0.0555 | 0.0317 | 0.0042 | 3.26E-14 | 484680 | 1 |
| rs12462975 | 19 | 30272202 | A | G | 0.3243 | 0.0193 | 0.0018 | 1.47E-25 | 717340 | 0.988016 |
| 19_30610400_A_G | 19 | 30610400 | A | G | 0.4826 | -0.0107 | 0.0017 | 3.85E-10 | 717983 | 1 |
| rs4805525 | 19 | 30673835 | A | C | 0.2387 | -0.0129 | 0.002 | 4.62E-11 | 717978 | 0.989992 |
| rs33430 | 19 | 30939443 | A | G | 0.3815 | -0.0123 | 0.0017 | 1.94E-12 | 718206 | 0.979267 |
| rs11084553 | 19 | 31019780 | A | G | 0.8497 | 0.0195 | 0.0024 | 3.03E-16 | 718701 | 0.996992 |
| rs7255223 | 19 | 32824310 | A | C | 0.2746 | -0.0109 | 0.0019 | 1.24E-08 | 718379 | 0.996523 |
| rs10402145 | 19 | 33966641 | A | G | 0.3041 | 0.012 | 0.0019 | 1.90E-10 | 718675 | 0.997093 |
| rs29938 | 19 | 34311481 | T | C | 0.3425 | -0.0158 | 0.0017 | 3.81E-20 | 803927 | 0.995608 |
| rs895330 | 19 | 4060707 | C | G | 0.806 | 0.0196 | 0.0022 | 1.59E-19 | 712588 | 0.994002 |
| rs3826705 | 19 | 42637232 | T | C | 0.8883 | -0.0148 | 0.0027 | 2.30E-08 | 717293 | 0.998184 |
| rs9352 | 19 | 4442336 | T | C | 0.5349 | 0.0095 | 0.0017 | 2.24E-08 | 718642 | 1 |
| rs7254892 | 19 | 45389596 | A | G | 0.0344 | 0.0265 | 0.0046 | 1.18E-08 | 731144 | 1 |
| rs6857 | 19 | 45392254 | T | C | 0.1656 | -0.0246 | 0.0023 | 5.92E-26 | 712142 | 0.99456 |
| rs405509 | 19 | 45408836 | T | G | 0.4895 | -0.0097 | 0.0017 | 5.36E-09 | 749922 | 1 |
| rs62118504 | 19 | 45734751 | A | G | 0.6104 | -0.0116 | 0.002 | 3.03E-09 | 484680 | 0.989049 |
| rs12981080 | 19 | 46117532 | T | C | 0.0495 | -0.0269 | 0.0046 | 4.61E-09 | 484680 | 0.9198 |
| 19_46158473_C_T | 19 | 46158473 | T | C | 0.1673 | 0.0125 | 0.0022 | 1.23E-08 | 788944 | 0.995782 |
| rs11672660 | 19 | 46180184 | T | C | 0.1871 | -0.0338 | 0.0021 | 6.83E-60 | 781854 | 0.99966 |
| rs3810329 | 19 | 46367945 | C | G | 0.1476 | 0.0124 | 0.0022 | 2.43E-08 | 805631 | 1 |
| rs2341637 | 19 | 46482344 | A | G | 0.3137 | 0.0117 | 0.0021 | 2.01E-08 | 484680 | 0.981612 |
| 19_47569003_A_G | 19 | 47569003 | A | G | 0.6503 | 0.0262 | 0.0017 | 2.36E-51 | 780941 | 1 |
| rs117516426 | 19 | 47660977 | T | C | 0.0764 | 0.0235 | 0.0036 | 8.29E-11 | 484680 | 0.987632 |
| rs4545921 | 19 | 49646006 | A | G | 0.3971 | 0.0117 | 0.0019 | 1.81E-09 | 484680 | 0.996312 |
| rs61746970 | 19 | 51132746 | A | G | 0.0386 | 0.0308 | 0.0051 | 1.36E-09 | 484680 | 0.948128 |
| rs12979351 | 19 | 51304404 | C | G | 0.0792 | 0.0177 | 0.0032 | 3.33E-08 | 634044 | 1 |
| rs148108087 | 19 | 51781455 | A | G | 0.0522 | -0.0302 | 0.0045 | 2.34E-11 | 484680 | 0.905705 |
| rs4802778 | 19 | 51812034 | A | G | 0.5887 | -0.0111 | 0.0018 | 2.57E-10 | 715023 | 0.99496 |
| rs2423668 | 20 | 12430673 | T | C | 0.4196 | 0.0106 | 0.0018 | 7.84E-09 | 557523 | 0.996127 |
| rs1884389 | 20 | 1410582 | T | C | 0.4386 | -0.0108 | 0.0017 | 3.72E-10 | 718491 | 0.990338 |
| rs17272434 | 20 | 15085365 | A | G | 0.3014 | -0.0097 | 0.0018 | 4.27E-08 | 806748 | 0.989526 |
| rs8123881 | 20 | 15819495 | A | G | 0.864 | -0.0177 | 0.0024 | 1.05E-13 | 805952 | 0.990829 |
| rs4814512 | 20 | 16564210 | A | C | 0.8075 | 0.0126 | 0.0021 | 1.29E-09 | 718651 | 1 |
| rs947088 | 20 | 17171373 | T | G | 0.7351 | 0.0126 | 0.0019 | 3.51E-11 | 718449 | 0.991826 |
| rs6136033 | 20 | 17208504 | A | C | 0.4246 | -0.0095 | 0.0017 | 2.57E-08 | 782042 | 0.970403 |
| rs2226103 | 20 | 2095431 | A | G | 0.5711 | 0.011 | 0.0019 | 9.39E-09 | 484680 | 0.999325 |
| 20_21381121_C_T | 20 | 21381121 | T | C | 0.1074 | 0.0195 | 0.0028 | 2.59E-12 | 718603 | 0.991149 |
| rs6083632 | 20 | 24802508 | T | C | 0.3712 | -0.0121 | 0.0018 | 6.60E-12 | 718619 | 0.990561 |
| rs8122855 | 20 | 25192049 | A | G | 0.3377 | 0.0137 | 0.0018 | 4.11E-14 | 717822 | 0.999506 |
| 20_25195509_A_G | 20 | 25195509 | A | G | 0.0318 | -0.0346 | 0.0047 | 3.17E-13 | 783943 | 0.981924 |
| rs4813619 | 20 | 2815715 | T | G | 0.5305 | -0.0106 | 0.0018 | 1.70E-09 | 664186 | 0.982677 |
| rs4911382 | 20 | 32553095 | T | C | 0.5939 | 0.0127 | 0.0017 | 1.69E-13 | 718661 | 0.996055 |
| 20_33452600_C_T | 20 | 33452600 | T | C | 0.2368 | -0.0116 | 0.002 | 5.50E-09 | 718612 | 0.996427 |
| rs4812405 | 20 | 35276585 | A | C | 0.0763 | -0.0202 | 0.0033 | 1.28E-09 | 706013 | 0.98774 |
| rs6029180 | 20 | 39178923 | A | G | 0.6882 | -0.0106 | 0.0018 | 9.00E-09 | 718622 | 0.973801 |
| rs16989232 | 20 | 39291784 | A | G | 0.3864 | 0.0115 | 0.0017 | 6.19E-12 | 805861 | 0.973828 |
| rs6130360 | 20 | 42010996 | A | G | 0.8492 | 0.0162 | 0.0024 | 7.49E-12 | 718089 | 0.997625 |
| rs2284265 | 20 | 43517041 | T | C | 0.2697 | 0.0117 | 0.0022 | 4.81E-08 | 484680 | 0.999508 |
| rs2425840 | 20 | 44904838 | A | C | 0.5958 | -0.0119 | 0.0017 | 6.38E-12 | 714506 | 0.992876 |
| rs3092209 | 20 | 45489808 | A | T | 0.5753 | -0.0101 | 0.0017 | 8.26E-09 | 717420 | 0.986981 |
| rs6019483 | 20 | 47495656 | A | T | 0.1743 | -0.0175 | 0.0023 | 3.68E-14 | 717290 | 0.987558 |
| rs6067688 | 20 | 49857211 | T | G | 0.6939 | -0.0103 | 0.0018 | 1.77E-08 | 718552 | 1 |
| rs17201143 | 20 | 50402079 | T | C | 0.3054 | -0.0112 | 0.0018 | 1.10E-09 | 718638 | 0.998769 |
| rs2426424 | 20 | 50875602 | T | C | 0.0765 | 0.0185 | 0.0028 | 5.73E-11 | 794898 | 0.99295 |
| rs17806224 | 20 | 51065854 | A | G | 0.1808 | -0.026 | 0.0022 | 7.91E-32 | 718699 | 0.996429 |
| rs6023633 | 20 | 53453850 | A | G | 0.7767 | -0.013 | 0.0021 | 2.32E-10 | 717208 | 0.965453 |
| rs559267 | 20 | 54157497 | A | G | 0.6696 | -0.0103 | 0.0017 | 2.53E-09 | 806790 | 0.996407 |
| rs6014523 | 20 | 54394975 | T | C | 0.7938 | 0.0134 | 0.0021 | 4.40E-10 | 718656 | 0.995921 |
| rs6010784 | 20 | 61540319 | T | C | 0.5062 | 0.0106 | 0.0016 | 6.91E-11 | 803088 | 0.999086 |
| rs310618 | 20 | 62127121 | T | C | 0.6597 | -0.0104 | 0.0018 | 5.58E-09 | 741776 | 0.980311 |
| rs11699828 | 20 | 62157198 | A | G | 0.0344 | -0.0371 | 0.0057 | 9.55E-11 | 484680 | 0.834314 |
| rs6512302 | 20 | 62691550 | C | G | 0.7391 | 0.0134 | 0.002 | 1.52E-11 | 718338 | 0.988665 |
| 20_6612832_A_G | 20 | 6612832 | A | G | 0.3747 | -0.0184 | 0.0017 | 2.70E-28 | 806711 | 0.992545 |
| rs961917 | 20 | 8308190 | C | G | 0.7244 | 0.0107 | 0.0019 | 1.51E-08 | 714818 | 0.992741 |
| rs2327129 | 20 | 9047668 | A | G | 0.8232 | -0.0146 | 0.0025 | 7.40E-09 | 484680 | 0.986677 |
| rs2064044 | 21 | 22119890 | A | C | 0.8062 | -0.0123 | 0.0021 | 1.02E-08 | 718416 | 0.997063 |
| rs2832283 | 21 | 30690558 | A | G | 0.2254 | 0.0115 | 0.002 | 4.72E-09 | 806772 | 0.991883 |
| rs17193211 | 21 | 38885506 | T | C | 0.0695 | -0.0209 | 0.0035 | 1.74E-09 | 714254 | 0.96796 |
| rs11702843 | 21 | 40123740 | A | G | 0.2531 | 0.0121 | 0.002 | 2.16E-09 | 704082 | 0.978172 |
| rs13047416 | 21 | 40309436 | C | G | 0.6263 | 0.0152 | 0.0018 | 6.93E-18 | 717914 | 0.990281 |
| rs8134638 | 21 | 40644170 | T | C | 0.6273 | -0.0133 | 0.002 | 1.57E-11 | 484680 | 0.998467 |
| rs2837398 | 21 | 41427168 | A | C | 0.6075 | -0.0112 | 0.0017 | 9.50E-11 | 706864 | 0.998366 |
| rs2838006 | 21 | 42653567 | T | C | 0.3566 | -0.0132 | 0.0018 | 2.03E-13 | 718633 | 0.988289 |
| rs4819021 | 21 | 46466927 | T | C | 0.5173 | 0.0125 | 0.0019 | 9.94E-11 | 484680 | 0.979275 |
| rs76040172 | 21 | 46488959 | A | G | 0.054 | -0.0345 | 0.0043 | 6.04E-16 | 484680 | 0.986318 |
| rs427943 | 21 | 46570896 | A | C | 0.4288 | -0.0177 | 0.0017 | 3.60E-25 | 742360 | 0.991994 |
| rs62240487 | 22 | 18147568 | A | G | 0.0405 | -0.0265 | 0.0049 | 4.99E-08 | 484680 | 0.99431 |
| rs11538 | 22 | 18220831 | A | G | 0.8284 | -0.0138 | 0.0023 | 1.09E-09 | 718441 | 1 |
| rs165656 | 22 | 19948863 | C | G | 0.5133 | 0.0109 | 0.0019 | 1.60E-08 | 484680 | 0.985185 |
| rs2238799 | 22 | 20109325 | A | G | 0.6187 | 0.0102 | 0.0018 | 8.65E-09 | 714858 | 0.988747 |
| rs6000329 | 22 | 36959219 | A | G | 0.5626 | -0.0112 | 0.002 | 1.12E-08 | 484680 | 0.955674 |
| rs12628891 | 22 | 38317137 | T | C | 0.3186 | -0.0115 | 0.0019 | 5.85E-10 | 718155 | 0.990813 |
| rs2277844 | 22 | 38577515 | A | G | 0.5483 | -0.0095 | 0.0017 | 2.48E-08 | 718710 | 0.998612 |
| rs11089885 | 22 | 38809485 | T | C | 0.515 | -0.0097 | 0.0017 | 1.78E-08 | 714780 | 0.99806 |
| rs2064258 | 22 | 40534964 | T | C | 0.5254 | 0.0114 | 0.0019 | 2.78E-09 | 484680 | 0.978614 |
| 22_40654276_C_T | 22 | 40654276 | T | C | 0.6407 | 0.0161 | 0.0018 | 2.90E-19 | 715009 | 1 |
| rs28489620 | 22 | 41804716 | A | G | 0.2818 | -0.0151 | 0.0021 | 1.42E-12 | 484680 | 0.981569 |
| rs9615723 | 22 | 48386670 | T | C | 0.5479 | -0.0118 | 0.0019 | 1.25E-09 | 484680 | 0.971168 |
| rs13053342 | 22 | 48871624 | A | G | 0.4032 | 0.0125 | 0.0018 | 1.67E-12 | 716899 | 0.99037 |
| rs5771118 | 22 | 50714289 | T | C | 0.26 | -0.013 | 0.0022 | 2.56E-09 | 484680 | 0.985077 |

| **WHRadjBMI SNPs** | **CHR** | **POS** | **Effect Allele** | **Other Allele** | **Effect Allele Frequency** | **BETA** | **SE** | **P** | **N** | **INFO** |
| --- | --- | --- | --- | --- | --- | --- | --- | --- | --- | --- |
| rs1415361 | 1 | 103562263 | T | C | 0.593 | 0.0161 | 0.002 | 5.83E-16 | 484563 | 0.995191 |
| rs11579145 | 1 | 110501597 | A | G | 0.5044 | -0.0099 | 0.0018 | 2.79E-08 | 626911 | 0.992413 |
| rs3789615 | 1 | 114941326 | T | C | 0.4281 | -0.0118 | 0.0017 | 7.33E-12 | 681864 | 0.99654 |
| rs7534091 | 1 | 118864616 | A | G | 0.7202 | 0.0171 | 0.0019 | 1.40E-18 | 692250 | 0.99869 |
| rs149691914 | 1 | 119097362 | C | G | 0.0351 | -0.0329 | 0.0054 | 1.38E-09 | 484563 | 0.970215 |
| rs115499511 | 1 | 119322589 | T | C | 0.9641 | 0.0587 | 0.0054 | 8.13E-28 | 484563 | 0.973448 |
| rs12144610 | 1 | 119501103 | A | T | 0.831 | -0.0214 | 0.0022 | 4.00E-22 | 694541 | 0.998752 |
| rs10923724 | 1 | 119546842 | T | C | 0.5569 | 0.0347 | 0.0017 | 1.25E-89 | 694500 | 0.992599 |
| rs3790549 | 1 | 119575818 | C | G | 0.9513 | 0.0256 | 0.0042 | 1.15E-09 | 693212 | 1 |
| rs6428798 | 1 | 119680967 | A | G | 0.4347 | 0.0102 | 0.0017 | 3.11E-09 | 694507 | 0.995363 |
| rs6656857 | 1 | 119846011 | C | G | 0.9042 | 0.0197 | 0.0031 | 1.14E-10 | 626991 | 0.972753 |
| rs146061372 | 1 | 119876724 | A | G | 0.0542 | 0.0294 | 0.0045 | 4.27E-11 | 484563 | 0.951996 |
| rs11204762 | 1 | 150999737 | A | G | 0.7695 | 0.014 | 0.0021 | 3.39E-11 | 625850 | 1 |
| rs951240 | 1 | 154793763 | A | G | 0.737 | 0.012 | 0.002 | 3.06E-09 | 627174 | 1 |
| rs905938 | 1 | 154991389 | T | C | 0.7233 | 0.0243 | 0.002 | 1.35E-35 | 692430 | 1 |
| rs140953793 | 1 | 155908657 | C | G | 0.9813 | 0.058 | 0.0076 | 1.51E-14 | 484563 | 0.931496 |
| rs2274319 | 1 | 156450873 | T | C | 0.3462 | 0.0116 | 0.0019 | 5.34E-10 | 627161 | 0.99708 |
| rs4656912 | 1 | 160412880 | A | G | 0.2654 | -0.0152 | 0.0022 | 8.77E-12 | 484563 | 0.99076 |
| rs4656994 | 1 | 161179877 | A | G | 0.2065 | 0.0123 | 0.0022 | 1.60E-08 | 627308 | 0.998196 |
| rs4657305 | 1 | 163762232 | T | C | 0.462 | -0.0117 | 0.0018 | 6.55E-11 | 627238 | 0.99331 |
| rs2275560 | 1 | 164529666 | A | G | 0.2547 | 0.0135 | 0.0023 | 5.30E-09 | 594287 | 0.78392 |
| rs2279469 | 1 | 164740405 | C | G | 0.4996 | 0.013 | 0.0018 | 7.92E-13 | 616758 | 0.983999 |
| rs6426912 | 1 | 165321568 | T | C | 0.1087 | 0.0191 | 0.0029 | 6.15E-11 | 605393 | 0.986598 |
| rs12142060 | 1 | 170176678 | C | G | 0.1046 | 0.0203 | 0.003 | 1.25E-11 | 627198 | 0.993338 |
| rs4575136 | 1 | 170198294 | T | G | 0.4596 | 0.011 | 0.002 | 4.45E-08 | 484563 | 0.969725 |
| rs139949504 | 1 | 170362856 | T | G | 0.9844 | 0.0457 | 0.0082 | 2.60E-08 | 484563 | 0.938717 |
| rs10919388 | 1 | 170372503 | A | C | 0.2729 | -0.0333 | 0.0019 | 9.71E-66 | 665612 | 0.991758 |
| rs6702078 | 1 | 170719666 | A | C | 0.2155 | 0.0153 | 0.0022 | 2.45E-12 | 626795 | 0.989867 |
| rs912299 | 1 | 172187250 | A | G | 0.3997 | -0.0139 | 0.0018 | 4.26E-14 | 626161 | 0.959617 |
| rs7552877 | 1 | 172269398 | T | C | 0.9328 | 0.0204 | 0.0036 | 2.12E-08 | 610559 | 0.998047 |
| rs714515 | 1 | 172352990 | A | G | 0.5555 | -0.0276 | 0.0017 | 6.49E-59 | 687964 | 0.999231 |
| rs2269615 | 1 | 172415451 | T | C | 0.5515 | -0.0095 | 0.0017 | 3.37E-08 | 694579 | 0.993977 |
| rs9435732 | 1 | 17308158 | T | C | 0.2396 | -0.0128 | 0.0019 | 2.57E-11 | 693273 | 1 |
| rs6425326 | 1 | 175198598 | T | C | 0.254 | -0.0112 | 0.0019 | 6.53E-09 | 694631 | 0.995896 |
| rs352300 | 1 | 176250052 | C | G | 0.5453 | 0.0106 | 0.0018 | 2.92E-09 | 627306 | 0.999405 |
| rs10913257 | 1 | 176778610 | T | G | 0.4507 | -0.0107 | 0.0018 | 2.38E-09 | 627006 | 0.992108 |
| rs6658424 | 1 | 200049302 | A | T | 0.2845 | -0.0143 | 0.002 | 2.88E-13 | 627301 | 0.993948 |
| rs1874142 | 1 | 203094406 | A | G | 0.3895 | -0.0107 | 0.0018 | 8.23E-09 | 626699 | 0.984136 |
| rs2821231 | 1 | 203518382 | T | C | 0.4599 | 0.0115 | 0.0018 | 4.90E-10 | 598262 | 0.983807 |
| rs3747636 | 1 | 204403659 | A | G | 0.7717 | 0.0129 | 0.0021 | 1.60E-09 | 627280 | 0.988522 |
| rs3903399 | 1 | 205041542 | T | C | 0.7847 | -0.0124 | 0.0022 | 1.31E-08 | 626549 | 0.996769 |
| rs3851294 | 1 | 205130413 | A | G | 0.0965 | -0.0257 | 0.0031 | 9.03E-17 | 627266 | 1 |
| rs4951583 | 1 | 212416078 | T | C | 0.5863 | -0.0133 | 0.0018 | 2.15E-13 | 627164 | 0.999212 |
| rs61838772 | 1 | 219587384 | T | C | 0.052 | -0.0249 | 0.0044 | 1.96E-08 | 484563 | 1 |
| rs2791550 | 1 | 219655369 | T | G | 0.2478 | -0.037 | 0.0018 | 3.35E-93 | 694588 | 0.999956 |
| rs1337100 | 1 | 219724937 | A | G | 0.0796 | 0.0184 | 0.0032 | 6.30E-09 | 694516 | 0.996347 |
| rs2494192 | 1 | 219749261 | A | G | 0.8113 | -0.0266 | 0.0022 | 3.03E-34 | 694585 | 0.998708 |
| rs61836039 | 1 | 220023215 | A | C | 0.0237 | 0.0475 | 0.0071 | 2.42E-11 | 484563 | 0.830947 |
| rs2784270 | 1 | 221094967 | A | G | 0.4811 | 0.0106 | 0.0018 | 3.61E-09 | 626968 | 0.987673 |
| rs715300 | 1 | 221290755 | T | C | 0.3095 | 0.0144 | 0.0019 | 1.11E-13 | 627315 | 0.995543 |
| rs10779436 | 1 | 221626007 | T | C | 0.2854 | -0.012 | 0.002 | 1.78E-09 | 627005 | 0.997838 |
| rs6699339 | 1 | 224050693 | T | C | 0.294 | -0.0124 | 0.0022 | 1.16E-08 | 484563 | 0.990662 |
| rs2235529 | 1 | 22450487 | T | C | 0.1612 | -0.0182 | 0.0025 | 1.34E-13 | 627109 | 0.993003 |
| rs10917200 | 1 | 22626370 | T | G | 0.1706 | -0.015 | 0.0024 | 3.33E-10 | 627288 | 0.993994 |
| rs10799424 | 1 | 227755475 | A | T | 0.2013 | -0.0134 | 0.0023 | 3.09E-09 | 627296 | 0.997166 |
| rs10803236 | 1 | 236191170 | A | G | 0.5275 | -0.0103 | 0.0019 | 2.94E-08 | 624671 | 0.998041 |
| rs7532311 | 1 | 23708486 | T | C | 0.9309 | -0.0211 | 0.0035 | 1.75E-09 | 627302 | 0.991121 |
| rs2298632 | 1 | 23710475 | T | C | 0.4861 | -0.0155 | 0.0017 | 4.96E-19 | 682626 | 0.961337 |
| rs3218121 | 1 | 23858317 | A | G | 0.0844 | 0.0217 | 0.0032 | 9.41E-12 | 693833 | 0.996839 |
| rs807067 | 1 | 26197063 | T | C | 0.5285 | 0.011 | 0.0017 | 1.23E-10 | 693717 | 0.997679 |
| rs2997447 | 1 | 26387423 | A | G | 0.2139 | -0.0137 | 0.0021 | 1.38E-10 | 693774 | 0.976298 |
| rs547976 | 1 | 29503214 | A | G | 0.2677 | -0.0106 | 0.0019 | 3.12E-08 | 694556 | 1 |
| rs717795 | 1 | 2970464 | T | C | 0.8014 | -0.0196 | 0.0023 | 6.86E-18 | 627060 | 0.991382 |
| rs645383 | 1 | 36373823 | C | G | 0.9192 | -0.0188 | 0.0033 | 1.17E-08 | 625924 | 0.994906 |
| rs11205773 | 1 | 51284905 | T | C | 0.9074 | 0.0187 | 0.0031 | 2.31E-09 | 627317 | 0.999116 |
| rs875868 | 1 | 65539253 | A | C | 0.5663 | 0.0124 | 0.0019 | 6.88E-11 | 626686 | 0.993017 |
| rs10518327 | 1 | 85487520 | A | G | 0.2898 | 0.0107 | 0.0019 | 2.31E-08 | 693127 | 0.952811 |
| rs598104 | 1 | 86266109 | A | T | 0.5944 | -0.0137 | 0.0018 | 5.37E-14 | 626434 | 0.998999 |
| rs6688233 | 1 | 9335745 | T | C | 0.2393 | 0.021 | 0.0021 | 9.64E-24 | 626909 | 0.982661 |
| rs114709597 | 1 | 9352704 | C | G | 0.7945 | 0.0173 | 0.0024 | 1.35E-12 | 484563 | 0.990622 |
| rs236319 | 1 | 94054382 | T | C | 0.3906 | 0.0105 | 0.0019 | 4.50E-08 | 579625 | 1 |
| rs3792142 | 2 | 100057208 | A | C | 0.4167 | -0.0099 | 0.0017 | 8.92E-09 | 694615 | 0.999547 |
| rs9678859 | 2 | 100288478 | A | G | 0.1816 | 0.0188 | 0.0023 | 7.97E-16 | 627274 | 0.99681 |
| rs7576541 | 2 | 111607401 | T | C | 0.68 | -0.0112 | 0.0019 | 5.22E-09 | 627108 | 0.999399 |
| rs113135335 | 2 | 111887754 | T | G | 0.9009 | 0.0251 | 0.0033 | 4.51E-14 | 484563 | 0.983776 |
| rs3761706 | 2 | 111899881 | A | G | 0.0734 | 0.0321 | 0.0037 | 1.70E-18 | 626748 | 0.997217 |
| rs58099056 | 2 | 112156865 | T | C | 0.5666 | -0.0146 | 0.0023 | 1.75E-10 | 484563 | 0.757545 |
| rs1345203 | 2 | 112253851 | T | C | 0.7829 | 0.0264 | 0.0024 | 2.23E-28 | 484563 | 0.99293 |
| rs72823412 | 2 | 112408424 | T | C | 0.9072 | -0.0197 | 0.0035 | 1.67E-08 | 484563 | 0.939979 |
| rs4372913 | 2 | 114517748 | A | G | 0.7875 | -0.0159 | 0.0022 | 2.65E-13 | 626669 | 0.996809 |
| rs4849294 | 2 | 114619997 | T | C | 0.6137 | 0.0134 | 0.0018 | 1.77E-13 | 627261 | 1 |
| rs6432188 | 2 | 11499032 | T | C | 0.2016 | 0.0143 | 0.0023 | 2.25E-10 | 693855 | 0.984825 |
| rs332105 | 2 | 119444229 | A | G | 0.5547 | -0.0137 | 0.0018 | 4.93E-14 | 624989 | 0.982559 |
| rs711869 | 2 | 13073967 | A | G | 0.5566 | -0.0186 | 0.0018 | 1.96E-24 | 627083 | 0.981668 |
| rs1563206 | 2 | 158348630 | T | C | 0.0953 | -0.0184 | 0.0032 | 5.72E-09 | 627225 | 0.991846 |
| rs1902032 | 2 | 158466273 | T | C | 0.9434 | -0.0235 | 0.0038 | 7.11E-10 | 668427 | 0.986377 |
| rs2444770 | 2 | 158503739 | T | C | 0.8453 | 0.0182 | 0.0025 | 1.78E-13 | 626805 | 0.996277 |
| rs114218039 | 2 | 158663086 | A | G | 0.0504 | -0.0261 | 0.0045 | 6.57E-09 | 484563 | 1 |
| rs6722159 | 2 | 161340112 | A | T | 0.4828 | -0.0099 | 0.0018 | 4.63E-08 | 614926 | 0.999654 |
| rs270960 | 2 | 161714729 | A | C | 0.663 | 0.0111 | 0.0018 | 9.04E-10 | 687116 | 0.998562 |
| rs4487103 | 2 | 165485978 | C | G | 0.6016 | 0.0123 | 0.002 | 7.28E-10 | 484563 | 0.99775 |
| rs10195252 | 2 | 165513091 | T | C | 0.5846 | 0.0324 | 0.0017 | 8.43E-78 | 693958 | 1 |
| rs115631825 | 2 | 165559101 | A | G | 0.8678 | -0.0385 | 0.0045 | 1.66E-17 | 546457 | 1 |
| rs355793 | 2 | 165658890 | A | T | 0.8411 | 0.0185 | 0.0024 | 4.54E-15 | 689365 | 0.988117 |
| rs355818 | 2 | 165707855 | A | G | 0.8904 | 0.0187 | 0.0027 | 8.81E-12 | 677494 | 0.996964 |
| rs7582206 | 2 | 165758909 | A | T | 0.4705 | -0.0167 | 0.002 | 1.56E-17 | 484563 | 0.993467 |
| rs6433219 | 2 | 171421125 | A | G | 0.2663 | 0.0162 | 0.002 | 2.84E-15 | 625531 | 0.997033 |
| rs7585974 | 2 | 172377212 | C | G | 0.1826 | 0.0187 | 0.0026 | 2.31E-13 | 484563 | 0.995766 |
| rs1569135 | 2 | 188115398 | A | G | 0.5373 | 0.0205 | 0.0017 | 2.99E-33 | 694469 | 0.996958 |
| rs56054257 | 2 | 188163521 | C | G | 0.044 | 0.0267 | 0.0049 | 4.02E-08 | 484563 | 0.974246 |
| rs78219026 | 2 | 191746233 | T | G | 0.9159 | 0.0203 | 0.0036 | 1.20E-08 | 484563 | 0.991537 |
| rs113386058 | 2 | 20143831 | A | T | 0.9716 | 0.0346 | 0.006 | 1.03E-08 | 484563 | 0.967929 |
| rs3732083 | 2 | 207041053 | T | C | 0.575 | -0.0114 | 0.0018 | 1.74E-10 | 627171 | 1 |
| rs1250259 | 2 | 216300482 | A | T | 0.7409 | -0.0164 | 0.002 | 1.04E-15 | 614270 | 0.993558 |
| rs6736742 | 2 | 218277149 | A | G | 0.3001 | 0.0111 | 0.002 | 1.45E-08 | 623327 | 0.993196 |
| rs2373078 | 2 | 218392555 | T | C | 0.0999 | 0.0215 | 0.0029 | 1.11E-13 | 627188 | 0.986517 |
| rs3731861 | 2 | 219191256 | T | C | 0.6307 | -0.0112 | 0.0019 | 1.40E-09 | 627073 | 0.995181 |
| rs78058190 | 2 | 219699999 | A | G | 0.0493 | 0.0357 | 0.0052 | 4.27E-12 | 484563 | 0.778797 |
| rs2602680 | 2 | 225066513 | T | G | 0.5455 | 0.0116 | 0.0018 | 1.01E-10 | 627099 | 0.997167 |
| rs1522811 | 2 | 226992454 | A | C | 0.226 | -0.0129 | 0.0021 | 1.64E-09 | 627151 | 0.98928 |
| rs3891424 | 2 | 239365456 | A | G | 0.0527 | -0.0252 | 0.0045 | 1.80E-08 | 484563 | 0.966238 |
| rs12466434 | 2 | 239674329 | T | C | 0.5538 | 0.0104 | 0.0019 | 2.41E-08 | 625390 | 0.953935 |
| rs146095395 | 2 | 239769885 | T | C | 0.18 | -0.02 | 0.0029 | 2.60E-12 | 484563 | 0.799913 |
| rs17509001 | 2 | 24021231 | T | C | 0.8424 | -0.0161 | 0.0025 | 5.14E-11 | 694609 | 0.9995 |
| rs7589318 | 2 | 25378372 | A | G | 0.3111 | -0.0162 | 0.0018 | 1.24E-18 | 694457 | 0.995739 |
| rs12999687 | 2 | 25512438 | T | G | 0.5162 | 0.0146 | 0.0017 | 3.07E-17 | 692962 | 0.977665 |
| rs10171315 | 2 | 25582791 | A | G | 0.0782 | 0.024 | 0.0032 | 5.58E-14 | 675110 | 0.996833 |
| rs13406302 | 2 | 37874850 | A | C | 0.7272 | -0.0135 | 0.0019 | 2.17E-12 | 692183 | 0.996115 |
| rs17511102 | 2 | 37960613 | A | T | 0.9184 | -0.02 | 0.0033 | 7.96E-10 | 611740 | 1 |
| rs10153926 | 2 | 43189120 | A | G | 0.2187 | 0.0134 | 0.0024 | 2.61E-08 | 484563 | 0.97686 |
| rs6752964 | 2 | 43754655 | T | C | 0.8967 | -0.0189 | 0.0028 | 1.66E-11 | 694429 | 0.995229 |
| rs17326656 | 2 | 48962291 | T | G | 0.2346 | 0.0153 | 0.0021 | 5.77E-13 | 627216 | 0.989881 |
| rs6717480 | 2 | 58210522 | A | C | 0.4075 | 0.0122 | 0.0018 | 1.57E-11 | 627161 | 0.99292 |
| rs7606002 | 2 | 58690167 | A | G | 0.834 | 0.0132 | 0.0024 | 2.84E-08 | 627128 | 0.99176 |
| rs13028903 | 2 | 59951465 | T | C | 0.4599 | 0.0104 | 0.0018 | 9.33E-09 | 626853 | 0.986178 |
| rs3856523 | 2 | 65969274 | A | G | 0.5762 | 0.0106 | 0.0018 | 5.43E-09 | 626889 | 0.997386 |
| rs11889877 | 2 | 66080617 | T | C | 0.1835 | 0.0149 | 0.0023 | 9.36E-11 | 627144 | 1 |
| rs2860971 | 2 | 66170689 | A | G | 0.3897 | 0.0113 | 0.0018 | 6.59E-10 | 627136 | 0.996975 |
| rs6719672 | 2 | 66234202 | A | G | 0.8196 | -0.0263 | 0.0023 | 1.67E-29 | 627231 | 0.993923 |
| rs11897119 | 2 | 66772000 | T | C | 0.5952 | -0.0142 | 0.0017 | 5.69E-16 | 692050 | 1 |
| rs2062204 | 2 | 67472545 | A | C | 0.2415 | 0.0158 | 0.0021 | 2.62E-14 | 627163 | 0.99115 |
| rs10170407 | 2 | 67725604 | T | C | 0.9103 | 0.0238 | 0.0031 | 1.68E-14 | 627020 | 0.990315 |
| rs12478439 | 2 | 67751114 | C | G | 0.843 | -0.0209 | 0.0024 | 6.79E-18 | 625085 | 0.998373 |
| rs7598832 | 2 | 67853013 | T | C | 0.3333 | -0.0183 | 0.0018 | 1.11E-24 | 694468 | 0.997101 |
| rs930653 | 2 | 68464271 | A | T | 0.3558 | 0.0112 | 0.0019 | 2.89E-09 | 627070 | 0.998172 |
| rs749458 | 2 | 96795857 | T | C | 0.1998 | -0.0126 | 0.0023 | 2.52E-08 | 626842 | 1 |
| rs11893688 | 2 | 9695282 | T | C | 0.6614 | 0.0138 | 0.0019 | 7.75E-13 | 592078 | 0.998474 |
| rs16853606 | 3 | 107270460 | A | G | 0.8412 | -0.0151 | 0.0023 | 1.25E-10 | 626887 | 1 |
| rs13092681 | 3 | 113290450 | T | C | 0.5731 | -0.011 | 0.002 | 3.54E-08 | 484563 | 0.999525 |
| rs2595004 | 3 | 11406721 | T | C | 0.8252 | -0.0156 | 0.0024 | 3.39E-11 | 627230 | 0.991141 |
| rs7630417 | 3 | 11530752 | T | C | 0.9525 | 0.0277 | 0.0046 | 1.71E-09 | 484563 | 0.994083 |
| rs17780995 | 3 | 11979818 | T | C | 0.196 | -0.014 | 0.0023 | 1.46E-09 | 609165 | 0.997034 |
| rs13068313 | 3 | 12262961 | A | G | 0.2516 | -0.0162 | 0.002 | 4.18E-16 | 682780 | 0.987946 |
| rs17036126 | 3 | 12287863 | T | C | 0.1189 | 0.0243 | 0.0026 | 2.82E-21 | 694411 | 0.994761 |
| rs12496583 | 3 | 123121286 | A | G | 0.7501 | -0.011 | 0.0019 | 1.57E-08 | 692137 | 0.99558 |
| rs12636454 | 3 | 12360214 | T | C | 0.7691 | -0.0111 | 0.002 | 2.03E-08 | 689409 | 0.998156 |
| rs1175381 | 3 | 12485844 | A | G | 0.9632 | 0.0374 | 0.0051 | 1.72E-13 | 608621 | 0.976796 |
| rs17819328 | 3 | 12489342 | T | G | 0.5677 | -0.0247 | 0.0017 | 4.79E-46 | 693372 | 0.990447 |
| rs6766666 | 3 | 12690855 | T | G | 0.1889 | -0.0205 | 0.0023 | 3.01E-19 | 627257 | 0.999205 |
| rs1979527 | 3 | 127400781 | A | C | 0.1989 | 0.0134 | 0.0022 | 2.36E-09 | 625840 | 0.989766 |
| rs11718898 | 3 | 12848822 | T | C | 0.3233 | 0.0157 | 0.0019 | 8.87E-17 | 626204 | 1 |
| rs62266885 | 3 | 128994633 | C | G | 0.9648 | 0.0431 | 0.0055 | 3.93E-15 | 484563 | 0.948136 |
| rs6765930 | 3 | 129020778 | A | G | 0.2087 | 0.0116 | 0.0021 | 2.62E-08 | 694431 | 0.991983 |
| rs72982655 | 3 | 129108915 | T | C | 0.1752 | 0.0192 | 0.0026 | 8.90E-14 | 484563 | 0.98721 |
| rs72628504 | 3 | 129287004 | A | G | 0.933 | -0.0388 | 0.0041 | 2.04E-21 | 484563 | 0.932268 |
| rs6795831 | 3 | 129341403 | A | C | 0.8144 | 0.0348 | 0.0023 | 4.91E-53 | 627216 | 0.984604 |
| rs1514025 | 3 | 129676582 | C | G | 0.9661 | 0.0291 | 0.0051 | 1.02E-08 | 610571 | 0.988438 |
| rs76074386 | 3 | 129677841 | A | G | 0.0846 | 0.0251 | 0.0036 | 2.43E-12 | 484563 | 0.979876 |
| rs28850104 | 3 | 129747349 | T | C | 0.2842 | -0.0177 | 0.0023 | 6.49E-15 | 484563 | 0.924133 |
| rs10935182 | 3 | 136137422 | A | G | 0.4285 | 0.0109 | 0.0018 | 1.42E-09 | 626927 | 0.9993 |
| rs34905952 | 3 | 138104635 | A | G | 0.1433 | 0.0209 | 0.0026 | 1.23E-15 | 535689 | 0.995385 |
| rs62271373 | 3 | 150066540 | A | T | 0.0578 | 0.0408 | 0.0043 | 3.44E-21 | 484563 | 0.957592 |
| rs10049088 | 3 | 156797648 | T | C | 0.3815 | -0.029 | 0.0018 | 1.45E-59 | 681080 | 0.995574 |
| rs998749 | 3 | 168972802 | A | G | 0.5024 | 0.0139 | 0.0017 | 6.23E-16 | 678637 | 0.991604 |
| rs9647379 | 3 | 171785168 | C | G | 0.3988 | -0.015 | 0.0019 | 5.14E-16 | 624516 | 0.9925 |
| rs4686696 | 3 | 185516520 | A | G | 0.3139 | 0.0152 | 0.0018 | 5.67E-17 | 693522 | 0.999926 |
| rs522833 | 3 | 187677380 | C | G | 0.7801 | -0.0127 | 0.0022 | 6.04E-09 | 624145 | 0.985577 |
| rs1286769 | 3 | 25585166 | A | G | 0.456 | -0.0098 | 0.0017 | 1.58E-08 | 688180 | 0.985524 |
| rs10212473 | 3 | 33872984 | A | G | 0.7573 | -0.0118 | 0.002 | 5.70E-09 | 627322 | 0.999518 |
| rs11129657 | 3 | 35636709 | T | C | 0.7925 | -0.0147 | 0.0022 | 2.31E-11 | 627182 | 0.989043 |
| rs174829 | 3 | 37535577 | A | G | 0.3523 | -0.014 | 0.0019 | 6.28E-13 | 591813 | 0.988313 |
| rs2507948 | 3 | 37832172 | A | G | 0.4507 | 0.0107 | 0.0018 | 3.45E-09 | 627130 | 0.982925 |
| rs740838 | 3 | 43764315 | A | C | 0.0248 | -0.0431 | 0.0058 | 8.98E-14 | 606754 | 0.998522 |
| rs9855938 | 3 | 46957298 | A | G | 0.6505 | 0.0154 | 0.0018 | 1.59E-17 | 693754 | 0.995975 |
| rs6446204 | 3 | 49034879 | T | C | 0.2435 | -0.0207 | 0.002 | 1.16E-24 | 694611 | 0.999587 |
| rs762705 | 3 | 50313527 | A | C | 0.8348 | -0.018 | 0.0027 | 3.62E-11 | 484563 | 0.948656 |
| rs112256201 | 3 | 50671766 | T | C | 0.0462 | -0.0257 | 0.0047 | 4.73E-08 | 484563 | 1 |
| rs4378999 | 3 | 51208646 | A | T | 0.8882 | -0.0178 | 0.0029 | 9.78E-10 | 626887 | 0.99799 |
| rs76382119 | 3 | 52106828 | A | G | 0.1725 | -0.0144 | 0.0026 | 4.46E-08 | 484563 | 0.972794 |
| rs2109558 | 3 | 52501616 | C | G | 0.9435 | 0.0383 | 0.0037 | 3.92E-25 | 678972 | 0.999129 |
| rs2276824 | 3 | 52637486 | C | G | 0.461 | 0.0222 | 0.0017 | 8.90E-38 | 693464 | 0.998659 |
| rs34341044 | 3 | 52637631 | T | C | 0.8721 | -0.0328 | 0.0049 | 1.51E-11 | 551172 | 0.993525 |
| rs7630325 | 3 | 53215799 | A | G | 0.1467 | -0.0176 | 0.0028 | 3.22E-10 | 484563 | 0.989899 |
| rs3736153 | 3 | 53262400 | C | G | 0.1953 | 0.017 | 0.0023 | 5.77E-14 | 623664 | 0.994704 |
| rs116511141 | 3 | 53455300 | A | G | 0.033 | -0.0316 | 0.0055 | 1.15E-08 | 484563 | 0.988805 |
| rs312487 | 3 | 53545622 | T | C | 0.489 | -0.0102 | 0.0018 | 2.06E-08 | 613039 | 0.973549 |
| rs17289049 | 3 | 57187036 | A | G | 0.9111 | -0.0191 | 0.003 | 2.03E-10 | 686026 | 0.990054 |
| rs73094710 | 3 | 61557775 | T | G | 0.896 | 0.0197 | 0.0032 | 1.20E-09 | 484563 | 0.992204 |
| rs11721258 | 3 | 64605119 | A | G | 0.7181 | -0.0118 | 0.0019 | 6.27E-10 | 689023 | 0.99575 |
| rs74388175 | 3 | 64629904 | T | C | 0.2075 | -0.0142 | 0.0025 | 1.02E-08 | 484563 | 0.957387 |
| rs6776363 | 3 | 64664769 | C | G | 0.3223 | -0.0137 | 0.002 | 3.26E-12 | 613880 | 0.946428 |
| rs11130981 | 3 | 64681767 | A | G | 0.0742 | 0.0227 | 0.0035 | 5.01E-11 | 610543 | 0.984508 |
| rs115463373 | 3 | 64693140 | A | G | 0.969 | 0.037 | 0.0057 | 8.41E-11 | 484563 | 1 |
| rs2371767 | 3 | 64718258 | C | G | 0.2703 | -0.0402 | 0.0019 | 1.00E-100 | 679069 | 0.995637 |
| rs2371762 | 3 | 64774688 | T | C | 0.9583 | 0.0282 | 0.0049 | 1.14E-08 | 484563 | 0.983852 |
| rs704447 | 3 | 64891099 | T | G | 0.5774 | 0.0142 | 0.0018 | 8.33E-15 | 626974 | 0.979042 |
| rs1442029 | 3 | 64898094 | T | C | 0.2241 | -0.0155 | 0.0021 | 3.10E-13 | 627020 | 0.994079 |
| rs7651129 | 3 | 66474954 | A | G | 0.9258 | 0.0189 | 0.0034 | 2.63E-08 | 627059 | 0.994824 |
| rs17311057 | 3 | 78841789 | T | C | 0.285 | 0.0145 | 0.002 | 1.72E-12 | 615028 | 0.997905 |
| rs12490079 | 3 | 99242097 | T | C | 0.7473 | -0.0118 | 0.002 | 3.55E-09 | 694344 | 0.994962 |
| rs73858966 | 3 | 99654183 | A | T | 0.0788 | 0.0244 | 0.0037 | 2.46E-11 | 484563 | 0.998054 |
| rs2270894 | 3 | 9975386 | C | G | 0.793 | -0.0137 | 0.0024 | 1.08E-08 | 591000 | 0.922584 |
| rs13107325 | 4 | 103188709 | T | C | 0.0808 | -0.0306 | 0.0034 | 3.69E-19 | 693873 | 1 |
| rs7680608 | 4 | 1050437 | C | G | 0.0738 | -0.0218 | 0.0035 | 6.24E-10 | 601061 | 0.982729 |
| rs56185013 | 4 | 106160133 | A | G | 0.2179 | -0.0168 | 0.0024 | 2.49E-12 | 484563 | 0.999202 |
| rs1511022 | 4 | 120126233 | T | C | 0.1232 | 0.0169 | 0.0027 | 6.40E-10 | 627153 | 0.996552 |
| rs7680787 | 4 | 122624065 | T | C | 0.6476 | 0.0138 | 0.0018 | 1.17E-14 | 693901 | 1 |
| rs7678199 | 4 | 123724239 | A | T | 0.7092 | 0.0121 | 0.002 | 1.01E-09 | 626913 | 0.988694 |
| rs10518406 | 4 | 123762897 | A | G | 0.9636 | 0.0247 | 0.0045 | 3.05E-08 | 679418 | 0.989422 |
| rs149921263 | 4 | 123923955 | A | G | 0.0367 | 0.0401 | 0.0053 | 4.27E-14 | 484563 | 0.977793 |
| rs303084 | 4 | 124066948 | A | G | 0.7843 | 0.0187 | 0.0021 | 2.29E-19 | 694504 | 0.989562 |
| rs11726981 | 4 | 125220708 | A | C | 0.724 | -0.0149 | 0.002 | 1.26E-13 | 627230 | 0.991933 |
| rs917681 | 4 | 13247539 | T | C | 0.2945 | 0.0115 | 0.0019 | 7.58E-10 | 694552 | 0.99252 |
| rs12639777 | 4 | 145425040 | T | G | 0.7168 | -0.0147 | 0.0022 | 1.75E-11 | 484563 | 0.996729 |
| rs75686861 | 4 | 145621328 | A | G | 0.0888 | 0.0288 | 0.0035 | 1.80E-16 | 484563 | 0.985174 |
| rs951252 | 4 | 145825151 | A | G | 0.4514 | -0.0185 | 0.0017 | 3.44E-27 | 694562 | 1 |
| rs4586926 | 4 | 15375527 | A | C | 0.6363 | 0.0119 | 0.0019 | 1.32E-10 | 627293 | 0.998192 |
| rs1425486 | 4 | 157683685 | T | C | 0.3506 | -0.0119 | 0.0018 | 2.91E-11 | 693348 | 0.996884 |
| rs4865444 | 4 | 1624125 | C | G | 0.6339 | -0.0113 | 0.0021 | 4.35E-08 | 484563 | 0.976418 |
| rs2333496 | 4 | 177609609 | T | C | 0.686 | 0.0114 | 0.0018 | 7.15E-10 | 692255 | 0.994871 |
| rs4558863 | 4 | 26079129 | T | C | 0.8104 | -0.021 | 0.0024 | 4.69E-19 | 627314 | 0.998678 |
| rs73245728 | 4 | 26206514 | A | C | 0.9378 | -0.0246 | 0.0041 | 2.58E-09 | 484563 | 0.980671 |
| rs6853254 | 4 | 26352363 | T | G | 0.349 | 0.0174 | 0.0019 | 4.02E-20 | 627201 | 0.996143 |
| rs3121419 | 4 | 3232257 | T | C | 0.2974 | -0.0111 | 0.0019 | 8.54E-09 | 627139 | 0.998584 |
| rs4450871 | 4 | 4990298 | A | G | 0.5506 | 0.0166 | 0.0019 | 3.23E-18 | 582854 | 1 |
| rs11722554 | 4 | 5016883 | A | G | 0.0377 | -0.0334 | 0.0049 | 1.32E-11 | 565482 | 1 |
| rs6446312 | 4 | 5019932 | C | G | 0.1894 | 0.0169 | 0.0025 | 1.57E-11 | 484563 | 0.996093 |
| rs10462028 | 4 | 56298300 | A | G | 0.3237 | 0.0189 | 0.0019 | 2.95E-23 | 627124 | 0.99994 |
| rs2602116 | 4 | 89666776 | A | G | 0.1826 | -0.0183 | 0.0023 | 3.62E-15 | 627238 | 0.998242 |
| rs2167750 | 4 | 89730074 | T | C | 0.4729 | 0.0274 | 0.0018 | 5.53E-53 | 627208 | 0.997953 |
| rs10024506 | 4 | 89764197 | C | G | 0.234 | -0.016 | 0.0021 | 2.69E-14 | 626517 | 0.996862 |
| rs11724804 | 4 | 965779 | A | G | 0.4436 | -0.017 | 0.0018 | 7.61E-21 | 624890 | 0.995671 |
| rs3114600 | 5 | 101955199 | T | C | 0.7194 | -0.0141 | 0.0019 | 3.83E-13 | 694498 | 0.995273 |
| rs17154889 | 5 | 102305065 | A | C | 0.3108 | -0.0158 | 0.0019 | 3.90E-16 | 627201 | 0.993996 |
| rs250302 | 5 | 118677340 | C | G | 0.7739 | -0.0178 | 0.0022 | 5.50E-16 | 623012 | 0.986466 |
| rs1045241 | 5 | 118729286 | T | C | 0.2817 | -0.0185 | 0.0019 | 3.51E-22 | 694273 | 0.990114 |
| rs17764730 | 5 | 127357526 | T | C | 0.2359 | -0.0162 | 0.0021 | 9.68E-15 | 627292 | 0.99116 |
| rs1029472 | 5 | 130867733 | A | G | 0.9407 | -0.0251 | 0.0042 | 1.74E-09 | 484563 | 0.997384 |
| rs11747001 | 5 | 132412299 | A | G | 0.7639 | 0.0172 | 0.002 | 7.73E-18 | 694337 | 0.998801 |
| rs6881034 | 5 | 134405482 | T | C | 0.7072 | -0.0136 | 0.002 | 7.91E-12 | 625587 | 0.956202 |
| rs10055995 | 5 | 137698299 | T | C | 0.5788 | -0.0109 | 0.0018 | 1.94E-09 | 627307 | 1 |
| rs254431 | 5 | 141769375 | A | G | 0.1275 | 0.0151 | 0.0026 | 9.82E-09 | 627230 | 0.971718 |
| rs17098725 | 5 | 141860124 | C | G | 0.4235 | -0.0118 | 0.0019 | 2.33E-10 | 613500 | 0.972527 |
| rs34000 | 5 | 141973501 | T | C | 0.6 | 0.014 | 0.0019 | 3.68E-14 | 604297 | 0.985019 |
| rs10477191 | 5 | 142077715 | A | G | 0.9358 | -0.0332 | 0.004 | 1.34E-16 | 484563 | 0.911492 |
| rs7712968 | 5 | 142086214 | T | C | 0.9162 | -0.0219 | 0.0032 | 5.05E-12 | 626824 | 0.991698 |
| rs10463416 | 5 | 148572915 | A | G | 0.3589 | 0.0137 | 0.0018 | 9.28E-14 | 627148 | 0.998324 |
| rs2343813 | 5 | 149878840 | T | C | 0.0841 | 0.0192 | 0.0031 | 8.62E-10 | 626418 | 0.994112 |
| rs17703354 | 5 | 157986357 | T | C | 0.9152 | -0.0202 | 0.0033 | 1.25E-09 | 626997 | 0.988307 |
| rs72846967 | 5 | 171849098 | A | G | 0.7032 | -0.0137 | 0.0022 | 2.14E-10 | 484563 | 0.999081 |
| rs12657303 | 5 | 172142551 | A | C | 0.3972 | -0.0114 | 0.0018 | 7.93E-10 | 626919 | 0.973199 |
| rs4868256 | 5 | 172748679 | T | C | 0.5006 | 0.0128 | 0.0019 | 1.16E-11 | 616777 | 0.906314 |
| rs755643 | 5 | 173136667 | A | G | 0.2882 | -0.0119 | 0.002 | 1.56E-09 | 627000 | 0.986999 |
| rs6861681 | 5 | 173362458 | A | G | 0.2918 | 0.0275 | 0.0019 | 4.18E-49 | 693674 | 1 |
| rs6556301 | 5 | 176527577 | T | G | 0.3706 | 0.0206 | 0.0018 | 2.03E-29 | 663437 | 0.983643 |
| rs12055154 | 5 | 176675423 | A | G | 0.9727 | 0.0322 | 0.0057 | 1.33E-08 | 625011 | 0.998176 |
| rs1445845 | 5 | 178507090 | A | G | 0.6523 | -0.0104 | 0.0019 | 3.55E-08 | 627178 | 1 |
| rs11746028 | 5 | 180645425 | T | C | 0.1813 | -0.0141 | 0.0024 | 2.00E-09 | 626925 | 1 |
| rs3792751 | 5 | 32773314 | T | C | 0.3642 | 0.0136 | 0.0018 | 1.41E-14 | 694315 | 0.987811 |
| rs299615 | 5 | 34042097 | A | G | 0.1518 | -0.0148 | 0.0025 | 1.71E-09 | 627105 | 1 |
| rs4293945 | 5 | 38736142 | A | T | 0.5926 | 0.0117 | 0.0018 | 1.69E-10 | 624922 | 0.994431 |
| rs11134029 | 5 | 4025324 | T | C | 0.5456 | 0.0143 | 0.002 | 5.72E-13 | 484563 | 0.994925 |
| rs6874524 | 5 | 53343529 | T | C | 0.7713 | 0.0131 | 0.0021 | 8.93E-10 | 627295 | 0.997685 |
| rs7721054 | 5 | 54890802 | T | C | 0.2948 | 0.0169 | 0.0019 | 1.56E-19 | 693843 | 0.989511 |
| rs3902134 | 5 | 55750805 | A | G | 0.2077 | -0.0118 | 0.0021 | 3.72E-08 | 694413 | 0.97131 |
| rs61653333 | 5 | 55779080 | T | G | 0.03 | 0.042 | 0.0058 | 6.56E-13 | 484563 | 0.97583 |
| rs459193 | 5 | 55806751 | A | G | 0.2494 | 0.0278 | 0.0019 | 3.99E-47 | 694515 | 1 |
| rs3936510 | 5 | 55860866 | T | G | 0.1969 | 0.0299 | 0.0022 | 4.62E-43 | 692459 | 1 |
| rs13186194 | 5 | 60795485 | T | C | 0.6166 | -0.0102 | 0.0018 | 7.02E-09 | 694288 | 0.997161 |
| rs6867518 | 5 | 64679283 | T | C | 0.8754 | -0.018 | 0.0027 | 1.93E-11 | 627200 | 0.987071 |
| rs2545401 | 5 | 66348255 | C | G | 0.3915 | 0.0103 | 0.0018 | 2.10E-08 | 627002 | 0.992734 |
| rs2227831 | 5 | 76023494 | A | G | 0.9533 | -0.0258 | 0.0043 | 1.65E-09 | 678751 | 0.995947 |
| rs4704389 | 5 | 76446103 | A | G | 0.4126 | 0.0123 | 0.0017 | 1.74E-12 | 692759 | 1 |
| rs7448322 | 5 | 77426083 | A | G | 0.2575 | 0.0114 | 0.002 | 2.71E-08 | 625042 | 0.996576 |
| rs188703 | 5 | 82834299 | A | G | 0.3694 | 0.0103 | 0.0018 | 2.57E-08 | 626905 | 1 |
| rs2503100 | 6 | 100613915 | A | G | 0.8345 | -0.0245 | 0.0024 | 4.86E-25 | 627198 | 0.998911 |
| rs11757455 | 6 | 112522852 | A | G | 0.0603 | -0.0221 | 0.0037 | 2.20E-09 | 620513 | 1 |
| rs9375417 | 6 | 126248374 | T | C | 0.4984 | -0.0126 | 0.002 | 1.87E-10 | 484563 | 0.998101 |
| rs6920788 | 6 | 126331988 | T | C | 0.7133 | 0.0149 | 0.002 | 4.78E-14 | 626550 | 0.997908 |
| rs9372833 | 6 | 126446699 | T | G | 0.6912 | 0.0132 | 0.0018 | 7.66E-13 | 694372 | 0.990256 |
| rs17753769 | 6 | 126588589 | A | G | 0.9683 | -0.0312 | 0.0056 | 3.10E-08 | 484563 | 1 |
| rs17054080 | 6 | 127109799 | A | G | 0.9449 | 0.0259 | 0.004 | 9.41E-11 | 612079 | 0.996942 |
| rs1361831 | 6 | 127181089 | T | C | 0.5465 | 0.016 | 0.0017 | 8.99E-21 | 694404 | 1 |
| rs115171655 | 6 | 127368128 | A | C | 0.0565 | 0.0249 | 0.0043 | 1.03E-08 | 484563 | 0.968999 |
| rs74762765 | 6 | 127444091 | T | C | 0.8569 | 0.0341 | 0.0055 | 4.63E-10 | 551631 | 1 |
| rs72959041 | 6 | 127454893 | A | G | 0.0417 | 0.1624 | 0.0044 | 2.08E-293 | 549629 | 0.969127 |
| rs77097175 | 6 | 127472589 | A | C | 0.0233 | 0.0464 | 0.006 | 7.17E-15 | 544013 | 1 |
| rs4318902 | 6 | 127521127 | A | G | 0.4294 | -0.0396 | 0.0017 | 1.98E-117 | 694415 | 0.992545 |
| rs9791192 | 6 | 127542953 | T | C | 0.1932 | -0.0145 | 0.0025 | 6.17E-09 | 484563 | 1 |
| rs79618076 | 6 | 127869047 | A | G | 0.9332 | -0.0292 | 0.004 | 2.26E-13 | 484563 | 0.979027 |
| rs75123935 | 6 | 127922536 | A | G | 0.9619 | -0.0364 | 0.0051 | 1.43E-12 | 484563 | 1 |
| rs9388766 | 6 | 130354855 | T | C | 0.3132 | -0.017 | 0.0019 | 3.96E-20 | 694603 | 0.994722 |
| rs4130903 | 6 | 130370168 | T | C | 0.086 | 0.0188 | 0.0032 | 3.64E-09 | 670568 | 0.947461 |
| rs3757298 | 6 | 132785016 | A | C | 0.8941 | -0.0205 | 0.003 | 6.06E-12 | 615027 | 0.998001 |
| rs799227 | 6 | 133487440 | C | G | 0.4675 | 0.0103 | 0.0018 | 1.01E-08 | 627259 | 0.994971 |
| rs6940715 | 6 | 133601845 | A | C | 0.8944 | 0.0222 | 0.003 | 6.11E-14 | 627196 | 0.996094 |
| rs634869 | 6 | 139831757 | T | C | 0.4193 | 0.0229 | 0.0017 | 2.60E-40 | 694613 | 0.999457 |
| rs6932767 | 6 | 14595873 | T | G | 0.7926 | 0.0154 | 0.0022 | 4.26E-12 | 627215 | 0.998656 |
| rs73005768 | 6 | 152021049 | A | G | 0.9574 | -0.0281 | 0.0049 | 8.04E-09 | 484563 | 1 |
| rs1281990 | 6 | 153490116 | T | C | 0.0474 | -0.026 | 0.0047 | 2.87E-08 | 484563 | 0.982624 |
| rs2745581 | 6 | 1598227 | A | G | 0.5869 | 0.0105 | 0.0019 | 1.70E-08 | 616559 | 1 |
| rs541091 | 6 | 160770552 | A | G | 0.4288 | -0.0199 | 0.0019 | 2.99E-26 | 545328 | 0.997066 |
| rs7744833 | 6 | 20581828 | A | G | 0.6823 | 0.0117 | 0.0019 | 1.42E-09 | 627233 | 0.986807 |
| rs2294823 | 6 | 22596987 | T | C | 0.3092 | 0.0116 | 0.0019 | 2.72E-09 | 626461 | 0.987775 |
| rs9379945 | 6 | 26907831 | T | C | 0.8508 | 0.0216 | 0.0028 | 5.58E-15 | 484563 | 0.99637 |
| rs9260405 | 6 | 29918887 | T | C | 0.4958 | -0.0123 | 0.002 | 4.51E-10 | 484563 | 0.999542 |
| rs10947038 | 6 | 29925134 | A | G | 0.0376 | 0.029 | 0.0047 | 8.10E-10 | 607752 | 0.997076 |
| rs2517600 | 6 | 30074163 | A | G | 0.2426 | -0.0146 | 0.0023 | 2.26E-10 | 484563 | 1 |
| rs9394007 | 6 | 30452962 | T | G | 0.6257 | -0.0142 | 0.002 | 3.81E-12 | 484563 | 0.989045 |
| rs142090224 | 6 | 30783487 | T | C | 0.0941 | 0.0204 | 0.0035 | 5.52E-09 | 484563 | 0.909135 |
| rs2517456 | 6 | 31059825 | A | T | 0.9835 | -0.0503 | 0.0069 | 2.99E-13 | 592429 | 1 |
| rs2233955 | 6 | 31081251 | A | G | 0.1816 | 0.0161 | 0.0024 | 2.04E-11 | 535550 | 1 |
| rs2844624 | 6 | 31232247 | T | C | 0.6408 | 0.0113 | 0.0021 | 3.87E-08 | 484563 | 0.998809 |
| rs6457374 | 6 | 31272261 | T | C | 0.6865 | 0.0274 | 0.0019 | 4.52E-47 | 691561 | 1 |
| rs6457402 | 6 | 31334864 | T | G | 0.194 | 0.0175 | 0.0022 | 1.43E-15 | 614166 | 0.999984 |
| rs62395355 | 6 | 31476297 | A | C | 0.1756 | 0.0241 | 0.0026 | 1.25E-20 | 484563 | 0.998395 |
| rs2516480 | 6 | 31496791 | C | G | 0.8668 | -0.017 | 0.0026 | 1.09E-10 | 625834 | 0.999749 |
| rs2857702 | 6 | 31577283 | C | G | 0.2052 | 0.0199 | 0.0025 | 5.74E-16 | 484563 | 0.983344 |
| rs6457536 | 6 | 32273765 | A | G | 0.777 | 0.0207 | 0.002 | 3.36E-24 | 693196 | 1 |
| rs3806156 | 6 | 32373698 | T | G | 0.3672 | 0.0172 | 0.0018 | 1.55E-22 | 693190 | 1 |
| rs9391786 | 6 | 32448561 | A | G | 0.5708 | -0.0233 | 0.0019 | 9.24E-35 | 625786 | 0.905883 |
| rs148967059 | 6 | 32454755 | A | T | 0.2046 | 0.0231 | 0.0027 | 4.41E-18 | 484563 | 0.812733 |
| rs113664977 | 6 | 32471008 | C | G | 0.8488 | 0.0229 | 0.0039 | 3.41E-09 | 484563 | 0.50081 |
| rs185320691 | 6 | 32490292 | C | G | 0.1013 | 0.0282 | 0.0036 | 6.85E-15 | 484563 | 0.804841 |
| rs73727915 | 6 | 32497046 | T | C | 0.0774 | -0.035 | 0.0054 | 7.65E-11 | 484563 | 0.453611 |
| rs145244672 | 6 | 32556461 | T | G | 0.8807 | -0.0242 | 0.0034 | 7.87E-13 | 484563 | 0.802551 |
| rs80163833 | 6 | 32559444 | T | C | 0.2695 | 0.018 | 0.0024 | 5.51E-14 | 484563 | 0.848851 |
| rs113315602 | 6 | 32574575 | A | C | 0.907 | -0.0251 | 0.0036 | 2.78E-12 | 484563 | 0.887409 |
| rs9273453 | 6 | 32627812 | C | G | 0.8767 | 0.0327 | 0.0032 | 2.53E-24 | 484563 | 0.86597 |
| rs200160235 | 6 | 32628070 | T | C | 0.9414 | 0.0469 | 0.006 | 4.68E-15 | 484563 | 0.497011 |
| rs41263825 | 6 | 32632543 | A | G | 0.1703 | 0.0241 | 0.0031 | 8.43E-15 | 484563 | 0.712078 |
| rs3998154 | 6 | 32685380 | A | G | 0.414 | 0.0124 | 0.002 | 7.33E-10 | 484563 | 0.983318 |
| rs4148878 | 6 | 32822186 | T | G | 0.9302 | -0.0201 | 0.0035 | 8.56E-09 | 677639 | 1 |
| rs11756897 | 6 | 32899139 | A | G | 0.7732 | -0.0126 | 0.0022 | 4.44E-09 | 625296 | 1 |
| rs411136 | 6 | 33408542 | A | G | 0.4121 | -0.0116 | 0.0017 | 3.96E-11 | 692102 | 0.999516 |
| rs12206376 | 6 | 34048416 | A | G | 0.2557 | 0.0125 | 0.0023 | 2.74E-08 | 484563 | 1 |
| rs114760566 | 6 | 34192036 | A | C | 0.0432 | 0.0932 | 0.0049 | 9.03E-82 | 484563 | 0.99322 |
| rs6691 | 6 | 34252352 | T | C | 0.4911 | 0.0103 | 0.0018 | 1.21E-08 | 625421 | 0.983111 |
| rs4711390 | 6 | 34286424 | T | C | 0.0377 | -0.0277 | 0.0042 | 3.80E-11 | 675081 | 0.99866 |
| rs2814998 | 6 | 34625211 | T | C | 0.2092 | -0.0173 | 0.0021 | 4.89E-17 | 693233 | 0.999468 |
| rs9469847 | 6 | 34658003 | T | C | 0.8487 | -0.0155 | 0.0027 | 1.80E-08 | 484563 | 0.991712 |
| rs2395628 | 6 | 35473252 | T | C | 0.307 | 0.011 | 0.002 | 1.73E-08 | 626075 | 0.992965 |
| rs13198178 | 6 | 41702227 | C | G | 0.0641 | 0.0308 | 0.0037 | 1.16E-16 | 614209 | 0.993145 |
| rs11756454 | 6 | 41798578 | A | T | 0.4406 | -0.0101 | 0.0018 | 1.61E-08 | 627070 | 0.999342 |
| rs16896344 | 6 | 43172430 | A | G | 0.1071 | 0.0175 | 0.0029 | 1.90E-09 | 627227 | 0.997982 |
| rs9369409 | 6 | 43346462 | C | G | 0.4153 | 0.0154 | 0.002 | 9.37E-15 | 484563 | 0.99934 |
| rs12204413 | 6 | 43711366 | A | C | 0.9094 | -0.0245 | 0.0035 | 1.96E-12 | 484563 | 0.972172 |
| rs3024987 | 6 | 43740840 | T | C | 0.126 | 0.0223 | 0.003 | 5.58E-14 | 484563 | 0.994576 |
| rs10434 | 6 | 43753212 | A | G | 0.451 | -0.0171 | 0.0019 | 1.24E-18 | 550475 | 0.967224 |
| rs998584 | 6 | 43757896 | A | C | 0.4799 | 0.0487 | 0.0017 | 1.22E-170 | 674183 | 0.994408 |
| rs2396086 | 6 | 43781528 | T | C | 0.1281 | -0.0207 | 0.0026 | 1.83E-15 | 647906 | 0.993049 |
| rs35349911 | 6 | 43785255 | T | C | 0.5871 | 0.0179 | 0.002 | 1.27E-18 | 484563 | 0.967221 |
| rs72862280 | 6 | 43793092 | T | C | 0.0256 | -0.038 | 0.0065 | 3.90E-09 | 484563 | 0.937583 |
| rs1317983 | 6 | 43806335 | T | C | 0.3117 | -0.0247 | 0.0021 | 1.58E-31 | 484563 | 0.997257 |
| rs1934328 | 6 | 45465753 | A | T | 0.5223 | -0.0101 | 0.0018 | 2.71E-08 | 626150 | 0.987726 |
| rs1294410 | 6 | 6738752 | T | C | 0.3825 | -0.0309 | 0.0018 | 2.26E-69 | 694393 | 0.985914 |
| rs115623524 | 6 | 6766378 | T | C | 0.0963 | 0.02 | 0.0034 | 4.60E-09 | 484563 | 0.960067 |
| rs35688039 | 6 | 6766704 | A | G | 0.1767 | 0.0168 | 0.0026 | 1.42E-10 | 484563 | 0.974586 |
| rs6921610 | 6 | 6997161 | T | C | 0.5257 | 0.0102 | 0.0018 | 1.14E-08 | 626770 | 1 |
| rs1285884 | 6 | 7143075 | T | C | 0.8776 | 0.0151 | 0.0028 | 4.88E-08 | 615461 | 0.987907 |
| rs1334576 | 6 | 7211818 | A | G | 0.4254 | -0.0171 | 0.0018 | 3.46E-21 | 627236 | 1 |
| rs6912381 | 6 | 80592356 | A | G | 0.325 | -0.0132 | 0.0021 | 4.40E-10 | 484563 | 0.991318 |
| rs12662365 | 6 | 80905389 | A | T | 0.3087 | -0.0122 | 0.0019 | 2.22E-10 | 627080 | 0.989722 |
| rs12527712 | 6 | 80916967 | T | C | 0.0881 | 0.0339 | 0.0033 | 6.85E-25 | 627314 | 0.999789 |
| rs62406467 | 6 | 81114126 | T | G | 0.1455 | -0.0188 | 0.0029 | 4.34E-11 | 484563 | 0.95179 |
| rs625136 | 6 | 81209025 | A | G | 0.9121 | -0.02 | 0.0032 | 7.38E-10 | 627179 | 0.998615 |
| rs1902066 | 6 | 81346033 | T | C | 0.4452 | -0.018 | 0.0018 | 2.38E-23 | 627242 | 0.990948 |
| rs79904243 | 6 | 81444898 | T | G | 0.9711 | 0.0422 | 0.006 | 2.20E-12 | 484563 | 0.958226 |
| rs72895340 | 6 | 82297307 | A | G | 0.0501 | 0.0298 | 0.0046 | 7.12E-11 | 484563 | 0.975668 |
| rs1010437 | 6 | 85098237 | A | G | 0.3346 | -0.0129 | 0.0018 | 1.04E-12 | 694469 | 0.994829 |
| rs13193461 | 6 | 85211666 | T | C | 0.1666 | 0.0153 | 0.0024 | 2.80E-10 | 626911 | 0.976353 |
| rs9341990 | 6 | 85391568 | A | G | 0.4131 | 0.0151 | 0.0018 | 9.46E-17 | 627285 | 0.997719 |
| rs16875501 | 6 | 85721376 | T | C | 0.0643 | 0.0236 | 0.0036 | 9.79E-11 | 619164 | 0.997209 |
| rs367026 | 7 | 101728638 | T | C | 0.6144 | -0.0133 | 0.002 | 7.28E-11 | 484563 | 0.976745 |
| rs1144 | 7 | 104756355 | T | C | 0.6642 | -0.015 | 0.0018 | 5.57E-17 | 688248 | 0.996493 |
| rs62487041 | 7 | 105334443 | T | G | 0.3882 | 0.0116 | 0.002 | 1.18E-08 | 484563 | 0.988998 |
| rs2237687 | 7 | 107585473 | T | C | 0.5161 | -0.0105 | 0.0019 | 1.67E-08 | 613400 | 0.963947 |
| rs11770285 | 7 | 107616011 | C | G | 0.9006 | 0.0309 | 0.0031 | 7.06E-24 | 627284 | 0.997863 |
| rs38902 | 7 | 116889718 | A | G | 0.5367 | 0.0138 | 0.0018 | 1.60E-14 | 627201 | 0.992334 |
| rs10249651 | 7 | 117064625 | T | C | 0.6269 | -0.0147 | 0.0018 | 1.22E-16 | 694556 | 0.996415 |
| rs2222543 | 7 | 120902185 | C | G | 0.3761 | -0.0147 | 0.0018 | 1.62E-15 | 624476 | 0.999547 |
| rs7776617 | 7 | 121065112 | A | T | 0.746 | 0.0128 | 0.002 | 4.37E-10 | 627268 | 0.998859 |
| rs2268382 | 7 | 130027037 | A | C | 0.6611 | 0.0105 | 0.0019 | 3.28E-08 | 627035 | 1 |
| rs1364422 | 7 | 130445981 | T | C | 0.2684 | 0.0157 | 0.0019 | 1.07E-16 | 694615 | 0.993429 |
| rs10258495 | 7 | 135248558 | T | C | 0.3824 | -0.0114 | 0.002 | 2.29E-08 | 484563 | 0.990116 |
| rs357438 | 7 | 138023288 | C | G | 0.3522 | -0.0114 | 0.0019 | 3.79E-09 | 612429 | 0.993367 |
| rs2158828 | 7 | 14227425 | A | G | 0.8271 | -0.0135 | 0.0023 | 3.42E-09 | 665297 | 0.991572 |
| rs73084574 | 7 | 20344126 | A | G | 0.0727 | -0.0211 | 0.0038 | 2.86E-08 | 484563 | 1 |
| rs3807947 | 7 | 20424889 | T | G | 0.6058 | 0.0137 | 0.0018 | 7.54E-14 | 627221 | 1 |
| rs974340 | 7 | 25559106 | T | C | 0.2387 | -0.0121 | 0.0022 | 2.14E-08 | 626563 | 0.972345 |
| rs7798002 | 7 | 25860580 | T | G | 0.1969 | 0.0319 | 0.0021 | 4.23E-50 | 694434 | 0.99546 |
| rs12672425 | 7 | 25939723 | T | G | 0.1851 | 0.0195 | 0.0022 | 1.10E-18 | 693958 | 0.976441 |
| rs1534696 | 7 | 26397239 | A | C | 0.5687 | -0.0244 | 0.0017 | 3.15E-44 | 682757 | 1 |
| rs113269332 | 7 | 26740757 | A | G | 0.0361 | 0.0318 | 0.0054 | 4.68E-09 | 484563 | 0.951323 |
| rs17501007 | 7 | 27206019 | A | T | 0.7908 | 0.0217 | 0.0026 | 5.62E-17 | 484563 | 0.874249 |
| rs7801581 | 7 | 27223771 | T | C | 0.2535 | 0.0225 | 0.002 | 1.20E-28 | 679778 | 0.96126 |
| rs17437657 | 7 | 27249617 | A | G | 0.0867 | -0.0372 | 0.0033 | 2.07E-29 | 620671 | 0.99224 |
| rs142106866 | 7 | 27293363 | T | C | 0.0242 | 0.0413 | 0.0064 | 1.54E-10 | 484563 | 0.989935 |
| rs1513272 | 7 | 28200097 | T | C | 0.4361 | -0.0198 | 0.0019 | 1.25E-26 | 551845 | 0.999004 |
| rs12112380 | 7 | 30935900 | T | C | 0.9433 | -0.0214 | 0.0038 | 1.70E-08 | 690575 | 0.979198 |
| rs7811609 | 7 | 32930597 | T | C | 0.3742 | -0.0121 | 0.0018 | 4.91E-11 | 627180 | 0.999362 |
| rs13223034 | 7 | 42701627 | T | C | 0.3174 | -0.0133 | 0.0019 | 3.67E-12 | 627108 | 0.998989 |
| rs3110697 | 7 | 45955029 | A | G | 0.4348 | 0.0123 | 0.0018 | 1.08E-11 | 626435 | 1 |
| rs2057869 | 7 | 46572992 | A | G | 0.3009 | 0.0119 | 0.002 | 2.06E-09 | 605289 | 0.993134 |
| rs2529411 | 7 | 50738482 | C | G | 0.6354 | -0.0114 | 0.0019 | 2.83E-09 | 591750 | 0.995441 |
| rs7797307 | 7 | 68686127 | C | G | 0.0598 | -0.0218 | 0.004 | 4.73E-08 | 614622 | 0.979572 |
| rs17145717 | 7 | 72929608 | A | G | 0.0621 | 0.027 | 0.0038 | 1.24E-12 | 626697 | 0.979328 |
| rs55747707 | 7 | 73037366 | A | G | 0.1783 | -0.0244 | 0.0024 | 3.05E-25 | 551855 | 0.995534 |
| rs3108415 | 7 | 76876982 | A | C | 0.7349 | 0.0131 | 0.0022 | 4.09E-09 | 484563 | 0.993527 |
| rs10264590 | 7 | 77357748 | A | G | 0.4795 | 0.0175 | 0.0018 | 8.32E-23 | 625011 | 0.998754 |
| rs917191 | 7 | 80570871 | C | G | 0.5706 | 0.0138 | 0.0018 | 5.11E-14 | 626903 | 0.980862 |
| rs13232789 | 7 | 84554998 | T | G | 0.6614 | -0.0129 | 0.0019 | 1.10E-11 | 627142 | 0.99839 |
| rs2023772 | 7 | 93131162 | A | C | 0.5115 | 0.0115 | 0.0017 | 1.42E-11 | 692196 | 0.998064 |
| rs9657541 | 8 | 10643164 | T | C | 0.1974 | 0.0163 | 0.0022 | 3.22E-14 | 694413 | 1 |
| rs55874847 | 8 | 11773913 | T | C | 0.3489 | -0.0118 | 0.0021 | 1.19E-08 | 484563 | 0.995827 |
| rs7003062 | 8 | 121358527 | T | C | 0.6051 | -0.0124 | 0.0018 | 2.06E-11 | 614969 | 0.994436 |
| rs4474021 | 8 | 12632903 | T | G | 0.3076 | 0.0118 | 0.0019 | 2.44E-10 | 679364 | 1 |
| rs144435165 | 8 | 126419938 | C | G | 0.0529 | 0.0431 | 0.005 | 5.61E-18 | 484563 | 0.782128 |
| rs10808546 | 8 | 126495818 | T | C | 0.422 | -0.0159 | 0.0017 | 1.98E-20 | 694436 | 0.990671 |
| rs4545126 | 8 | 126525035 | A | G | 0.2187 | 0.0132 | 0.0022 | 1.11E-09 | 693634 | 0.971347 |
| rs191785584 | 8 | 128183871 | A | G | 0.9728 | -0.034 | 0.0062 | 3.96E-08 | 484563 | 0.963221 |
| rs13256367 | 8 | 128334900 | A | C | 0.6535 | 0.0182 | 0.0019 | 6.15E-22 | 627055 | 0.997213 |
| rs2060145 | 8 | 129807905 | T | C | 0.289 | 0.0116 | 0.002 | 3.94E-09 | 627248 | 0.998081 |
| rs10100533 | 8 | 135706219 | A | G | 0.6217 | 0.0143 | 0.0018 | 8.43E-15 | 627307 | 0.996823 |
| rs15285 | 8 | 19824667 | T | C | 0.2636 | -0.0107 | 0.002 | 4.41E-08 | 685566 | 1 |
| rs9644033 | 8 | 23610639 | A | T | 0.7545 | 0.0222 | 0.0021 | 3.40E-26 | 626939 | 0.997191 |
| rs6557715 | 8 | 23807336 | T | G | 0.1392 | 0.0149 | 0.0027 | 3.43E-08 | 602625 | 0.997 |
| rs11786566 | 8 | 23884506 | T | G | 0.546 | -0.0109 | 0.0018 | 2.77E-09 | 620250 | 0.983579 |
| rs2012485 | 8 | 25374910 | T | C | 0.0454 | -0.0293 | 0.0044 | 2.67E-11 | 674509 | 0.998089 |
| rs11992444 | 8 | 25464690 | T | G | 0.5085 | 0.0233 | 0.002 | 5.57E-32 | 484563 | 1 |
| rs13281474 | 8 | 25664334 | T | G | 0.2857 | -0.0136 | 0.0022 | 6.15E-10 | 484563 | 0.987374 |
| rs13257034 | 8 | 25774337 | T | C | 0.637 | 0.0146 | 0.0019 | 3.20E-15 | 627159 | 0.998917 |
| rs1485745 | 8 | 25891545 | T | G | 0.5131 | 0.0118 | 0.0018 | 4.84E-11 | 626997 | 0.991067 |
| rs2874670 | 8 | 26245658 | A | G | 0.4437 | 0.0102 | 0.0018 | 2.05E-08 | 627217 | 0.985181 |
| rs12543555 | 8 | 69562014 | A | G | 0.8088 | -0.0138 | 0.0022 | 1.79E-10 | 694376 | 0.983153 |
| rs3098872 | 8 | 71932204 | T | G | 0.5924 | -0.0119 | 0.0018 | 4.72E-11 | 627306 | 0.999723 |
| rs3887553 | 8 | 71966458 | A | G | 0.0959 | -0.017 | 0.003 | 1.72E-08 | 627310 | 0.989891 |
| rs16937456 | 8 | 72077955 | A | G | 0.8718 | -0.0155 | 0.0027 | 1.08E-08 | 627035 | 0.990604 |
| rs146706620 | 8 | 72388360 | T | C | 0.0221 | 0.0458 | 0.0069 | 2.79E-11 | 484563 | 0.953235 |
| rs4738141 | 8 | 72469742 | A | G | 0.7433 | -0.0262 | 0.002 | 9.58E-41 | 661288 | 0.991122 |
| rs1455813 | 8 | 75995961 | A | G | 0.7157 | -0.0117 | 0.002 | 2.27E-09 | 627227 | 0.997468 |
| rs7827182 | 8 | 8380471 | C | G | 0.4407 | -0.0166 | 0.0019 | 3.92E-19 | 567657 | 0.999162 |
| rs7828497 | 8 | 89339721 | T | G | 0.7308 | -0.0172 | 0.0022 | 1.31E-14 | 484563 | 0.992448 |
| rs10087445 | 8 | 98936178 | T | C | 0.1669 | -0.013 | 0.0024 | 3.75E-08 | 626673 | 0.997651 |
| rs4743305 | 9 | 101760026 | T | C | 0.6196 | 0.0112 | 0.0018 | 1.08E-09 | 627116 | 0.993794 |
| rs71511786 | 9 | 107578059 | A | T | 0.9207 | 0.0228 | 0.0037 | 5.18E-10 | 484563 | 0.989553 |
| rs1800978 | 9 | 107665978 | C | G | 0.8788 | 0.0262 | 0.0026 | 2.62E-23 | 688307 | 0.989117 |
| rs10820747 | 9 | 107686823 | A | G | 0.2404 | 0.0271 | 0.0023 | 1.10E-31 | 484563 | 0.995642 |
| rs10124755 | 9 | 107692242 | T | C | 0.6499 | -0.013 | 0.0021 | 4.64E-10 | 484563 | 0.97737 |
| rs12006440 | 9 | 107703337 | T | C | 0.0316 | -0.039 | 0.0057 | 9.43E-12 | 484563 | 0.972661 |
| rs4743775 | 9 | 107727489 | A | G | 0.7639 | 0.0167 | 0.0021 | 8.10E-15 | 627005 | 0.985799 |
| rs10991444 | 9 | 107748492 | A | T | 0.8275 | -0.0191 | 0.0026 | 3.94E-13 | 484563 | 0.984418 |
| rs2937371 | 9 | 107826753 | A | T | 0.5664 | -0.0146 | 0.0019 | 6.03E-15 | 611824 | 0.966992 |
| rs9792666 | 9 | 107887738 | A | G | 0.9603 | 0.0503 | 0.0047 | 3.92E-27 | 614388 | 0.969295 |
| rs11788299 | 9 | 107900842 | A | T | 0.0681 | -0.0234 | 0.0039 | 2.23E-09 | 593667 | 0.92216 |
| rs12684047 | 9 | 111972671 | A | T | 0.1914 | -0.0154 | 0.0024 | 6.85E-11 | 626144 | 0.960641 |
| rs7020604 | 9 | 112583554 | A | G | 0.6653 | 0.0129 | 0.0021 | 7.04E-10 | 484563 | 0.99457 |
| rs150841499 | 9 | 112762434 | T | C | 0.1085 | -0.0201 | 0.0032 | 4.11E-10 | 484563 | 0.974827 |
| rs4978959 | 9 | 113597938 | T | C | 0.3016 | 0.0107 | 0.0019 | 3.27E-08 | 626881 | 0.999379 |
| rs10980797 | 9 | 113912553 | A | G | 0.5194 | -0.0182 | 0.002 | 4.52E-20 | 484563 | 0.993975 |
| rs10817896 | 9 | 119232655 | T | C | 0.2768 | 0.0126 | 0.002 | 3.30E-10 | 626971 | 0.991428 |
| rs6478851 | 9 | 131561110 | A | G | 0.25 | 0.0123 | 0.002 | 1.75E-09 | 627165 | 1 |
| rs12554943 | 9 | 137541413 | T | C | 0.8727 | 0.0172 | 0.003 | 6.59E-09 | 484563 | 1 |
| rs28562046 | 9 | 139241595 | C | G | 0.2431 | -0.0126 | 0.0023 | 4.53E-08 | 484563 | 0.997294 |
| rs10963067 | 9 | 17367946 | A | C | 0.0941 | -0.0209 | 0.0032 | 5.86E-11 | 627315 | 0.996294 |
| rs3731246 | 9 | 21971989 | C | G | 0.8986 | -0.016 | 0.0028 | 1.05E-08 | 692623 | 0.992369 |
| rs16907277 | 9 | 93972658 | A | G | 0.8906 | -0.0228 | 0.0029 | 9.08E-15 | 627291 | 0.995757 |
| rs10992414 | 9 | 95448381 | A | C | 0.8256 | -0.0223 | 0.0023 | 1.10E-21 | 627301 | 0.999143 |
| rs2398893 | 9 | 96758342 | A | G | 0.7142 | 0.0153 | 0.0019 | 6.75E-16 | 694566 | 0.999774 |
| rs7854560 | 9 | 98382950 | T | C | 0.2902 | 0.0116 | 0.0019 | 5.85E-10 | 694360 | 1 |
| rs28408682 | 10 | 104403310 | A | G | 0.3562 | -0.0122 | 0.0019 | 1.82E-10 | 547577 | 0.998983 |
| rs4073980 | 10 | 114746580 | C | G | 0.474 | -0.0196 | 0.0019 | 1.53E-25 | 551821 | 0.980275 |
| rs4751628 | 10 | 119315825 | T | G | 0.6281 | -0.0133 | 0.0018 | 6.16E-13 | 627235 | 0.99356 |
| rs2254069 | 10 | 122875589 | A | G | 0.1283 | 0.0248 | 0.0027 | 2.51E-20 | 623807 | 1 |
| rs11199781 | 10 | 122882194 | A | T | 0.8479 | 0.0165 | 0.0025 | 4.68E-11 | 613293 | 0.97944 |
| rs3943077 | 10 | 122945086 | A | G | 0.2693 | -0.0118 | 0.002 | 2.75E-09 | 626860 | 0.995897 |
| rs7913390 | 10 | 24906143 | T | C | 0.309 | 0.0118 | 0.0021 | 3.65E-08 | 484563 | 0.997856 |
| rs1494204 | 10 | 27904321 | T | C | 0.419 | -0.0147 | 0.0018 | 8.06E-16 | 626936 | 0.991012 |
| rs12770117 | 10 | 30360965 | A | G | 0.9356 | 0.0215 | 0.0038 | 2.00E-08 | 614991 | 0.978445 |
| rs2907794 | 10 | 32391109 | A | G | 0.2546 | -0.0113 | 0.002 | 1.25E-08 | 694569 | 0.996855 |
| rs11592754 | 10 | 32619572 | A | C | 0.8592 | -0.0215 | 0.0025 | 3.22E-18 | 694498 | 0.996712 |
| rs2506133 | 10 | 33464630 | A | T | 0.7272 | 0.0134 | 0.0022 | 1.69E-09 | 484563 | 0.990062 |
| rs10827226 | 10 | 33559319 | T | C | 0.1417 | 0.0198 | 0.0029 | 4.80E-12 | 484563 | 0.977712 |
| rs1474921 | 10 | 34165699 | A | G | 0.3852 | 0.0155 | 0.0019 | 1.97E-16 | 614829 | 0.969398 |
| rs7086377 | 10 | 3596526 | T | G | 0.3952 | 0.0119 | 0.0018 | 6.88E-11 | 627258 | 0.997261 |
| rs12774134 | 10 | 4963327 | T | C | 0.1186 | -0.0188 | 0.0027 | 1.99E-12 | 681187 | 1 |
| rs7350438 | 10 | 5643511 | T | C | 0.3763 | -0.0116 | 0.0019 | 3.63E-10 | 626977 | 0.990482 |
| rs7919055 | 10 | 63808475 | T | C | 0.9579 | -0.028 | 0.0046 | 1.04E-09 | 687680 | 0.971832 |
| rs10761602 | 10 | 63813802 | T | G | 0.5553 | -0.0117 | 0.0018 | 1.35E-10 | 625403 | 0.984928 |
| rs7070749 | 10 | 63882682 | A | G | 0.5531 | 0.0136 | 0.002 | 9.10E-12 | 484563 | 0.995798 |
| rs9415106 | 10 | 77295957 | A | G | 0.7138 | 0.0142 | 0.002 | 9.78E-13 | 627049 | 1 |
| rs6480914 | 10 | 80772591 | A | G | 0.5844 | 0.0133 | 0.002 | 3.21E-11 | 484563 | 0.991411 |
| rs779671 | 10 | 80897515 | A | G | 0.4717 | 0.0154 | 0.002 | 1.37E-14 | 484563 | 0.968601 |
| rs780159 | 10 | 80907147 | A | G | 0.4287 | -0.0163 | 0.0018 | 3.33E-20 | 686401 | 0.969682 |
| rs2802364 | 10 | 80960486 | C | G | 0.4868 | 0.012 | 0.0018 | 2.74E-11 | 626599 | 0.988132 |
| rs10887759 | 10 | 89603424 | A | G | 0.1595 | 0.0173 | 0.0024 | 1.06E-12 | 694465 | 0.988031 |
| rs11186914 | 10 | 94062631 | A | T | 0.5732 | 0.0104 | 0.0018 | 9.20E-09 | 627212 | 0.998419 |
| rs1111875 | 10 | 94462882 | T | C | 0.4179 | -0.0112 | 0.0017 | 8.72E-11 | 694419 | 1 |
| rs11187537 | 10 | 95346805 | C | G | 0.2633 | 0.0163 | 0.0021 | 2.43E-15 | 610077 | 0.9976 |
| rs1223583 | 10 | 95756500 | A | G | 0.5204 | -0.0112 | 0.0017 | 5.89E-11 | 694208 | 0.997391 |
| rs17417407 | 10 | 95931087 | T | G | 0.1705 | 0.0138 | 0.0023 | 1.31E-09 | 684754 | 1 |
| rs12419064 | 11 | 10320608 | A | G | 0.461 | -0.0141 | 0.0019 | 4.53E-14 | 551840 | 0.997454 |
| rs2957658 | 11 | 10393468 | A | G | 0.4913 | -0.0127 | 0.002 | 1.46E-10 | 484563 | 0.992933 |
| rs7932891 | 11 | 10921512 | A | G | 0.2954 | 0.0141 | 0.002 | 1.39E-12 | 626262 | 0.998727 |
| rs10502148 | 11 | 111647998 | T | C | 0.3572 | -0.023 | 0.0018 | 5.87E-36 | 627223 | 0.993649 |
| rs11214040 | 11 | 111786628 | A | G | 0.1205 | 0.0195 | 0.0031 | 2.15E-10 | 484563 | 0.980664 |
| rs10891490 | 11 | 112885527 | T | C | 0.4153 | 0.01 | 0.0017 | 8.94E-09 | 694519 | 0.995728 |
| rs7943309 | 11 | 116773653 | A | G | 0.0276 | 0.0272 | 0.0047 | 9.59E-09 | 691026 | 0.996471 |
| rs7914 | 11 | 119180316 | A | G | 0.2403 | 0.0118 | 0.0021 | 2.45E-08 | 614273 | 1 |
| rs747249 | 11 | 130271647 | A | G | 0.3589 | 0.0114 | 0.0019 | 1.25E-09 | 626947 | 0.985753 |
| rs2970332 | 11 | 14360435 | A | G | 0.7608 | 0.0156 | 0.0021 | 1.01E-13 | 627144 | 0.994859 |
| rs79634051 | 11 | 14561945 | C | G | 0.0269 | -0.0343 | 0.0061 | 2.08E-08 | 484563 | 1 |
| rs11023364 | 11 | 14872568 | A | G | 0.5843 | -0.0121 | 0.002 | 1.43E-09 | 484563 | 0.99821 |
| rs7928810 | 11 | 17372443 | A | C | 0.6105 | -0.0113 | 0.0018 | 1.41E-10 | 692557 | 0.996009 |
| rs4141261 | 11 | 17708379 | T | G | 0.3617 | -0.0104 | 0.0019 | 2.33E-08 | 627207 | 0.989974 |
| rs3842761 | 11 | 2179352 | C | G | 0.2512 | 0.0131 | 0.0023 | 1.11E-08 | 484563 | 0.971039 |
| rs11605956 | 11 | 26212866 | T | C | 0.6948 | 0.0122 | 0.0022 | 1.57E-08 | 484563 | 0.98942 |
| rs72889643 | 11 | 26236292 | T | G | 0.0632 | 0.0235 | 0.0041 | 8.94E-09 | 484563 | 0.991079 |
| rs2301254 | 11 | 32457675 | A | G | 0.6212 | 0.0133 | 0.002 | 5.35E-11 | 484563 | 0.990454 |
| rs7114403 | 11 | 36361607 | A | T | 0.5179 | 0.0127 | 0.0018 | 1.20E-12 | 626659 | 0.996562 |
| rs62621409 | 11 | 36458997 | A | G | 0.9586 | -0.0273 | 0.005 | 3.43E-08 | 484563 | 1 |
| rs34312154 | 11 | 47470345 | A | G | 0.0945 | 0.0188 | 0.003 | 3.18E-10 | 551850 | 1 |
| rs2509963 | 11 | 62192931 | T | C | 0.2609 | -0.017 | 0.002 | 9.36E-17 | 626704 | 0.99285 |
| rs2509985 | 11 | 62216041 | A | G | 0.3776 | 0.0116 | 0.0019 | 5.67E-10 | 613940 | 0.986632 |
| rs2509967 | 11 | 62312786 | C | G | 0.6338 | -0.0199 | 0.0019 | 1.41E-26 | 626343 | 0.991433 |
| rs76300755 | 11 | 62421760 | T | G | 0.905 | 0.0209 | 0.0034 | 7.55E-10 | 484563 | 0.985363 |
| rs12291674 | 11 | 62926353 | T | C | 0.9699 | 0.0331 | 0.0055 | 1.51E-09 | 611274 | 0.973728 |
| rs2186643 | 11 | 63878074 | A | G | 0.467 | -0.0141 | 0.0018 | 4.63E-15 | 626788 | 0.984445 |
| rs56271783 | 11 | 64004723 | C | G | 0.0439 | 0.059 | 0.0049 | 1.21E-33 | 484563 | 0.975925 |
| rs588298 | 11 | 65253574 | A | G | 0.6569 | -0.0111 | 0.0019 | 3.78E-09 | 627270 | 0.992859 |
| rs3741378 | 11 | 65408937 | T | C | 0.144 | -0.0224 | 0.0025 | 1.13E-19 | 691070 | 1 |
| rs68162171 | 11 | 66125461 | A | C | 0.7135 | -0.0146 | 0.0022 | 1.87E-11 | 484563 | 0.999377 |
| rs7952436 | 11 | 67024534 | T | C | 0.0785 | -0.022 | 0.0037 | 2.03E-09 | 484563 | 1 |
| rs76684833 | 11 | 68957933 | A | C | 0.9823 | 0.0447 | 0.0076 | 5.04E-09 | 484563 | 0.961623 |
| rs67330701 | 11 | 69079707 | T | C | 0.0927 | -0.0263 | 0.0035 | 1.06E-13 | 484563 | 0.922528 |
| rs72932183 | 11 | 69150932 | A | G | 0.1695 | -0.0222 | 0.0027 | 1.54E-16 | 484563 | 0.951649 |
| rs4357716 | 11 | 69163161 | T | C | 0.137 | 0.0208 | 0.0027 | 7.06E-15 | 616618 | 0.988243 |
| rs4980598 | 11 | 69231796 | T | C | 0.3366 | -0.0182 | 0.0019 | 1.32E-21 | 627265 | 0.997443 |
| rs7395513 | 11 | 69262756 | A | G | 0.4389 | -0.0214 | 0.002 | 9.35E-27 | 484563 | 0.99223 |
| rs12225226 | 11 | 69303977 | A | G | 0.8441 | 0.0152 | 0.0027 | 2.47E-08 | 484563 | 0.994103 |
| rs3017621 | 11 | 69456901 | C | G | 0.9065 | -0.0227 | 0.0035 | 6.35E-11 | 484563 | 0.955398 |
| rs150046733 | 11 | 732354 | T | C | 0.9456 | -0.0273 | 0.0044 | 5.47E-10 | 484563 | 0.98251 |
| rs2186955 | 11 | 74375475 | T | C | 0.8103 | 0.0123 | 0.0022 | 1.53E-08 | 694481 | 0.988988 |
| rs1138714 | 11 | 825110 | A | G | 0.4445 | 0.0148 | 0.0018 | 3.41E-16 | 624448 | 0.981783 |
| rs11042077 | 11 | 8825966 | A | G | 0.6564 | 0.0124 | 0.0019 | 4.99E-11 | 627010 | 0.990878 |
| rs2195243 | 12 | 102922986 | C | G | 0.2084 | -0.0119 | 0.0021 | 1.15E-08 | 690382 | 1 |
| rs833715 | 12 | 103068400 | A | G | 0.1297 | 0.0147 | 0.0027 | 3.92E-08 | 615045 | 0.993017 |
| rs10778504 | 12 | 107120424 | T | C | 0.3497 | 0.0158 | 0.0021 | 2.53E-14 | 484563 | 0.999088 |
| rs3764002 | 12 | 108618630 | T | C | 0.2635 | -0.0177 | 0.002 | 1.86E-19 | 663799 | 1 |
| rs10850127 | 12 | 113512945 | T | C | 0.3056 | 0.013 | 0.002 | 8.17E-11 | 603657 | 0.993936 |
| rs1896313 | 12 | 115395280 | T | C | 0.4357 | -0.0099 | 0.0017 | 8.60E-09 | 694596 | 0.995151 |
| rs11043299 | 12 | 122483139 | T | C | 0.3779 | 0.0189 | 0.0022 | 7.72E-18 | 484563 | 0.856555 |
| rs142827533 | 12 | 122521486 | A | G | 0.9579 | -0.0281 | 0.005 | 2.17E-08 | 484563 | 0.959505 |
| rs11060051 | 12 | 122674143 | C | G | 0.3996 | 0.0134 | 0.0023 | 3.51E-09 | 484563 | 0.775534 |
| rs11058375 | 12 | 122971142 | A | T | 0.9649 | -0.0331 | 0.0054 | 1.11E-09 | 484563 | 0.975392 |
| rs11615433 | 12 | 123018475 | T | C | 0.8723 | 0.0185 | 0.0028 | 3.84E-11 | 610604 | 0.981372 |
| rs584844 | 12 | 123155899 | T | C | 0.3536 | -0.0185 | 0.0024 | 4.39E-15 | 484563 | 0.764791 |
| rs486036 | 12 | 123160730 | T | G | 0.1269 | -0.0191 | 0.003 | 1.94E-10 | 484563 | 0.981665 |
| rs3922628 | 12 | 123209295 | A | T | 0.2113 | -0.0283 | 0.0024 | 1.81E-31 | 484563 | 0.989359 |
| rs11060344 | 12 | 123329101 | A | G | 0.8628 | -0.0356 | 0.0029 | 1.64E-35 | 484563 | 0.997866 |
| rs10846509 | 12 | 123792848 | A | C | 0.0765 | 0.0297 | 0.0036 | 1.22E-16 | 689345 | 0.999648 |
| rs112160305 | 12 | 123824516 | T | C | 0.0136 | -0.049 | 0.0086 | 1.51E-08 | 484563 | 0.96966 |
| rs28577594 | 12 | 123895906 | C | G | 0.7103 | 0.0193 | 0.0022 | 2.84E-19 | 484563 | 0.996414 |
| rs1706477 | 12 | 123913697 | T | G | 0.5778 | -0.0112 | 0.0019 | 3.93E-09 | 597009 | 0.974784 |
| rs73216963 | 12 | 124034690 | T | C | 0.0918 | -0.0297 | 0.0036 | 6.25E-17 | 484563 | 0.921109 |
| rs10846527 | 12 | 124043296 | C | G | 0.0865 | 0.0296 | 0.0032 | 1.07E-20 | 627133 | 1 |
| rs1114628 | 12 | 124280357 | T | G | 0.3369 | 0.0209 | 0.0019 | 2.66E-28 | 626806 | 0.993506 |
| rs141193534 | 12 | 124348787 | A | G | 0.019 | -0.056 | 0.0075 | 7.07E-14 | 484563 | 0.931538 |
| rs139192229 | 12 | 124412928 | A | G | 0.0161 | -0.0464 | 0.0079 | 5.33E-09 | 484563 | 0.971904 |
| rs112236972 | 12 | 124470432 | T | C | 0.0211 | -0.0458 | 0.0069 | 2.74E-11 | 484563 | 0.99554 |
| rs7307277 | 12 | 124475156 | A | G | 0.6556 | 0.0382 | 0.0018 | 7.63E-99 | 694291 | 0.997565 |
| rs11608738 | 12 | 124483614 | C | G | 0.92 | -0.026 | 0.0034 | 1.53E-14 | 610637 | 1 |
| rs863750 | 12 | 124505444 | T | C | 0.5842 | 0.0373 | 0.0017 | 4.17E-101 | 693934 | 0.993593 |
| rs825498 | 12 | 124580058 | A | G | 0.1199 | 0.0202 | 0.0032 | 2.17E-10 | 484563 | 0.900947 |
| rs61953638 | 12 | 124633209 | T | C | 0.0507 | -0.0377 | 0.0045 | 5.04E-17 | 484563 | 1 |
| rs11057465 | 12 | 124634880 | A | C | 0.8018 | -0.0222 | 0.0023 | 4.36E-22 | 627249 | 0.996494 |
| rs138808037 | 12 | 124666336 | T | C | 0.0301 | 0.045 | 0.0061 | 1.93E-13 | 484563 | 0.889866 |
| rs4765495 | 12 | 124739888 | C | G | 0.8887 | -0.0178 | 0.0031 | 1.14E-08 | 484563 | 0.98309 |
| rs10773111 | 12 | 125332955 | T | C | 0.3976 | -0.0098 | 0.0017 | 1.88E-08 | 693844 | 0.992697 |
| rs7973997 | 12 | 128756938 | T | C | 0.3585 | 0.0106 | 0.0019 | 2.20E-08 | 625704 | 0.990765 |
| rs34322 | 12 | 12879570 | T | C | 0.4663 | 0.01 | 0.0018 | 2.67E-08 | 626680 | 1 |
| rs12823266 | 12 | 131441717 | A | G | 0.7241 | 0.0131 | 0.0021 | 2.37E-10 | 606371 | 0.995955 |
| rs12828318 | 12 | 133766122 | A | G | 0.8262 | 0.0173 | 0.0023 | 3.84E-14 | 684927 | 0.994361 |
| rs10842689 | 12 | 26338365 | T | C | 0.4875 | -0.0123 | 0.0018 | 3.99E-12 | 627275 | 0.99992 |
| rs9668810 | 12 | 26426420 | T | C | 0.2691 | -0.0182 | 0.002 | 2.49E-20 | 627295 | 1 |
| rs11048456 | 12 | 26463082 | T | C | 0.7648 | -0.0342 | 0.0019 | 1.05E-69 | 694431 | 0.99471 |
| rs4964058 | 12 | 27522766 | A | G | 0.5145 | 0.0113 | 0.0018 | 2.84E-10 | 626919 | 0.993021 |
| rs56196860 | 12 | 2908330 | A | C | 0.03 | -0.0357 | 0.0058 | 6.45E-10 | 484563 | 1 |
| rs11051005 | 12 | 30783475 | A | G | 0.7677 | 0.0159 | 0.002 | 6.41E-16 | 694572 | 0.998327 |
| rs2200155 | 12 | 33734935 | A | G | 0.3718 | -0.0138 | 0.0019 | 1.26E-13 | 627133 | 0.997285 |
| rs73108788 | 12 | 45985272 | T | G | 0.1746 | 0.0163 | 0.0026 | 3.81E-10 | 484563 | 0.98696 |
| rs2544027 | 12 | 48216529 | A | G | 0.474 | 0.0132 | 0.002 | 3.34E-11 | 484563 | 0.989402 |
| rs75824359 | 12 | 54320490 | A | G | 0.9678 | 0.043 | 0.0057 | 3.61E-14 | 484563 | 0.960302 |
| rs71455259 | 12 | 54331583 | T | C | 0.0754 | -0.0288 | 0.0037 | 1.27E-14 | 484563 | 1 |
| rs1443512 | 12 | 54342684 | A | C | 0.2215 | 0.0308 | 0.002 | 1.16E-53 | 694543 | 1 |
| rs3803042 | 12 | 54387947 | A | G | 0.4295 | 0.0268 | 0.0018 | 4.84E-49 | 625688 | 0.996149 |
| rs12308675 | 12 | 54496006 | T | C | 0.4354 | -0.0117 | 0.002 | 3.69E-09 | 484563 | 0.996008 |
| rs79811812 | 12 | 54584330 | T | C | 0.0636 | 0.0263 | 0.004 | 7.41E-11 | 484563 | 1 |
| rs2293413 | 12 | 56096816 | T | C | 0.501 | 0.0105 | 0.0018 | 4.98E-09 | 624624 | 0.995538 |
| rs2277339 | 12 | 57146069 | T | G | 0.8931 | -0.0224 | 0.0029 | 2.35E-14 | 614842 | 1 |
| rs10784510 | 12 | 66422753 | T | C | 0.6784 | 0.0149 | 0.0019 | 9.12E-16 | 693198 | 0.994028 |
| rs10878367 | 12 | 66436097 | A | T | 0.295 | 0.019 | 0.002 | 5.84E-21 | 611538 | 0.984942 |
| rs7300366 | 12 | 6652227 | A | G | 0.2646 | -0.0109 | 0.002 | 2.47E-08 | 694480 | 0.997234 |
| rs711076 | 12 | 77905207 | T | C | 0.6797 | -0.0123 | 0.0018 | 1.49E-11 | 694439 | 0.995925 |
| rs1805740 | 12 | 9075014 | T | G | 0.7185 | -0.0179 | 0.002 | 1.16E-19 | 627211 | 0.998009 |
| rs10745659 | 12 | 94092690 | C | G | 0.5607 | -0.0127 | 0.0018 | 1.82E-12 | 627031 | 0.992346 |
| rs7311622 | 12 | 98772975 | T | C | 0.4528 | -0.0104 | 0.0018 | 1.07E-08 | 625488 | 0.988621 |
| rs9557378 | 13 | 100747566 | A | G | 0.2647 | 0.0113 | 0.002 | 2.50E-08 | 626569 | 0.99216 |
| rs664532 | 13 | 110932363 | T | C | 0.3811 | 0.0134 | 0.0019 | 1.38E-12 | 551855 | 0.987828 |
| rs61964262 | 13 | 110937654 | T | C | 0.1366 | -0.0149 | 0.0026 | 7.50E-09 | 551878 | 0.990574 |
| rs4773173 | 13 | 111025118 | A | G | 0.6743 | 0.0153 | 0.0018 | 4.23E-17 | 686141 | 0.994639 |
| rs1547149 | 13 | 22476288 | A | G | 0.6464 | -0.0123 | 0.0019 | 6.55E-11 | 627074 | 0.963649 |
| rs1340819 | 13 | 29145323 | A | C | 0.6625 | 0.0102 | 0.0018 | 2.18E-08 | 692892 | 0.997634 |
| rs1360485 | 13 | 31031884 | T | C | 0.6789 | 0.0114 | 0.0019 | 8.04E-10 | 690926 | 0.989807 |
| rs10507524 | 13 | 44684600 | T | C | 0.9046 | -0.0182 | 0.0031 | 2.68E-09 | 627283 | 0.993459 |
| rs9316439 | 13 | 49895644 | A | G | 0.3282 | 0.0124 | 0.0019 | 2.69E-11 | 694210 | 0.989406 |
| rs1535470 | 13 | 50260029 | A | G | 0.6286 | 0.0106 | 0.0019 | 1.44E-08 | 626210 | 0.997547 |
| rs6561566 | 13 | 50445012 | A | T | 0.2072 | -0.0131 | 0.0022 | 2.30E-09 | 661355 | 0.993587 |
| rs2475837 | 13 | 50535863 | T | C | 0.1191 | 0.0186 | 0.0026 | 1.15E-12 | 678119 | 0.998869 |
| rs150493080 | 13 | 50609483 | T | G | 0.0525 | 0.0264 | 0.0045 | 6.38E-09 | 484563 | 0.953332 |
| rs11842698 | 13 | 50896325 | A | G | 0.9613 | 0.041 | 0.0044 | 1.87E-20 | 615586 | 0.997742 |
| rs7334326 | 13 | 50967732 | C | G | 0.146 | 0.0229 | 0.0028 | 1.71E-16 | 484563 | 0.995604 |
| rs188014 | 13 | 50969923 | T | C | 0.1127 | -0.0153 | 0.0028 | 3.21E-08 | 694541 | 0.998063 |
| rs2225226 | 13 | 51127270 | T | C | 0.2062 | 0.016 | 0.0021 | 1.87E-14 | 694152 | 0.994112 |
| rs797486 | 13 | 51221618 | A | C | 0.893 | 0.0373 | 0.0026 | 4.38E-46 | 694585 | 0.987915 |
| rs17067999 | 13 | 78163787 | T | C | 0.1356 | -0.0164 | 0.0026 | 1.45E-10 | 627309 | 0.998523 |
| rs9601364 | 13 | 80952100 | T | C | 0.7909 | 0.0136 | 0.0024 | 2.36E-08 | 484563 | 0.987198 |
| rs1577099 | 13 | 93894245 | T | C | 0.5157 | 0.0099 | 0.0018 | 3.13E-08 | 627211 | 1 |
| rs1057119 | 14 | 23746269 | C | G | 0.7725 | 0.0134 | 0.0022 | 9.17E-10 | 620993 | 1 |
| rs12435790 | 14 | 35154381 | A | G | 0.9209 | 0.0214 | 0.0035 | 1.16E-09 | 605319 | 0.963582 |
| rs140664623 | 14 | 52321987 | T | G | 0.023 | -0.0366 | 0.0067 | 4.21E-08 | 484563 | 0.974753 |
| rs1253682 | 14 | 52463153 | T | C | 0.4795 | 0.0104 | 0.0017 | 1.68E-09 | 691750 | 0.995205 |
| rs146182298 | 14 | 52531408 | T | C | 0.0381 | 0.0314 | 0.0052 | 1.45E-09 | 484563 | 0.988814 |
| rs2000224 | 14 | 53987220 | A | G | 0.3357 | 0.0109 | 0.0019 | 9.24E-09 | 627203 | 0.998482 |
| rs7160860 | 14 | 54339493 | T | C | 0.1533 | -0.0139 | 0.0024 | 7.64E-09 | 627230 | 0.996736 |
| rs1190982 | 14 | 58815839 | T | C | 0.3027 | 0.0159 | 0.0019 | 2.46E-16 | 626709 | 0.997466 |
| rs1254319 | 14 | 60903757 | A | G | 0.3021 | -0.0121 | 0.0019 | 7.11E-11 | 694412 | 0.992653 |
| rs1977211 | 14 | 68829295 | A | G | 0.477 | -0.0116 | 0.002 | 5.04E-09 | 484563 | 0.998212 |
| rs4902632 | 14 | 69149428 | A | T | 0.1778 | 0.0201 | 0.0024 | 2.39E-16 | 604448 | 0.946236 |
| rs2526886 | 14 | 71359064 | T | G | 0.69 | 0.0138 | 0.0021 | 1.01E-10 | 484563 | 0.995027 |
| rs862031 | 14 | 74991855 | A | G | 0.3582 | -0.0124 | 0.0018 | 7.21E-12 | 689384 | 0.995277 |
| rs7492628 | 14 | 91547136 | C | G | 0.6801 | -0.0229 | 0.0019 | 4.91E-32 | 626927 | 0.981371 |
| rs8003238 | 14 | 98345604 | A | G | 0.5958 | 0.0127 | 0.0018 | 2.61E-12 | 627295 | 0.996646 |
| rs2603229 | 15 | 100263499 | T | C | 0.2495 | 0.0123 | 0.0021 | 2.99E-09 | 627113 | 0.991767 |
| rs12441543 | 15 | 31689543 | A | G | 0.2896 | -0.0189 | 0.002 | 8.68E-22 | 625657 | 0.994264 |
| rs1826947 | 15 | 36105651 | A | G | 0.4095 | -0.0101 | 0.0018 | 7.83E-09 | 694318 | 0.980252 |
| rs2701523 | 15 | 37384284 | A | G | 0.741 | 0.0129 | 0.002 | 3.21E-10 | 626427 | 0.98337 |
| rs35565646 | 15 | 40660515 | A | G | 0.1687 | 0.0181 | 0.0026 | 7.17E-12 | 484563 | 0.996311 |
| rs7169010 | 15 | 41047024 | A | G | 0.5137 | -0.0117 | 0.002 | 6.28E-09 | 484563 | 0.972025 |
| rs1105881 | 15 | 42072530 | C | G | 0.6349 | -0.0156 | 0.0019 | 1.10E-16 | 627207 | 0.994047 |
| rs11549015 | 15 | 42235316 | T | C | 0.1649 | 0.0134 | 0.0024 | 3.53E-08 | 626413 | 1 |
| rs12908370 | 15 | 47905717 | T | C | 0.6587 | -0.0107 | 0.0019 | 1.86E-08 | 627303 | 0.994622 |
| rs17703883 | 15 | 51530097 | T | C | 0.7627 | -0.0149 | 0.0021 | 7.27E-13 | 627178 | 0.995352 |
| rs77507269 | 15 | 51723139 | T | C | 0.9799 | 0.0409 | 0.0071 | 7.49E-09 | 484563 | 0.989633 |
| rs12595496 | 15 | 56528806 | A | G | 0.8664 | -0.0257 | 0.0026 | 1.05E-23 | 694565 | 0.999631 |
| rs12913413 | 15 | 56761266 | A | G | 0.782 | 0.0128 | 0.0022 | 4.90E-09 | 627148 | 0.994548 |
| rs4775348 | 15 | 61313142 | A | T | 0.9144 | 0.0179 | 0.0031 | 1.15E-08 | 625015 | 0.995741 |
| rs12440695 | 15 | 62435156 | T | C | 0.6153 | -0.0103 | 0.0018 | 6.33E-09 | 681364 | 0.996761 |
| rs2058914 | 15 | 63831984 | A | G | 0.7272 | -0.0118 | 0.0019 | 1.56E-10 | 694589 | 0.997051 |
| rs1440372 | 15 | 67033151 | T | C | 0.2666 | -0.0155 | 0.0019 | 9.53E-16 | 692010 | 0.993347 |
| rs12442323 | 15 | 67654919 | T | C | 0.2084 | -0.0149 | 0.0022 | 1.71E-11 | 551869 | 0.997427 |
| rs2306022 | 15 | 68628163 | T | C | 0.0873 | 0.0179 | 0.0032 | 2.23E-08 | 626992 | 1 |
| rs750460 | 15 | 74241506 | A | G | 0.4393 | -0.013 | 0.0018 | 4.81E-13 | 627104 | 1 |
| rs8027447 | 15 | 74321481 | C | G | 0.5766 | 0.0127 | 0.0018 | 3.09E-12 | 627221 | 0.992009 |
| rs7176070 | 15 | 79033489 | T | C | 0.2508 | 0.0138 | 0.0023 | 1.19E-09 | 484563 | 1 |
| rs1516797 | 15 | 89410314 | T | G | 0.677 | 0.0105 | 0.0019 | 2.84E-08 | 615255 | 0.998312 |
| rs8030277 | 15 | 94029153 | A | T | 0.6766 | -0.0132 | 0.0019 | 5.97E-12 | 626916 | 0.996694 |
| rs28439341 | 15 | 95851378 | A | G | 0.0944 | -0.0204 | 0.0036 | 1.85E-08 | 484563 | 0.857896 |
| rs13379908 | 15 | 96176302 | T | C | 0.8288 | -0.0133 | 0.0024 | 3.73E-08 | 627212 | 0.986037 |
| rs13379794 | 15 | 98367100 | A | G | 0.3945 | 0.0109 | 0.0018 | 4.34E-09 | 624626 | 0.995554 |
| rs2684787 | 15 | 99505062 | T | C | 0.242 | 0.013 | 0.0021 | 4.73E-10 | 626740 | 0.997123 |
| rs11074934 | 16 | 10979440 | T | C | 0.2719 | 0.0119 | 0.002 | 3.79E-09 | 626749 | 0.988985 |
| rs876476 | 16 | 11150248 | A | G | 0.279 | 0.0112 | 0.002 | 1.29E-08 | 627194 | 0.997165 |
| rs7102 | 16 | 11642242 | T | C | 0.646 | -0.0115 | 0.0018 | 1.38E-10 | 686994 | 0.98977 |
| rs7187167 | 16 | 1349209 | T | C | 0.287 | 0.0112 | 0.002 | 2.13E-08 | 627271 | 0.989434 |
| rs28610092 | 16 | 2155819 | A | C | 0.8276 | 0.0158 | 0.0026 | 1.62E-09 | 484563 | 0.991364 |
| rs12325187 | 16 | 3364997 | C | G | 0.7323 | 0.0127 | 0.0021 | 8.30E-10 | 626249 | 0.990636 |
| rs11641742 | 16 | 427784 | A | G | 0.5886 | 0.0105 | 0.0019 | 1.71E-08 | 592081 | 0.999506 |
| rs2387280 | 16 | 4339684 | A | T | 0.7585 | 0.0178 | 0.0023 | 1.72E-14 | 484563 | 0.98506 |
| rs611704 | 16 | 4395241 | A | G | 0.0414 | 0.0258 | 0.0047 | 4.89E-08 | 609335 | 1 |
| rs3747577 | 16 | 4415696 | C | G | 0.2689 | 0.0215 | 0.002 | 3.34E-26 | 626179 | 0.987155 |
| rs2047937 | 16 | 49864791 | T | C | 0.517 | -0.0147 | 0.0017 | 7.31E-18 | 694322 | 0.999687 |
| rs1861649 | 16 | 49886366 | T | C | 0.869 | 0.0175 | 0.0029 | 2.04E-09 | 484563 | 1 |
| rs8054299 | 16 | 53498655 | C | G | 0.6861 | 0.0146 | 0.0019 | 5.79E-14 | 615528 | 0.999166 |
| rs16957415 | 16 | 67418957 | A | G | 0.9554 | 0.0313 | 0.0043 | 3.29E-13 | 627093 | 1 |
| rs400223 | 16 | 73809928 | T | C | 0.4845 | 0.0104 | 0.0018 | 9.24E-09 | 614135 | 0.986336 |
| rs17767383 | 16 | 79744401 | A | G | 0.341 | 0.0103 | 0.0018 | 2.26E-08 | 694570 | 0.999546 |
| rs2925979 | 16 | 81534790 | T | C | 0.2959 | 0.0265 | 0.0019 | 7.33E-46 | 692391 | 1 |
| rs79311290 | 16 | 85150163 | A | G | 0.8951 | -0.0195 | 0.0034 | 1.03E-08 | 484563 | 0.89648 |
| rs7198287 | 16 | 85258191 | T | C | 0.218 | -0.0137 | 0.0022 | 1.05E-09 | 623331 | 0.993015 |
| rs1169644 | 16 | 86537032 | A | G | 0.7708 | 0.0121 | 0.0022 | 3.90E-08 | 626787 | 0.980136 |
| rs11078594 | 17 | 1612777 | C | G | 0.187 | -0.0149 | 0.0025 | 3.66E-09 | 484563 | 1 |
| rs1269402 | 17 | 17361137 | T | C | 0.2638 | 0.0178 | 0.002 | 5.35E-19 | 626961 | 0.988403 |
| rs9944423 | 17 | 17484934 | A | G | 0.9463 | -0.0258 | 0.0036 | 4.27E-13 | 692225 | 0.990587 |
| rs12936587 | 17 | 17543722 | A | G | 0.4782 | -0.0184 | 0.0017 | 9.25E-27 | 693646 | 1 |
| rs854785 | 17 | 18039263 | A | T | 0.6331 | -0.0126 | 0.0021 | 3.44E-09 | 484563 | 0.91656 |
| rs672356 | 17 | 18266567 | A | G | 0.2964 | -0.0172 | 0.0022 | 1.92E-15 | 484563 | 0.998709 |
| rs7213608 | 17 | 21279289 | T | C | 0.6826 | -0.0115 | 0.0019 | 3.15E-09 | 626694 | 0.977134 |
| rs8076739 | 17 | 27714587 | T | C | 0.5366 | -0.0101 | 0.0017 | 2.74E-09 | 692392 | 0.999097 |
| rs3087591 | 17 | 29630970 | A | G | 0.6849 | 0.0114 | 0.0019 | 1.00E-09 | 692400 | 0.998545 |
| rs8070260 | 17 | 34866915 | A | G | 0.4734 | 0.0099 | 0.0018 | 3.41E-08 | 627179 | 1 |
| rs3744070 | 17 | 36924339 | A | G | 0.1048 | 0.0161 | 0.0029 | 3.46E-08 | 627026 | 0.99246 |
| rs7225453 | 17 | 3960620 | T | C | 0.825 | -0.0145 | 0.0023 | 2.66E-10 | 627294 | 0.99837 |
| rs60090576 | 17 | 40001119 | A | G | 0.4343 | 0.0127 | 0.002 | 2.54E-10 | 484563 | 0.986485 |
| rs148849317 | 17 | 40313182 | A | G | 0.0575 | -0.0247 | 0.0043 | 1.29E-08 | 484563 | 0.955016 |
| rs9908237 | 17 | 40455690 | T | C | 0.4715 | 0.0122 | 0.002 | 7.94E-10 | 484563 | 0.974939 |
| rs72823057 | 17 | 40567260 | C | G | 0.0731 | 0.0294 | 0.0038 | 1.69E-14 | 484563 | 0.98187 |
| rs7359598 | 17 | 40897470 | T | C | 0.4521 | 0.0129 | 0.0018 | 2.23E-12 | 591983 | 0.995297 |
| rs7613 | 17 | 43471481 | A | G | 0.2855 | -0.0147 | 0.0022 | 1.83E-11 | 484563 | 0.995689 |
| rs55938136 | 17 | 43798360 | A | G | 0.7833 | -0.0181 | 0.0024 | 3.29E-14 | 484563 | 0.999806 |
| rs7221167 | 17 | 43933307 | T | C | 0.5719 | -0.0182 | 0.002 | 6.80E-19 | 484563 | 0.950548 |
| rs118185397 | 17 | 44141223 | T | C | 0.0709 | -0.0257 | 0.0038 | 2.51E-11 | 484563 | 1 |
| rs12452064 | 17 | 44868187 | A | G | 0.4463 | -0.0109 | 0.0018 | 2.77E-09 | 626838 | 0.974441 |
| rs4794033 | 17 | 47358481 | A | G | 0.1012 | -0.0194 | 0.003 | 8.69E-11 | 626793 | 0.983585 |
| rs8074638 | 17 | 53731579 | A | G | 0.191 | 0.0157 | 0.0023 | 2.12E-11 | 626827 | 0.977124 |
| rs227733 | 17 | 54769174 | A | C | 0.2835 | 0.0128 | 0.002 | 7.52E-11 | 626990 | 0.99438 |
| rs757608 | 17 | 59497277 | A | G | 0.3207 | 0.0194 | 0.0018 | 1.10E-26 | 694592 | 1 |
| rs9902847 | 17 | 59526121 | A | G | 0.5713 | -0.0133 | 0.0018 | 1.95E-13 | 626690 | 0.992351 |
| rs2854152 | 17 | 61986027 | A | G | 0.3189 | 0.0133 | 0.0019 | 3.94E-12 | 626386 | 0.989808 |
| rs4247361 | 17 | 65396360 | A | G | 0.3929 | -0.0137 | 0.0019 | 4.42E-13 | 619757 | 0.958238 |
| rs9915538 | 17 | 68230544 | A | G | 0.6933 | 0.0109 | 0.0019 | 1.42E-08 | 627099 | 0.994267 |
| rs8066985 | 17 | 68453345 | A | G | 0.4878 | 0.0228 | 0.0017 | 2.03E-40 | 694540 | 0.996236 |
| rs149057443 | 17 | 68586720 | A | G | 0.035 | 0.0325 | 0.0054 | 1.72E-09 | 484563 | 0.986601 |
| rs7220854 | 17 | 69307999 | T | C | 0.3432 | 0.012 | 0.0021 | 8.85E-09 | 484563 | 0.985971 |
| rs1029645 | 17 | 70295760 | A | G | 0.545 | -0.0129 | 0.0018 | 7.20E-13 | 627175 | 0.995081 |
| rs3744405 | 17 | 7193255 | A | G | 0.5032 | -0.01 | 0.0018 | 4.54E-08 | 607595 | 0.98473 |
| rs2279162 | 17 | 73259083 | T | C | 0.6806 | 0.013 | 0.0018 | 8.67E-13 | 692313 | 1 |
| rs10512606 | 17 | 74211208 | A | C | 0.9204 | -0.0278 | 0.0032 | 2.36E-18 | 687831 | 0.989622 |
| rs9891533 | 17 | 74239595 | A | G | 0.3575 | -0.0119 | 0.0021 | 7.63E-09 | 484563 | 0.998766 |
| rs727428 | 17 | 7537792 | T | C | 0.454 | 0.0158 | 0.0018 | 4.25E-18 | 626586 | 1 |
| rs2376585 | 17 | 76417883 | T | C | 0.1963 | -0.0175 | 0.0023 | 5.50E-14 | 626927 | 0.992505 |
| rs35344256 | 17 | 79934194 | A | C | 0.3042 | 0.0142 | 0.0021 | 3.82E-11 | 484563 | 1 |
| rs1561 | 17 | 8263334 | T | C | 0.0705 | 0.0216 | 0.0035 | 9.25E-10 | 687694 | 0.928054 |
| rs7235010 | 18 | 20724810 | A | G | 0.774 | 0.0186 | 0.0021 | 5.65E-19 | 694222 | 0.998884 |
| rs11664106 | 18 | 2846812 | A | T | 0.6304 | 0.0282 | 0.0021 | 5.90E-41 | 484563 | 0.948036 |
| rs76724145 | 18 | 2855161 | T | C | 0.0765 | -0.026 | 0.0039 | 3.49E-11 | 484563 | 0.896483 |
| rs1893781 | 18 | 42443643 | A | C | 0.1158 | 0.0175 | 0.0028 | 3.27E-10 | 625010 | 0.996859 |
| rs17739187 | 18 | 42749456 | A | C | 0.2583 | -0.013 | 0.0021 | 5.51E-10 | 627313 | 0.996937 |
| rs35710478 | 18 | 45377882 | C | G | 0.4406 | 0.0118 | 0.002 | 3.76E-09 | 484563 | 0.98514 |
| rs7242873 | 18 | 46885025 | A | G | 0.9184 | -0.0242 | 0.0032 | 2.55E-14 | 694627 | 0.998169 |
| rs12454712 | 18 | 60845884 | T | C | 0.6243 | 0.017 | 0.0018 | 1.33E-20 | 654356 | 1 |
| rs889129 | 19 | 10070954 | A | T | 0.8774 | -0.0198 | 0.0029 | 9.32E-12 | 593244 | 0.96509 |
| rs11085744 | 19 | 10819967 | T | C | 0.5645 | 0.0109 | 0.0018 | 1.95E-09 | 626346 | 0.98824 |
| rs17766692 | 19 | 11342599 | T | C | 0.0652 | 0.0243 | 0.004 | 1.14E-09 | 484563 | 0.992205 |
| rs113091342 | 19 | 13090688 | T | C | 0.0407 | -0.0316 | 0.005 | 4.03E-10 | 484563 | 0.966541 |
| rs34597097 | 19 | 13950294 | A | G | 0.2239 | -0.0137 | 0.0024 | 1.09E-08 | 484563 | 0.972597 |
| rs55957788 | 19 | 17207851 | A | G | 0.296 | 0.0161 | 0.0022 | 7.82E-14 | 484563 | 0.99899 |
| rs91710 | 19 | 18141123 | A | G | 0.4408 | 0.0106 | 0.0018 | 2.31E-09 | 675243 | 0.971481 |
| rs12608504 | 19 | 18389135 | A | G | 0.3596 | 0.0258 | 0.0018 | 3.09E-47 | 694553 | 0.9985 |
| rs2232967 | 19 | 18423638 | C | G | 0.891 | 0.0177 | 0.0032 | 2.71E-08 | 484563 | 0.992516 |
| rs12162223 | 19 | 18635457 | C | G | 0.5661 | 0.0129 | 0.002 | 9.79E-11 | 484563 | 0.991082 |
| rs73001065 | 19 | 19460541 | C | G | 0.0606 | 0.0219 | 0.0037 | 3.68E-09 | 551749 | 0.992649 |
| rs12459350 | 19 | 2176586 | A | G | 0.5422 | 0.0141 | 0.0017 | 2.39E-16 | 694419 | 0.999463 |
| rs12461296 | 19 | 33774591 | A | C | 0.2053 | -0.0126 | 0.0023 | 3.34E-08 | 598580 | 1 |
| rs41355649 | 19 | 33790556 | A | G | 0.0649 | -0.0363 | 0.004 | 1.59E-19 | 484563 | 1 |
| rs7251505 | 19 | 33802542 | A | G | 0.0935 | -0.0455 | 0.003 | 1.26E-51 | 691217 | 0.997892 |
| rs7250362 | 19 | 33805720 | C | G | 0.4057 | 0.0209 | 0.002 | 1.43E-24 | 484563 | 0.967298 |
| rs7254869 | 19 | 33814693 | T | C | 0.9232 | -0.0256 | 0.0037 | 4.06E-12 | 484563 | 0.987411 |
| rs3786897 | 19 | 33893008 | A | G | 0.5803 | -0.0279 | 0.0017 | 3.00E-58 | 693294 | 0.997489 |
| rs891038 | 19 | 34044928 | A | G | 0.1377 | 0.0174 | 0.0029 | 1.43E-09 | 484563 | 0.990703 |
| rs58519763 | 19 | 4058395 | A | G | 0.1121 | 0.0182 | 0.0033 | 2.20E-08 | 484563 | 0.931483 |
| rs13041 | 19 | 4502282 | T | C | 0.5153 | 0.012 | 0.002 | 2.42E-09 | 484563 | 0.960623 |
| rs4420638 | 19 | 45422946 | A | G | 0.813 | 0.0229 | 0.0024 | 6.81E-22 | 604317 | 1 |
| rs73942938 | 19 | 46162346 | T | C | 0.7362 | -0.0146 | 0.0025 | 4.46E-09 | 551862 | 0.995002 |
| rs838145 | 19 | 49248730 | A | G | 0.5479 | -0.0115 | 0.0018 | 2.44E-10 | 613255 | 0.986765 |
| rs8103017 | 19 | 55999142 | C | G | 0.7044 | -0.0196 | 0.0022 | 2.98E-19 | 484563 | 0.980636 |
| rs1799815 | 19 | 7125519 | A | G | 0.0663 | -0.0245 | 0.004 | 7.47E-10 | 484563 | 1 |
| rs7251963 | 19 | 7170241 | T | C | 0.4491 | -0.0094 | 0.0017 | 4.78E-08 | 694540 | 0.995716 |
| rs3786680 | 19 | 7183551 | T | C | 0.0963 | -0.0206 | 0.0029 | 1.08E-12 | 692661 | 0.988069 |
| rs1035942 | 19 | 7199803 | A | G | 0.2746 | 0.0141 | 0.002 | 2.14E-12 | 626240 | 0.997307 |
| rs2241588 | 19 | 8468485 | T | C | 0.1672 | 0.0152 | 0.0027 | 1.11E-08 | 484563 | 0.978205 |
| rs4542783 | 19 | 8642160 | T | C | 0.5655 | 0.01 | 0.0018 | 2.01E-08 | 641414 | 0.994527 |
| rs62621197 | 19 | 8670147 | T | C | 0.0361 | -0.0358 | 0.0055 | 8.88E-11 | 484563 | 0.924188 |
| rs6040229 | 20 | 10883131 | A | G | 0.5481 | -0.0112 | 0.0018 | 5.27E-10 | 627216 | 0.99839 |
| rs6047259 | 20 | 21098319 | T | C | 0.6188 | 0.0104 | 0.0018 | 4.32E-09 | 694395 | 0.989467 |
| rs143384 | 20 | 34025756 | A | G | 0.5867 | 0.0178 | 0.0017 | 9.86E-25 | 693189 | 1 |
| rs6124220 | 20 | 38880243 | T | C | 0.2867 | -0.0129 | 0.0019 | 1.08E-11 | 686399 | 0.997209 |
| rs910071 | 20 | 39213710 | T | C | 0.3151 | -0.0171 | 0.0019 | 6.27E-19 | 627030 | 0.994225 |
| rs1997833 | 20 | 39690342 | T | C | 0.7071 | -0.0138 | 0.0019 | 1.63E-13 | 694226 | 0.998656 |
| rs2236519 | 20 | 45529571 | A | G | 0.3693 | 0.0298 | 0.0019 | 4.07E-58 | 626533 | 0.993531 |
| rs113377538 | 20 | 45543857 | T | G | 0.059 | -0.0297 | 0.0044 | 1.56E-11 | 484563 | 0.908474 |
| rs6066148 | 20 | 45602037 | C | G | 0.2556 | -0.0127 | 0.0021 | 6.72E-10 | 626561 | 0.995806 |
| rs6018291 | 20 | 45756772 | C | G | 0.4453 | -0.0139 | 0.0018 | 1.33E-14 | 627099 | 0.993289 |
| rs17789520 | 20 | 48839705 | A | G | 0.0963 | 0.0171 | 0.0031 | 4.64E-08 | 611590 | 0.982312 |
| rs2904155 | 20 | 50963243 | A | G | 0.372 | -0.0169 | 0.0018 | 4.63E-20 | 627245 | 0.99724 |
| rs910382 | 20 | 51699189 | A | G | 0.4937 | -0.0187 | 0.0018 | 2.76E-25 | 625776 | 0.991927 |
| rs1293407 | 20 | 51776315 | T | C | 0.9244 | -0.0241 | 0.0037 | 1.35E-10 | 484563 | 0.995641 |
| rs6084945 | 20 | 549364 | T | C | 0.2223 | 0.0123 | 0.0022 | 3.77E-08 | 596823 | 1 |
| rs1328757 | 20 | 56135199 | T | C | 0.4745 | 0.0141 | 0.0018 | 3.41E-15 | 626402 | 0.991493 |
| rs805770 | 20 | 5668714 | T | C | 0.3979 | 0.0222 | 0.0018 | 1.33E-33 | 624279 | 1 |
| rs8126001 | 20 | 62711459 | T | C | 0.4853 | -0.0164 | 0.002 | 1.60E-16 | 484563 | 0.992915 |
| rs2145272 | 20 | 6626218 | A | G | 0.6367 | -0.0247 | 0.0018 | 3.95E-43 | 694482 | 1 |
| rs6054471 | 20 | 6674887 | T | C | 0.2582 | 0.0122 | 0.0021 | 4.10E-09 | 627050 | 0.989721 |
| rs170986 | 20 | 6760431 | A | C | 0.1693 | 0.0145 | 0.0026 | 1.52E-08 | 579343 | 0.979741 |
| rs28451064 | 21 | 35593827 | A | G | 0.1268 | 0.018 | 0.003 | 2.87E-09 | 484563 | 0.955171 |
| rs2898237 | 21 | 36763769 | A | G | 0.6879 | 0.0118 | 0.0018 | 7.47E-11 | 694572 | 0.99577 |
| rs79593853 | 21 | 36835576 | T | C | 0.9243 | -0.0216 | 0.0038 | 1.20E-08 | 484563 | 0.977636 |
| rs2836179 | 21 | 39544159 | A | G | 0.4133 | -0.0167 | 0.0018 | 7.16E-20 | 627290 | 0.9967 |
| rs11088991 | 21 | 46783285 | T | C | 0.4172 | 0.0125 | 0.002 | 5.26E-10 | 484563 | 0.988268 |
| rs2330328 | 21 | 47308501 | T | C | 0.1595 | -0.0134 | 0.0024 | 2.28E-08 | 694494 | 0.995605 |
| rs2839108 | 21 | 47535248 | T | C | 0.7385 | -0.0149 | 0.002 | 1.74E-13 | 624905 | 0.995233 |
| rs8142329 | 22 | 27647289 | A | G | 0.8752 | -0.0165 | 0.0026 | 1.60E-10 | 680293 | 1 |
| rs2283847 | 22 | 28181399 | T | C | 0.5594 | 0.0116 | 0.0018 | 1.21E-10 | 665284 | 0.945527 |
| rs5762906 | 22 | 29338235 | T | C | 0.956 | -0.0483 | 0.0046 | 1.31E-25 | 627274 | 0.986263 |
| rs2294239 | 22 | 29449477 | A | G | 0.5685 | 0.0243 | 0.0017 | 4.04E-44 | 694017 | 0.989833 |
| rs134666 | 22 | 29495254 | A | G | 0.7234 | 0.011 | 0.002 | 2.36E-08 | 627170 | 0.99247 |
| rs17451053 | 22 | 29537124 | T | C | 0.1849 | 0.017 | 0.0024 | 7.76E-13 | 627193 | 0.997285 |
| rs13056562 | 22 | 30677722 | T | C | 0.1275 | 0.0226 | 0.003 | 3.80E-14 | 484563 | 0.980267 |
| rs2027982 | 22 | 31593435 | T | C | 0.4255 | -0.0121 | 0.0018 | 2.20E-11 | 627258 | 0.998906 |
| rs1053593 | 22 | 35660875 | T | G | 0.6424 | -0.014 | 0.0018 | 3.07E-15 | 685581 | 1 |
| rs4821897 | 22 | 39835587 | A | G | 0.2431 | 0.0118 | 0.0021 | 9.93E-09 | 627002 | 0.993083 |
| rs801593 | 22 | 47195050 | C | G | 0.7396 | -0.0123 | 0.002 | 6.68E-10 | 625084 | 1 |

| **HIPadjBMI SNPs** | **CHR** | **POS** | **Effect Allele** | **Other Allele** | **Effect Allele Frequency** | **BETA** | **SE** | **P** | **N** |
| --- | --- | --- | --- | --- | --- | --- | --- | --- | --- |
| rs1046934 | 1 | 184023529 | C | A | 0.375 | 0.023 | 0.0037 | 5.50E-10 | 210450 |
| rs11205303 | 1 | 149906413 | C | T | 0.3583 | 0.041 | 0.004 | 5.80E-25 | 196314 |
| rs11808940 | 1 | 172244386 | T | A | 0.1833 | 0.023 | 0.0041 | 2.30E-08 | 208157 |
| rs12086130 | 1 | 51173603 | T | C | 0.1 | 0.037 | 0.0063 | 2.80E-09 | 206610 |
| rs12744534 | 1 | 170658355 | G | A | 0.1441 | 0.035 | 0.0054 | 2.00E-10 | 210115 |
| rs17346473 | 1 | 172083093 | G | A | 0.2167 | 0.03 | 0.004 | 2.90E-14 | 210431 |
| rs2820443 | 1 | 219753509 | C | T | 0.3 | 0.048 | 0.0039 | 1.50E-35 | 211030 |
| rs3748656 | 1 | 113236681 | T | C | 0.2 | -0.024 | 0.0042 | 6.10E-09 | 210890 |
| rs6657613 | 1 | 17328200 | A | T | 0.4661 | -0.031 | 0.0036 | 3.90E-18 | 210917 |
| rs6672530 | 1 | 227771216 | C | A | 0.2333 | -0.028 | 0.0046 | 8.40E-10 | 208172 |
| rs714515 | 1 | 172352990 | G | A | 0.4583 | -0.022 | 0.0036 | 2.60E-10 | 204574 |
| rs7521902 | 1 | 22490724 | A | C | 0.1917 | 0.024 | 0.0044 | 3.40E-08 | 210318 |
| rs991967 | 1 | 218615451 | C | A | 0.1897 | 0.022 | 0.0038 | 6.50E-09 | 210314 |
| rs10195252 | 2 | 165513091 | T | C | 0.5583 | -0.023 | 0.0036 | 1.10E-10 | 210403 |
| rs1545552 | 2 | 33360338 | A | G | 0.2917 | -0.029 | 0.004 | 6.10E-13 | 208132 |
| rs3791679 | 2 | 56096892 | A | G | 0.725 | 0.038 | 0.0041 | 1.20E-20 | 210979 |
| rs4973517 | 2 | 233161818 | C | T | 0.25 | -0.029 | 0.0045 | 1.90E-10 | 175930 |
| rs6739772 | 2 | 241840711 | G | A | 0.7667 | 0.022 | 0.0039 | 1.20E-08 | 210702 |
| rs10804591 | 3 | 129334233 | A | C | 0.85 | -0.038 | 0.0044 | 7.40E-18 | 210953 |
| rs11242 | 3 | 53125922 | C | T | 0.575 | -0.027 | 0.0036 | 6.30E-14 | 204637 |
| rs1388251 | 3 | 56559244 | G | A | 0.2583 | -0.023 | 0.004 | 2.10E-08 | 211029 |
| rs17819328 | 3 | 12489342 | G | T | 0.45 | -0.023 | 0.0037 | 8.60E-10 | 209843 |
| rs2098771 | 3 | 185563274 | A | G | 0.6667 | -0.022 | 0.0041 | 3.60E-08 | 196732 |
| rs2597513 | 3 | 13555836 | C | T | 0.125 | 0.033 | 0.0059 | 2.00E-08 | 210951 |
| rs4243400 | 3 | 171970859 | G | A | 0.5 | 0.025 | 0.0036 | 2.70E-12 | 210478 |
| rs724016 | 3 | 141105570 | A | G | 0.5167 | -0.048 | 0.0035 | 7.50E-43 | 211032 |
| rs12648786 | 4 | 145521703 | G | A | 0.5917 | -0.032 | 0.0039 | 1.80E-16 | 199289 |
| rs1662837 | 4 | 82168889 | C | T | 0.275 | 0.028 | 0.0038 | 1.40E-13 | 210825 |
| rs1812175 | 4 | 145574844 | A | G | 0.1917 | -0.059 | 0.0048 | 1.60E-34 | 211054 |
| rs2247341 | 4 | 1701317 | G | A | 0.5833 | -0.023 | 0.0037 | 6.50E-10 | 211076 |
| rs6830062 | 4 | 18017730 | C | T | 0.1583 | -0.034 | 0.0051 | 2.30E-11 | 211015 |
| rs9993613 | 4 | 73476014 | G | T | 0.4917 | -0.027 | 0.0045 | 6.80E-10 | 143494 |
| rs1173771 | 5 | 32815028 | A | G | 0.475 | 0.026 | 0.0036 | 6.10E-13 | 210986 |
| rs6556079 | 5 | 172997078 | A | G | 0.5917 | -0.028 | 0.0045 | 4.50E-10 | 143741 |
| rs12207675 | 6 | 76237741 | C | T | 0.1333 | 0.041 | 0.0055 | 1.10E-13 | 211077 |
| rs1294410 | 6 | 6738752 | C | T | 0.625 | -0.029 | 0.0037 | 1.70E-15 | 210861 |
| rs13216391 | 6 | 7789943 | A | G | 0.85 | -0.028 | 0.0047 | 2.60E-09 | 199240 |
| rs1538170 | 6 | 126752874 | C | T | 0.6167 | -0.026 | 0.0038 | 2.50E-12 | 201926 |
| rs16894959 | 6 | 34825662 | C | T | 0.1 | 0.036 | 0.005 | 3.20E-13 | 210242 |
| rs1759645 | 6 | 34194866 | C | T | 0.125 | 0.029 | 0.0051 | 9.20E-09 | 209671 |
| rs2230365 | 6 | 31525448 | T | C | 0.125 | -0.027 | 0.0049 | 4.80E-08 | 209805 |
| rs2844619 | 6 | 31242223 | G | C | 0.5333 | -0.022 | 0.0039 | 2.50E-08 | 200426 |
| rs6570509 | 6 | 142716286 | T | G | 0.2583 | -0.045 | 0.004 | 1.20E-29 | 197803 |
| rs6903448 | 6 | 76173832 | T | C | 0.1583 | -0.034 | 0.005 | 4.50E-12 | 211031 |
| rs7759938 | 6 | 105378954 | C | T | 0.3583 | 0.028 | 0.0038 | 1.90E-13 | 211029 |
| rs806794 | 6 | 26200677 | G | A | 0.275 | -0.046 | 0.004 | 2.00E-31 | 204203 |
| rs9388766 | 6 | 130354855 | C | T | 0.6667 | -0.028 | 0.0039 | 7.50E-13 | 211072 |
| rs9491696 | 6 | 127452639 | G | C | 0.525 | -0.023 | 0.0036 | 1.40E-10 | 210813 |
| rs975496 | 6 | 35233443 | G | A | 0.8417 | 0.031 | 0.0051 | 1.20E-09 | 199331 |
| rs10950949 | 7 | 23519260 | A | C | 0.6167 | 0.021 | 0.0038 | 1.50E-08 | 201716 |
| rs42235 | 7 | 92248076 | C | T | 0.6583 | -0.036 | 0.0039 | 8.40E-20 | 208455 |
| rs798497 | 7 | 2795957 | A | G | 0.7167 | 0.035 | 0.0038 | 3.60E-20 | 210942 |
| rs849141 | 7 | 28185091 | G | A | 0.7083 | -0.032 | 0.0039 | 1.60E-16 | 211081 |
| rs10958476 | 8 | 57095808 | T | C | 0.8583 | -0.028 | 0.0046 | 8.80E-10 | 199716 |
| rs6470764 | 8 | 130725665 | T | C | 0.1917 | -0.039 | 0.0045 | 8.40E-18 | 210864 |
| rs6984782 | 8 | 57135889 | C | T | 0.1167 | -0.033 | 0.0054 | 2.00E-09 | 210592 |
| rs7008867 | 8 | 23383204 | G | A | 0.7917 | -0.024 | 0.0041 | 6.00E-09 | 211066 |
| rs894345 | 8 | 135613581 | T | C | 0.375 | -0.02 | 0.0036 | 1.00E-08 | 210876 |
| rs10123368 | 9 | 99111384 | T | C | 0.8 | -0.026 | 0.0044 | 5.00E-09 | 210933 |
| rs4448343 | 9 | 98266370 | A | G | 0.6833 | -0.024 | 0.0037 | 3.30E-11 | 210984 |
| rs473902 | 9 | 98256235 | T | G | 0.9417 | 0.058 | 0.0075 | 6.60E-15 | 184792 |
| rs606452 | 11 | 75276178 | A | C | 0.1583 | 0.029 | 0.0051 | 9.70E-09 | 210552 |
| rs686320 | 11 | 65244538 | C | G | 0.0917 | -0.038 | 0.0055 | 6.80E-12 | 199308 |
| rs10748128 | 12 | 69827658 | T | G | 0.3583 | 0.023 | 0.004 | 4.00E-09 | 197305 |
| rs11612228 | 12 | 576984 | T | C | 0.3167 | 0.023 | 0.0041 | 2.80E-08 | 181613 |
| rs12817549 | 12 | 94121314 | C | T | 0.4333 | -0.029 | 0.0036 | 2.20E-16 | 210856 |
| rs1351394 | 12 | 66351826 | T | C | 0.4833 | 0.025 | 0.0035 | 4.90E-13 | 210068 |
| rs1727294 | 12 | 123616514 | G | A | 0.8 | -0.032 | 0.0043 | 8.30E-14 | 208707 |
| rs1809889 | 12 | 124801226 | T | C | 0.2417 | 0.023 | 0.0041 | 1.60E-08 | 208493 |
| rs2638953 | 12 | 28534415 | C | G | 0.6333 | 0.024 | 0.0038 | 2.00E-10 | 207847 |
| rs7953508 | 12 | 93983554 | C | T | 0.75 | -0.024 | 0.004 | 3.50E-09 | 210624 |
| rs3118906 | 13 | 51106788 | A | G | 0.2417 | -0.03 | 0.0039 | 8.00E-15 | 211005 |
| rs558003 | 13 | 51172963 | G | A | 0.9583 | -0.049 | 0.0063 | 3.80E-15 | 199267 |
| rs10140922 | 14 | 35830114 | T | G | 0.375 | -0.03 | 0.0046 | 4.60E-11 | 143568 |
| rs1254257 | 14 | 60832179 | T | C | 0.7167 | -0.029 | 0.0048 | 9.10E-10 | 143808 |
| rs16942341 | 15 | 89388905 | T | C | 0.025 | -0.066 | 0.012 | 3.80E-08 | 192440 |
| rs4246307 | 15 | 100763824 | G | A | 0.4917 | 0.024 | 0.0043 | 4.20E-08 | 143549 |
| rs4262914 | 15 | 84484897 | A | G | 0.5417 | 0.02 | 0.0036 | 1.60E-08 | 210620 |
| rs7162542 | 15 | 84514290 | G | C | 0.5167 | 0.041 | 0.0036 | 1.80E-30 | 209929 |
| rs17193922 | 16 | 53544630 | C | G | 0.6167 | -0.024 | 0.004 | 1.40E-09 | 198589 |
| rs2377058 | 16 | 89734831 | A | G | 0.75 | -0.023 | 0.0038 | 6.90E-10 | 210642 |
| rs3747579 | 16 | 4445327 | C | T | 0.2667 | -0.023 | 0.004 | 6.60E-09 | 210715 |
| rs7187776 | 16 | 28857645 | G | A | 0.3667 | 0.02 | 0.0036 | 4.00E-08 | 210057 |
| rs1120297 | 17 | 68441938 | C | T | 0.5167 | -0.021 | 0.0035 | 1.70E-09 | 210991 |
| rs13695 | 17 | 38545193 | C | T | 0.725 | -0.027 | 0.0044 | 9.50E-10 | 190010 |
| rs2034088 | 17 | 423051 | T | C | 0.6167 | 0.021 | 0.0036 | 4.80E-09 | 210737 |
| rs561341 | 17 | 30316385 | T | G | 0.825 | -0.031 | 0.005 | 3.30E-10 | 211106 |
| rs7223966 | 17 | 61893398 | G | A | 0.675 | -0.029 | 0.0039 | 1.70E-13 | 211080 |
| rs9890032 | 17 | 29165934 | C | G | 0.625 | 0.026 | 0.0037 | 2.00E-12 | 207385 |
| rs181553 | 18 | 46562030 | G | A | 0.3167 | -0.029 | 0.0038 | 8.90E-15 | 210832 |
| rs4369779 | 18 | 20735408 | C | T | 0.7417 | 0.035 | 0.0044 | 3.30E-15 | 210787 |
| rs12980348 | 19 | 2181607 | T | G | 0.625 | -0.029 | 0.0036 | 8.80E-16 | 210456 |
| rs891088 | 19 | 7184762 | A | G | 0.725 | -0.026 | 0.004 | 1.00E-10 | 210109 |
| rs143384 | 20 | 34025756 | A | G | 0.6 | -0.044 | 0.0038 | 1.10E-31 | 209682 |
| rs169797 | 20 | 31926490 | A | G | 0.75 | 0.024 | 0.004 | 9.70E-10 | 204594 |
| rs2326788 | 20 | 6470094 | A | G | 0.3083 | -0.022 | 0.0036 | 1.50E-09 | 210499 |
| rs6088619 | 20 | 33411871 | A | G | 0.8667 | -0.039 | 0.0054 | 9.30E-13 | 199166 |
| rs1053593 | 22 | 35660875 | G | T | 0.3417 | -0.021 | 0.0038 | 3.90E-08 | 202070 |

| **WC SNPs** | **CHR** | **POS** | **Effect Allele** | **Other Allele** | **Effect Allele Frequency** | **BETA** | **SE** | **P** | **N** |
| --- | --- | --- | --- | --- | --- | --- | --- | --- | --- |
| rs11165623 | 1 | 96893000 | A | G | 0.4833 | 0.02 | 0.0034 | 5.20E-09 | 232057 |
| rs2820292 | 1 | 201784287 | A | C | 0.4917 | -0.019 | 0.0034 | 2.40E-08 | 231899 |
| rs3127553 | 1 | 49438005 | G | A | 0.3667 | 0.023 | 0.0035 | 1.60E-10 | 231815 |
| rs4130548 | 1 | 78463868 | C | T | 0.425 | 0.022 | 0.0035 | 3.40E-10 | 232042 |
| rs633715 | 1 | 177852580 | C | T | 0.2667 | 0.043 | 0.0043 | 3.30E-23 | 218883 |
| rs7531118 | 1 | 72837239 | T | C | 0.3917 | -0.027 | 0.0035 | 1.50E-14 | 231912 |
| rs7550711 | 1 | 110082886 | T | C | 0.0339 | 0.058 | 0.0098 | 3.40E-09 | 212149 |
| rs6545714 | 2 | 59307725 | G | A | 0.375 | 0.022 | 0.0035 | 1.90E-10 | 232046 |
| rs6755502 | 2 | 635721 | T | C | 0.125 | -0.051 | 0.0045 | 2.00E-30 | 231849 |
| rs929641 | 2 | 58792377 | A | G | 0.6167 | 0.021 | 0.0034 | 1.20E-09 | 231976 |
| rs1516725 | 3 | 185824004 | T | C | 0.0917 | -0.031 | 0.0051 | 1.70E-09 | 230610 |
| rs2325036 | 3 | 85819412 | A | C | 0.5917 | 0.023 | 0.0035 | 2.10E-11 | 232048 |
| rs3849570 | 3 | 81792112 | A | C | 0.3667 | 0.021 | 0.0038 | 2.20E-08 | 196103 |
| rs6440003 | 3 | 141094209 | G | A | 0.5167 | -0.021 | 0.0034 | 2.90E-10 | 231985 |
| rs10938397 | 4 | 45182527 | A | G | 0.5667 | -0.032 | 0.0035 | 6.10E-20 | 231679 |
| rs2112347 | 5 | 75015242 | G | T | 0.375 | -0.025 | 0.0035 | 3.20E-13 | 232028 |
| rs16894959 | 6 | 34825662 | C | T | 0.1 | 0.026 | 0.0048 | 3.40E-08 | 230687 |
| rs2489623 | 6 | 127455821 | C | A | 0.5583 | 0.019 | 0.0034 | 3.40E-08 | 231857 |
| rs806794 | 6 | 26200677 | G | A | 0.275 | -0.022 | 0.0037 | 2.10E-09 | 225694 |
| rs9400239 | 6 | 108977663 | C | T | 0.7 | 0.024 | 0.0036 | 1.90E-11 | 232015 |
| rs943005 | 6 | 50865820 | T | C | 0.1 | 0.039 | 0.0044 | 7.20E-19 | 232080 |
| rs10968576 | 9 | 28414339 | G | A | 0.2917 | 0.025 | 0.0036 | 1.20E-11 | 232050 |
| rs6163 | 10 | 104596924 | C | A | 0.6083 | -0.019 | 0.0035 | 3.70E-08 | 225708 |
| rs7903146 | 10 | 114758349 | T | C | 0.25 | -0.022 | 0.0037 | 3.90E-09 | 232078 |
| rs10742178 | 11 | 27613718 | G | A | 0.75 | 0.028 | 0.004 | 2.10E-12 | 232040 |
| rs10767658 | 11 | 27672252 | C | G | 0.3583 | 0.031 | 0.0037 | 3.30E-17 | 225754 |
| rs10840100 | 11 | 8669437 | G | A | 0.725 | 0.02 | 0.0035 | 5.40E-09 | 232029 |
| rs2293576 | 11 | 47434986 | A | G | 0.3667 | -0.022 | 0.0036 | 9.40E-10 | 226024 |
| rs7138803 | 12 | 50247468 | G | A | 0.5583 | -0.028 | 0.0035 | 1.60E-15 | 232059 |
| rs12429545 | 13 | 54102206 | G | A | 0.9 | -0.031 | 0.0052 | 2.50E-09 | 222998 |
| rs10132280 | 14 | 25928179 | A | C | 0.3333 | -0.022 | 0.0037 | 2.20E-09 | 231985 |
| rs12885454 | 14 | 29736838 | C | A | 0.6333 | 0.02 | 0.0035 | 2.60E-08 | 231357 |
| rs7144011 | 14 | 79940383 | T | G | 0.275 | 0.033 | 0.0041 | 9.40E-16 | 232007 |
| rs4776970 | 15 | 68080886 | T | A | 0.3417 | -0.02 | 0.0035 | 2.30E-08 | 230989 |
| rs11643744 | 16 | 53791798 | A | G | 0.7 | 0.027 | 0.0036 | 4.10E-14 | 230653 |
| rs12446632 | 16 | 19935389 | A | G | 0.1333 | -0.036 | 0.005 | 5.20E-13 | 226701 |
| rs1549293 | 16 | 31141993 | T | C | 0.3917 | -0.02 | 0.0035 | 7.30E-09 | 232022 |
| rs1558902 | 16 | 53803574 | A | T | 0.45 | 0.074 | 0.0035 | 3.70E-101 | 230183 |
| rs2531992 | 16 | 4021734 | A | G | 0.1667 | -0.028 | 0.0048 | 3.00E-09 | 232034 |
| rs7498665 | 16 | 28883241 | G | A | 0.3583 | 0.034 | 0.0035 | 1.40E-22 | 230193 |
| rs11873305 | 18 | 58049192 | A | C | 0.9667 | 0.058 | 0.0092 | 3.10E-10 | 211782 |
| rs6567160 | 18 | 57829135 | C | T | 0.2833 | 0.048 | 0.004 | 2.60E-33 | 231894 |
| rs7239883 | 18 | 40147671 | G | A | 0.3167 | 0.021 | 0.0035 | 2.30E-09 | 231731 |
| rs9675376 | 18 | 57969244 | A | G | 0.325 | 0.036 | 0.0038 | 3.80E-21 | 220004 |
| rs2075650 | 19 | 45395619 | A | G | 0.8583 | 0.031 | 0.005 | 8.90E-10 | 222559 |
| rs2287019 | 19 | 46202172 | C | T | 0.85 | 0.035 | 0.0046 | 1.70E-14 | 217525 |
| rs3810291 | 19 | 47569003 | A | G | 0.625 | 0.026 | 0.004 | 1.70E-10 | 210501 |
| rs16996700 | 20 | 50981945 | T | C | 0.7 | 0.023 | 0.0037 | 1.50E-09 | 231903 |

| **T2D SNPs** | **LD r2 with index SNP** | **CHR** | **BP** | **Effect Allele** | **Other Allele** | **Effect Allele Frequency** | **BETA** | **SE** | **P** | **N** |
| --- | --- | --- | --- | --- | --- | --- | --- | --- | --- | --- |
| rs3768321 |  | 1 | 40035928 | T | G | 0.2 | 0.085 | 0.008 | 1.30E-26 | 231420 |
| rs58432198 |  | 1 | 51256091 | T | C | 0.12 | -0.065 | 0.01 | 1.80E-10 | 231109 |
| rs12140153 |  | 1 | 62579891 | T | G | 0.095 | -0.064 | 0.011 | 1.20E-08 | 231420 |
| rs1127215 |  | 1 | 117532790 | T | C | 0.42 | -0.047 | 0.0064 | 2.30E-13 | 231420 |
| rs1493694 |  | 1 | 120526982 | T | C | 0.11 | 0.084 | 0.01 | 2.10E-16 | 231420 |
| rs10305745 |  | 1 | 150786038 | A | G | 0.015 | 0.25 | 0.053 | 3.90E-06 | 229543 |
| rs145904381 |  | 1 | 151017991 | T | C | 0.99 | 0.17 | 0.031 | 2.20E-08 | 231420 |
| rs539515 |  | 1 | 177889025 | A | C | 0.8 | -0.051 | 0.008 | 1.20E-10 | 231420 |
| rs12048743 |  | 1 | 205114873 | C | G | 0.56 | -0.038 | 0.0064 | 4.40E-09 | 231420 |
| rs9430095 |  | 1 | 206593900 | C | G | 0.49 | 0.036 | 0.0064 | 2.30E-08 | 231420 |
| rs79687284 |  | 1 | 214150821 | C | G | 0.035 | 0.18 | 0.018 | 9.20E-23 | 231420 |
| 1_214159256_C_T |  | 1 | 214159256 | T | C | 0.44 | -0.068 | 0.0064 | 5.60E-26 | 231420 |
| rs114526150 |  | 1 | 214175531 | T | G | 0.98 | -0.073 | 0.022 | 9.10E-04 | 231420 |
| rs2820446 |  | 1 | 219748818 | C | G | 0.71 | 0.057 | 0.007 | 3.70E-16 | 231420 |
| rs348330 |  | 1 | 229672955 | A | G | 0.64 | -0.051 | 0.0067 | 3.90E-14 | 231420 |
| rs291368 | 1 | 1 | 235691384 | T | C | 0.37 | -0.04 | 0.0066 | 2.30E-09 | 231420 |
| rs62107261 |  | 2 | 422144 | T | C | 0.95 | 0.11 | 0.016 | 1.80E-11 | 231420 |
| rs35913461 |  | 2 | 653575 | T | C | 0.17 | -0.056 | 0.0086 | 5.90E-11 | 231420 |
| rs11680058 |  | 2 | 16574669 | A | G | 0.86 | 0.058 | 0.01 | 1.30E-08 | 231419 |
| rs17802463 |  | 2 | 25643221 | T | G | 0.27 | -0.039 | 0.0071 | 3.50E-08 | 231420 |
| rs1260326 |  | 2 | 27730940 | T | C | 0.39 | -0.067 | 0.0065 | 1.30E-24 | 231420 |
| rs28525376 |  | 2 | 43207872 | T | G | 0.58 | -0.027 | 0.0064 | 2.10E-05 | 231420 |
| rs6708643 |  | 2 | 43430440 | A | G | 0.5 | 0.038 | 0.0064 | 3.30E-09 | 231420 |
| rs80147536 |  | 2 | 43698028 | A | T | 0.9 | 0.13 | 0.011 | 2.70E-30 | 231420 |
| rs1116357 |  | 2 | 57287411 | A | G | 0.49 | -0.019 | 0.0062 | 2.60E-03 | 231420 |
| rs10193538 |  | 2 | 58981064 | T | G | 0.61 | 0.037 | 0.0065 | 1.70E-08 | 231420 |
| rs6545714 |  | 2 | 59307725 | A | G | 0.61 | -0.037 | 0.0065 | 1.70E-08 | 231420 |
| rs243024 |  | 2 | 60583665 | A | G | 0.46 | 0.058 | 0.0063 | 4.40E-20 | 231420 |
| rs2249105 |  | 2 | 65287896 | A | G | 0.63 | 0.053 | 0.0066 | 1.20E-15 | 231420 |
| rs2052261 |  | 2 | 65355270 | A | G | 0.7 | 0.032 | 0.0069 | 3.80E-06 | 231420 |
| rs2028150 |  | 2 | 65655012 | C | G | 0.6 | 0.052 | 0.0065 | 3.10E-15 | 231420 |
| rs11123407 | 0.67 | 2 | 111950594 | A | C | 0.46 | 0.023 | 0.0063 | 2.40E-04 | 231420 |
| rs11688931 |  | 2 | 121318166 | C | G | 0.85 | 0.061 | 0.009 | 1.10E-11 | 231420 |
| rs11688682 |  | 2 | 121347612 | C | G | 0.27 | -0.058 | 0.0075 | 1.40E-14 | 231420 |
| rs66477705 |  | 2 | 121378852 | T | C | 0.97 | 0.08 | 0.018 | 1.50E-05 | 231420 |
| rs6707326 | 0.645 | 2 | 135357705 | A | G | 0.57 | -0.018 | 0.0066 | 8.40E-03 | 231109 |
| rs35999103 |  | 2 | 147861633 | T | C | 0.15 | 0.052 | 0.0091 | 8.30E-09 | 231420 |
| rs13426680 |  | 2 | 158339550 | A | G | 0.94 | 0.082 | 0.013 | 6.40E-10 | 231420 |
| rs3772071 |  | 2 | 161135544 | T | C | 0.71 | 0.048 | 0.007 | 1.60E-11 | 231420 |
| 2_165513091_C_T |  | 2 | 165513091 | T | C | 0.59 | 0.06 | 0.0064 | 1.60E-20 | 231420 |
| rs13024606 |  | 2 | 165573194 | T | C | 0.047 | 0.055 | 0.015 | 3.80E-04 | 231420 |
| rs113414093 |  | 2 | 219859171 | A | G | 0.061 | 0.08 | 0.016 | 6.40E-07 | 231420 |
| rs2972144 |  | 2 | 227101411 | A | G | 0.36 | -0.094 | 0.0066 | 7.90E-46 | 231420 |
| 2_228973660_A_G |  | 2 | 228973660 | A | G | 0.52 | -0.034 | 0.0063 | 1.30E-07 | 231420 |
| rs11709077 |  | 3 | 12336507 | A | G | 0.12 | -0.11 | 0.0098 | 1.60E-27 | 231420 |
| rs17819328 |  | 3 | 12489342 | T | G | 0.58 | -0.031 | 0.0064 | 1.10E-06 | 231420 |
| rs13094957 | 1 | 3 | 23457080 | T | C | 0.79 | 0.071 | 0.0079 | 1.10E-19 | 231420 |
| rs17013314 |  | 3 | 23510044 | A | G | 0.97 | -0.12 | 0.019 | 1.00E-10 | 231420 |
| rs11926707 |  | 3 | 46925539 | T | C | 0.37 | -0.038 | 0.0066 | 1.50E-08 | 231420 |
| rs75423501 |  | 3 | 47242923 | A | G | 0.9 | -0.062 | 0.011 | 4.10E-08 | 231109 |
| rs4688760 |  | 3 | 49980596 | T | C | 0.68 | 0.043 | 0.0069 | 4.50E-10 | 231420 |
| rs7640294 | 1 | 3 | 53130913 | A | C | 0.56 | 0.036 | 0.0064 | 3.00E-08 | 231420 |
| rs76263492 |  | 3 | 54828827 | T | G | 0.045 | 0.091 | 0.016 | 6.30E-09 | 231420 |
| rs3774723 |  | 3 | 63962339 | A | G | 0.16 | -0.065 | 0.0089 | 2.00E-13 | 231420 |
| rs4504165 | 1 | 3 | 64701890 | T | C | 0.7 | 0.054 | 0.007 | 1.20E-14 | 231420 |
| rs13085136 |  | 3 | 72865183 | T | C | 0.072 | -0.074 | 0.013 | 1.40E-08 | 231420 |
| rs2272163 |  | 3 | 77671721 | A | C | 0.38 | -0.037 | 0.0065 | 1.20E-08 | 231420 |
| 3_123065778_A_G |  | 3 | 123065778 | A | G | 0.77 | 0.089 | 0.0076 | 1.30E-31 | 231420 |
| rs649961 |  | 3 | 124926637 | T | C | 0.47 | 0.038 | 0.0063 | 1.30E-09 | 231420 |
| rs9828772 |  | 3 | 129333182 | C | G | 0.9 | 0.059 | 0.011 | 4.20E-08 | 231420 |
| 3_138119952_C_T |  | 3 | 138119952 | T | C | 0.84 | -0.036 | 0.0088 | 3.60E-05 | 231420 |
| rs62271373 |  | 3 | 150066540 | A | T | 0.055 | 0.088 | 0.014 | 1.00E-09 | 231420 |
| rs4472028 |  | 3 | 152053250 | T | C | 0.45 | 0.034 | 0.0064 | 2.00E-07 | 231420 |
| rs147579559 |  | 3 | 152086533 | A | G | 0.6 | 0.039 | 0.0065 | 3.60E-09 | 231420 |
| rs74653713 |  | 3 | 152417881 | A | C | 0.043 | -0.094 | 0.016 | 5.70E-09 | 231420 |
| rs35497231 |  | 3 | 152433628 | T | C | 0.68 | -0.034 | 0.0068 | 5.80E-07 | 231420 |
| rs7629630 |  | 3 | 168218841 | A | T | 0.86 | 0.051 | 0.0091 | 2.20E-08 | 231420 |
| rs9873618 |  | 3 | 170733076 | A | G | 0.29 | -0.066 | 0.007 | 8.50E-21 | 231420 |
| rs2872246 |  | 3 | 183738460 | A | C | 0.45 | 0.036 | 0.0063 | 1.80E-08 | 231420 |
| rs6780171 |  | 3 | 185503456 | A | T | 0.31 | 0.11 | 0.0068 | 2.50E-58 | 231420 |
| rs150111048 |  | 3 | 185514421 | A | G | 0.76 | -0.11 | 0.021 | 9.10E-08 | 217712 |
| rs11717959 |  | 3 | 185541213 | T | G | 0.38 | 0.039 | 0.0065 | 1.70E-09 | 231420 |
| rs1516728 |  | 3 | 185829891 | A | T | 0.76 | 0.032 | 0.0074 | 1.60E-05 | 231420 |
| 3_186665645_C_T |  | 3 | 186665645 | T | C | 0.55 | 0.055 | 0.0064 | 1.40E-17 | 231420 |
| rs7645517 |  | 3 | 186675277 | A | G | 0.058 | 0.046 | 0.014 | 9.50E-04 | 231420 |
| rs4686471 |  | 3 | 187740899 | T | C | 0.39 | -0.06 | 0.0065 | 3.10E-20 | 231420 |
| rs111827885 |  | 4 | 616608 | T | C | 0.98 | -0.17 | 0.037 | 9.70E-06 | 175554 |
| rs1531583 |  | 4 | 744972 | T | G | 0.046 | 0.11 | 0.015 | 1.20E-12 | 231420 |
| rs35654957 |  | 4 | 1010077 | T | C | 0.63 | -0.03 | 0.0067 | 7.50E-06 | 231420 |
| 4_1309901_C_G |  | 4 | 1309901 | C | G | 0.97 | 0.068 | 0.021 | 1.50E-03 | 231420 |
| rs56337234 |  | 4 | 1784403 | T | C | 0.5 | -0.057 | 0.0066 | 1.40E-17 | 231420 |
| rs362307 |  | 4 | 3241845 | T | C | 0.077 | 0.074 | 0.012 | 1.10E-09 | 231420 |
| rs1801212 |  | 4 | 6302519 | A | G | 0.71 | 0.091 | 0.007 | 3.60E-38 | 231420 |
| rs10937721 |  | 4 | 6306763 | C | G | 0.59 | 0.087 | 0.0065 | 1.60E-40 | 231420 |
| rs12640250 |  | 4 | 17792869 | A | C | 0.29 | -0.039 | 0.0071 | 4.50E-08 | 231420 |
| rs10938398 |  | 4 | 45186139 | A | G | 0.43 | 0.044 | 0.0064 | 4.90E-12 | 231420 |
| rs2102278 |  | 4 | 52818664 | A | G | 0.68 | -0.038 | 0.0069 | 4.50E-08 | 231419 |
| rs17086692 |  | 4 | 53134293 | T | G | 0.31 | -0.028 | 0.0069 | 7.10E-05 | 231420 |
| rs114447556 |  | 4 | 53207093 | T | C | 0.084 | 0.06 | 0.012 | 7.70E-07 | 231420 |
| rs138641407 |  | 4 | 83578271 | A | G | 0.34 | 0.042 | 0.0067 | 5.70E-10 | 231420 |
| rs993380 |  | 4 | 83584496 | A | G | 0.34 | 0.042 | 0.0067 | 7.60E-10 | 231420 |
| rs1903002 |  | 4 | 89740894 | C | G | 0.5 | -0.036 | 0.0064 | 3.00E-08 | 231420 |
| rs6821438 |  | 4 | 95091911 | A | G | 0.53 | 0.042 | 0.0063 | 5.40E-11 | 231420 |
| rs1580278 |  | 4 | 104140848 | A | C | 0.53 | -0.041 | 0.0064 | 2.90E-10 | 231420 |
| rs2706785 |  | 4 | 122660250 | A | G | 0.95 | -0.062 | 0.015 | 6.00E-05 | 231420 |
| rs11098676 |  | 4 | 123833154 | T | C | 0.22 | -0.034 | 0.0077 | 1.50E-05 | 231420 |
| rs1296328 |  | 4 | 137083193 | A | C | 0.45 | 0.035 | 0.0064 | 4.30E-08 | 231420 |
| rs7669833 |  | 4 | 153513369 | A | T | 0.3 | -0.054 | 0.007 | 1.80E-14 | 231420 |
| rs28819812 |  | 4 | 157652753 | A | C | 0.32 | -0.04 | 0.0072 | 2.70E-08 | 187437 |
| rs58730668 |  | 4 | 185717759 | T | C | 0.86 | 0.068 | 0.0092 | 1.00E-13 | 231420 |
| rs3845281 |  | 5 | 14610134 | A | G | 0.096 | -0.048 | 0.011 | 1.40E-05 | 231420 |
| rs17250977 |  | 5 | 14753745 | A | G | 0.96 | -0.12 | 0.018 | 3.70E-12 | 231420 |
| rs6885132 |  | 5 | 14768092 | C | G | 0.9 | 0.078 | 0.011 | 9.50E-13 | 231420 |
| rs76549217 |  | 5 | 14768766 | T | C | 0.029 | 0.1 | 0.02 | 4.60E-07 | 231420 |
| rs1061813 |  | 5 | 14847331 | A | G | 0.54 | -0.036 | 0.0064 | 2.10E-08 | 231420 |
| rs62368490 |  | 5 | 44534364 | T | C | 0.031 | 0.078 | 0.02 | 1.20E-04 | 231109 |
| rs6884702 |  | 5 | 44682589 | A | G | 0.61 | -0.038 | 0.0065 | 5.80E-09 | 231109 |
| rs17261179 |  | 5 | 51791225 | T | C | 0.52 | 0.034 | 0.0063 | 8.20E-08 | 231420 |
| rs3811978 |  | 5 | 52100489 | A | G | 0.83 | -0.053 | 0.0085 | 4.20E-10 | 231420 |
| rs62357230 |  | 5 | 52315682 | A | G | 0.034 | 0.074 | 0.018 | 6.70E-05 | 231420 |
| rs62370480 |  | 5 | 52774510 | A | G | 0.22 | 0.039 | 0.0076 | 3.70E-07 | 231420 |
| 5_53271420_A_G |  | 5 | 53271420 | A | G | 0.69 | 0.051 | 0.0069 | 2.10E-13 | 231420 |
| rs279744 |  | 5 | 53412620 | A | C | 0.31 | -0.037 | 0.0069 | 1.30E-07 | 231420 |
| rs465002 |  | 5 | 55808475 | T | C | 0.74 | 0.073 | 0.0073 | 3.80E-23 | 231420 |
| rs2431115 |  | 5 | 55848669 | A | G | 0.4 | 0.026 | 0.0065 | 6.60E-05 | 231420 |
| rs9687832 |  | 5 | 55861595 | A | G | 0.2 | 0.067 | 0.008 | 4.90E-17 | 231420 |
| rs96844 |  | 5 | 56196604 | A | G | 0.74 | -0.038 | 0.0072 | 1.70E-07 | 231420 |
| 5_67714246_A_G |  | 5 | 67714246 | A | G | 0.59 | -0.035 | 0.0065 | 1.00E-07 | 231420 |
| 5_75003678_C_T |  | 5 | 75003678 | T | C | 0.61 | 0.053 | 0.0065 | 3.30E-16 | 231420 |
| rs4457053 |  | 5 | 76424949 | A | G | 0.7 | -0.059 | 0.0069 | 1.40E-17 | 231420 |
| rs10042338 | 1 | 5 | 78431077 | C | G | 0.35 | -0.046 | 0.0066 | 3.90E-12 | 231420 |
| rs7719891 |  | 5 | 86577352 | A | G | 0.74 | -0.04 | 0.0072 | 2.90E-08 | 231420 |
| rs115246607 | 1 | 5 | 102461890 | T | C | 0.95 | -0.17 | 0.015 | 5.20E-29 | 231420 |
| rs10077431 |  | 5 | 112927686 | A | C | 0.22 | -0.021 | 0.0079 | 8.40E-03 | 231420 |
| rs244665 |  | 5 | 133414622 | A | G | 0.7 | 0.03 | 0.007 | 2.30E-05 | 231420 |
| rs329122 |  | 5 | 133864599 | A | G | 0.43 | 0.037 | 0.0064 | 9.20E-09 | 231420 |
| rs3934712 |  | 5 | 157928196 | T | C | 0.79 | -0.039 | 0.0079 | 6.30E-07 | 231420 |
| rs112498319 |  | 6 | 7035734 | A | C | 0.59 | -0.041 | 0.0064 | 1.40E-10 | 231420 |
| rs9379084 |  | 6 | 7231843 | A | G | 0.11 | -0.097 | 0.011 | 2.30E-20 | 231420 |
| rs9505097 |  | 6 | 7255650 | T | C | 0.2 | -0.041 | 0.0081 | 3.30E-07 | 231420 |
| 6_20679709_A_G |  | 6 | 20679709 | A | G | 0.73 | -0.14 | 0.007 | 3.00E-87 | 231420 |
| 6_31136453_A_G |  | 6 | 31136453 | A | G | 0.26 | -0.052 | 0.0072 | 8.80E-13 | 231109 |
| rs2244020 |  | 6 | 31347451 | A | G | 0.65 | -0.02 | 0.0066 | 2.20E-03 | 231109 |
| rs115884658 |  | 6 | 31864538 | A | G | 0.029 | 0.13 | 0.02 | 1.30E-10 | 230308 |
| rs601945 |  | 6 | 32573415 | A | G | 0.82 | -0.08 | 0.0085 | 2.70E-21 | 231109 |
| rs9271771 | 0.938 | 6 | 32594274 | T | C | 0.22 | -0.065 | 0.0082 | 1.30E-15 | 225277 |
| rs77136196 |  | 6 | 34247047 | T | C | 0.041 | 0.086 | 0.017 | 2.00E-07 | 231420 |
| rs2233632 |  | 6 | 34524698 | T | C | 0.69 | 0.024 | 0.0069 | 6.10E-04 | 231420 |
| rs1535500 |  | 6 | 39284050 | T | G | 0.49 | 0.026 | 0.0063 | 4.70E-05 | 231420 |
| rs34298980 |  | 6 | 40409243 | T | C | 0.5 | 0.04 | 0.0066 | 1.20E-09 | 231420 |
| rs11967262 |  | 6 | 43760327 | C | G | 0.51 | -0.041 | 0.0064 | 1.40E-10 | 231420 |
| rs6458354 |  | 6 | 43814190 | T | C | 0.71 | -0.051 | 0.007 | 3.70E-13 | 231420 |
| rs3798519 |  | 6 | 50788778 | A | C | 0.82 | -0.058 | 0.0082 | 1.10E-12 | 231420 |
| rs2465043 |  | 6 | 51180765 | A | G | 0.36 | -0.032 | 0.0066 | 2.00E-06 | 231420 |
| 6_107431688_A_G |  | 6 | 107431688 | A | G | 0.33 | -0.039 | 0.0068 | 1.00E-08 | 231420 |
| rs11759026 |  | 6 | 126792095 | A | G | 0.77 | -0.067 | 0.0075 | 1.30E-18 | 231420 |
| rs2800733 |  | 6 | 127416930 | A | G | 0.72 | 0.051 | 0.007 | 3.70E-13 | 231420 |
| rs2246012 |  | 6 | 131898208 | T | C | 0.83 | -0.037 | 0.0085 | 1.30E-05 | 231420 |
| rs9494624 |  | 6 | 137300960 | A | G | 0.29 | 0.041 | 0.007 | 7.60E-09 | 231420 |
| rs2982521 |  | 6 | 139835329 | A | T | 0.38 | 0.034 | 0.0065 | 3.00E-07 | 231420 |
| rs616279 |  | 6 | 140249466 | A | G | 0.73 | 0.036 | 0.0072 | 6.30E-07 | 231109 |
| rs539298 | 1 | 6 | 160770360 | A | G | 0.52 | 0.039 | 0.0063 | 1.10E-09 | 231420 |
| rs4709746 |  | 6 | 164133001 | T | C | 0.13 | -0.056 | 0.0096 | 5.00E-09 | 231420 |
| rs17168486 |  | 7 | 14898282 | T | C | 0.18 | 0.069 | 0.0083 | 6.90E-17 | 231420 |
| rs10228066 |  | 7 | 15063569 | T | C | 0.54 | 0.066 | 0.0063 | 1.90E-25 | 231420 |
| rs2908334 |  | 7 | 15206239 | T | C | 0.63 | 0.019 | 0.0066 | 3.50E-03 | 231420 |
| rs78840640 |  | 7 | 23434606 | C | G | 0.98 | -0.12 | 0.022 | 2.00E-07 | 231420 |
| rs4279506 |  | 7 | 23512896 | C | G | 0.39 | -0.039 | 0.0066 | 5.70E-09 | 231420 |
| rs1708302 |  | 7 | 28198677 | T | C | 0.49 | -0.092 | 0.0063 | 4.20E-48 | 231420 |
| rs917195 |  | 7 | 30728452 | T | C | 0.23 | -0.051 | 0.0077 | 5.60E-11 | 231420 |
| rs878521 |  | 7 | 44255643 | A | G | 0.25 | 0.057 | 0.0074 | 1.60E-14 | 231420 |
| rs116913033 |  | 7 | 44365549 | T | C | 0.17 | -0.045 | 0.0088 | 3.30E-07 | 231420 |
| rs56376556 |  | 7 | 102038318 | T | C | 0.053 | 0.079 | 0.015 | 2.20E-07 | 231420 |
| rs11496066 |  | 7 | 102486254 | T | C | 0.82 | 0.047 | 0.0083 | 1.20E-08 | 231420 |
| rs62482405 |  | 7 | 102987583 | T | G | 0.92 | -0.056 | 0.012 | 1.60E-06 | 231420 |
| rs39328 |  | 7 | 103444978 | T | C | 0.43 | 0.036 | 0.0064 | 3.00E-08 | 231420 |
| rs6976111 |  | 7 | 117495667 | A | C | 0.31 | 0.042 | 0.0073 | 1.50E-08 | 187437 |
| rs2268382 |  | 7 | 130027037 | A | C | 0.67 | -0.028 | 0.0067 | 4.30E-05 | 231420 |
| rs1562396 |  | 7 | 130457914 | A | G | 0.68 | -0.058 | 0.0069 | 7.60E-17 | 231420 |
| rs9648716 |  | 7 | 140612163 | A | T | 0.86 | -0.032 | 0.0092 | 4.40E-04 | 231420 |
| rs62492368 |  | 7 | 150537635 | A | G | 0.31 | 0.044 | 0.0069 | 1.50E-10 | 231420 |
| rs6459733 |  | 7 | 156930550 | C | G | 0.33 | -0.058 | 0.0068 | 3.90E-17 | 231420 |
| rs17689007 |  | 8 | 9974824 | A | G | 0.47 | -0.048 | 0.0064 | 1.70E-13 | 231109 |
| rs57327348 |  | 8 | 10808687 | A | T | 0.78 | 0.053 | 0.0079 | 2.10E-11 | 231109 |
| 8_19830921_C_T |  | 8 | 19830921 | T | C | 0.12 | -0.07 | 0.0098 | 8.70E-13 | 231420 |
| rs10954772 |  | 8 | 30863938 | T | C | 0.31 | 0.041 | 0.0068 | 2.30E-09 | 231420 |
| rs12681990 |  | 8 | 36859186 | T | C | 0.83 | -0.034 | 0.0087 | 9.90E-05 | 231420 |
| rs13262861 |  | 8 | 41508577 | A | C | 0.17 | -0.094 | 0.0087 | 1.80E-27 | 231420 |
| rs4736819 |  | 8 | 41509915 | T | C | 0.55 | 0.064 | 0.0064 | 2.10E-23 | 231420 |
| rs148766658 |  | 8 | 41552046 | T | C | 0.96 | -0.11 | 0.017 | 7.90E-11 | 231420 |
| rs11786992 |  | 8 | 95685147 | A | C | 0.64 | 0.04 | 0.0066 | 1.70E-09 | 231420 |
| rs10097617 |  | 8 | 95961626 | T | C | 0.48 | 0.051 | 0.0063 | 1.10E-15 | 231420 |
| rs187936726 |  | 8 | 96092422 | A | G | 0.98 | -0.12 | 0.022 | 2.30E-08 | 231420 |
| rs4236802 | 1 | 8 | 110127292 | T | C | 0.47 | -0.035 | 0.0063 | 3.40E-08 | 231420 |
| 8_118185025_A_G |  | 8 | 118185025 | A | G | 0.31 | -0.11 | 0.0069 | 6.30E-55 | 231420 |
| rs80244329 |  | 8 | 118404672 | A | G | 0.022 | -0.11 | 0.024 | 7.70E-06 | 231420 |
| rs17772814 |  | 8 | 128711742 | A | G | 0.085 | -0.078 | 0.013 | 5.00E-10 | 231420 |
| rs1561927 |  | 8 | 129568078 | T | C | 0.73 | -0.043 | 0.0071 | 1.90E-09 | 231420 |
| rs4265220 | 1 | 8 | 145508000 | A | G | 0.38 | 0.051 | 0.0067 | 4.40E-14 | 231420 |
| rs12719778 |  | 8 | 145879883 | T | C | 0.54 | 0.039 | 0.0064 | 2.10E-09 | 231420 |
| rs510807 |  | 9 | 3965689 | A | C | 0.49 | 0.028 | 0.0064 | 1.70E-05 | 231420 |
| rs16920974 | 1 | 9 | 4244218 | C | G | 0.014 | -0.14 | 0.029 | 8.60E-07 | 231420 |
| rs10974438 |  | 9 | 4291928 | A | C | 0.64 | -0.051 | 0.0066 | 1.60E-14 | 231420 |
| rs7022807 |  | 9 | 19067833 | A | G | 0.6 | -0.04 | 0.0064 | 3.60E-10 | 231420 |
| rs7867635 |  | 9 | 20241069 | T | C | 0.59 | -0.036 | 0.0065 | 4.10E-08 | 231420 |
| rs7847880 |  | 9 | 20662703 | T | C | 0.16 | -0.044 | 0.0089 | 6.80E-07 | 231420 |
| rs1412830 |  | 9 | 22043612 | T | C | 0.37 | -0.053 | 0.0066 | 1.20E-15 | 231420 |
| rs76011118 |  | 9 | 22133773 | A | G | 0.034 | 0.19 | 0.019 | 1.70E-22 | 231420 |
| rs10811660 |  | 9 | 22134068 | A | G | 0.17 | -0.16 | 0.0086 | 6.60E-79 | 231420 |
| rs10757283 |  | 9 | 22134172 | T | C | 0.43 | 0.021 | 0.0064 | 9.40E-04 | 231420 |
| rs1333052 |  | 9 | 22157908 | A | C | 0.66 | 0.018 | 0.0067 | 9.10E-03 | 231420 |
| rs1575972 |  | 9 | 22301092 | A | T | 0.033 | -0.077 | 0.018 | 2.00E-05 | 231420 |
| rs1412234 |  | 9 | 28410683 | T | C | 0.68 | -0.043 | 0.0068 | 2.50E-10 | 231420 |
| rs12001437 |  | 9 | 34074476 | T | C | 0.63 | -0.041 | 0.0065 | 3.70E-10 | 231420 |
| rs11137820 |  | 9 | 81359113 | C | G | 0.58 | 0.035 | 0.0064 | 3.60E-08 | 231420 |
| rs17791513 |  | 9 | 81905590 | A | G | 0.93 | 0.1 | 0.013 | 2.90E-14 | 231420 |
| rs2796441 |  | 9 | 84308948 | A | G | 0.41 | -0.066 | 0.0065 | 8.50E-24 | 231420 |
| rs55653563 |  | 9 | 97001682 | A | C | 0.73 | 0.043 | 0.0072 | 3.20E-09 | 231420 |
| rs12236906 |  | 9 | 97497494 | T | C | 0.99 | 0.14 | 0.03 | 3.90E-06 | 231420 |
| 9_136149229_C_T |  | 9 | 136149229 | T | C | 0.67 | -0.046 | 0.0067 | 5.40E-12 | 231420 |
| rs78403475 |  | 9 | 139235606 | C | G | 0.1 | -0.035 | 0.011 | 1.50E-03 | 231420 |
| rs28505901 |  | 9 | 139241030 | A | G | 0.25 | -0.076 | 0.0081 | 2.60E-21 | 212857 |
| rs11793035 |  | 9 | 139507212 | T | C | 0.67 | -0.033 | 0.0074 | 1.10E-05 | 212857 |
| 10_12307894_C_T |  | 10 | 12307894 | T | C | 0.22 | 0.09 | 0.0076 | 3.70E-32 | 231420 |
| rs177045 |  | 10 | 71321279 | A | G | 0.68 | -0.05 | 0.0069 | 1.00E-12 | 231420 |
| rs41277236 |  | 10 | 71332301 | T | C | 0.043 | 0.096 | 0.017 | 6.40E-09 | 231420 |
| 10_71466578_G_T |  | 10 | 71466578 | T | G | 0.3 | -0.052 | 0.0069 | 6.30E-14 | 231420 |
| rs2633310 |  | 10 | 75594050 | T | G | 0.44 | -0.026 | 0.0064 | 5.10E-05 | 231420 |
| rs703972 |  | 10 | 80952826 | C | G | 0.47 | -0.071 | 0.0064 | 2.50E-28 | 231420 |
| rs1317617 |  | 10 | 81096589 | A | G | 0.2 | -0.035 | 0.008 | 1.10E-05 | 231420 |
| rs11202627 |  | 10 | 89769340 | T | C | 0.15 | 0.044 | 0.0089 | 7.60E-07 | 231420 |
| rs7078559 |  | 10 | 93924663 | T | C | 0.58 | 0.049 | 0.0064 | 1.70E-14 | 231420 |
| rs10882101 |  | 10 | 94462427 | T | C | 0.59 | 0.11 | 0.0064 | 1.60E-62 | 231420 |
| rs1112718 |  | 10 | 94479107 | A | G | 0.6 | 0.11 | 0.0065 | 2.90E-59 | 231420 |
| rs11591741 |  | 10 | 101976501 | C | G | 0.43 | -0.03 | 0.0064 | 3.70E-06 | 231420 |
| rs11196150 | 0.896 | 10 | 114678843 | A | G | 0.49 | 0.018 | 0.0064 | 4.50E-03 | 231420 |
| rs184509201 |  | 10 | 114740337 | C | G | 0.98 | 0.18 | 0.026 | 2.80E-12 | 231420 |
| rs180988137 |  | 10 | 114751173 | A | G | 0.99 | -0.32 | 0.034 | 1.50E-21 | 231420 |
| rs78025551 |  | 10 | 114757956 | C | G | 0.85 | 0.15 | 0.0092 | 8.40E-63 | 231420 |
| rs34855922 |  | 10 | 114871594 | A | G | 0.72 | 0.034 | 0.0072 | 2.50E-06 | 231420 |
| rs4918796 |  | 10 | 114880342 | T | C | 0.78 | -0.068 | 0.0077 | 1.70E-18 | 231420 |
| rs72631105 |  | 10 | 122915345 | A | G | 0.19 | 0.044 | 0.0083 | 8.00E-08 | 231420 |
| rs2280141 |  | 10 | 124193181 | T | G | 0.52 | 0.047 | 0.0063 | 2.00E-13 | 231420 |
| rs12802972 |  | 11 | 1704596 | A | G | 0.43 | 0.03 | 0.0065 | 3.60E-06 | 231420 |
| rs11042596 |  | 11 | 2118860 | T | G | 0.34 | -0.036 | 0.0069 | 2.70E-07 | 231420 |
| rs4929965 |  | 11 | 2197286 | A | G | 0.38 | 0.07 | 0.0067 | 4.80E-25 | 231420 |
| rs4930091 |  | 11 | 2372356 | T | C | 0.24 | -0.029 | 0.0075 | 1.10E-04 | 231420 |
| rs2283164 |  | 11 | 2579163 | A | G | 0.95 | 0.066 | 0.015 | 1.40E-05 | 231420 |
| rs80102379 |  | 11 | 2634177 | T | G | 0.018 | -0.086 | 0.026 | 1.10E-03 | 231420 |
| rs231349 |  | 11 | 2672821 | T | C | 0.1 | 0.06 | 0.011 | 1.40E-08 | 231420 |
| rs231361 |  | 11 | 2691500 | A | G | 0.26 | 0.064 | 0.0074 | 6.00E-18 | 231420 |
| rs11023936 | 1 | 11 | 2755552 | T | C | 0.69 | 0.043 | 0.0071 | 2.10E-09 | 231420 |
| rs234853 |  | 11 | 2850828 | A | G | 0.75 | 0.029 | 0.0075 | 1.30E-04 | 231420 |
| 11_2857194_A_C |  | 11 | 2857194 | A | C | 0.57 | -0.093 | 0.0066 | 3.60E-44 | 231420 |
| rs2237897 |  | 11 | 2858546 | T | C | 0.046 | -0.19 | 0.017 | 1.80E-31 | 231420 |
| rs445084 |  | 11 | 2908754 | A | G | 0.64 | -0.032 | 0.0068 | 2.20E-06 | 231420 |
| rs141521721 |  | 11 | 14763828 | A | C | 0.024 | 0.12 | 0.021 | 2.80E-08 | 231420 |
| rs4923543 |  | 11 | 28534898 | A | G | 0.33 | 0.026 | 0.0067 | 1.00E-04 | 231420 |
| rs7943101 |  | 11 | 32460873 | T | C | 0.16 | 0.044 | 0.0087 | 4.20E-07 | 231420 |
| rs145678014 |  | 11 | 32927778 | T | G | 0.043 | -0.11 | 0.016 | 1.10E-11 | 231420 |
| rs286925 |  | 11 | 34642668 | A | G | 0.18 | 0.039 | 0.0083 | 2.70E-06 | 231420 |
| rs2767036 |  | 11 | 34982148 | A | C | 0.71 | -0.039 | 0.0069 | 2.50E-08 | 231420 |
| rs57635800 | 1 | 11 | 43878485 | A | G | 0.29 | 0.05 | 0.007 | 8.50E-13 | 231420 |
| rs7115753 |  | 11 | 45912013 | A | G | 0.45 | 0.038 | 0.0064 | 4.80E-09 | 231420 |
| 11_47529947_A_C |  | 11 | 47529947 | A | C | 0.41 | 0.037 | 0.0064 | 6.40E-09 | 231109 |
| rs7929543 |  | 11 | 49351026 | A | C | 0.91 | -0.048 | 0.011 | 2.30E-05 | 231109 |
| 11_61557803_C_T |  | 11 | 61557803 | T | C | 0.65 | 0.028 | 0.0066 | 2.00E-05 | 231420 |
| 11_64031241_C_T |  | 11 | 64031241 | T | C | 0.065 | 0.062 | 0.013 | 1.90E-06 | 231420 |
| rs1144928 | 1 | 11 | 65301410 | A | G | 0.8 | -0.061 | 0.008 | 2.50E-14 | 231420 |
| rs61881115 |  | 11 | 68997225 | A | G | 0.16 | -0.043 | 0.0088 | 6.80E-07 | 231420 |
| rs11820019 |  | 11 | 69448758 | T | C | 0.97 | 0.14 | 0.021 | 1.00E-11 | 231420 |
| rs77464186 |  | 11 | 72460398 | A | C | 0.84 | 0.11 | 0.0088 | 2.30E-33 | 231420 |
| rs10830963 |  | 11 | 92708710 | C | G | 0.72 | -0.099 | 0.0071 | 1.50E-43 | 231420 |
| rs57235767 |  | 11 | 93013531 | T | C | 0.29 | -0.046 | 0.007 | 6.60E-11 | 231420 |
| rs10893829 |  | 11 | 128042575 | T | C | 0.85 | 0.057 | 0.0091 | 2.70E-10 | 231420 |
| 11_128234144_A_G |  | 11 | 128234144 | A | G | 0.28 | 0.045 | 0.007 | 2.00E-10 | 231420 |
| rs67232546 |  | 11 | 128398938 | T | C | 0.21 | 0.056 | 0.008 | 1.40E-12 | 231420 |
| rs112595469 |  | 11 | 128583975 | T | C | 0.028 | 0.093 | 0.02 | 4.40E-06 | 231420 |
| 12 |  | 12 | 4031104 | T | C | 0.2 | -0.041 | 0.0082 | 4.90E-07 | 231420 |
| 12 |  | 12 | 4300172 | T | C | 0.82 | -0.047 | 0.0083 | 1.30E-08 | 231420 |
| 12 |  | 12 | 4376089 | T | C | 0.79 | -0.058 | 0.008 | 3.30E-13 | 231420 |
| 12 |  | 12 | 4384696 | T | C | 0.087 | -0.12 | 0.012 | 2.50E-22 | 231420 |
| 12 |  | 12 | 4384844 | T | G | 0.98 | 0.48 | 0.027 | 5.30E-70 | 231420 |
| 12 |  | 12 | 4399050 | A | G | 0.74 | -0.057 | 0.0074 | 2.00E-14 | 231420 |
| 12 |  | 12 | 4406281 | A | G | 0.53 | -0.049 | 0.0065 | 9.80E-14 | 231419 |
| 12 |  | 12 | 12871099 | T | G | 0.77 | -0.044 | 0.0081 | 3.50E-08 | 231420 |
| 12 | 1 | 12 | 26456188 | A | T | 0.75 | -0.047 | 0.0073 | 1.10E-10 | 231420 |
| 12 |  | 12 | 27965150 | T | C | 0.19 | -0.074 | 0.0081 | 2.50E-20 | 231420 |
| 12 |  | 12 | 66221060 | A | T | 0.9 | -0.11 | 0.011 | 2.00E-25 | 231420 |
| 12 | 1 | 12 | 66360164 | T | C | 0.49 | 0.054 | 0.0063 | 2.20E-17 | 231420 |
| 12 |  | 12 | 71522953 | C | G | 0.43 | -0.049 | 0.0064 | 3.20E-14 | 231420 |
| 12 |  | 12 | 93978504 | T | G | 0.22 | 0.021 | 0.0076 | 6.50E-03 | 231420 |
| 12 |  | 12 | 95928560 | T | C | 0.54 | 0.035 | 0.0063 | 4.40E-08 | 231420 |
| 12 |  | 12 | 97848775 | A | G | 0.93 | 0.073 | 0.013 | 2.20E-08 | 231420 |
| 12 |  | 12 | 108629780 | A | G | 0.26 | -0.05 | 0.0073 | 1.10E-11 | 231420 |
| 12 |  | 12 | 118412373 | A | G | 0.14 | 0.054 | 0.0092 | 3.50E-09 | 231420 |
| 12 |  | 12 | 118489636 | A | T | 0.83 | 0.04 | 0.0086 | 3.90E-06 | 231420 |
| 12 |  | 12 | 121297815 | A | G | 0.075 | 0.08 | 0.012 | 2.80E-11 | 231420 |
| 12 |  | 12 | 121380541 | A | G | 0.033 | -0.12 | 0.019 | 7.00E-11 | 231420 |
| 12_121416864_C_T |  | 12 | 121416864 | T | C | 0.03 | 0.16 | 0.019 | 8.90E-17 | 231420 |
| 12 |  | 12 | 121432117 | C | G | 0.31 | -0.062 | 0.0069 | 3.80E-19 | 231420 |
| 12 |  | 12 | 121501461 | A | C | 0.044 | 0.093 | 0.016 | 7.20E-09 | 231420 |
| 12 |  | 12 | 121686929 | A | G | 0.32 | -0.035 | 0.0068 | 4.00E-07 | 231420 |
| 12 |  | 12 | 123450765 | C | G | 0.78 | 0.049 | 0.0077 | 2.20E-10 | 231420 |
| 12 | 1 | 12 | 124469738 | A | G | 0.67 | 0.034 | 0.0067 | 6.30E-07 | 231420 |
| 12 |  | 12 | 124509177 | A | G | 0.61 | 0.032 | 0.0065 | 1.30E-06 | 231420 |
| 12 |  | 12 | 133069698 | A | G | 0.33 | 0.049 | 0.007 | 2.40E-12 | 231420 |
| rs34584161 |  | 13 | 26776999 | A | G | 0.76 | 0.048 | 0.0075 | 2.90E-10 | 231420 |
| rs11842871 |  | 13 | 31042452 | T | G | 0.27 | -0.042 | 0.0073 | 1.50E-08 | 231420 |
| 13_33554302_A_G |  | 13 | 33554302 | A | G | 0.83 | -0.053 | 0.0086 | 6.80E-10 | 231420 |
| rs963740 |  | 13 | 51096095 | A | T | 0.71 | 0.039 | 0.007 | 2.60E-08 | 231420 |
| rs9537803 |  | 13 | 58366634 | T | C | 0.72 | -0.034 | 0.007 | 1.30E-06 | 231420 |
| rs9569864 |  | 13 | 58965435 | T | C | 0.18 | -0.048 | 0.0084 | 8.50E-09 | 231420 |
| rs9563615 |  | 13 | 59077406 | A | T | 0.71 | 0.042 | 0.007 | 3.90E-09 | 231420 |
| 13_80717156_A_G |  | 13 | 80717156 | A | G | 0.28 | -0.083 | 0.0071 | 5.70E-31 | 231420 |
| rs7994469 | 1 | 13 | 91940484 | A | G | 0.25 | -0.037 | 0.0073 | 6.30E-07 | 231420 |
| rs7987740 |  | 13 | 109947213 | T | C | 0.61 | 0.036 | 0.0065 | 4.10E-08 | 231420 |
| rs4771648 |  | 13 | 110431626 | A | G | 0.33 | -0.037 | 0.0068 | 8.90E-08 | 231420 |
| rs17122772 |  | 14 | 23288935 | C | G | 0.77 | -0.043 | 0.0077 | 2.00E-08 | 231420 |
| rs17522122 |  | 14 | 33302882 | T | G | 0.47 | 0.038 | 0.0064 | 4.00E-09 | 231420 |
| rs8017808 |  | 14 | 38848419 | T | G | 0.26 | -0.041 | 0.0073 | 2.60E-08 | 231420 |
| rs17836088 |  | 14 | 79932041 | C | G | 0.22 | 0.058 | 0.0077 | 9.70E-14 | 231420 |
| rs8010382 |  | 14 | 91963722 | A | G | 0.58 | -0.038 | 0.0066 | 8.10E-09 | 231420 |
| rs3783395 | 1 | 14 | 103895020 | T | C | 0.65 | 0.037 | 0.0067 | 5.30E-08 | 231420 |
| rs8032939 |  | 15 | 38834033 | T | C | 0.75 | -0.043 | 0.0074 | 8.40E-09 | 231420 |
| rs34715063 |  | 15 | 38873115 | T | C | 0.88 | -0.076 | 0.01 | 3.30E-14 | 231420 |
| rs67839313 |  | 15 | 40619724 | T | C | 0.89 | -0.03 | 0.01 | 3.10E-03 | 231420 |
| rs11070332 |  | 15 | 41809205 | A | G | 0.36 | 0.049 | 0.0066 | 1.30E-13 | 231420 |
| rs2456530 |  | 15 | 53091553 | T | C | 0.13 | 0.056 | 0.0096 | 4.70E-09 | 231420 |
| rs117483894 |  | 15 | 57456802 | A | G | 0.96 | -0.093 | 0.017 | 3.90E-08 | 231420 |
| rs8037894 |  | 15 | 62394264 | C | G | 0.43 | -0.047 | 0.0064 | 3.70E-13 | 231420 |
| 15_63871292_C_T |  | 15 | 63871292 | T | C | 0.54 | -0.039 | 0.0063 | 7.00E-10 | 231420 |
| 15_68080886_A_T |  | 15 | 68080886 | A | T | 0.64 | 0.039 | 0.0066 | 6.20E-09 | 231420 |
| rs13737 |  | 15 | 75932129 | T | G | 0.24 | -0.046 | 0.0075 | 7.30E-10 | 231420 |
| rs1005752 |  | 15 | 77818128 | A | C | 0.72 | 0.079 | 0.007 | 5.70E-29 | 231420 |
| 15_80432222_A_G |  | 15 | 80432222 | A | G | 0.35 | -0.031 | 0.0067 | 4.90E-06 | 231420 |
| rs4932265 |  | 15 | 90423293 | T | C | 0.27 | 0.065 | 0.0071 | 7.20E-20 | 231420 |
| rs12910825 |  | 15 | 91511260 | A | G | 0.64 | -0.053 | 0.0066 | 2.40E-15 | 231420 |
| 16_295795_C_T |  | 16 | 295795 | T | C | 0.82 | 0.061 | 0.0085 | 7.00E-13 | 231420 |
| rs3751837 |  | 16 | 3583173 | T | C | 0.22 | 0.044 | 0.0077 | 1.70E-08 | 231420 |
| rs10499 | 1 | 16 | 28915527 | A | G | 0.64 | -0.037 | 0.0066 | 2.80E-08 | 231420 |
| rs11642430 |  | 16 | 30045789 | C | G | 0.6 | -0.042 | 0.0065 | 1.20E-10 | 231420 |
| rs4281707 |  | 16 | 53501946 | A | G | 0.46 | -0.035 | 0.0064 | 3.60E-08 | 231420 |
| rs11642015 | 1 | 16 | 53802494 | T | C | 0.42 | 0.12 | 0.0064 | 4.40E-78 | 231420 |
| rs862320 |  | 16 | 69651866 | T | C | 0.42 | -0.042 | 0.0064 | 5.10E-11 | 231420 |
| rs72802342 |  | 16 | 75234872 | A | C | 0.077 | -0.13 | 0.012 | 1.30E-27 | 231420 |
| 16_81534790_C_T |  | 16 | 81534790 | T | C | 0.3 | 0.053 | 0.0069 | 2.10E-14 | 231420 |
| rs12920022 |  | 16 | 89564055 | A | T | 0.16 | 0.053 | 0.009 | 2.90E-09 | 231420 |
| rs12103726 | 0.564 | 17 | 2192839 | T | C | 0.26 | 0.019 | 0.0072 | 8.70E-03 | 231420 |
| rs1043246 |  | 17 | 3828086 | C | G | 0.84 | -0.059 | 0.0096 | 6.00E-10 | 231420 |
| rs3826482 |  | 17 | 3860356 | A | T | 0.58 | 0.036 | 0.0065 | 4.80E-08 | 231420 |
| rs1377807 |  | 17 | 4045440 | C | G | 0.31 | 0.057 | 0.0068 | 5.70E-17 | 231420 |
| rs1641523 |  | 17 | 7549681 | T | C | 0.57 | -0.028 | 0.0064 | 1.30E-05 | 231420 |
| rs62059712 |  | 17 | 7740170 | T | C | 0.92 | 0.046 | 0.012 | 1.20E-04 | 231420 |
| rs62067560 | 1 | 17 | 9785427 | A | G | 0.32 | 0.038 | 0.0068 | 2.80E-08 | 231420 |
| 17_17661802_A_G |  | 17 | 17661802 | A | G | 0.32 | 0.048 | 0.0068 | 3.90E-12 | 231420 |
| rs71372253 |  | 17 | 29413019 | T | C | 0.94 | -0.073 | 0.013 | 4.30E-08 | 231420 |
| rs10962 |  | 17 | 36046451 | C | G | 0.23 | 0.043 | 0.0079 | 4.30E-08 | 231420 |
| rs2189301 |  | 17 | 36063685 | A | G | 0.13 | -0.06 | 0.0097 | 6.50E-10 | 231420 |
| rs10908278 |  | 17 | 36099952 | A | T | 0.52 | -0.074 | 0.0064 | 3.10E-30 | 231420 |
| rs34855406 |  | 17 | 40731411 | C | G | 0.28 | 0.05 | 0.0071 | 3.20E-12 | 231420 |
| rs9911983 |  | 17 | 45885756 | T | C | 0.57 | 0.024 | 0.0064 | 2.30E-04 | 231420 |
| rs35895680 |  | 17 | 47060322 | A | C | 0.32 | -0.055 | 0.0069 | 3.80E-15 | 231420 |
| rs302864 |  | 17 | 56757584 | A | G | 0.089 | 0.056 | 0.011 | 8.90E-07 | 231420 |
| rs2727301 |  | 17 | 61965043 | T | C | 0.75 | 0.035 | 0.0074 | 1.90E-06 | 231420 |
| rs60276348 |  | 17 | 62203304 | T | C | 0.14 | 0.052 | 0.0095 | 2.90E-08 | 231420 |
| rs11657492 |  | 17 | 65648427 | T | G | 0.9 | -0.058 | 0.011 | 7.10E-08 | 231420 |
| rs61676547 |  | 17 | 65892507 | C | G | 0.19 | 0.055 | 0.0081 | 1.00E-11 | 231420 |
| rs7240767 |  | 18 | 7070642 | T | C | 0.62 | -0.037 | 0.0065 | 2.00E-08 | 231420 |
| rs62080313 |  | 18 | 36278709 | T | C | 0.88 | -0.056 | 0.0098 | 9.10E-09 | 231420 |
| rs72926932 |  | 18 | 53050646 | A | C | 0.92 | -0.083 | 0.011 | 3.60E-13 | 231420 |
| rs9952111 | 1 | 18 | 53453113 | A | G | 0.16 | 0.034 | 0.0087 | 7.80E-05 | 231420 |
| rs17684074 |  | 18 | 54675384 | C | G | 0.26 | -0.041 | 0.0073 | 3.50E-08 | 231420 |
| rs9957145 |  | 18 | 56876228 | A | G | 0.17 | -0.05 | 0.0086 | 6.70E-09 | 231420 |
| rs523288 |  | 18 | 57848369 | A | T | 0.76 | -0.056 | 0.0074 | 7.50E-14 | 231420 |
| rs112297194 | 1 | 18 | 58056958 | A | G | 0.024 | -0.14 | 0.022 | 1.00E-10 | 231420 |
| rs10469140 |  | 18 | 60668270 | A | G | 0.52 | -0.029 | 0.0063 | 6.00E-06 | 231420 |
| rs12454712 |  | 18 | 60845884 | T | C | 0.61 | 0.049 | 0.0067 | 5.10E-13 | 231420 |
| rs7249758 |  | 19 | 4948862 | A | G | 0.2 | 0.045 | 0.008 | 1.20E-08 | 231420 |
| rs116953931 |  | 19 | 5224998 | A | G | 0.037 | 0.071 | 0.017 | 2.90E-05 | 231420 |
| rs75253922 |  | 19 | 7240848 | T | C | 0.81 | -0.046 | 0.0082 | 2.20E-08 | 231420 |
| rs4804833 |  | 19 | 7970635 | A | G | 0.39 | 0.047 | 0.0066 | 1.10E-12 | 231420 |
| rs3111316 |  | 19 | 13038415 | A | G | 0.59 | 0.046 | 0.0065 | 1.60E-12 | 231420 |
| rs8107974 |  | 19 | 19388500 | A | T | 0.92 | -0.093 | 0.012 | 6.30E-15 | 231420 |
| rs10406327 |  | 19 | 33890838 | C | G | 0.52 | 0.035 | 0.0064 | 4.60E-08 | 231420 |
| rs429358 |  | 19 | 45411941 | T | C | 0.85 | 0.08 | 0.0092 | 1.80E-18 | 231420 |
| rs10406431 |  | 19 | 46157019 | A | G | 0.56 | 0.059 | 0.0065 | 2.50E-19 | 231420 |
| rs2238689 |  | 19 | 46178661 | T | C | 0.58 | -0.05 | 0.0065 | 1.30E-14 | 231420 |
| 19_47569003_A_G |  | 19 | 47569003 | A | G | 0.67 | 0.046 | 0.0068 | 1.20E-11 | 231420 |
| rs13041756 |  | 20 | 21466795 | T | C | 0.89 | -0.058 | 0.01 | 1.30E-08 | 231420 |
| rs6515236 |  | 20 | 22435749 | A | C | 0.75 | 0.036 | 0.0073 | 8.40E-07 | 231420 |
| rs2268078 |  | 20 | 32596704 | A | G | 0.66 | 0.043 | 0.0067 | 2.90E-10 | 231420 |
| 20_39832628_C_T |  | 20 | 39832628 | T | C | 0.8 | -0.024 | 0.0079 | 2.40E-03 | 231420 |
| rs76811102 |  | 20 | 42905415 | T | C | 0.042 | 0.069 | 0.017 | 3.30E-05 | 231420 |
| rs4810426 |  | 20 | 43001721 | T | C | 0.11 | 0.082 | 0.011 | 6.90E-15 | 231420 |
| 20_43042364_C_T |  | 20 | 43042364 | T | C | 0.035 | 0.16 | 0.017 | 3.20E-20 | 231420 |
| rs11696357 |  | 20 | 43233649 | A | G | 0.93 | 0.053 | 0.014 | 1.40E-04 | 231420 |
| rs6063048 |  | 20 | 45598564 | A | G | 0.28 | -0.047 | 0.0071 | 5.80E-11 | 231420 |
| rs11699802 |  | 20 | 48832135 | T | C | 0.46 | -0.043 | 0.0064 | 2.50E-11 | 231420 |
| rs34454109 |  | 20 | 51223594 | A | T | 0.77 | 0.044 | 0.0076 | 8.80E-09 | 231420 |
| rs4810145 | 1 | 20 | 57396495 | T | C | 0.48 | -0.045 | 0.0064 | 4.40E-12 | 231420 |
| rs862016 |  | 20 | 57551099 | A | G | 0.92 | -0.055 | 0.012 | 4.90E-06 | 231420 |
| rs6011155 |  | 20 | 62450664 | T | C | 0.63 | 0.031 | 0.0066 | 3.80E-06 | 231420 |
| rs59944054 |  | 20 | 62693175 | A | G | 0.24 | 0.033 | 0.0079 | 2.90E-05 | 231420 |
| rs6518681 |  | 22 | 30609554 | A | G | 0.086 | -0.083 | 0.012 | 9.60E-13 | 231420 |
| rs117001013 |  | 22 | 32348841 | T | C | 0.088 | -0.065 | 0.011 | 1.50E-08 | 231420 |
| rs5758223 |  | 22 | 41489920 | A | G | 0.72 | 0.038 | 0.007 | 4.60E-08 | 231420 |
| rs738408 |  | 22 | 44324730 | T | C | 0.23 | 0.049 | 0.0076 | 1.80E-10 | 231420 |
| rs1801645 |  | 22 | 50356850 | T | C | 0.72 | -0.048 | 0.0074 | 1.50E-10 | 231420 |
| rs112915006 |  | 22 | 50604696 | A | G | 0.95 | -0.092 | 0.015 | 7.50E-10 | 231420 |

**Supplementary Table S2** Association of GRSs with T2D remission. Abbreviations: GRS, genetic risk scores; WCadjBMI, waist circumference adjusted for BMI; WHRadjBMI, waist-hip ratio adjusted for BMI; HIPadjBMI, hip adjusted for BMI

|  | **Complete and partial remitters vs. non-remitters (n = 9/8)** | | | **Complete vs. partial vs. non-remitters (n = 3/6/8)** | | |
| --- | --- | --- | --- | --- | --- | --- |
| **GRS** | **β** | **SE** | **P-value** | **β** | **SE** | **P-value** |
| **T2D (400 SNPs)** | 5.04 | 36.13 | 0.89 | -4.67 | 11.28 | 0.69 |
| **β-cell function (47 SNPs)** | -3.72 | 8.56 | 0.66 | -1.3 | 2.78 | 0.65 |
| **BMI (1,441 SNPs)** | 89.77 | 113.51 | 0.43 | 8.91 | 36.65 | 0.81 |
| **WCadjBMI (80 SNPs)** | 26.27 | 36.19 | 0.47 | 3.831 | 12.16 | 0.76 |
| **HIPadjBMI (98 SNPs)** | 224.2 | 129.7 | 0.08 | 26.677 | 12.9 | 0.07 |
| **WHRadjBMI (812 SNPs)** | 160.5 | 161.6 | 0.32 | 26.995 | 39.42 | 0.51 |

97 patients approached

- Non-remitters: 35
- Remitters: 62

77 were excluded

- Declined: 42
- No answer: 10
- Conversion of surgery: 4
- On liraglutide: 4
- Never had T2D: 2
- Active hypoglycemia: 2
- Contraindication for BIA: 1
- Mobility problems: 1
- Intestinal stoma: 1
- Lung cancer: 1
- Deceased: 1
- ESRD: 1
- LADA: 1
- No show: 3
- Insufficient data: 1
- Stroke: 1
- Use of insulin prior to the surgery: 1

Non-remitters: 10

Remitters: 10

**Supplementary Figure S1** Subject enrolment


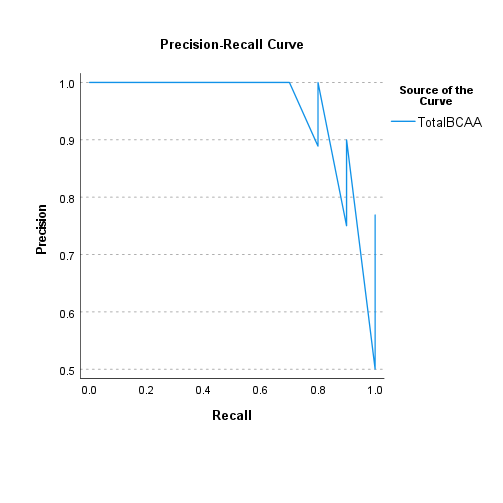

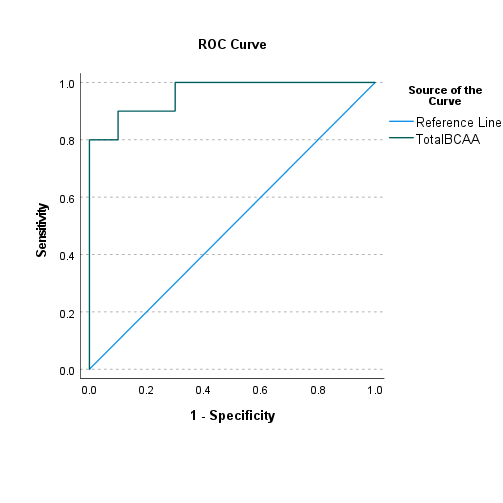


**Supplementary Figure S2** Receiver operating characteristic (ROC) curve determining the best cut-off value of AUC_0-180_ total BCAAs for being non-remitters after bariatric/metabolic surgery
